# Ni‐Catalyzed Borylation of Aryl Sulfoxides

**DOI:** 10.1002/chem.202100342

**Published:** 2021-05-06

**Authors:** Mingming Huang, Zhu Wu, Johannes Krebs, Alexandra Friedrich, Xiaoling Luo, Stephen A. Westcott, Udo Radius, Todd B. Marder

**Affiliations:** ^1^ Institut für Anorganische Chemie and Institute for Sustainable Chemistry & Catalysis with Boron Julius-Maximilians-Universität Würzburg Am Hubland 97074 Würzburg Germany; ^2^ Department of Chemistry & Biochemistry Mount Allison University Sackville NB E4L 1G8 Canada; ^3^ Chongqing Key Laboratory of Inorganic Functional Materials College of Chemistry Chongqing Normal University Chongqing 401331 China

**Keywords:** Boron, borylation, cross-coupling, N-heterocyclic carbenes, nickel

## Abstract

A nickel/*N*‐heterocyclic carbene (NHC) catalytic system has been developed for the borylation of aryl sulfoxides with B_2_(neop)_2_ (neop=neopentyl glycolato). A wide range of aryl sulfoxides with different electronic and steric properties were converted into the corresponding arylboronic esters in good yields. The regioselective borylation of unsymmetric diaryl sulfoxides was also feasible leading to borylation of the sterically less encumbered aryl substituent. Competition experiments demonstrated that an electron‐deficient aryl moiety reacts preferentially. The origin of the selectivity in the Ni‐catalyzed borylation of electronically biased unsymmetrical diaryl sulfoxide lies in the oxidative addition step of the catalytic cycle, as oxidative addition of methoxyphenyl 4‐(trifluoromethyl)phenyl sulfoxide to the Ni(0) complex occurs selectively to give the structurally characterized complex *trans*‐[Ni(ICy)_2_(4‐CF_3_‐C_6_H_4_){(SO)‐4‐MeO‐C_6_H_4_}] **4**. For complex **5**, the isomer *trans*‐[Ni(ICy)_2_(C_6_H_5_)(OSC_6_H_5_)] **5**‐**I** was structurally characterized in which the phenyl sulfinyl ligand is bound *via* the oxygen atom to nickel. In solution, the complex *trans*‐[Ni(ICy)_2_(C_6_H_5_)(OSC_6_H_5_)] **5**‐**I** is in equilibrium with the S‐bonded isomer *trans*‐[Ni(ICy)_2_(C_6_H_5_)(SOC_6_H_5_)] **5**, as shown by NMR spectroscopy. DFT calculations reveal that these isomers are separated by a mere 0.3 kJ/mol (M06/def2‐TZVP‐level of theory) and connected *via* a transition state *trans*‐[Ni(ICy)_2_(C_6_H_5_)(η^2^‐{SO}‐C_6_H_5_)], which lies only 10.8 kcal/mol above **5**.

## Graphical abstract

**A nickel/*N*‐heterocyclic carbene** (NHC) catalytic system has been developed for the borylation of aryl sulfoxides with B_2_(neop)_2_ (neop=neopentyl glycolato). A wide range of aryl sulfoxides with different electronic and steric properties were converted into the corresponding arylboronic esters in good yields. Elucidation of key mechanistic features of this newly developed reaction led to the identification of fully characterized nickel intermediates.

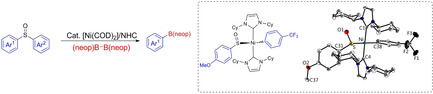

## Introduction

The expansion of the range of alternative electrophilic coupling partners is an important and valuable topic in research on transition metal‐catalyzed cross‐coupling reactions.^[1]^ Recently, cross‐coupling of organosulfur compounds as electrophiles has gained much attention.[^2^, ^3^] Due to their ubiquity and versatility in synthetic organic chemistry, organosulfur compounds are expected to be useful surrogates for aryl halides in transition‐metal‐catalyzed coupling reactions. In contrast, cross‐coupling of aryl sulfoxides has rarely been explored.^[4]^ Sulfoxides are prevalent in nature and can be found in bioactive products and pharmaceuticals, and they are also useful as synthetic intermediates.[^5^, ^6^] Due to the electron deficiency of the sulfur atoms in the sulfoxide groups, the C−S bonds are considered to be easily cleavable and more reactive than aryl sulfides.

**Scheme 1:**
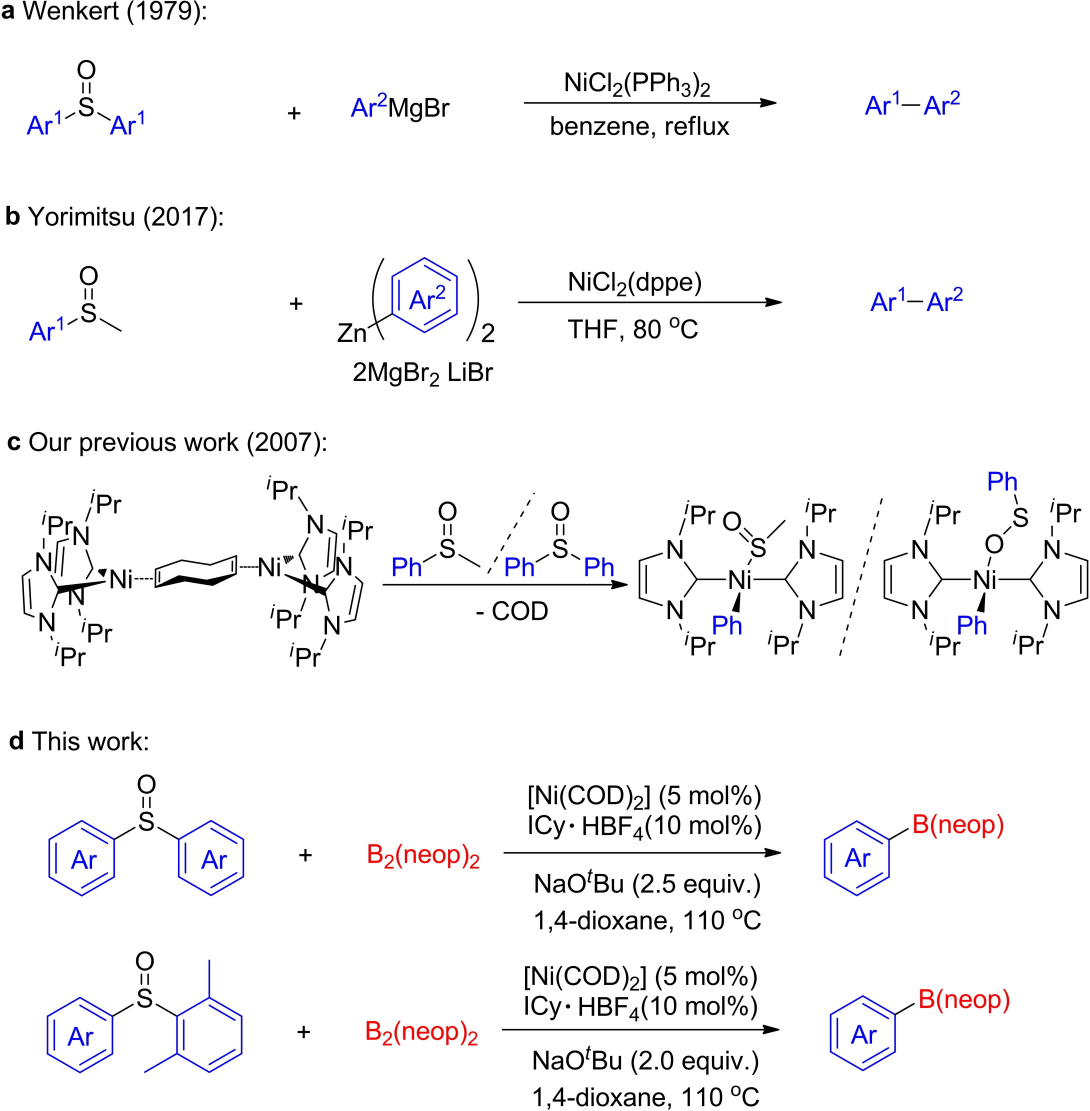
Nickel‐mediated C−S activation of sulfoxides.

Previous work described C−S bond cleavage of sulfoxides by nickel‐catalyzed reactions using Grignard reagents.^[7]^ The groups of Wenkert and Enthaler developed Kumada‐type cross‐coupling reactions of aryl sulfoxides with aryl magnesium reagents and nickel catalysts (Scheme 1a).^[8]^ Recently, the Yorimitsu group reported the NiCl_2_(dppe)‐catalyzed Negishi‐type cross‐coupling of aryl methyl sulfoxides with aryl zinc reagents (Scheme 1b).^[9]^ However, a large amount of homocoupling byproducts of the aryl zinc reagents is formed along with the desired heterocoupling products. In 2007, one of our groups investigated the reactivity of the NHC‐stabilized nickel(0) complex [Ni_2_(I^*i*^Pr)_4_{*μ*‐(*η* ^2^:*η* ^2^)‐COD}] (I^*i*^Pr=1,3‐di‐*iso*‐propylimidazolin‐2‐ylidene; COD=1,5‐cyclooctadiene) with sulfoxides in stoichiometric bond‐activation reactions. We first demonstrated the transition metal‐mediated C−S cleavage of sulfoxides containing sp^2^‐ and sp^3^‐hybridized carbon bonds attached to the sulfur atom, leading to a [Ni−S(=O)R] structure, and reported the first structurally characterized complex *trans*‐[Ni(I^*i*^Pr)_2_(Ph)(OSPh)] featuring an oxygen‐bound sulfinyl ligand (Scheme 1c).^[10]^ We then extended our work to C−S bond activation in thioethers, benzothiophene and dibenzothiophene using [Ni_2_(I^*i*^Pr)_4_{*μ*‐(*η* ^2^:*η* ^2^)‐COD}].^[11]^

Aryl‐ and heteroboronate esters are extremely important because of their exceptional utility as synthetic building blocks,^[12]^ especially in C−C, C−O, C−N and C−X bond‐forming reactions, as exemplified by the Suzuki−Miyaura coupling reaction.^[13]^ To date, numerous methodologies have been developed for the synthesis of arylboronate esters.[^14^, ^15^] In recent years, the development of transition‐metal‐catalyzed Miyaura‐type borylation reactions has allowed the synthesis of arylboronate esters under mild reaction conditions.^[15]^

As first row “Earth‐abundant” metal catalysts have become of increasing interest for chemists due to their low cost and toxicity, recent research has enabled nickel‐catalyzed borylation of less reactive C−Cl,^[16]^ C−F,^[17]^ C−O,^[18]^ C−N,^[19]^ and C−C bonds.^[20]^ Our groups have reported the reactivity of the nickel(0)‐NHC complex [Ni_2_(I^*i*^Pr)_4_{*μ*‐(*η* ^2^:*η* ^2^)‐COD}] towards organic halides and other substrates in stoichiometric bond‐activations.^[21]^ Recently, we demonstrated efficient thermal^[22a]^ as well as photocatalytic^[22b]^ procedures for the borylation of C−F bonds of aryl fluorides in the presence of [Ni(IMes)_2_] (IMes=1,3‐dimesitylimidazolin‐2‐ylidene) and a Ni/Rh tandem catalyst system, respectively. Subsequently, by applying a readily prepared NHC‐stabilized nickel(0) catalyst precursor [Ni_2_(ICy)_4_{*μ*‐(*η* ^2^:*η* ^2^)‐COD}] (ICy=1,3‐dicyclohexylimidazolin‐2‐ylidene) and the base NaOAc, we demonstrated the catalytic C−Cl borylation of aryl chlorides.^[23]^ Very recently, we reported an efficient [Ni(IMes)_2_]‐catalyzed directed C3‐selective C−H borylation of indoles.^[24]^ In 2006, Hosoya^[25a]^ and Yorimitsu^[25b]^ independently demonstrated the borylation of aryl sulfides employing rhodium and palladium‐NHC catalysts, respectively. Yorimitsu's group continued to develop the borylation of diaryl sulfoxides using a phosphine‐ligated palladium catalyst and LiN(SiMe_3_)_2_ as the base.^[26]^ Very recently, Pd‐catalyzed as well as photoinduced strategies for the borylation of C−S bonds in aryl sulfonium salts were reported by Yorimitsu^[27a]^ and Gao,^[27b]^ respectively.

With the first demonstration of a Ni‐mediated C−S bond cleavage of sulfoxides by our group,[^10^, ^11^] and our successful previous work on the NHC nickel‐catalyzed borylation of aryl halides,[^22^, ^23^] we envisioned transforming aryl sulfoxides to arylboronate esters using an NHC nickel catalyst. Inexpensive Ni and ligand were used in place of expensive Rh and Pd metals which were employed previously. We note that the prices of those 2 metals have increased drastically over the past year. Furthermore, compared with conventional groups, such as aryl (pseudo)halides or alcohols, sulfoxides represent an alternative and complimentary substitute in coupling reactions, as aryl sulfoxides can be prepared directly from arenes by reaction with thionyl chloride. Herein, we report initial results on the NHC Ni‐catalyzed borylation of aryl sulfoxides.

## Results and Discussion

Our initial studies involved evaluation of the borylation of diphenyl sulfoxide **1 a** with B_2_(neop)_2_ ^[28]^ (Table 1), noting that B_2_pin_2_ proved unreactive under our conditions (see Table S1 in the Supporting Information). In the presence of 5 mol % of [Ni(COD)_2_], 10 mol % of IMes, and 2.5 equivalents of KO^*t*^Bu, at 110 °C under an argon atmosphere in toluene solvent, the desired borylated product **3 a** was obtained in 26 % yield (entry 1 in Table 1). With this promising first result, we screened a range of ligands, bases, solvents, catalysts and boron sources to determine the scope and limitations of this reaction (Table 1). The reaction was accompanied by the reduction of sulfoxides to sulfides.^[29]^ Among the various NHC ligands we examined (entries 1–4 in Table 1), a dramatic effect of ICy ⋅ HBF_4_ is notable, affording a 61 % yield of **3 a**. Employing the free carbene ICy instead of ICy ⋅ HBF_4_ and a base did not affect the reactivity within experimental error (entry 2 in Table 1). However, the reaction was sensitive to the nature of the alkoxide under these conditions, as KOMe was not as effective as KO^*t*^Bu (entry 5 in Table 1), and no reaction took place with NaOMe or LiO^*t*^Bu (entries 6 and 7 in Table 1). The replacement of KO^*t*^Bu with NaO^*t*^Bu resulted in an increased yield of 73 % (entry 8 in Table 1). Solvent screening showed 1,4‐dioxane to be the most effective among those examined (entry 9 in Table 1). Polar solvents, such as THF, MTBE (methyl *tert*‐butyl ether), CH_3_CN and DMF, were not suitable for this borylation reaction (see Table S3 in the Supporting Information).

**Table 1:** Screening of reaction conditions for the Ni‐catalyzed borylation of diphenyl sulfoxide **1 a**.^[a]^

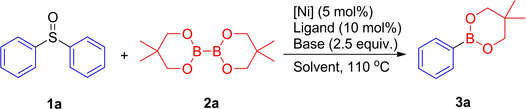					
Entry	Base	Catalyst	Ligand	Solvent	Yield of **3 a** [%]^[b]^
1	KO^*t*^Bu	[Ni(COD)_2_]	IMes	toluene	26
2	KO^*t*^Bu	[Ni(COD)_2_]	ICy	toluene	58
3	KO^*t*^Bu	[Ni(COD)_2_]	ICy ⋅ HBF_4_	toluene	61
4	KO^*t*^Bu	[Ni(COD)_2_]	IDipp ⋅ HBF_4_	toluene	37
5	KOMe	[Ni(COD)_2_]	ICy ⋅ HBF_4_	toluene	22
6	NaOMe	[Ni(COD)_2_]	ICy ⋅ HBF_4_	toluene	0
7	LiO^*t*^Bu	[Ni(COD)_2_]	ICy ⋅ HBF_4_	toluene	0
8	NaO^*t*^Bu	[Ni(COD)_2_]	ICy ⋅ HBF_4_	toluene	73
9	NaO^*t*^Bu	[Ni(COD)_2_]	ICy ⋅ HBF_4_	1,4‐dioxane	93 (81)^[c]^
10	NaO^*t*^Bu	[Ni_2_(I^*i*^Pr)_4_{*μ*‐(*η* ^2^:*η* ^2^)‐COD}]	–	1,4‐dioxane	79
11	NaO^*t*^Bu	[Ni_2_(ICy)_4_{*μ*‐(*η* ^2^:*η* ^2^)‐COD}]	–	1,4‐dioxane	88
12	NaO^*t*^Bu	[Ni(IMes)_2_]	–	1,4‐dioxane	26
13	NaO^*t*^Bu	[NiCl_2_]	ICy ⋅ HBF_4_	1,4‐dioxane	63
14	NaO^*t*^Bu	[Ni(OAc)_2_]	ICy ⋅ HBF_4_	1,4‐dioxane	66
15	NaO^*t*^Bu	[Ni(acac)_2_]	ICy ⋅ HBF_4_	1,4‐dioxane	62
16^[d]^	NaO^*t*^Bu	[Ni(COD)_2_]	ICy ⋅ HBF_4_	1,4‐dioxane	70
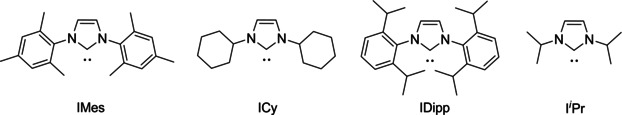					

[a] Reaction conditions, unless otherwise stated: diphenyl sulfoxide **1 a** (0.5 mmol, 1.0 equiv.), [Ni]‐catalyst precursor (5 mol %), ligand (10 mol %), B_2_(neop)_2_ (2.5 equiv.), base (2.5 equiv.), solvent (3 mL), 110 °C, 20 h. [b] The yields were determined by GC‐MS of a diluted and filtered aliquot of the reaction mixture using dodecane as the internal standard (average of two runs). [c] Isolated yield. [d] [Ni(COD)_2_] (1 mol %), ICy ⋅ HBF_4_ (2 mol %).

We then investigated the catalytic activity of different NHC nickel complexes for the borylation using NaO^*t*^Bu as a base and 1,4‐dioxane as the solvent at 110 °C for 20 h. The complex [Ni_2_(I^*i*^Pr)_4_{*μ*‐(*η* ^2^ : *η* ^2^)‐COD}]^23^ also afforded the borylated compound **3 a** in a very good yield of 79 % (entry 10 in Table 1). The reaction of [Ni(COD)_2_] with two equivalents of the free carbene ICy in THF, which affords the dinuclear, COD‐bridged complex [Ni_2_(ICy)_4_{*μ*‐(*η* ^2^ : *η* ^2^)‐COD}], led to formation of the borylation product **3 a** in a similar high yield of 88 % (entry 11 in Table 1). The application of [Ni(IMes)_2_], however, containing the sterically more demanding NHC IMes, turned out to be less efficient, showing only moderate catalytic activity (26 % yield) for the borylation of sulfoxides under the current conditions (entry 12 in Table 1). Other commercially available nickel sources such as [NiCl_2_], [Ni(acac)_2_] and [Ni(OAc)_2_] as catalyst precursors also proved successful in this coupling reaction (entries 13–15 in Table 1). Notably, decreasing the [Ni(COD)_2_] loading to 1 mol % still resulted in 70 % yield (entry 16 in Table 1), and no product was observed in the absence of Ni catalyst.

**Figure 1:**
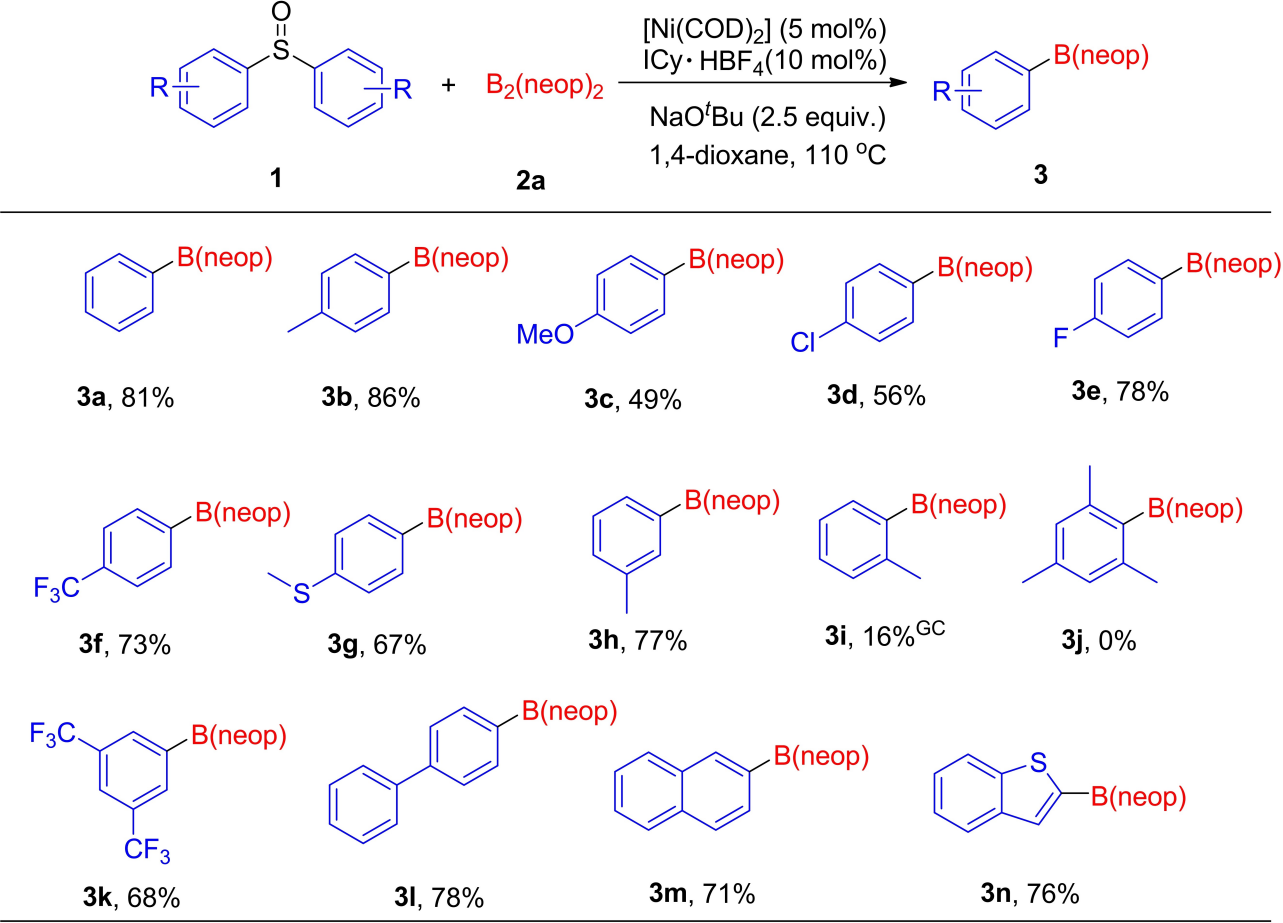
Screening of diaryl sulfoxides for the Ni‐catalyzed borylation reaction.^[a]^ [a] Reaction conditions, unless otherwise stated: diaryl sulfoxides **1** (0.5 mmol, 1.0 equiv.), [Ni(COD)_2_] (5 mol %), ICy ⋅ HBF_4_ (10 mol %), B_2_(neop)_2_ (2.5 equiv.), NaO^*t*^Bu (2.5 equiv.), 1,4‐dioxane (3 mL), 110 °C, 20 h. Isolated yield after chromatographic workup. The yield with a “GC” superscript is the GC‐MS yield with dodecane as the internal standard.

Having identified the optimized conditions with diphenyl sulfoxide **1 a** as the standard substrate, we then conducted the borylation of a series of diaryl sulfoxides **1 b**–**1 n** (Figure 1). Diaryl sulfoxides bearing electron‐donating substituents (**1 b**,**c**,**g**,**h**,**i**) gave the borylated products in yields up to 86 %. Electron‐rich substrates containing a *p*‐OMe or *p*‐SMe group (**1 c** and **1 g**) were converted into their corresponding arylboronic esters in 49 % and 67 % yield, respectively. Unfortunately, the attempted borylation of *ortho*‐tolyl sulfoxide (2‐CH_3_−C_6_H_4_)_2_S=O **1 i** gave the product **3 i** in only 16 % GC yield, whereas bis(1,3,5‐trimethylphenyl) sulfoxide **1 j** (disubstituted at both *ortho* positions) failed to give the product **3 j**, and only unreacted starting material was identified by GC‐MS. Electron‐poor diaryl sulfoxides (**1 d**–**1 f**, **1 k**) were also borylated in satisfying to good yields. Thus, chloro‐ or fluoro‐ moieties on **1 d** or **1 e**, respectively, were compatible with the borylation reaction, undergoing selective cleavage of the C−S(=O) bond over that of the C−F or C−Cl bond. Applying *para*‐substituted **3 f**, and the sulfoxide **3 k**, substituted in both *meta*‐positions with −CF_3_ groups, gave the products in 73 % and 68 % yields, respectively. In an analogous fashion, biphenyl sulfoxide **1 l** reacted well under the standard conditions. The π‐extended dinaphthyl sulfoxide **1 m** also underwent the reaction to furnish **3 m** in 71 % yield. Interestingly, a substrate containing benzothiophene moieties was also tolerated, demonstrated by the synthesis of borylated compound **3 n** in 76 % yield.

**Figure 2:**
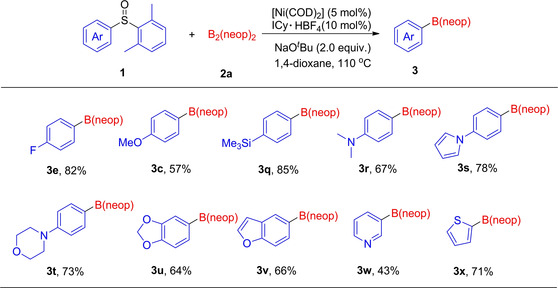
Regioselective borylation of unsymmetrical diaryl sulfoxides.^[a]^ [a] Reaction conditions, unless otherwise stated: diaryl sulfoxides **1** (0.5 mmol, 1.0 equiv.), [Ni(COD)_2_] (5 mol %), ICy ⋅ HBF_4_ (10 mol %), B_2_(neop)_2_ (2.0 equiv.), NaO^*t*^Bu (2.0 equiv.), 1,4‐dioxane (3 mL), 110 °C, 20 h. Isolated yield after chromatographic workup.

Considering the failure when utilizing **1 j** (disubstituted at both *ortho* positions) as the substrate, we anticipated that regioselective borylation of unsymmetrical diaryl sulfoxides would be feasible by means of steric bias (Figure 2). The borylation of sterically biased fluoro‐substituted **1 o** and methoxy‐substituted **1 p** with **2 a** proceeded smoothly to afford **3 e** and **3 c** in 82 % and 57 % yields, respectively. Using a substrate containing a trimethylsilyl moiety, under the conditions employed, generated borylated **3 q** in 85 % yield. Furthermore, a wide variety of π‐extended systems participated in the reaction to afford the borylated products **3 r**–**3 v** in good yields, and the presence of substituted nitrogen‐ and oxygen‐containing heterocycles did not interfere with productive C−B bond formation. This nickel‐catalyzed method was also applicable to the borylation of 3‐((2,6‐dimethylphenyl)sulfinyl)pyridine **1 w** and 2‐((2,6‐dimethylphenyl)sulfinyl)thiophene **1 x** derivatives. Although the reaction shows a broad scope, there are some functional groups which were not tolerated (as shown in Supporting Information). Cyano‐, amino‐, ester‐ and indole‐substituted substrates failed to provide borylated pro‐ ducts, and the starting materials were recovered. As expected, 2‐((2,6‐difluorophenyl)sulfinyl)‐1,3‐dimethylbenzene (substituted at four *ortho* positions) was also unsuccessful. Furthermore, an aryl alkyl sulfoxide also proved to be ineffective in this Ni‐NHC system.[^9^, ^30^]

**Scheme 2:**
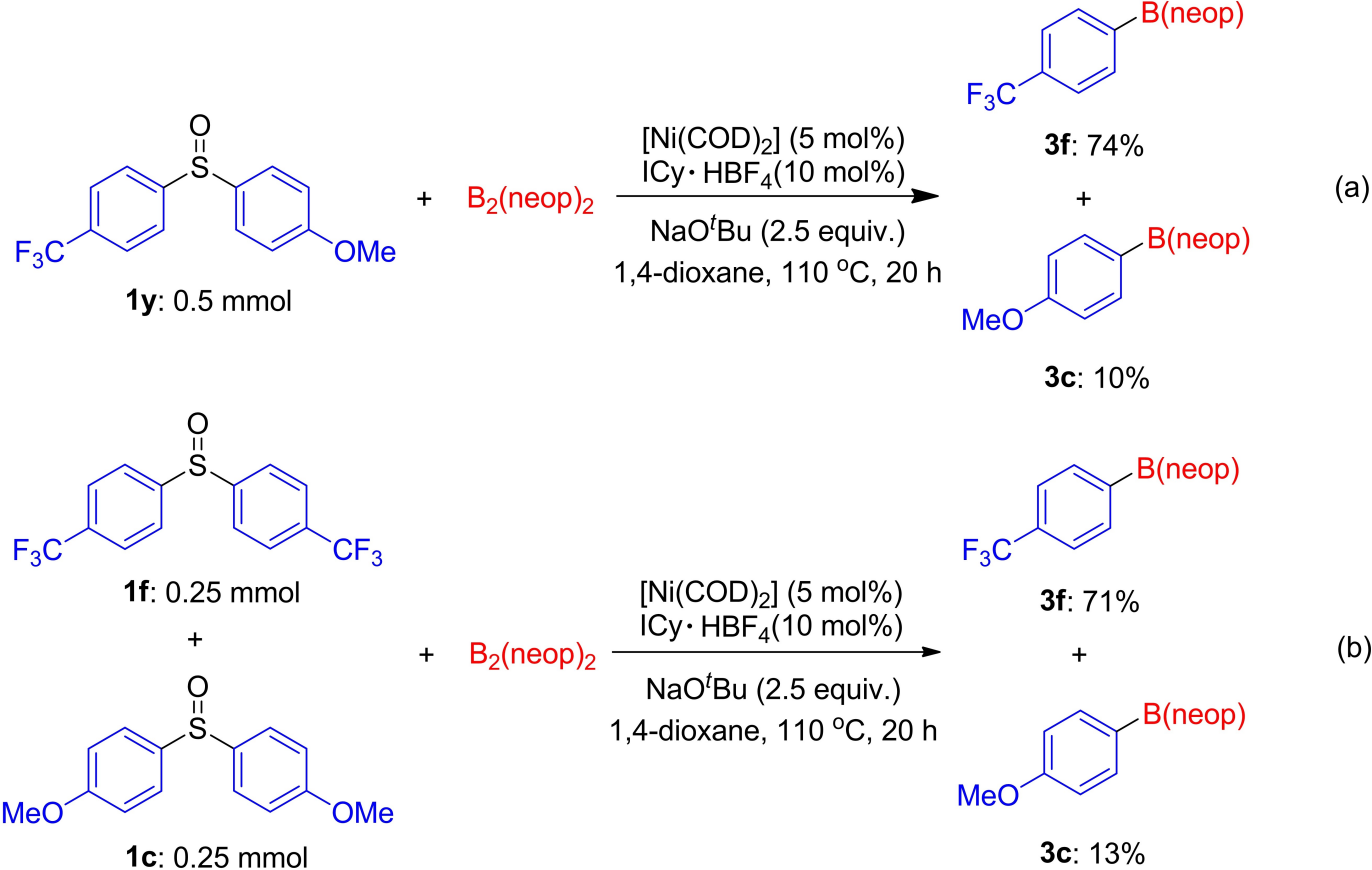
Competition experiments. (a) Intramolecular competitive borylation of unsymmetrical diaryl sulfoxide **1 y**. (b) Intermolecular competitive borylation of **1 c** and **1 f** in the same vessel.

**Scheme 3:**
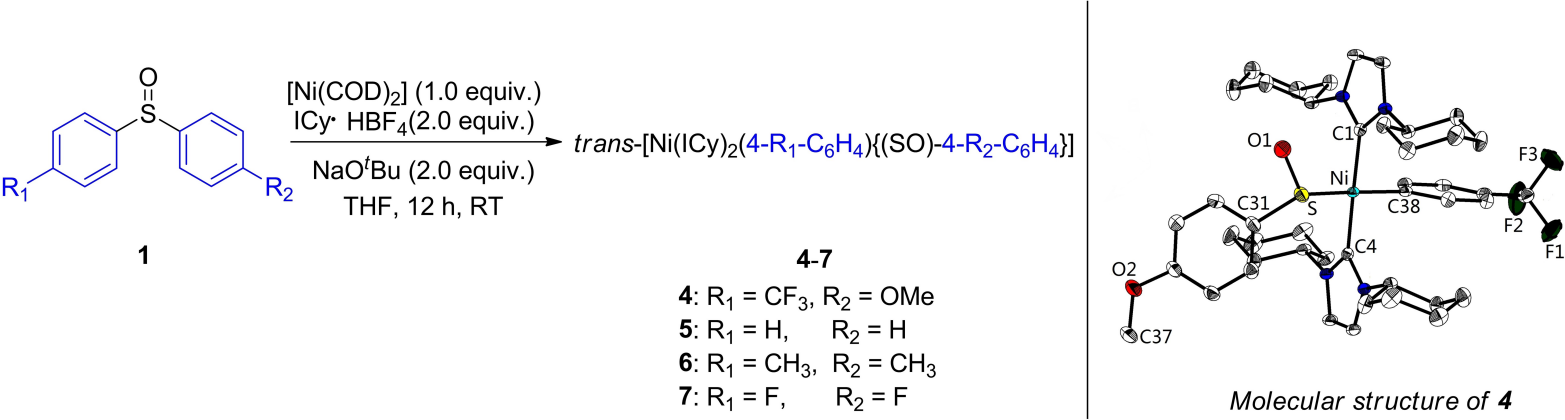
Synthesis of the oxidative‐addition products of the type *trans*‐[Ni(ICy)_2_(Ar^1^){(SO)Ar^2^}] **4**–**7**. Molecular structure of **4** shown with thermal ellipsoids drawn at the 50 % probability level; hydrogen atoms are omitted for clarity. Selected bond lengths [Å] and angles [°] of **4**: Ni−C1 1.894(2), Ni−C4 1.907(2), Ni−C38 1.925(2), Ni−S 2.2259(8), S−C31 1.801(2), S=O1 1.535(2); C1−Ni−C4 176.16(7), C1−Ni−C38 88.31(7), C4−Ni−C38 88.59(7), C1−Ni−S 91.09(6), C4−Ni−S 92.37(6), C38−Ni−S 169.45(5).

Next, we conducted competition experiments to gain additional insight into the effect of the electronic properties of aryl sulfoxides on the reaction (Scheme 2). First, the intramolecular competition reaction of electronically biased unsymmetrical diaryl sulfoxide **1 y** was conducted with B_2_(neop)_2_ (Scheme 2a). The electron‐deficient aryl moiety reacted preferentially to afford **3 f** in 74 % yield accompanied by 10 % of **3 c** as a minor product. A similar trend was found for the intermolecular version. Thus, in the same vessel, 0.25 mmol of electron‐deficient and ‐rich aryl sulfoxides **1 f** and **1 c** were treated with 2.5 equivalents of B_2_(neop)_2_, and the reaction predominantly consumed electron‐deficient **1 f** providing **3 f** in 71 % yield (Scheme 2b). To examine the origin of the selectivity in the Ni‐catalyzed borylation of the electronically biased unsymmetrical diaryl sulfoxide, we reacted methoxyphenyl‐4‐(trifluoromethyl)phenyl sulfoxide (**1 y**) with a Ni(0)‐NHC complex, and the oxidative addition product *trans*‐[Ni(ICy)_2_(4‐CF_3_‐C_6_H_4_){(SO)‐4‐MeO‐C_6_H_4_}] **4** was isolated in 86 % yield (Scheme 3, left). Single‐crystal X‐ray diffraction analysis of **4** showed that the C−S oxidative addition to Ni proceeded mainly at the side of electron‐poor (trifluoromethyl)phenyl group (Scheme 3, right).

We therefore investigated further the first step of a possible catalytic cycle, namely the oxidative addition of the C−(S=O) bond. According to our previous work, we prepared *trans*‐[Ni(ICy)_2_(C_6_H_5_){(SO)‐C_6_H_5_}] **5** by treatment of diphenyl sulfoxide **1 a** with [Ni(COD)_2_], ICy ⋅ HBF_4_ and NaO^*t*^Bu in THF at room temperature, which was isolated as a yellow solid in 78 % yield (Scheme 3). In analogous reactions utilizing bis(4‐methylphenyl) sulfoxide **1 b** and bis(4‐fluorophenyl) sulfoxide **1 e**, we isolated the oxidative addition products *trans*‐[Ni(ICy)_2_(4‐CH_3_‐C_6_H_4_){(SO)‐4‐CH_3_‐C_6_H_4_}] **6** and *trans*‐[Ni(ICy)_2_(4‐F‐C_6_H_4_){(SO)‐4‐F‐C_6_H_4_}] **7** in 73 % and 63 % yields, respectively. Complexes **4**–**7** were characterized by ^1^H, ^13^C and ^19^F NMR spectroscopy, elemental analysis, HRMS, IR spectroscopy and, for **4** and **5**, by single‐crystal X‐ray diffraction.^[31]^ In all cases, the ensuing Ni(II)‐(Ar){(SO)‐Ar’} complexes were found to be stable both in the solid state and in solution. Remarkably, in contrast with previously reported phosphine ligated Ni(II) aryl complexes, which rapidly decompose to nickel(I) species, complexes **4**–**7** are stable in solution even upon heating at 110 °C for several hours.^[32]^

**Figure 3:**
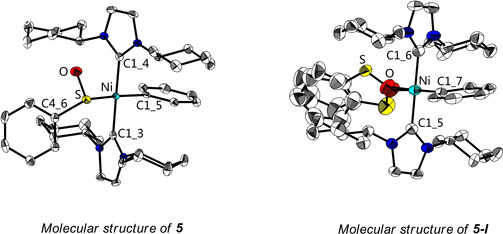
Molecular structures of **5** and **5**‐**I** shown with thermal ellipsoids drawn at the 50 % probability level; hydrogen atoms are omitted for clarity. Selected bond lengths [Å] and angles [°] of **5**: Ni−C1_4 1.894(2), Ni−C1_3 1.900(2), Ni−C1_5 1.929(2), Ni−S 2.2284(8), S−C4_6 1.798(2), S=O 1.537(2); C1_4‐Ni−C1_3 177.73(9), C1_4‐Ni−C1_5 89.93(9), C1_3‐Ni−C1_5 88.08(9), C1_5‐Ni−S 170.33(7), C1_4‐Ni−S 90.87(7), C1_3‐Ni−S 91.27(7). Selected bond lengths [Å] and angles [°] of **5**‐**I**: Ni−C1_5 1.895(4), Ni−C1_6 1.901(4), Ni−C1_7 1.930(4), Ni−O 1.924(7) / 1.91(2), S−O 1.606(8) / 1.611(13); C1_5‐Ni−C1_6 178.66(18), C1_5‐Ni−C1_7 90.02(17), C1_6‐Ni−C1_7 88.79(18), C1_7‐Ni−O 173.5(2) / 175.8(7), C1_5‐Ni−O 87.3(5) / 91.6(16), C1_6‐Ni−O 94.0(5)/89.5(16).

Surprisingly, we found a small number of single crystals of another compound which had also grown from a solution of **5**, namely *trans*‐[Ni(ICy)_2_(C_6_H_5_)(OSC_6_H_5_)] **5**‐**I**, a product in which the phenyl sulfinyl ligand is bound *via* oxygen to the nickel atom, which was confirmed by X‐ray diffraction (Figure 3).^[31]^ In both structures, the coordination around the Ni atom is nearly perfectly square‐planar with the sum of the angles around Ni being 360° in each case (within 3 esd's, see Table S6 in the Supporting Information). In addition, the *trans*‐influence of the O− and S‐bound ligands appears to be the same, as the Ni−C(phenyl) bond lengths of 1.929(2) and1.930(4) Å for **5** and **5**‐**I**, respectively, are identical within less than 1 esd (see Table S6 in the Supporting Information).

**Figure 4:**
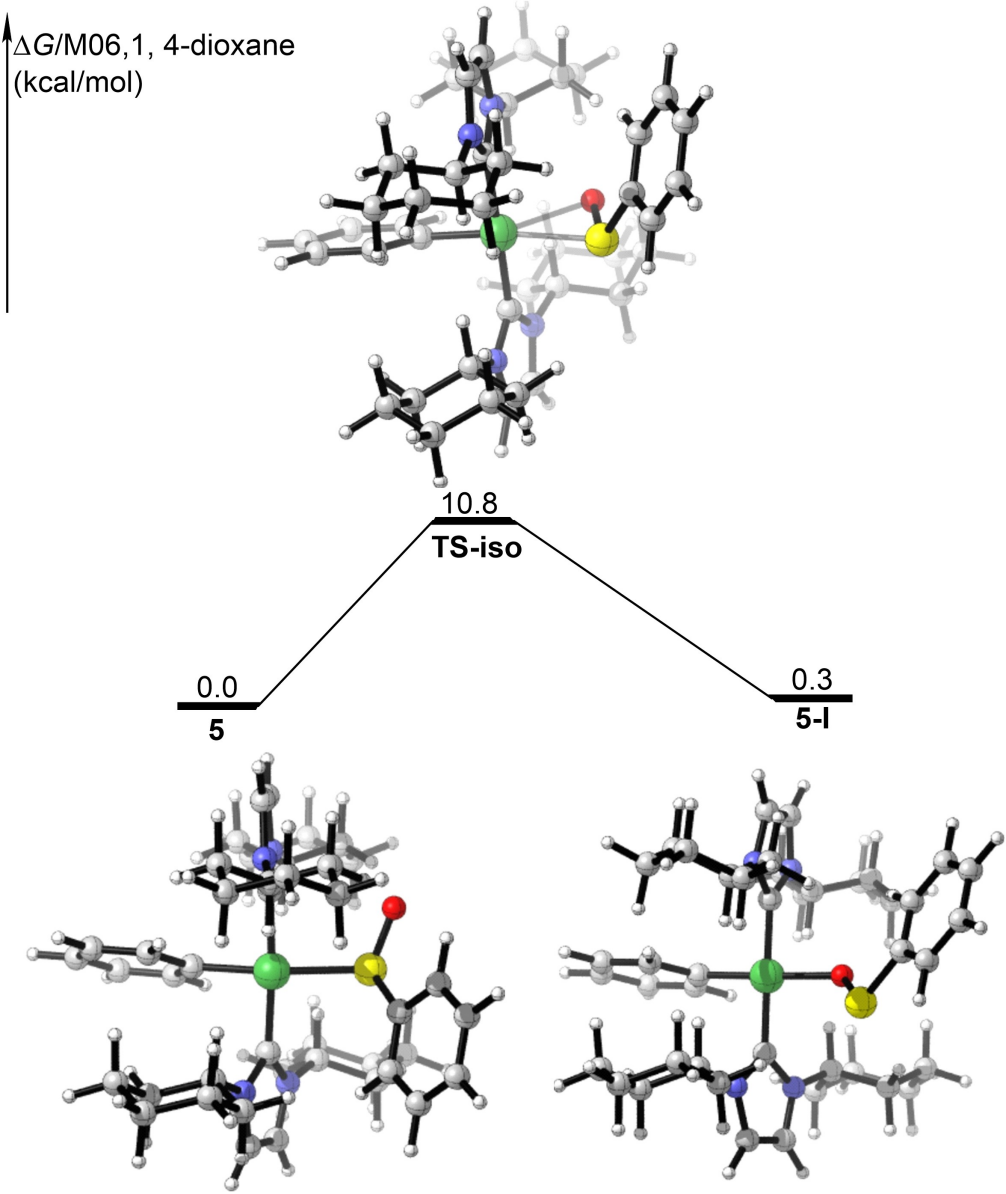
Free energy profile for the isomerization of *trans*‐[Ni(ICy)_2_(C_6_H_5_)(SOC_6_H_5_)] **5** to *trans*‐[Ni(ICy)_2_(C_6_H_5_)(OSC_6_H_5_)] **5**‐**I** calculated at the (M06/def2‐TZVP, SMD//B3‐LYP/def2‐SVP) level of theory. Energies relative to **5** is given in kcal/mol.

We performed DFT calculations to address the relative energies of both isomers and to investigate a possible transition state connecting them (Figure 4). The calculated results show that the relative energies of both isomers are similar. Both isomers are connected *via* a transition state (**TS**‐**iso**) with the *trans*‐[Ni(ICy)_2_(C_6_H_5_)(η^2^‐{SO}‐C_6_H_5_)] structure, in which the sulfoxide ligand is η^2^‐bound to the nickel center, which lies only 10.8 kcal/mol above **5**. As the barrier for the interconversion is low, we expect that both Ni complexes can easily interconvert and are thus in equilibrium, which is consistent with the low temperature NMR spectra of **5** (see Figures S11 and S12 in the Supporting Information), in which both isomers were observed at −90 °C.

**Scheme 4:**
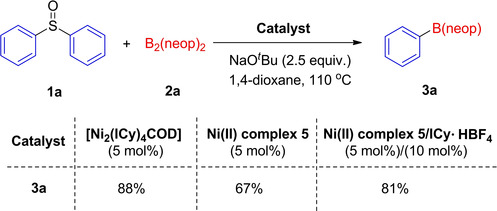
Catalytic activity of Ni(0) and Ni(II) species.

We next evaluated the catalytic behavior of different Ni(0) and Ni(II) species for the borylation of diphenyl sulfoxide (Scheme 4). The isolated COD‐bridged Ni(0) complex [Ni_2_(ICy)_4_{*μ*‐(*η* ^2^:*η* ^2^)‐COD}] showed high reactivity for the borylation, similar to that of a mixture of [Ni(COD)_2_] with ICy. Ni(II) complex **5** was also capable of facilitating the catalytic borylation with approximately identical efficiency to that of a combination of [Ni(COD)_2_] and ICy. To evaluate a possible resting state of the catalytic cycle, and thus gather information about the turnover‐limiting step, we performed an *in situ* NMR study. The reaction of [Ni(COD)_2_] and ICy with an aryl sulfoxide leads to rapid oxidative addition of the C−S bond with formation of *trans*‐[Ni(ICy)_2_(Ar^1^)(SOAr^2^)] as a mixture of S‐ and O‐bound isomers within one hour at room temperature. The oxidative addition product *trans*‐[Ni(ICy)_2_(Ar^1^)(SOAr^2^)] is the resting state of the catalytic cycle (see Figures S6‐S9 in the Supporting Information). The reaction of [Ni(COD)_2_], ICy with stoichiometric amounts of B_2_(neop)_2_ did not show any evidence for the formation of a likely nickel‐boryl complex intermediate (see Figure S10 in the Supporting Information). Heating a mixture of [Ni(ICy)_2_(Ar)(SOR)}] and B_2_(neop)_2_ to 110 °C for 1 h revealed the formation of a small amount of ArB(neop) formed by ^19^F and ^11^B NMR spectroscopy (see Figures S4–S5 in the Supporting Information), but no Ni‐boryl intermediate was observed. It was not possible to isolate or observe *trans*‐[Ni(ICy)_2_(Bneop)(Ar)], as Ni‐boryls are fairly unstable, and reductive elimination of the product is fast, as expected.^[33]^

**Scheme 5:**
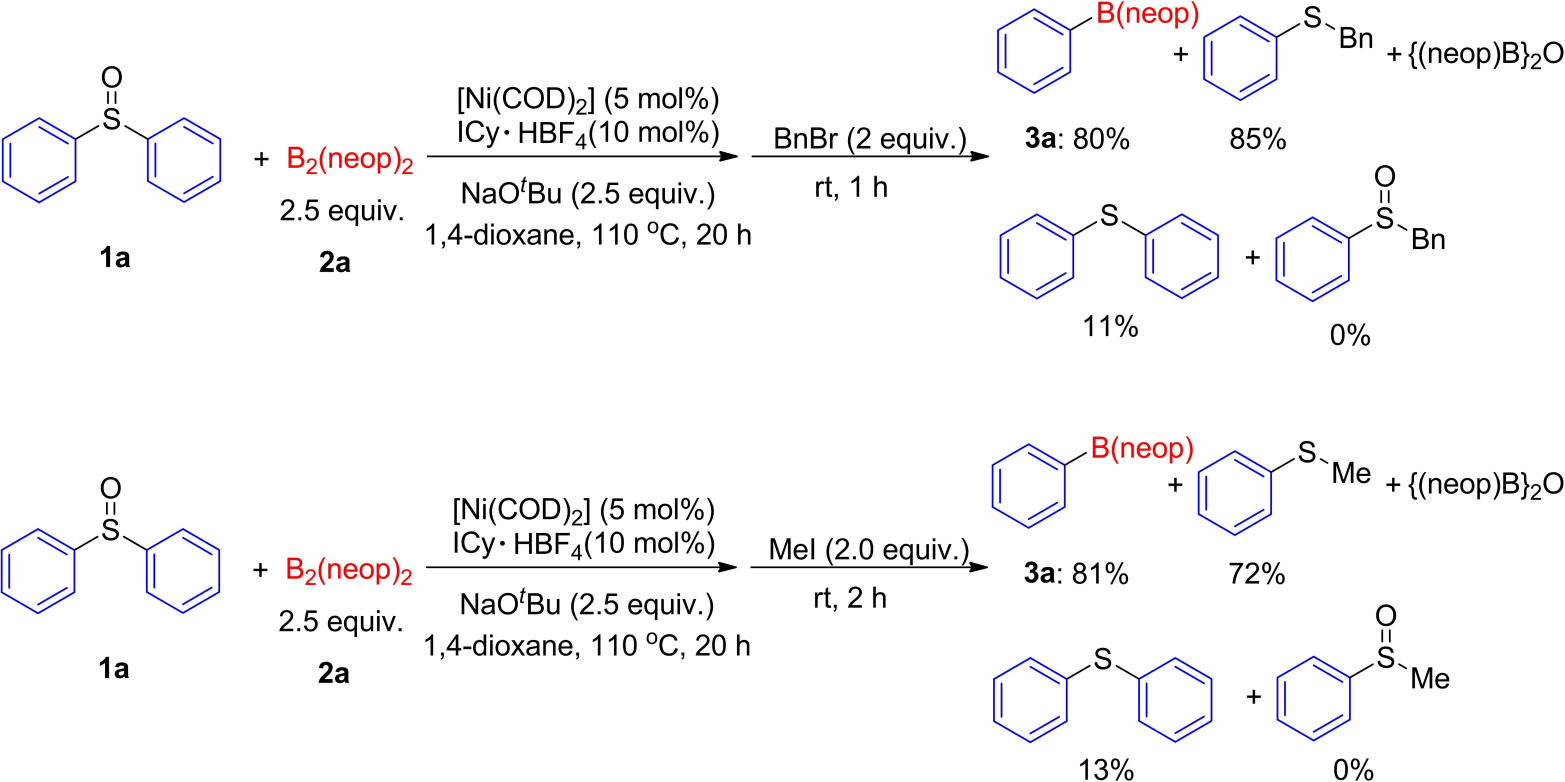
Electrophilic trapping of the anionic sulfur fragments.

To probe the fate of the leaving groups, we conducted experiments to trap the sulfur‐containing products (Scheme 5). The borylation of **1 a** with B_2_(neop)_2_ was carried out under standard conditions, and then the reaction mixture was treated with 2.0 equiv. of benzyl bromide or iodomethane, respectively. Interestingly, in the first case, benzyl phenyl sulfide was obtained in 85 % yield accompanied by the desired borylated product **3 a**, and benzyl phenyl sulfoxide was not observed. Likewise, in the second case, methyl phenyl sulfide was formed in 72 % yield, along with **3 a**. As sulfides rather than sufloxides were observed as byproducts, reduction of the latter by the diboron was accompanied by the formation of {(neop)B}_2_O (see Figure S3 in the Supporting Information).^[29]^ In order to explore whether the mechanism is a one‐step nickel‐catalyzed borylation of the sulfoxide or involves reduction of the sulfoxide to a sulfide with B_2_(neop)_2_ followed by nickel‐catalyzed borylation of the sulfide, we reacted diphenylsulfide as a potential substrate under the standard conditions. However, diphenylsulfide failed to give the borylated product and 91 % of the diphenylsulfide was recovered. When a mixture of diphenyl sulfoxide **1 a** and di‐*p*‐tolylsulfide were reacted under the standard conditions, PhB(neop) **3 a** was still obtained in good (78 %) yield (see Section 1.7c in the Supporting Information). These results prove that a diarylsulfide is neither a catalyst poison nor inhibitor in this reaction and is also unlikely to be an intermediate.

**Scheme 6:**
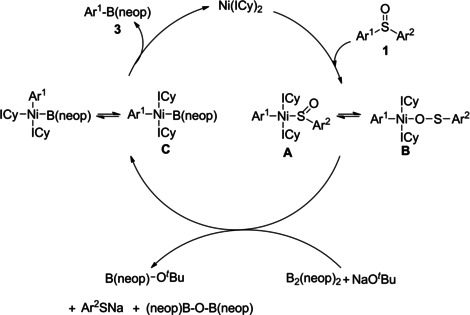
Proposed mechanism for the NHC‐nickel‐catalyzed borylation of aryl sulfoxides.

The isolation and characterization of the key intermediates and products of the C−S borylation of diaryl sulfoxides leads us to propose the following mechanism (Scheme 6). In the first step, the *in situ* formed [Ni(ICy)_2_] reacts with aryl sulfoxide **1** *via* oxidative addition of the C−S bond forming *trans*‐[Ni(ICy)_2_(Ar^1^)(SOAr^2^)] (**A**), which is in equilibrium with *trans*‐[Ni(ICy)_2_(Ar^1^)(OSAr^2^)] (**B**). This is followed by boryl transfer to generate *trans*‐[Ni(ICy)_2_{B(neop)}(Ar^1^)] (**C**) and B(neop)‐O^*t*^Bu^[34]^ assisted by the base, and subsequent rapid reductive elimination from a *cis* isomer delivers the target product **3** and regenerates the [Ni(ICy)_2_] species.

## Conclusion

We have developed an efficient nickel‐catalyzed borylation of diaryl sulfoxides *via* C−S bond activation producing a variety of useful organoboronates. This simple procedure has a wide substrate scope, including electron‐rich aryl sulfoxides, and broad functional group tolerance, using an inexpensive, and a relatively low toxicity first‐row transition metal. Elucidation of key mechanistic features of this newly developed reaction led to the identification of fully characterized nickel intermediates. The molecular structures of **5** and **5**‐**I** demonstrate the ambivalence of O *versus* S binding of sulfoxide ligands. The nickel sulfinyl moieties may be of significance to bioinorganic chemists regarding the deactivation of nickel‐containing enzymes.^[35]^ Further mechanistic studies of this borylation process, as well as expansion of the scope of this transformation, are underway in our laboratory.

## Conflict of interest

The authors declare no conflict of interest.

## Supporting information

As a service to our authors and readers, this journal provides supporting information supplied by the authors. Such materials are peer reviewed and may be re‐organized for online delivery, but are not copy‐edited or typeset. Technical support issues arising from supporting information (other than missing files) should be addressed to the authors.

Supplementary
