# Supplementary material for: Ni‐Catalyzed Borylation of Aryl Sulfoxides
Source: Chemistry. 2021 May 6;27(31):8149–58. doi: 10.1002/chem.202100342 (PMC8252015; doi:10.1002/chem.202100342)
Supplement: Supplementary file 1 — Supplementary [file CHEM-27-8149-s001.pdf]

Chemistry–A European Journal

Supporting Information

Ni-Catalyzed Borylation of Aryl Sulfoxides

Mingming Huang, Zhu Wu, Johannes Krebs, Alexandra Friedrich, Xiaoling Luo,*
Stephen A. Westcott, Udo Radius,* and Todd B. Marder*

Table of Contents

1 Experimental Section 1

1.1 General Considerations 1

1.2 Optimization of the Reaction Conditions 2

1.3 General Procedure for the Synthesis of Sulfoxides 6

1.4 Details of the Catalytic Borylation of Aryl Sulfoxides 16

1.5 Unsuccessful Substrates 24

1.6 Synthesis and Characterization of *trans*-[Ni(ICy)$_2$(Ar^1){(SO)Ar^2}] 25

1.7 Investigations Concerning the Reaction Mechanism 27

1.8 VT-NMR spectra of **5** in THF-d_8 39

2 Crystallographic Details 40

3 Computational Details 42

4 NMR Spectra 43

5 Cartesian Coordinates for All Optimized Geometries 105

6 References 115

1 Experimental Section

1.1 General Considerations

All reactions and subsequent manipulations were performed under an argon atmosphere using standard Schlenk techniques or in a glovebox (Innovative Technology Inc. and Braun Uni Lab). All reactions were carried out in oven-dried glassware. Reagent grade solvents (Fisher Scientific and J.T. Baker) were nitrogen saturated and were dried and deoxygenated using an Innovative Technology Inc. Pure-Solv 400 Solvent Purification System, and further deoxygenated using the freeze-pump-thaw method. C_6D_6 and $CDCl_3$ were purchased from Sigma-Aldrich. $[Ni(COD)_2]$,[1] $[Ni(IMes)_2]$,[2] $[Ni_2(ICy)_4\{\mu\text{-}(\eta^2{:}\eta^2)\text{-COD}\}]$,[3] $[Ni_2(I^iPr)_4\{\mu\text{-}(\eta^2{:}\eta^2)\text{-COD}\}]$,[5a-b] ICy•$HBF_4$,[4] ICy•HCl,[4] ICy,[4] IDipp•HBF_4,[5a-b] IMes,[5c] were prepared according to published procedures. The diboron reagents B_2pin_2 and $B_2(neop)_2$ were a generous gift from AllyChem Co. Ltd. All other reagents were purchased from Sigma-Aldrich or ABCR.

NMR spectra were recorded at 298 K using Bruker Avance 300 (^{1}H, 300 MHz; ^{13}C, 75 MHz, ^{11}B, 96 MHz), Bruker DPX-400 (^{1}H, 400 MHz; ^{13}C, 100 MHz, ^{11}B, 128 MHz; ^{19}F, 376 MHz), or Bruker Avance 500 (^{1}H, 500 MHz; ^{13}C, 125 MHz, ^{11}B, 160 MHz; ^{19}F, 470 MHz) spectrometers. ^{1}H NMR chemical shifts are reported relative to TMS and were referenced via residual proton resonances of the corresponding deuterated solvent ($CDCl_3$: 7.26 ppm; C_6D_6: 7.16 ppm) whereas ^{13}C{^{1}H} NMR spectra are reported relative to TMS using the natural-abundance carbon resonances ($CDCl_3$: 77.2 ppm; C_6D_6: 128.0 ppm). ^{11}B and ^{19}F NMR chemical shifts are reported relative to external BF_3•OEt_3 or $CFCl_3$, respectively. Coupling constants are given in Hertz. Elemental analyses were performed in the microanalytical laboratory of the Institute of Inorganic Chemistry, Universität Würzburg, using an Elementar vario micro cube instrument. Automated flash chromatography was performed using a Biotage® Isolera Four system, on silica gel (Biotage SNAP cartridge KP-Sil 10 g and KP-Sil 25 g). Commercially available, precoated TLC plates (Polygram® Sil G/UV254) were purchased from Machery-Nagel. The removal of solvent was performed on a rotary evaporator *in vacuo* at a maximum temperature of 30 °C. GC-MS analyses were performed using a Thermo Fisher Scientific Trace 1310 gas chromatograph (column: TG-SQC 5% phenyl methyl siloxane, 15 m, Ø 0.25 mm, film 0.25 μm; injector: 250 °C; oven: 40 °C (2 min), 40 °C to 280 °C; carrier gas: He (1.2 mL min^{-1}) or an Agilent 7890A gas chromatograph (column: HP-5MS 5% phenyl methyl siloxane, 30 m, Ø 0.25 mm, film 0.25 μm; injector: 250 °C; oven: 40 °C (2 min), 40 °C to 280 °C (20 °C min^{-1}); carrier gas: He (1.2 mL min^{-1}) equipped with an Agilent 5975C inert MSD with triple-axis detector operating in EI mode and an Agilent 7693A series auto sampler/injector. High-resolution mass spectra were obtained using a Thermo Scientific Exactive Plus spectrometer equipped with an Orbitrap Mass Analyzer. Measurements were accomplished using an ASAP/APCI source with a corona needle, and a carrier-gas (N_2) temperature of 250 °C.

1.2 Optimization of the Reaction Conditions

Table S1: Screening of catalysts/ligands for the borylation of **1a**.

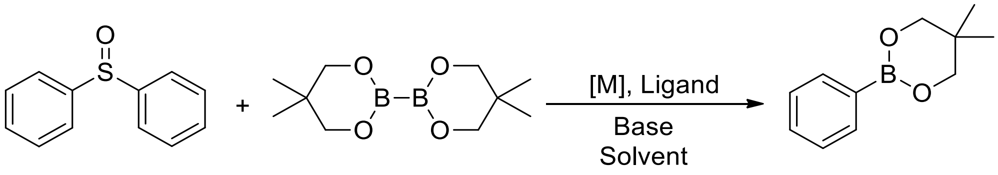

1a **2a** **3a**

Entry	Base	Catalyst	Ligand	Yield of **3a** (%)[a]
1	KO^{t}Bu	[Cu(ICy)Cl]	-	39
2	KO^{t}Bu	[Cu(IMes)Cl]	-	15
3	KO^{t}Bu	[Ni(COD)$_2$]	ICy•HBF$_4$	61
4	KO^{t}Bu	[Ni(COD)$_2$]	ICy•HCl	60
5	KO^{t}Bu	[Ni(COD)$_2$]	ICy	58
6	KO^{t}Bu	[Ni(COD)$_2$]	IDipp•HBF$_4$	37
7	KO^{t}Bu	[Ni(COD)$_2$]	IMes	26
8	KO^{t}Bu	[Ni(COD)$_2$]	dcype	23
9	KO^{t}Bu	[Ni(COD)$_2$]	PCy$_3$	32
10	KO^{t}Bu	[Ni(COD)$_2$]	P*n*Bu$_3$	41
11	KO^{t}Bu	[Ni(COD)$_2$]	dmpe	21
12	KO^{t}Bu	[Ni(COD)$_2$]	-	11
13[b]	KO^{t}Bu	[Ni(COD)$_2$]	ICy•HBF$_4$	0

Reaction conditions: **1a** (0.5 mmol, 1 equiv.), catalyst (5 mol%), ligand (10 mol%), base (2.5 equiv.), B_2(neop)$_2$ (2.5 equiv.), toluene (3 mL), at 110 °C for 20 h unless otherwise stated. [a] GC yields using dodecane as internal standard and are averages of 2 runs. [b] 2.5 equiv. B_2pin_2 was used instead of B_2(neop)$_2$.

Table S2: Screening of bases for the borylation of **1a**.

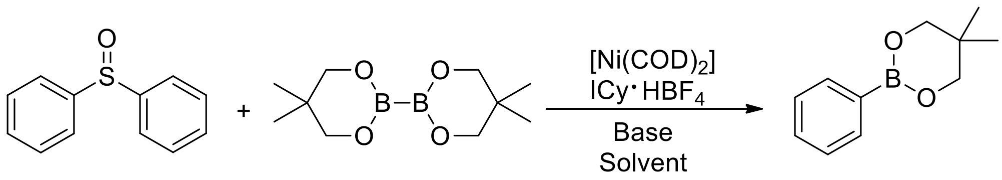

Entry	Base	Catalyst	Ligand	Yield of **3a** (%)[a]
1	KO^{t}Bu	[Ni(COD)$_2$]	ICy•HBF$_4$	61
2	KOMe	[Ni(COD)$_2$]	ICy•HBF$_4$	22
3	NaOMe	[Ni(COD)$_2$]	ICy•HBF$_4$	0
4	LiO^{t}Bu	[Ni(COD)$_2$]	ICy•HBF$_4$	0
5	NaO^{t}Bu	[Ni(COD)$_2$]	ICy•HBF$_4$	73
6[b]	K_3PO_4	[Ni(COD)$_2$]	ICy•HBF$_4$	0
7[b]	KOAc	[Ni(COD)$_2$]	ICy•HBF$_4$	0
8[b]	KF	[Ni(COD)$_2$]	ICy•HBF$_4$	0
9[c]	KOAc	[Ni(COD)$_2$]	ICy•HBF$_4$	0
10	-	[Ni(COD)$_2$]	ICy	0

Reaction conditions: **1a** (0.5 mmol, 1 equiv.), [Ni(COD)$_2$] (5 mol%), ICy•HBF$_4$ (10 mol%), base (2.5 equiv.), B_2(neop)$_2$ (2.5 equiv.), toluene (3 mL), at 110 °C for 20 h unless otherwise stated. [a] GC yields using dodecane as internal standard and are averages of 2 runs. [b] 10 mol% NaO^{t}Bu was added. [c] 2.5 equiv. 18-crown-6 was added.

Table S3: Screening of solvents for the borylation of **1a**.

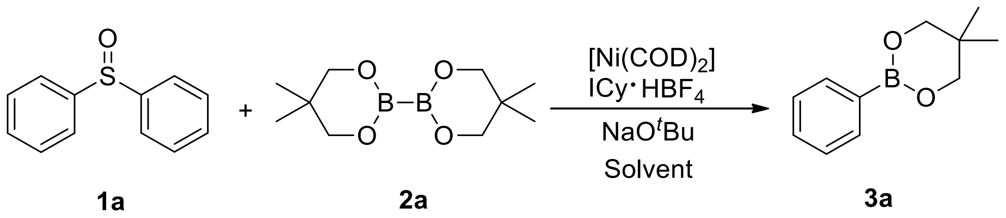

Entry	Solvent	Catalyst	Ligand	Yield of **3a** (%)[a]
1	Toluene	[Ni(COD)$_2$]	ICy•HBF$_4$	61
2	THF	[Ni(COD)$_2$]	ICy•HBF$_4$	55
3	MTBE	[Ni(COD)$_2$]	ICy•HBF$_4$	46
4	CH_3CN	[Ni(COD)$_2$]	ICy•HBF$_4$	14
5	DMF	[Ni(COD)$_2$]	ICy•HBF$_4$	0
6	Me-Cy	[Ni(COD)$_2$]	ICy•HBF$_4$	64
7	Hexane	[Ni(COD)$_2$]	ICy•HBF$_4$	47
8	1,4-dioxane	[Ni(COD)$_2$]	ICy•HBF$_4$	89

Reaction conditions: **1a** (0.5 mmol, 1 equiv.), [Ni(COD)$_2$] (5 mol%), ICy•HBF$_4$ (10 mol%), NaO^{t}Bu (2.5 equiv.), B_2(neop)$_2$ (2.5 equiv.), solvent (3 mL), at 110 °C for 20 h unless otherwise stated. [a] GC yields using dodecane as internal standard and are averages of 2 runs.

Table S4: Screening of catalysts for the borylation of **1a**.

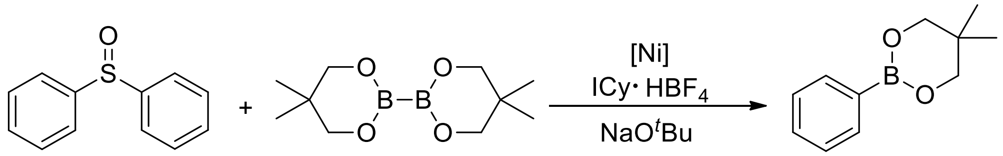

1a **2a** **3a**

Entry	Base	Catalyst	Ligand	Yield of **3a** (%)[a]
1	NaO^{t}Bu	[Ni(COD)$_2$]	ICy•HBF$_4$	93
2	NaO^{t}Bu	[NiCl$_2$]	ICy•HBF$_4$	63
3	NaO^{t}Bu	[NiCl$_2$(PPh$_3$)$_2$]	ICy•HBF$_4$	84
4	NaO^{t}Bu	[NiCl$_2$(dppp)]	ICy•HBF$_4$	24
5	NaO^{t}Bu	[NiCl$_2$(dppe)]	ICy•HBF$_4$	32
6	NaO^{t}Bu	[Ni(acac)$_2$]	ICy•HBF$_4$	62
7	NaO^{t}Bu	[Ni(OAc)$_2$]	ICy•HBF$_4$	66
8	NaO^{t}Bu	[Ni(IMes)$_2$]	-	26
9	NaO^{t}Bu	[Ni$_2$(ICy)$_4${μ-(η^2:η^2)-COD}]	-	88
10	NaO^{t}Bu	[Ni$_2$(I*i*Pr)$_4${μ-(η^2:η^2)-COD}]	-	79
11	NaO^{t}Bu	-	ICy•HBF$_4$	0

Reaction conditions: **1a** (0.5 mmol, 1 equiv), [Ni] (5 mol%), ICy•HBF$_4$ (10 mol%), NaO^{t}Bu (2.5 equiv.), B$_2$(neop)$_2$ (2.5 equiv.), 1,4-dioxane (3 mL), at 110 ^{o}C for 20 h unless otherwise stated. [a] GC yields using dodecane as internal standard and are averages of 2 runs.

1.3 General Procedure for the Synthesis of Sulfoxides

Synthesis of 1c-1e, 1j

These compounds were prepared as described previously.[6] A Schlenk flask was charged with thionyl chloride (0.73 mL, 10 mmol) and corresponding aromatic (25 mL) under argon. The mixture was cooled to 0 °C and stirred while 3.8 mL (43.0 mmol) of triflic acid was added. After 1 h, the resulting mixture was allowed to further stir at room temperature overnight. After the completion of the reaction, ice and saturated aqueous $NaHCO_3$ were added and the resulting biphasic solution was extracted with CH_2Cl_2 (30 mL × 3). The combined organic layer was dried over $MgSO_4$, and filtered through a pad of Celite (Ø 3 mm × 8 mm) followed by rotary evaporation. The product was isolated by flash column chromatography (hexane/ethyl acetate: 10/1).

Bis(4-methoxyphenyl) sulfoxide 1c

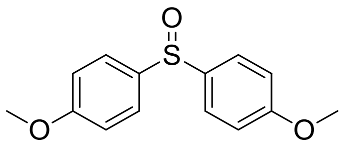

1c

Prepared from 10 mmol thionyl chloride and 25 mL of anisole. White solid (1.96 g, 7.5 mmol, 75%). **^{1}H NMR** (300 MHz, $CDCl_3$): δ = 7.54 – 7.49 (m, 4H), 6.96 – 6.91 (m, 4H), 3.79 (s, 6H). **^{13}C{^{1}H} NMR** (75 MHz, $CDCl_3$): δ = 161.9, 137.1, 127.0, 114.8, 55.6. **HRMS-ASAP** (m/z): Calculated (found) for $C_{14}H_{15}O_3S$ $[M+H]^+$ 263.0736 (263.0728).

The spectroscopic data for **1c** match those reported in the literature.[6]

Bis(4-chlorophenyl) sulfoxide 1d

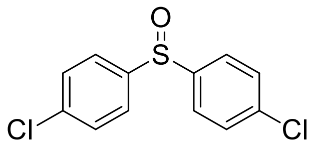

1d

Prepared from 10 mmol thionyl chloride and 25 mL of chlorobenzene. White solid (1.92 g, 7.1 mmol, 71%). **^{1}H NMR** (300 MHz, $CDCl_3$): δ = 7.59 – 7.54 (m, 4H), 7.46 – 7.42 (m, 4H). **^{13}C{^{1}H} NMR** (75 MHz, $CDCl_3$): δ = 144.0, 137.8, 129.9, 126.2. **HRMS-ASAP** (m/z): Calculated (found) for $C_{12}H_9Cl_2OS$ $[M+H]^+$ 270.9746 (270.9737).

The spectroscopic data for **1d** match those reported in the literature.[6]

Bis(4-fluorophenyl) sulfoxide 1e

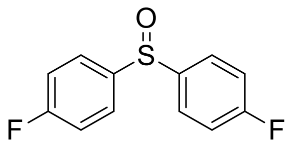

1e

Prepared from 5 mmol thionyl chloride and 10 mL of fluorobenzene. White solid (0.61 g, 2.55 mmol, 51%). ^{1}**H NMR** (500 MHz, $CDCl_3$): δ = 7.64 – 7.60 (m, 4H), 7.18 – 7.13 (m, 4H). ^{13}**C{^{1}H} NMR** (125 MHz, $CDCl_3$): δ = 164.5 (d, *J* = 251 Hz), 141.1 (d, *J* = 4 Hz), 127.2 (d, *J* = 10 Hz), 116.9 (d, *J* = 23 Hz). ^{19}**F{^{1}H} NMR** (470 MHz, $CDCl_3$): δ = -108.0. **HRMS-ASAP** (m/z): Calculated (found) for $C_{12}H_9F_2OS$ $[M+H]^+$ 239.0337 (239.0328).

The spectroscopic data for **1e** match those reported in the literature.[8]

Bis(1,3,5-trimethylphenyl) sulfoxide 1j

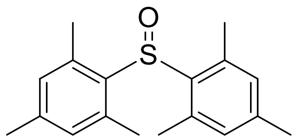

1j

Prepared from 5 mmol thionyl chloride and 10 mL of mesitylene. White solid (0.66 g, 2.3 mmol, 46%). ^{1}**H NMR** (300 MHz, $CDCl_3$): δ = 6.81 (s, 4H), 2.41 (s, 12H), 2.26 (s, 6H). ^{13}**C{^{1}H} NMR** (75 MHz, $CDCl_3$): δ = 140.6, 138.6, 136.6, 131.3, 21.0, 19.6. **HRMS-ASAP** (m/z): Calculated (found) for $C_{18}H_{23}OS$ $[M+H]^+$ 287.1464 (287.1456).

The spectroscopic data for **1j** match those reported in the literature.[6]

Synthesis of 1f-1i, 1k

These compounds were prepared as described previously.[7] A Schlenk flask was charged with thionyl chloride (0.40 mL, 5.5 mmol) and THF (5 mL) under argon. The mixture was cooled to 0 °C. 4-(Trifluoromethyl)phenylmagnesium bromide (ca. 1 M in THF, 10 mL) prepared from the corresponding aryl bromide (10 mmol) was added slowly and the resulting mixture was allowed to warm to room temperature. After the completion of the reaction, ice and saturated aqueous $NaHCO_3$ were added and the resulting biphasic solution was extracted with EtOAc (20 mL × 3). The combined organic layer was dried over $MgSO_4$, and filtered through a pad of Celite (Ø 3 mm × 8 mm) followed by rotary evaporation. The product was isolated by flash column chromatography (hexane/ethyl acetate: 10/1).

Bis[4-(trifluoromethyl)phenyl] sulfoxide 1f

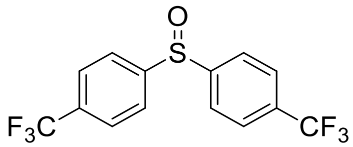

1f

Prepared from 10 mmol of 1-bromo-4-(trifluoromethyl)benzene. White solid (1.23 g, 3.65 mmol, 73%). **^{1}H NMR** (500 MHz, $CDCl_3$): δ = 7.82 – 7.79 (m, 4H), 7.76 – 7.73 (m, 4H). **^{13}C{^{1}H} NMR** (125 MHz, $CDCl_3$): δ = 149.3, 133.6 (q, J = 33 Hz), 126.8 (q, J = 4 Hz), 125.0, 123.4 (q, J = 271 Hz). **^{19}F{^{1}H} NMR** (470 MHz, $CDCl_3$): δ = -63.0. **HRMS-ASAP** (m/z): Calculated (found) for $C_{14}H_9F_6OS$ $[M+H]^+$ 339.0273 (339.0263).

The spectroscopic data for **1f** match those reported in the literature.[8]

Bis(3-methylphenyl) sulfoxide 1h

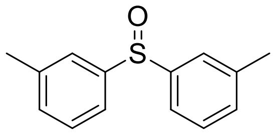

1h

Prepared from 10 mmol of 1-bromo-3-methylbenzene. White solid (0.78 g, 3.4 mmol, 68%). **^{1}H NMR** (300 MHz, $CDCl_3$): δ = 7.47 (s, 2H), 7.43 – 7.39 (m, 2H), 7.33 (t, J = 9 Hz, 2H), 7.25 – 7.21 (m, 2H), 2.37 (s, 6H). **^{13}C{^{1}H} NMR** (75 MHz, $CDCl_3$): δ = 145.5, 139.6, 132.0, 129.2, 125.1, 122.1, 21.5. **HRMS-ASAP** (m/z): Calculated (found) for $C_{14}H_{15}OS$ $[M+H]^+$ 231.0838 (231.0834).

The spectroscopic data for **1h** match those reported in the literature.[7]

Bis(2-methylphenyl) sulfoxide 1i

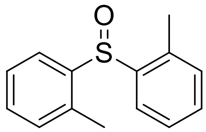

1i

Prepared from 10 mmol of 1-bromo-2-methylbenzene. White solid (0.61 g, 2.65 mmol, 53%). **^{1}H NMR** (300 MHz, $CDCl_3$): δ = 7.70 – 7.66 (m, 2H), 7.37 – 7.34 (m, 4H), 7.21 – 7.18 (m, 2H), 2.41 (s, 6H). **^{13}C{^{1}H} NMR** (75 MHz, $CDCl_3$): δ = 141.9, 136.8, 131.2, 131.0, 127.3, 126.2, 18.7. **HRMS-ASAP** (m/z): Calculated (found) for $C_{14}H_{15}OS$ $[M+H]^+$ 231.0838 (231.0834).

The spectroscopic data for **1i** match those reported in the literature.[7]

5,5'-Sulfinylbis(1,3-bis(trifluoromethyl)benzene) 1k

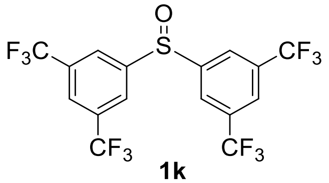

1k

Prepared from 10 mmol of 1-bromo-3,5-bis(trifluoromethyl)benzene. White solid (1.30 g, 2.75 mmol, 55%). **^{1}H NMR** (400 MHz, $CDCl_3$): δ = 8.16 (s, 4H), 8.02 (s, 2H). **^{13}C{^{1}H} NMR** (100 MHz, $CDCl_3$): δ = 147.8, 133.8 (q, J = 34 Hz), 126.0, 124.7, 122.5 (q, J = 272 Hz). **^{19}F{^{1}H} NMR** (376 MHz, $CDCl_3$): δ = -63.0. **HRMS-ASAP** (m/z): Calculated (found) for $C_{16}H_{16}F_{12}OS$ $[M]^+$ 473.9953 (473.9949).

The spectroscopic data for **1k** match those reported in the literature.[9]

Synthesis of 1l

This compound was prepared as described previously.[6] A Schlenk flask was charged with biphenyl (3.08 g, 20 mmol) and CH_2Cl_2 (15 mL) under argon. The mixture was cooled to 0 °C and stirred while thionyl chloride (0.37 mL, 5 mmol) and 1.9 mL (21.5 mmol) and triflic acid were added. After 1 h, the resulting mixture was allowed to further stir at room temperature overnight. After the completion of the reaction, ice and saturated aqueous $NaHCO_3$ were added and the resulting biphasic solution was extracted with CH_2Cl_2 (3 x 30 mL). The combined organic layer was dried over $MgSO_4$, and filtered through a pad of Celite (Ø 3 mm x 8 mm) followed by rotary evaporation. The product was isolated by flash column chromatography (hexane/ethyl acetate: 10/1).

4,4''-Sulfinyldi-1,1'-biphenyl 1l

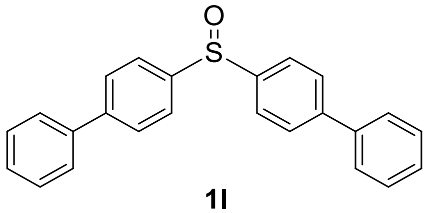

1l

White solid (1.19 g, 3.35 mmol, 67%). **^{1}H NMR** (300 MHz, $CDCl_3$): δ = 7.78 – 7.74 (m, 4H), 7.71 – 7.67 (m, 4H), 7.59 – 7.55 (m, 4H), 7.48 – 7.42 (m, 4H), 7.40 – 7.35 (m, 2H). **^{13}C{^{1}H} NMR** (75 MHz, $CDCl_3$): δ = 144.5, 144.3, 139.9, 129.1, 128.3, 128.25, 127.4, 125.5. **HRMS-ASAP** (m/z): Calculated (found) for $C_{18}H_{23}OS$ $[M+H]^+$ 355.1151 (355.1140).

The spectroscopic data for **1l** match those reported in the literature.[6]

Synthesis of 1m

This compound was prepared as described previously.[10] A Schlenk flask was charged with 2-bromonaphthalene (1.04 g, 5 mmol) and THF (15 mL) under argon. The mixture was cooled to -78 °C and stirred while *n*-BuLi (2.5 M solution in hexane, 2.2 mL, 5.5 mmol) was added dropwise. After 30 min, a solution of thionyl chloride (0.20 mL, 2.75 mmol) in THF (4 mL) was added dropwise. The reaction mixture was stirred at -78 °C for another 2 h. After 2 h, the resulting mixture was allowed to further stir at room temperature for 2 h. After completion of the reaction, *t*-BuOMe (20 mL), ice water (20 mL) and saturated aqueous $NaHCO_3$ were added and the resulting biphasic solution was extracted with *t*-BuOMe (3 x 20 mL). The combined organic layer was dried over $MgSO_4$, and filtered through a pad of Celite (Ø 3 mm x 8 mm) followed by rotary evaporation. The product was isolated by flash column chromatography (hexane/ethyl acetate: 4/1).

Bis(2-naphthyl) sulfoxide 1m

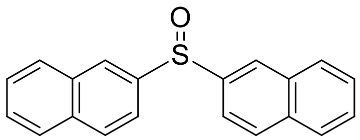

1m

White solid (0.33 g, 1.1 mmol, 44%). **^{1}H NMR** (300 MHz, $CDCl_3$): δ = 8.38 – 8.37 (m, 2H), 7.98 – 7.95 (m, 2H), 7.86 – 7.82 (m, 4H), 7.59 – 7.56 (m, 4H), 7.54 – 7.51 (m, 2H). **^{13}C{^{1}H} NMR** (75 MHz, $CDCl_3$): δ = 142.5, 134.5, 132.9, 129.9, 128.8, 128.2, 128.1, 127.4, 125.8, 121.0. **HRMS-ASAP** (m/z): Calculated (found) for $C_{20}H_{15}OS$ $[M+H]^+$ 303.0838 (303.0829).

The spectroscopic data for **1m** match those reported in the literature.[10]

Synthesis of 1n

This compound was prepared as described previously.[11] A Schlenk flask was charged with benzo[*b*]thiophene (1.3 g, 10 mmol) and THF (30 mL) under argon. The mixture was cooled to -78 °C and stirred while *n*-BuLi (2.5 M solution in hexane, 4.0 mL, 10 mmol) was added dropwise. After 30 min, a solution of thionyl chloride (0.40 mL, 5.5 mmol) in THF (5 mL) was added dropwise. After another 30 min at -78 °C, the reaction was warmed to room temperature. The reaction was quenched after 1 h with aqueous sat. NH_4Cl solution (20 mL). The resulting biphasic solution was extracted with *t*-BuOMe (3 x 20 mL). The combined organic layer was dried over $MgSO_4$, and filtered through a pad of Celite (Ø 3 mm x 8 mm) followed by rotary evaporation. The product was isolated by flash column chromatography (hexane/ethyl acetate: 4/1).

Bis(2-benzo[*b*]thienyl) sulfoxide 1n

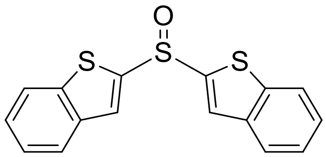

1n

White solid (0.487 g, 1.55 mmol, 31%). **^{1}H NMR** (300 MHz, $CDCl_3$): δ = 7.88 – 7.81 (m, 6H), 7.45 – 7.39 (m, 4H). **^{13}C{^{1}H} NMR** (75 MHz, $CDCl_3$): δ = 148.0, 142.2, 138.2, 127.2, 126.8, 125.40, 125.38, 123.0. **HRMS-ASAP** (m/z): Calculated (found) for $C_{16}H_{11}S_3O$ $[M+H]^+$ 314.9967 (314.9960).

The spectroscopic data for **1n** match those reported in the literature.[11]

Synthesis of 1o-1x

These compounds were prepared as described previously.[12] Synthesis of **1o** is representative. A glass tube was charged with K_2CO_3 (0.456 g, 3.3 mmol), CuI (2.5 mol%, 0.0143g), and *N*-methylpyrrolidinone (0.6 mL). The compounds 1-fluoro-4-iodobenzene (3 mmol, 0.67 g) and 2,6-dimethylbenzenethiol (3.6 mmol, 0.50 g) were added, and mixture was stirred at 100 °C for 16 h. After completion, the mixture was passed through a pad of Celite, and the filtrate was concentrated. The crude aryl sulfide was dissolved in DCM (10 mL) in an ice-water bath before *m*-CPBA (contains *ca*. 23 wt%, 3 mmol, 0.67 g) was added in portions. The mixture was allowed to warm to room temperature. After 12 h, saturated aqueous Na_2CO_3 were added and the resulting solution was extracted with EtOAc (3 x 30 mL). The combined organic layer was dried over $MgSO_4$, and filtered through a pad of Celite (Ø 3 mm x 8 mm). The product was isolated by flash column chromatography (hexane/ethyl acetate: 5/1).

2,6-Dimethylphenyl 4-fluorophenyl sulfoxide 1o

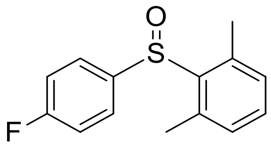

1o

White solid (0.66 g, 2.64 mmol, 88%). **^{1}H NMR** (500 MHz, $CDCl_3$): δ = 7.43 – 7.40 (m, 2H), 7.27 (t, *J* = 8 Hz, 1H), 7.15 – 7.11 (m, 2H), 7.06 (d, *J* = 8 Hz, 2H), 2.46 (s, 6H). **^{13}C{^{1}H} NMR** (125 MHz, $CDCl_3$): δ = 163.6 (d, *J* = 249 Hz), 140.0, 139.7 (d, *J* = 4 Hz), 139.5, 132.0, 130.3, 126.8 (d, *J* = 9 Hz), 116.3 (d, *J* = 23 Hz), 19.5. **^{19}F{^{1}H} NMR** (470 MHz, $CDCl_3$): δ = -111.1. **HRMS-ASAP** (m/z): Calculated (found) for $C_{14}H_{14}FOS$ $[M+H]^+$ 249.0744 (249.0738).

2,6-Dimethylphenyl 4-methoxyphenyl sulfoxide 1p

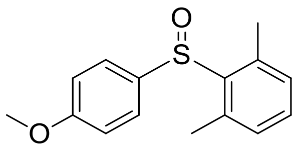

1p

Prepared from 3 mmol of 1-iodo-4-methoxybenzene. White solid (0.67 g, 2.58 mmol, 86%). **^{1}H NMR** (300 MHz, $CDCl_3$): δ = 7.35 (d, J = 8 Hz, 2H), 7.25 (t, J = 8 Hz, 1H), 7.05 (d, J = 8 Hz, 2H), 6.95 (d, J = 8 Hz, 2H), 3.82 (s, 3H), 2.47 (s, 6H). **^{13}C{^{1}H} NMR** (75 MHz, $CDCl_3$): δ = 160.9, 140.0, 139.8, 135.1, 131.7, 130.2, 126.3, 114.6, 55.6, 19.5. **HRMS-ASAP** (m/z): Calculated (found) for $C_{15}H_{17}O_2S$ $[M+H]^+$ 261.0944 (261.0933).

The spectroscopic data for **1p** match those reported in the literature.[7]

2,6-Dimethylphenyl 4-(trimethylsilyl)phenyl sulfoxide 1q

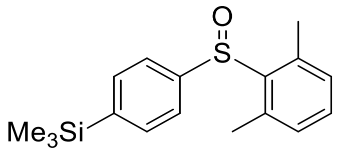

1q

Prepared from 3 mmol of (4-iodophenyl)trimethylsilane. White solid (0.64 g, 2.13 mmol, 71%). **^{1}H NMR** (300 MHz, $CDCl_3$): δ = 7.59 – 7.55 (m, 2H), 7.42 – 7.38 (m, 2H), 7.26 (t, J = 8 Hz, 1H), 7.06 (d, J = 8 Hz, 2H), 2.48 (s, 6H), 0.26 (s, 9H). **^{13}C{^{1}H} NMR** (75 MHz, $CDCl_3$): δ = 144.7, 142.9, 140.1, 139.8, 133.9, 131.8, 130.1, 123.8, 19.6, -1.1. **HRMS-ASAP** (m/z): Calculated (found) for $C_{17}H_{23}OSSi$ $[M+H]^+$ 303.1233 (303.1221).

4-((2,6-Dimethylphenyl)sulfinyl)-*N,N*-dimethylaniline 1r

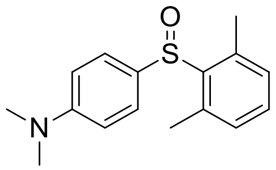

1r

Prepared from 3 mmol of 4-iodo-*N,N*-dimethylaniline. White solid (0.43 g, 1.59 mmol, 53%). **^{1}H NMR** (300 MHz, $CDCl_3$): δ = 7.21 – 7.12 (m, 2H), 7.16 (t, J = 9 Hz, 1H), 6.93 (d, J = 9 Hz, 2H), 6.62 (d, J = 9 Hz, 2H), 2.89 (s, 6H), 2.46 (s, 6H). **^{13}C{^{1}H} NMR** (75 MHz, $CDCl_3$): δ = 148.7, 143.5, 132.9, 128.7, 128.67, 128.4, 113.6, 40.8, 22.2. **HRMS-ASAP** (m/z): Calculated (found) for $C_{16}H_{20}NOS$ $[M+H]^+$ 274.1260 (274.1248).

1-(4-((2,6-Dimethylphenyl)sulfinyl)phenyl)-1*H*-pyrrole 1s

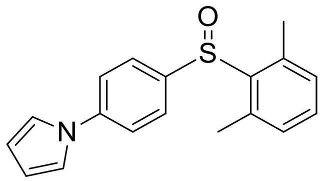

1s

Prepared from 3 mmol of 1-(4-iodophenyl)-1*H*-pyrrole. White solid (0.66 g, 2.25 mmol, 75%). **^{1}H NMR** (500 MHz, $CDCl_3$): δ = 7.50 – 7.47 (m, 2H), 7.47– 7.44 (m, 2H), 7.29 (t, *J* = 8 Hz, 1H), 7.10 (t, *J* = 3 Hz, 2H), 7.08 (d, *J* = 8 Hz, 2H), 6.36 (t, *J* = 3 Hz, 2H), 2.50 (s, 6H). **^{13}C{^{1}H} NMR** (125 MHz, $CDCl_3$): δ = 141.8, 140.8, 140.1, 139.6, 132.0, 130.3, 126.2, 120.5, 119.2, 111.4, 19.6. **HRMS-ASAP** (m/z): Calculated (found) for $C_{18}H_{18}NOS$ $[M+H]^+$ 296.1104 (296.1090).

4-(4-((2,6-Dimethylphenyl)sulfinyl)phenyl)morpholine 1t

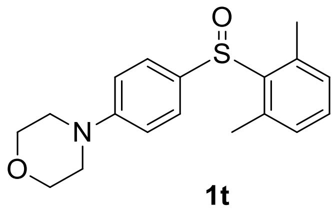

1t

Prepared from 3 mmol of 4-(4-iodophenyl)morpholine. White solid (0.64 g, 2.04 mmol, 68%). **^{1}H NMR** (300 MHz, $CDCl_3$): δ = 7.30 (d, *J* = 8 Hz, 2H), 7.25 (t, *J* = 8 Hz, 1H), 7.04 (d, *J* = 8 Hz, 2H), 6.92 (d, *J* = 8 Hz, 2H), 3.84 (t, *J* = 5 Hz, 4H), 3.19 (t, *J* = 5 Hz, 4H), 2.48 (s, 6H). **^{13}C{^{1}H} NMR** (75 MHz, $CDCl_3$): δ = 152.2, 140.0, 139.8, 131.6, 130.1, 128.6, 126.1, 115.3, 66.8, 48.6, 19.5. **HRMS-ASAP** (m/z): Calculated (found) for $C_{18}H_{22}NO_2S$ $[M+H]^+$ 316.1366 (316.1352).

5-((2,6-Dimethylphenyl)sulfinyl)benzo[*d*][1,3]dioxole 1u

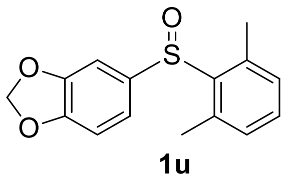

1u

Prepared from 3 mmol of 5-iodobenzo[*d*][1,3]dioxole. White solid (0.62 g, 2.25 mmol, 75%). **^{1}H NMR** (300 MHz, $CDCl_3$): δ = 7.26 (t, *J* = 8 Hz, 1H), 7.06 (s, 1H), 7.06 – 7.04 (m, 1H), 6.97 – 6.94 (m, 1H), 6.87 – 6.82 (m, 2H), 5.99 (s, 2H), 2.48 (s, 6H). **^{13}C{^{1}H} NMR** (75 MHz, $CDCl_3$): δ = 149.2, 148.5, 140.0, 139.6, 137.3, 131.8, 130.2, 119.1, 108.8, 105.2, 101.9, 19.5. **HRMS-ASAP** (m/z): Calculated (found) for $C_{15}H_{15}O_3S$ $[M+H]^+$ 275.0736 (275.0725).

5-((2,6-Dimethylphenyl)sulfinyl)benzofuran 1v

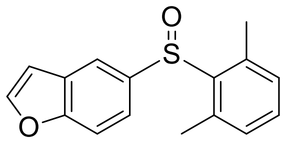

1v

Prepared from 3 mmol of 5-iodobenzofuran. White solid (0.50 g, 1.86 mmol, 62%). **^{1}H NMR** (300 MHz, $CDCl_3$): *δ* = 7.79 (s, 1H), 7.69 – 7.68 (m, 1H), 7.53 (d, *J* = 9 Hz, 1H), 7.27 (t, *J* = 9 Hz, 1H), 7.26 (d, *J* = 9 Hz, 1H), 7.07 (d, *J* = 9 Hz, 2H), 6.80 – 6.79 (m, 1H), 2.49 (s, 6H). **^{13}C{^{1}H} NMR** (75 MHz, $CDCl_3$): *δ* = 155.6, 146.7, 140.1, 140.0, 138.4, 131.8, 130.2, 128.2, 120.6, 118.3, 112.2, 106.9, 19.6. **HRMS-ASAP** (m/z): Calculated (found) for $C_{16}H_{15}O_2S$ $[M+H]^+$ 271.0787 (271.0781).

3-((2,6-Dimethylphenyl)sulfinyl)pyridine 1w

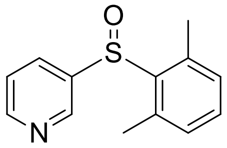

1w

Prepared from 3 mmol of 3-iodopyridine. White solid (0.37 g, 1.59 mmol, 53%). **^{1}H NMR** (300 MHz, $CDCl_3$): *δ* = 8.64 (d, *J* = 5 Hz, 1H), 8.50 (s, 1H), 7.92 – 7.88 (m, 1H), 7.41 (dd, *J* = 8, 5 Hz, 1H), 7.29 (t, *J* = 8 Hz, 1H), 7.08 (d, *J* = 8 Hz, 2H), 2.47 (s, 6H). **^{13}C{^{1}H} NMR** (75 MHz, $CDCl_3$): *δ* = 150.7, 146.0, 141.0, 140.0, 138.9, 132.9, 132.4, 130.4, 123.8, 19.5. **HRMS-ASAP** (m/z): Calculated (found) for $C_{13}H_{14}NOS$ $[M+H]^+$ 232.0791 (232.0786).

2-((2,6-Dimethylphenyl)sulfinyl)thiophene 1x

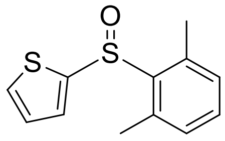

1x

Prepared from 3 mmol of 2-iodothiophene. White solid (0.47 g, 1.98 mmol, 66%). **^{1}H NMR** (300 MHz, $CDCl_3$): *δ* = 7.54 – 7.51 (m, 1H), 7.31 – 7.28 (m, 2H), 7.10 – 7.07 (m, 3H), 2.55 (s, 6H). **^{13}C{^{1}H} NMR** (75 MHz, $CDCl_3$): *δ* = 145.9, 139.7, 139.3, 131.8, 130.7, 130.3, 129.5, 128.0, 19.5. **HRMS-ASAP** (m/z): Calculated (found) for $C_{12}H_{13}OS_2$ $[M+H]^+$ 237.0402 (237.0398).

1-Methoxy-4-((4-(trifluoromethyl)phenyl)sulfinyl)benzene 1y

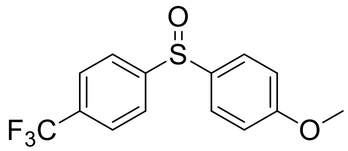

1y

Prepared from 3 mmol of 1-iodo-4-(trifluoromethyl)benzene and 3.6 mmol of 4-methoxybenzenethiol. White solid (0.73 g, 2.43 mmol, 81%). **^{1}H NMR** (500 MHz, $CDCl_3$): δ = 7.74 – 7.69 (m, 4H), 7.59 – 7.56 (m, 2H), 6.98 – 6.95 (m, 2H), 3.82 (s, 3H). **^{13}C{^{1}H} NMR** (125 MHz, $CDCl_3$): δ = 162.6, 150.4 (q, J = 1 Hz), 136.1, 132.7 (q, J = 33 Hz), 127.6, 126.3 (q, J = 4 Hz), 124.9, 123.6 (q, J = 270 Hz), 115.2, 55.7. **^{19}F{^{1}H} NMR** (470 MHz, $CDCl_3$): δ = -62.8. **HRMS-ASAP** (m/z): Calculated (found) for $C_{14}H_{12}F_3O_2S$ $[M+H]^+$ 301.0505 (301.0492).

The spectroscopic data for **1y** match those reported in the literature.[13]

1.4 Details of the Catalytic Borylation of Aryl Sulfoxides

1.4.1 General Procedure for Catalyst Screening

In an argon-filled glovebox, the catalyst and the solvent were added to a 10 mL thick-walled reaction tube equipped with a magnetic stirring bar. The base, the boron reagent, and the aryl sulfoxide were added. The reaction mixture was stirred at 110 °C for 20 h, then diluted with Et_2O (2 mL) and filtered through a pad of Celite (Ø 3 mm x 8 mm). Dodecane was added as an internal standard and the crude reaction mixture was analyzed by GC-MS.

1.4.2 General Procedures for the Synthesis of Organoboronic Esters

In an argon-filled glovebox, $[Ni(COD)_2]$ (5 mol%), ICy•HBF_4 (10 mol%) and 1,4-dioxane (3 mL) were added to a 10 mL thick-walled reaction tube equipped with a magnetic stirring bar. NaO^{t}Bu (1.25 mmol, 2.5 equiv.), the boron reagent (1.25 mmol, 2.5 equiv.) and the aryl sulfoxide (0.5 mmol, 1.0 equiv.) were added. The reaction mixture was stirred at 110 °C for 20 h, then diluted with Et_2O (2 mL) and filtered through a pad of Celite (Ø 3 mm x 8 mm). The product was isolated by flash column chromatography (hexane/ethyl acetate (95/5)) after careful removal of the solvent *in vacuo*. The reactions were commonly performed on a 500 µmol scale. All aryl boronate products were reported previously and were unambiguously identified by comparison of HRMS and ^{1}H, ^{13}C{^{1}H}, ^{11}B{^{1}H} and/or ^{19}F{^{1}H} NMR spectra with literature data. The boron-bonded carbon atom was not detected for all compounds, due to quadrupolar broadening by the ^{11}B nucleus.

<u>5,5-Dimethyl-2-phenyl-1,3,2-dioxaborinane 3a</u>

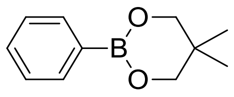

3a

Yield: 77.0 mg (405 µmol, 81%) of a colorless liquid. **^{1}H NMR** (300 MHz, $CDCl_3$): δ = 7.81 (d, J = 8 Hz, 2H), 7.43 – 7.41 (m, 1H), 7.38 – 7.32 (m, 2H), 3.78 (s, 4H), 1.03 (s, 6H). **^{13}C{^{1}H} NMR** (75 MHz, $CDCl_3$): δ = 134.0, 130.8, 127.7, 72.5, 32.0, 22.1. **^{11}B{^{1}H} NMR** (96 MHz, $CDCl_3$): δ = 26.8. **HRMS-ASAP** (m/z): Calculated (found) for $C_{11}H_{15}BO_2$ $[M]^+$ 190.1160 (190.1157).

The spectroscopic data for **3a** match those reported in the literature.[14]

2-(4-Methyl-phenyl)-5,5-dimethyl-[1,3,2]dioxaborinane 3b

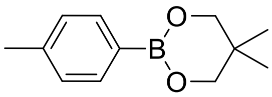

3b

Yield: 87.8 mg (430 μmol, 86%) of a colorless solid. ^{1}**H NMR** (500 MHz, $CDCl_3$): *δ* = 7.71 (d, *J* = 8 Hz, 2H), 7.19 (d, *J* = 8 Hz, 2H), 3.77 (s, 4H), 2.37 (s, 3H), 1.03 (s, 6H). ^{13}**C{^{1}H} NMR** (125 MHz, $CDCl_3$): *δ* = 140.8, 134.0, 128.5, 72.4, 32.0, 22.1, 21.8. ^{11}**B{^{1}H} NMR** (160 MHz, $CDCl_3$): *δ* = 27.0. **HRMS-ASAP** (m/z): Calculated (found) for $C_{12}H_{17}BO_2$ $[M]^+$ 204.1316 (204.1315).

The spectroscopic data for **3b** match those reported in the literature.[14]

2-(4-Methoxyphenyl)-5,5-dimethyl-[1,3,2]dioxaborinane 3c

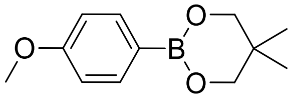

3c

Yield: 54.0 mg (245 μmol, 49%) of a colorless solid. ^{1}**H NMR** (500 MHz, $CDCl_3$): *δ* = 7.74 (d, *J* = 9 Hz, 2H), 6.89 (d, *J* = 9 Hz, 2H), 3.82 (s, 3H), 3.75 (s, 4H), 1.02 (s, 6H). ^{13}**C{^{1}H} NMR** (125 MHz, $CDCl_3$): *δ* = 161.9, 135.7, 113.3, 72.4, 55.2, 32.1, 22.1. ^{11}**B{^{1}H} NMR** (160 MHz, $CDCl_3$): *δ* = 27.1. **HRMS-ASAP** (m/z): Calculated (found) for $C_{12}H_{18}BO_3$ $[M+H]^+$ 221.1344 (221.1336).

The spectroscopic data for **3c** match those reported in the literature.[14]

2-(4-Chlorophenyl)-5,5-dimethyl-[1,3,2]dioxaborinane 3d

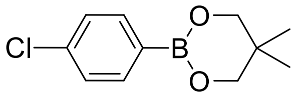

3d

Yield: 63.0 mg (280 μmol, 56%) of a colorless solid. ^{1}**H NMR** (300 MHz, $CDCl_3$): *δ* = 7.72 (d, *J* = 8 Hz, 2H), 7.32 (d, *J* = 8 Hz, 2H), 3.76 (s, 4H), 1.02 (s, 6H). ^{13}**C{^{1}H} NMR** (75 MHz, $CDCl_3$): *δ* = 137.0, 135.4, 128.0, 72.5, 32.0, 22.0. ^{11}**B{^{1}H} NMR** (96 MHz, $CDCl_3$): *δ* = 26.5. **HRMS-ASAP** (m/z): Calculated (found) for $C_{11}H_{15}BClO_2$ $[M+H]^+$ 225.0848 (225.0842).

The spectroscopic data for **3d** match those reported in the literature.[15]

2-(4-Fluorophenyl)-5,5-dimethyl-[1,3,2]dioxaborinane 3e

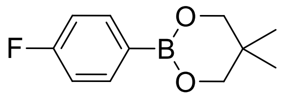

3e

Yield: 81.1 mg (390 µmol, 78%) of a colorless solid. **^{1}H NMR** (500 MHz, $CDCl_3$): δ = 7.81 – 7.77 (m, 2H), 7.05 – 7.01 (m, 2H), 3.76 (s, 4H), 1.02 (s, 6H). **^{13}C{^{1}H} NMR** (125 MHz, $CDCl_3$): δ = 165.0 (d, J = 248 Hz), 136.1 (d, J = 8 Hz), 114.7 (d, J = 20 Hz), 72.5, 32.0, 22.0. **^{11}B{^{1}H} NMR** (160 MHz, $CDCl_3$): δ = 26.6. **^{19}F{^{1}H} NMR** (470 MHz, $CDCl_3$): δ = -109.9 (s). **HRMS-ASAP** (m/z): Calculated (found) for $C_{11}H_{15}BFO_2$ $[M+H]^+$ 209.1144 (209.1137).

The spectroscopic data for **3e** match those reported in the literature.[14]

2-(4-Trifluoromethyl-phenyl)-5,5-dimethyl-[1,3,2]dioxaborinane 3f

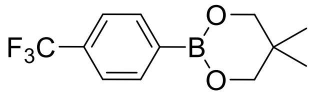

3f

Yield: 94.0 mg (365 µmol, 73%) of a pale yellow solid. **^{1}H NMR** (500 MHz, $CDCl_3$): δ = 7.90 (d, J = 8 Hz, 2H), 7.60 (d, J = 8 Hz, 2H), 3.79 (s, 4H), 1.03 (s, 6H). **^{13}C{^{1}H} NMR** (125 MHz, $CDCl_3$): δ = 134.2, 132.4 (q, J = 32 Hz), 124.3 (q, J = 4 Hz), 124.4 (q, J = 272 Hz), 72.5, 32.0, 22.0. **^{11}B{^{1}H} NMR** (160 MHz, $CDCl_3$): δ = 26.5. **^{19}F{^{1}H} NMR** (470 MHz, $CDCl_3$): δ = -62.9 (s). **HRMS-ASAP** (m/z): Calculated (found) for $C_{12}H_{14}BF_3O_2$ $[M]^+$ 258.1033 (258.1021).

The spectroscopic data for **3f** match those reported in the literature.[16]

2-(4-(Methylthio)phenyl)-5,5-dimethyl-[1,3,2]dioxaborinane 3g

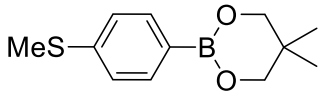

3g

Yield: 79.1 mg (335 µmol, 67%) of a colorless solid. **^{1}H NMR** (300 MHz, $CDCl_3$): δ = 7.71 (d, J = 8 Hz, 2H), 7.22 (d, J = 8 Hz, 2H), 3.76 (s, 4H), 2.49 (s, 3H), 1.02 (s, 6H). **^{13}C{^{1}H} NMR** (75 MHz, $CDCl_3$): δ = 141.8, 134.4, 125.2, 72.4, 32.0, 22.1, 15.3. **^{11}B{^{1}H} NMR** (96 MHz, $CDCl_3$): δ = 26.7. **HRMS-ASAP** (m/z): Calculated (found) for $C_{12}H_{18}BOS$ $[M+H]^+$ 237.1115 (237.1106).

The spectroscopic data for **3g** match those reported in the literature.[17]

2-(3-Methyl-phenyl)-5,5-dimethyl-[1,3,2]dioxaborinane 3h

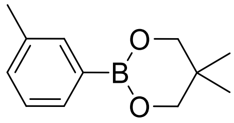

3h

Yield: 78.6 mg (385 µmol, 77%) of a colorless solid. **^{1}H NMR** (300 MHz, $CDCl_3$): δ = 7.65 – 7.61 (m, 2H), 7.31 – 7.26 (m, 2H), 3.79 (s, 4H), 2.37 (s, 3H), 1.04 (s, 6H). **^{13}C{^{1}H} NMR** (75 MHz, $CDCl_3$): δ = 137.1, 134.6, 131.6, 131.0, 127.7, 72.5, 32.0, 22.0, 21.5. **^{11}B{^{1}H} NMR** (96 MHz, $CDCl_3$): δ = 26.9. **HRMS-ASAP** (m/z): Calculated (found) for $C_{12}H_{18}BOS$ $[M+H]^+$ 205.1394 (205.1389).

The spectroscopic data for **3h** match those reported in the literature.[14]

2-(3,5-Bis(trifluoromethyl)phenyl)-5,5-dimethyl-1,3,2-dioxaborinane 3k

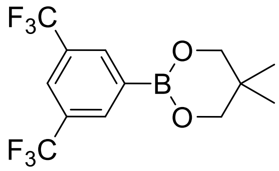

3k

Yield: 110.8 mg (340 µmol, 68%) of a pale yellow solid. **^{1}H NMR** (500 MHz, $CDCl_3$): δ = 8.23 (s, 2H), 7.91 (s, 1H), 3.81 (s, 4H), 1.04 (s, 6H). **^{13}C{^{1}H} NMR** (125 MHz, $CDCl_3$): δ = 134.0 (m), 130.8 (q, J = 34 Hz), 124.3 (q, J = 5 Hz), 123.8 (q, J = 271 Hz), 72.6, 32.1, 22.0. **^{11}B{^{1}H} NMR** (160 MHz, $CDCl_3$): δ = 26.1. **^{19}F{^{1}H} NMR** (470 MHz, $CDCl_3$): δ = -62.8 (s). **HRMS-ASAP** (m/z): Calculated (found) for $C_{13}H_{14}BF_6O_2$ $[M]^+$ 327.0986 (327.0975).

The spectroscopic data for **3k** match those reported in the literature.[18]

2-(Biphenyl-4-yl)-5,5-dimethyl-[1,3,2]dioxaborinane 3l

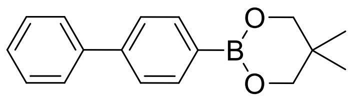

3l

Yield: 103.8 mg (390 µmol, 78%) of a colorless solid. **^{1}H NMR** (500 MHz, $CDCl_3$): δ = 7.91 – 7.89 (m, 2H), 7.65 – 7.61 (m, 4H), 7.47 – 7.44 (m, 2H), 7.38 – 7.35 (m, 1H), 3.81 (s, 4H), 1.04 (s, 6H). **^{13}C{^{1}H} NMR** (125 MHz, $CDCl_3$): δ = 143.4, 141.3, 134.5, 128.9, 127.5, 127.3, 126.5, 72.5, 32.0, 22.1. **^{11}B{^{1}H} NMR** (160 MHz, $CDCl_3$): δ = 27.0. **HRMS-ASAP** (m/z): Calculated (found) for $C_{17}H_{20}BO_2$ $[M+H]^+$ 267.1551 (267.1541).

The spectroscopic data for **3l** match those reported in the literature.[17]

2-(Naphthalen-2-yl)-5,5-dimethyl-[1,3,2]dioxaborinane 3m

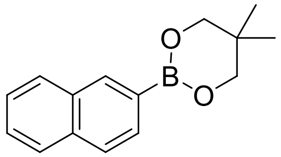

3m

Yield: 85.0 mg (355 µmol, 71%) of a colorless solid. ^{1}**H NMR** (300 MHz, $CDCl_3$): δ = 8.35 (s, 1H), 7.90 – 7.80 (m, 4H), 7.50 – 7.43 (m, 2H), 3.84 (s, 4H), 1.06 (s, 6H). ^{13}**C{^{1}H} NMR** (75 MHz, $CDCl_3$): δ = 135.2, 135.0, 133.1, 130.1, 128.8, 127.8, 126.9, 126.8, 125.7, 72.6, 32.1, 22.1. ^{11}**B{^{1}H} NMR** (96 MHz, $CDCl_3$): δ = 27.0. **HRMS-ASAP** (m/z): Calculated (found) for $C_{15}H_{18}BO_2$ $[M+H]^+$ 241.1394 (241.1386).

The spectroscopic data for **3m** match those reported in the literature.[17]

2-(Benzo[*b*]thiophen-2-yl)-5,5-dimethyl-1,3,2-dioxaborinane 3n

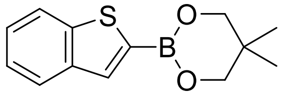

3n

Yield: 93.5 mg (380 µmol, 76%) of a colorless solid. ^{1}**H NMR** (300 MHz, $CDCl_3$): δ = 7.91 – 7.88 (m, 1H), 7.86 – 7.81 (m, 2H) 7.36 – 7.33 (m, 2H), 3.81 (s, 4H), 1.06 (s, 6H). ^{13}**C{^{1}H} NMR** (75 MHz, $CDCl_3$): δ = 143.5, 140.8, 133.0, 125.1, 124.4, 124.1, 122.7, 72.7, 32.2, 22.1. ^{11}**B{^{1}H} NMR** (96 MHz, $CDCl_3$): δ = 25.7. **HRMS-ASAP** (m/z): Calculated (found) for $C_{13}H_{16}BO_2S$ $[M+H]^+$ 247.0959 (247.0953).

The spectroscopic data for **3n** match those reported in the literature.[19]

(4-(5,5-Dimethyl-1,3,2-dioxaborinan-2-yl)phenyl)trimethylsilane 3q

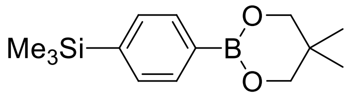

3q

Yield: 111.4 mg (425 µmol, 85%) of a colorless solid. ^{1}**H NMR** (300 MHz, $CDCl_3$): δ = 7.78 (d, J = 8 Hz, 2H), 7.53 (d, J = 8 Hz, 2H), 3.77 (s, 4H), 1.02 (s, 6H), 0.27 (s, 9H). ^{13}**C{^{1}H} NMR** (75 MHz, $CDCl_3$): δ = 143.5, 133.1, 132.7, 72.4, 32.0, 22.0, -1.1. ^{11}**B{^{1}H} NMR** (96 MHz, $CDCl_3$): δ = 26.8. **HRMS-ASAP** (m/z): Calculated (found) for $C_{14}H_{24}BO_2Si$ $[M+H]^+$ 263.1633 (263.1627).

The spectroscopic data for **3q** match those reported in the literature.[20]

4-(5,5-Dimethyl-1,3,2-dioxaborinan-2-yl)-*N,N*-dimethylaniline 3r

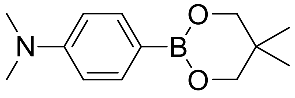

3r

Yield: 78.1 mg (335 μmol, 67%) of a colorless solid. **^{1}H NMR** (500 MHz, $CDCl_3$): *δ* = 7.69 (d, *J* = 9 Hz, 2H), 6.71 (d, *J* = 9 Hz, 2H), 3.75 (s, 4H), 2.99 (s, 6H), 1.02 (s, 6H). **^{13}C{^{1}H} NMR** (125 MHz, $CDCl_3$): *δ* = 152.4, 135.3, 111.5, 72.4, 40.4, 32.1, 22.1. **^{11}B{^{1}H} NMR** (160 MHz, $CDCl_3$): *δ* = 27.0. **HRMS-ASAP** (m/z): Calculated (found) for $C_{13}H_{21}BNO_2$ $[M+H]^+$ 234.1660 (234.1656).

The spectroscopic data for **3r** match those reported in the literature.[21]

1-(4-(5,5-Dimethyl-1,3,2-dioxaborinan-2-yl)phenyl)-1*H*-pyrrole 3s

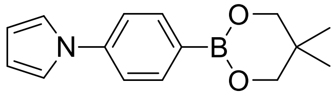

3s

Yield: 99.5 mg (390 μmol, 78%) of a colorless solid. **^{1}H NMR** (500 MHz, $CDCl_3$): *δ* = 7.86 (d, *J* = 9 Hz, 2H), 7.39 (d, *J* = 9 Hz, 2H), 7.14 (t, *J* = 3 Hz, 2H), 6.35 (t, *J* = 3 Hz, 2H), 3.79 (s, 4H), 1.04 (s, 6H). **^{13}C{^{1}H} NMR** (125 MHz, $CDCl_3$): *δ* = 142.6, 135.4, 119.4, 119.3, 110.7, 72.5, 32.1, 22.1. **^{11}B{^{1}H} NMR** (160 MHz, $CDCl_3$): *δ* = 26.8. **HRMS-ASAP** (m/z): Calculated (found) for $C_{15}H_{19}BNO_2$ $[M+H]^+$ 256.1503 (256.1494).

The spectroscopic data for **3s** match those reported in the literature.[21]

4-(4-(5,5-Dimethyl-1,3,2-dioxaborinan-2-yl)phenyl)morpholine 3t

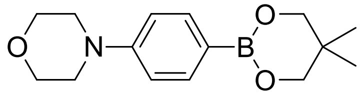

3t

Yield: 100.4 mg (365 μmol, 73%) of a colorless solid. **^{1}H NMR** (500 MHz, $CDCl_3$): *δ* = 7.72 (d, *J* = 9 Hz, 2H), 6.89 (d, *J* = 9 Hz, 2H), 3.86 (t, *J* = 5 Hz, 4H), 3.75 (s, 4H), 3.22 (t, *J* = 5 Hz, 4H), 1.01 (s, 6H). **^{13}C{^{1}H} NMR** (125 MHz, $CDCl_3$): *δ* = 153.1, 135.3, 114.3, 72.4, 67.0, 48.7, 32.0, 22.1. **^{11}B{^{1}H} NMR** (160 MHz, $CDCl_3$): *δ* = 26.9. **HRMS-ASAP** (m/z): Calculated (found) for $C_{15}H_{23}BNO_3$ $[M+H]^+$ 276.1766 (276.1756).

The spectroscopic data for **3t** match those reported in the literature.[17]

2-(Benzo[*d*][1,3]dioxol-5-yl)-5,5-dimethyl-1,3,2-dioxaborinane 3u

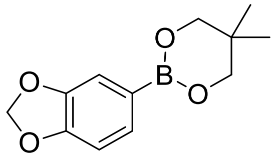

3u

Yield: 74.9 mg (320 µmol, 64%) of a colorless solid. **^{1}H NMR** (300 MHz, $CDCl_3$): δ = 7.35 (d, J = 8 Hz, 1H), 7.24 (s, 1H), 6.82 (d, J = 8 Hz, 1H), 5.94 (s, 2H), 3.74 (s, 4H), 1.01 (s, 6H). **^{13}C{^{1}H} NMR** (75 MHz, $CDCl_3$): δ = 149.8, 147.3, 128.7, 113.4, 108.2, 100.8, 72.4, 32.0, 22.0. **^{11}B{^{1}H} NMR** (96 MHz, $CDCl_3$): δ = 26.4. **HRMS-ASAP** (m/z): Calculated (found) for $C_{12}H_{16}BO_4$ $[M+H]^+$ 235.1136 (235.1127).

The spectroscopic data for **3u** match those reported in the literature.[17]

2-(Benzofuran-5-yl)-5,5-dimethyl-1,3,2-dioxaborinane 3v

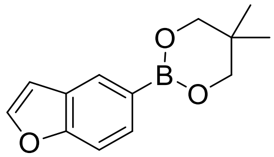

3v

Yield: 75.9 mg (330 µmol, 66%) of a colorless solid. **^{1}H NMR** (300 MHz, $CDCl_3$): δ = 8.10 (s, 1H), 7.77 (dd, J = 8, 1 Hz, 1H), 7.60 (d, J = 2 Hz, 1H), 7.49 (d, J = 8 Hz, 1H), 6.77 (dd, J = 2, 1 Hz, 1H), 3.80 (s, 4H), 1.04 (s, 6H). **^{13}C{^{1}H} NMR** (75 MHz, $CDCl_3$): δ = 157.0, 144.8, 130.1, 127.7, 127.2, 110.8, 106.9, 72.5, 32.1, 22.1. **^{11}B{^{1}H} NMR** (96 MHz, $CDCl_3$): δ = 26.9. **HRMS-ASAP** (m/z): Calculated (found) for $C_{13}H_{16}BO_3$ $[M+H]^+$ 231.1187 (231.1179).

The spectroscopic data for **3v** match those reported in the literature.[21]

3-(5,5-Dimethyl-1,3,2-dioxaborinan-2-yl)pyridine 3w

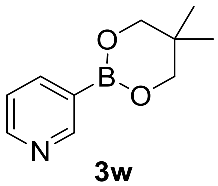

3w

Yield: 41.1 mg (215 µmol, 43%) of a colorless solid. **^{1}H NMR** (300 MHz, $CDCl_3$): δ = 8.94 (s, 1H), 8.64 (d, J = 8 Hz, 1H), 8.04 (d, J = 8 Hz, 1H), 7.28 – 7.24 (m, 1H), 3.77 (s, 4H), 1.03 (s, 6H). **^{13}C{^{1}H} NMR** (75 MHz, $CDCl_3$): δ = 154.9, 151.4, 141.7, 123.2, 72.5, 32.1, 22.0. **^{11}B{^{1}H} NMR** (96 MHz, $CDCl_3$): δ = 26.5. **HRMS-ASAP** (m/z): Calculated (found) for $C_{10}H_{14}BNO_2$ $[M+H]^+$ 192.1190 (192.1185).

The spectroscopic data for **3w** match those reported in the literature.[17]

5,5-Dimethyl-2-(thiophen-2-yl)-1,3,2-dioxaborinane 3x

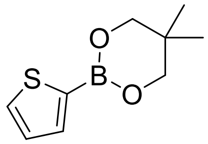

3x

Yield: 69.2 mg (355 µmol, 71%) of a colorless solid. **^{1}H NMR** (300 MHz, $CDCl_3$): δ = 7.60 – 7.57 (m, 2H), 7.17 (dd, J = 5, 4 Hz, 1H), 3.77 (s, 4H), 1.03 (s, 6H). **^{13}C{^{1}H} NMR** (75 MHz, $CDCl_3$): δ = 135.8, 131.5, 128.2, 72.5, 32.2, 22.1. **^{11}B{^{1}H} NMR** (96 MHz, $CDCl_3$): δ = 25.4. **HRMS-ASAP** (m/z): Calculated (found) for $C_9H_{14}BO_2S$ $[M+H]^+$ 197.0802 (197.0796).

The spectroscopic data for **3x** match those reported in the literature.[17]

1.5 Unsuccessful Substrates

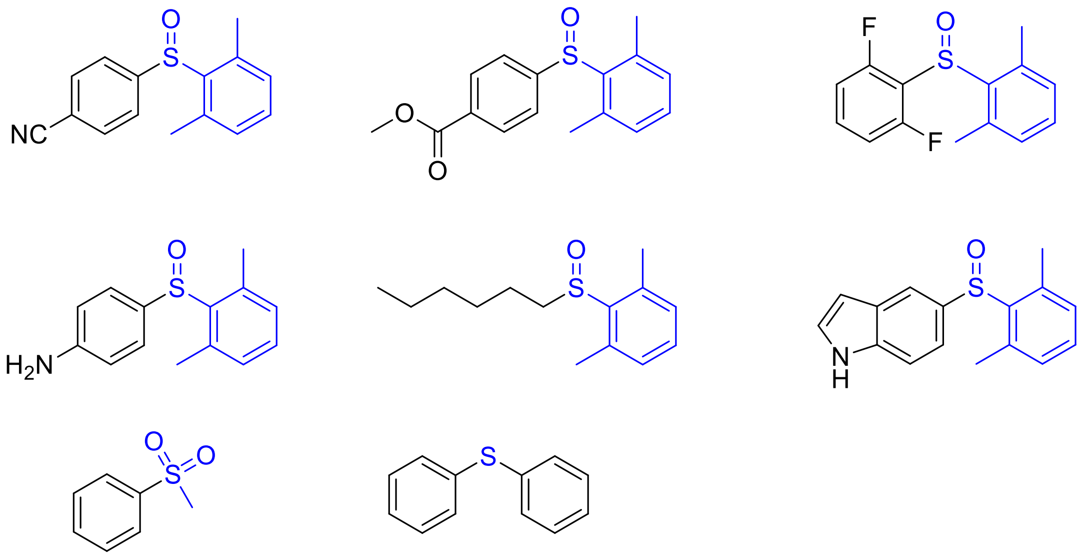

1.6 Synthesis and Characterization of *trans*-[Ni(ICy)$_2$(Ar^1){(SO)Ar^2}]

***trans*-[Ni(ICy)$_2$(4-CF$_3$-C$_6$H$_4$){(SO)-4-MeO-C$_6$H$_4$}] (4)**: In an argon-filled glovebox, 1-methoxy-4-((4-(trifluoromethyl)phenyl)sulfinyl)benzene **1y** (0.5 mmol, 1.0 equiv., 150 mg), [Ni(COD)$_2$] (0.5 mmol, 1.0 equiv., 138 mg), ICy•HBF$_4$ (1 mmol, 2.0 equiv., 320 mg), NaO^{t}Bu (1 mmol, 2.0 equiv., 96 mg) and THF (10 mL) were added to a 50 mL round-bottom flask equipped with a magnetic stirring bar. The reaction mixture was stirred overnight at room temperature. All volatiles were removed *in vacuo* and the resulting yellow residue was suspended in 25 mL of hexane. The product was collected by filtration and dried *in vacuo* to give **4** (354 mg, 86%) as a yellow powder. **Elemental analysis** for [$C_{44}H_{59}F_3N_4NiO_2S$] [823.72 g/mol]: Calc. (found) C 64.16 (63.88), H 7.22 (7.38), N 6.80 (6.83), S 3.89 (4.16). **^{1}H NMR** (400 MHz, 25 °C, C_6D_6): δ = 7.52 (d, J = 8 Hz, 2H), 7.01 (dd, J = 12 Hz, 8 Hz, 4H), 6.76 (d, J = 8 Hz, 2H), 6.35 (s, 4H), 5.98 (t, J = 12 Hz, 4H), 3.33 (s, 3H), 2.60 (d, J = 12 Hz, 4H), 1.82-1.56 (m, 24H), 1.35-1.18 (m, 8H), 1.09-1.00 (m, 4H). **^{13}C{^{1}H} NMR** (100 MHz, 25 °C, C_6D_6): δ = 182.3, 147.4, 138.7, 134.1, 121.0, 116.9, 116.6, 114.2, 113.2, 59.9, 55.0, 34.7, 34.3, 26.6, 26.4, 25.8. **^{19}F{^{1}H} NMR** (376 MHz, C_6D_6): δ = -61.2. **HRMS-LIFDI** (m/z): Calculated (found) for $C_{37}H_{52}F_3N_4Ni$ [*M*-(SO)4-OMePh]$^+$ 667.3492 (667.3488). **IR (ATR)**: 624 (w), 699 (s), 810 (s), 897 (w), 1008 (m),1037 (vw), 1061 (s) 1111 (s), 1152 (m), 1194 (w), 1235 (s), 1318 (s), 1420 (m), 1441 (w), 1478 (m), 1577 (vw), 2852 (vw), 2930 (w).

***trans*-[Ni(ICy)$_2$(C$_6$H$_5$){(SO)-C$_6$H$_5$}] (5)**: In an argon-filled glovebox, diphenyl sulfoxide **1a** (0.5 mmol, 1.0 equiv., 101 mg), [Ni(COD)$_2$] (0.5 mmol, 1.0 equiv., 138 mg), ICy•HBF$_4$ (1.0 mmol, 2.0 equiv., 320 mg), NaO^{t}Bu (1.0 mmol, 2.0 equiv., 96 mg) and THF (10 mL) were added to a 50 mL round-bottom flask equipped with a magnetic stirring bar. The reaction mixture was stirred overnight at room temperature. All volatiles were removed *in vacuo* and the resulting yellow residue was suspended in 25 mL of hexane. The product was collected by filtration and dried *in vacuo* to give **5** (282 mg, 78%) as a yellow powder. **Elemental analysis** for [$C_{42}H_{58}N_4NiOS$] [725.69 g/mol]: Calc. (found) C 69.51 (69.36), H 8.06 (8.27), N 7.72 (7.73), S 4.42 (4.26). **^{1}H NMR** (400 MHz, 25 °C, C_6D_6): δ = 7.43 (d, J = 7 Hz, 2H), 7.16-7.10 (m, 4H), 6.84 (t, J = 7 Hz, 2H), 6.75-6.72 (m, 2H), 6.39 (s, 4H), 6.11 (t, J = 11 Hz, 4H), 2.64 (d, J = 11 Hz, 4H), 1.94-1.66 (m, 24H), 1.35-1.05 (m, 12H). **^{13}C{^{1}H} NMR** (100 MHz, 25 °C, C_6D_6): δ = 183.8, 157.2, 139.2, 125.4, 120.8, 119.9, 118.5, 116.3, 59.7, 34.8, 34.2, 26.6, 26.5, 25.9. **HRMS-LIFDI** (m/z): Calculated (found) for $C_{42}H_{57}N_4NiSO$ [M]$^+$ 723.3601 (723.3594). **IR (ATR)**: 509 (w), 566 (vw), 692 (m), 703 (s), 733 (s), 821 (m), 836 (m), 896 (w), 986 (vw), 1018 (w), 1083 (vw), 1196 (w), 1235 (m), 1266 (vw), 1383 (vw), 1424 (m), 1446 (w), 1464 (m), 1560 (w), 1577 (m), 2852 (w), 2923 (m).

***trans*-[Ni(ICy)$_2$(4-CH$_3$-C$_6$H$_4$){(SO)-4-CH$_3$-C$_6$H$_4$}] (6)**: In an argon-filled glovebox, bis(4-methylphenyl) sulfoxide **1b** (0.5 mmol, 1.0 equiv., 115 mg), [Ni(COD)$_2$] (0.5 mmol, 1.0 equiv., 138 mg), ICy•HBF$_4$ (1.0 mmol, 2.0 equiv., 320 mg), NaO^{t}Bu (1.0 mmol, 2.0 equiv., 96 mg) and THF (10 mL) were added to

a 50 mL round-bottom flask equipped with a magnetic stirring bar. The reaction mixture was stirred overnight at room temperature. All volatiles were removed *in vacuo* and the resulting yellow residue was suspended in 25 mL of hexane. The product was collected by filtration and dried *in vacuo* to give **6** (275 mg, 73%) as a yellow powder. **Elemental analysis** for [$C_{44}H_{62}N_4NiOS$] [753.75 g/mol]: Calc. (found) C 70.11 (70.00), H 8.29 (8.42), N 7.43 (7.39), S 4.25 (4.52). **^{1}H NMR** (400 MHz, 25 °C, C_6D_6): δ = 7.35 (d, J = 8 Hz, 2H), 7.07 (d, J = 8 Hz, 2H), 6.93 (d, J = 8 Hz, 2H), 6.69 (d, J = 8 Hz, 2H), 6.39 (s, 4H), 6.15 (t, J = 12 Hz, 4H), 2.67 (d, J = 12 Hz, 4H), 2.14 (s, 3H), 2.07 (s, 3H), 1.99 (d, J = 12 Hz, 4H), 1.81-1.68 (m, 20H), 1.36-1.25 (m, 8H), 1.36-1.25 (m, 8H), 1.13-1.05 (m, 4H). **^{13}C{^{1}H} NMR** (100 MHz, 25 °C, C_6D_6): δ = 184.1, 153.8, 138.6, 129.1, 128.6, 126.5, 119.1, 116.3, 59.7, 34.8, 34.3, 26.63, 26.56, 26.0, 21.0, 20.9. **HRMS-LIFDI** (m/z): Calculated (found) for $C_{37}H_{55}N_4Ni$ [*M*-(SO)4-MePh]$^+$ 613.3775 (613.3768). **IR (ATR)**: 475 (m), 501 (m), 562 (vw), 624 (w), 702 (s), 727 (vw), 756 (vw), 793 (s), 826 (s), 897 (m), 995 (vw), 1037 (vw), 1078 (m), 1144 (vw), 1193 (m), 1235 (m), 1264 (vw), 1292 (vw), 1383 (w), 1424 (m), 1445 (m), 1482 (m), 1548 (vw), 1594 (vw), 2852 (m), 2926 (m).

***trans*-[Ni(ICy)$_2$(4-F-C_6H_4){(SO)-4-F-C_6H_4}] (7)**: In an argon-filled glovebox, bis(4-fluorophenyl) sulfoxide **1e** (0.5 mmol, 1.0 equiv., 119 mg), [Ni(COD)$_2$] (0.5 mmol, 1.0 equiv., 138 mg), ICy•HBF$_4$ (1 mmol, 2.0 equiv., 320 mg), NaO^{t}Bu (1 mmol, 2.0 equiv., 96 mg) and THF (10 mL) were added to a 50 mL round-bottom flask equipped with a magnetic stirring bar. The reaction mixture was stirred overnight at room temperature. All volatiles were removed *in vacuo* and the resulting yellow residue was suspended in 25 mL of hexane. The product was collected by filtration and dried *in vacuo* to give **7** (239 mg, 63%) as a yellow powder. **Elemental analysis** for [$C_{42}H_{56}F_2N_4NiOS$] [761.68 g/mol]: Calc. (found) C 66.23 (66.51), H 7.41 (7.67), N 7.36 (7.49), S 4.21 (4.17). **^{1}H NMR** (400 MHz, 25 °C, C_6D_6): δ = 7.20 (t, J = 9 Hz, 2H), 6.92-6.88 (m, 2H), 6.80 (t, J = 9 Hz, 2H), 6.63 (t, J = 9 Hz, 2H), 6.36 (s, 4H), 5.99 (t, J = 12 Hz, 4H), 2.56 (d, J = 12 Hz, 4H), 1.86-1.55 (m, 24H), 1.35-1.20 (m, 8H), 1.09-0.99 (m, 4H). **^{13}C{^{1}H} NMR** (100 MHz, 25 °C, C_6D_6): δ = 183.1, 161.2, 159.3, 151.8, 138.8 (d, J = 5 Hz), 116.5, 114.7 (d, J = 21 Hz), 112.3 (d, J = 18 Hz), 59.8, 34.8, 34.2, 26.54, 26.47, 25.8. **^{19}F{^{1}H} NMR** (376 MHz, C_6D_6): δ = -125.0, -125.4. **HRMS-LIFDI** (m/z): Calculated (found) for $C_{36}H_{52}FN_4Ni$ [M-(SO)-4-FPh]$^+$ 617.3524 (617.3518). **IR (ATR)**: 414 (w), 488 (m), 509 (w), 567 (w), 620 (m), 702 (s), 733 (s), 723 (vw), 756 (vw), 810 (s), 892 (m), 983 (vw), 1000 (vw), 1025 (vw), 1082 (w), 1144 (w), 1201 (s), 1235 (m), 1379 (vw), 1424 (m), 1445 (w), 1474 (s), 1565 (vw), 2852 (m), 2926 (m).

1.7 Investigations Concerning the Reaction Mechanism

a) $B_2(neop)_2$ Itself is Stable Under These Conditions (1 h at 110 °C in C_6D_6, see Figure S1).

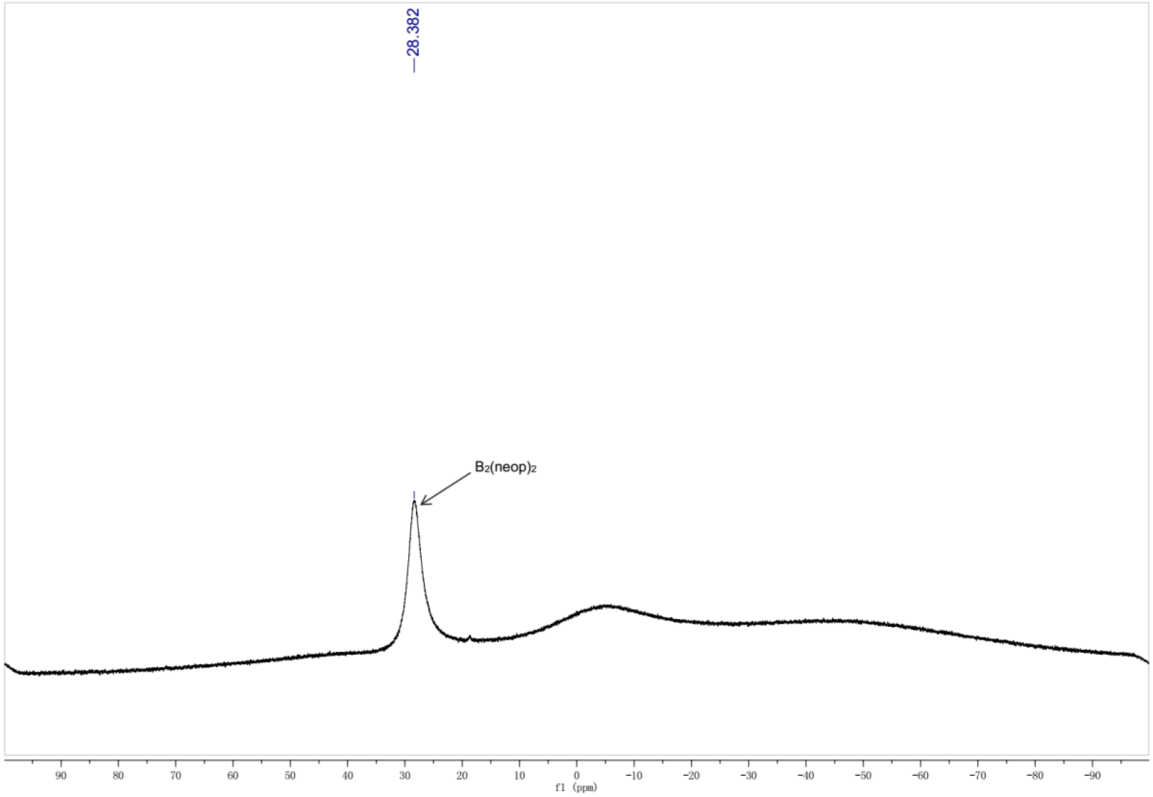

Figure S1. ^{11}B NMR spectrum after heating the starting material $B_2(neop)_2$ to 110 °C for 1 h without forming any decomposition product.

b) Reduction of Sulfoxide to Sulfide

In a Young's tap NMR tube, diphenyl sulfoxide (0.05 mmol, 10.1 mg), $B_2(neop)_2$ (0.10 mmol, 22.6 mg) and [Ni(COD)$_2$] (1.4 mg, 0.005 mmol) were added to C_6D_6 (0.7 mL). After measurement of $^{11}B\{^1H\}$ NMR at room temperature, the resulting solution was heated at 110 °C for 2 h, and $^{11}B\{^1H\}$ NMR of the mixture was measured again.

The signal corresponding to $B_2(neop)_2$ (δ = 28.3) was observed before heating.

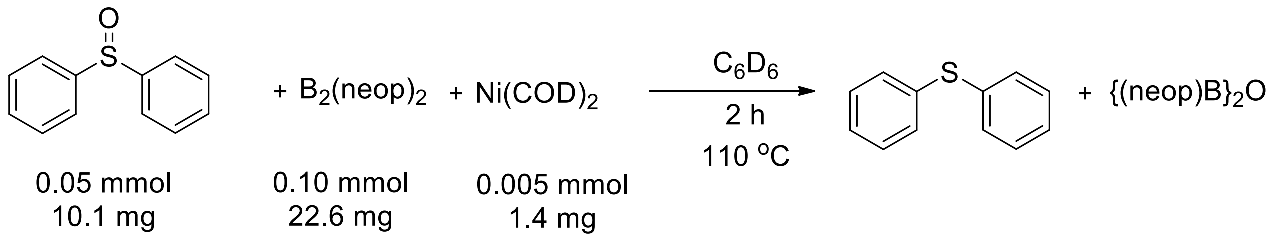

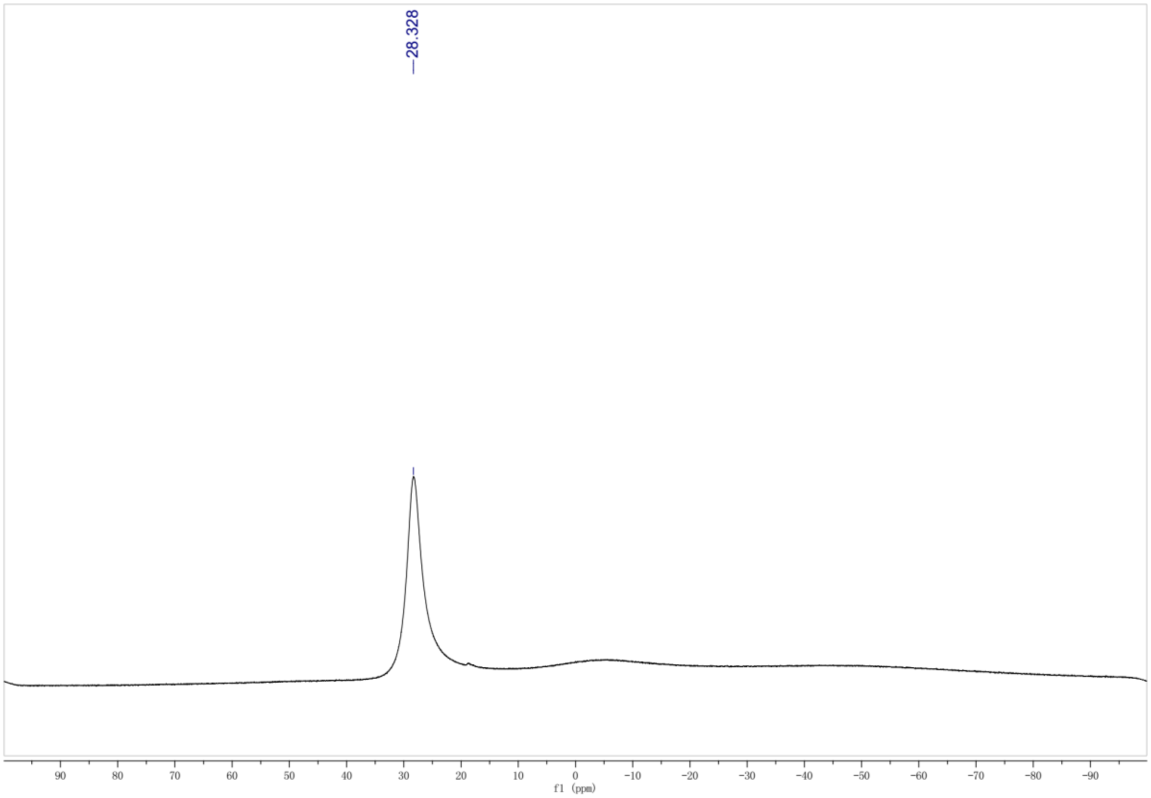

Figure S2. ^{11}B NMR spectrum of diphenyl sulfoxide, $B_2(neop)_2$ and [Ni(COD)$_2$] in C_6D_6 at room temperature.

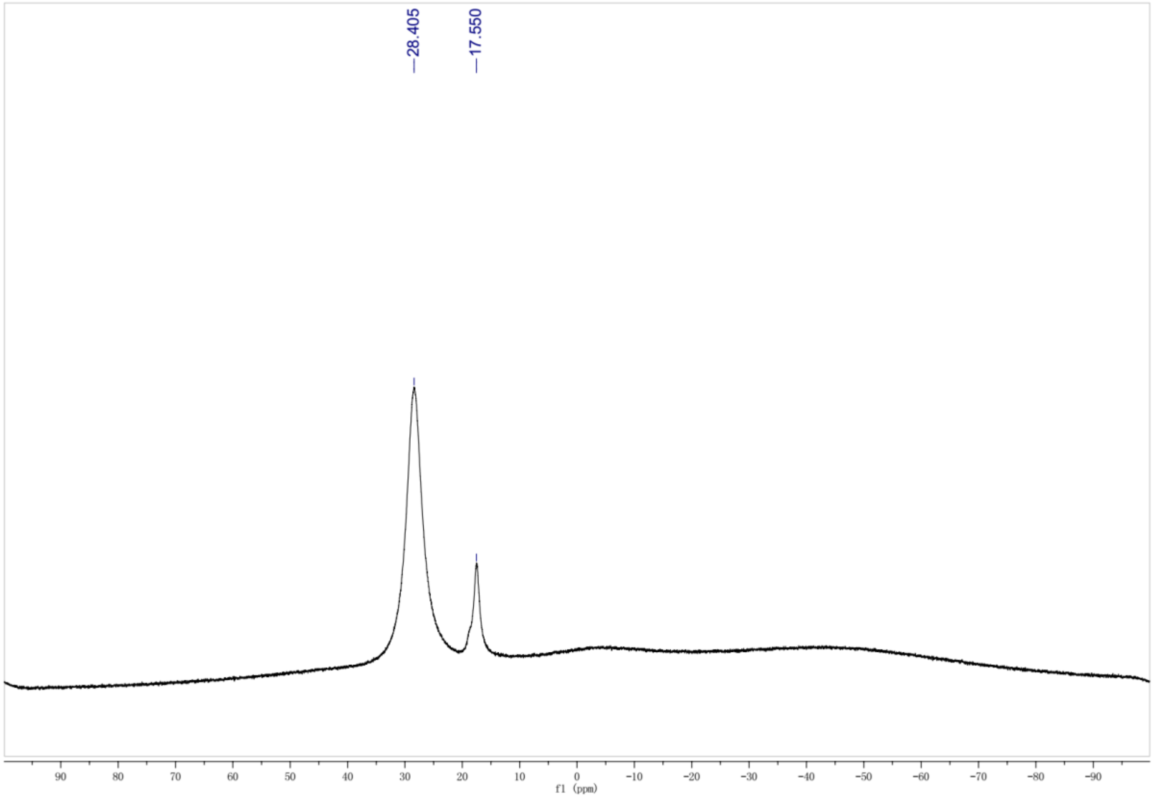

Figure S3. ^{11}B NMR spectrum of diphenyl sulfoxide, $B_2(neop)_2$ and $[Ni(COD)_2]$ in C_6D_6 at 110 °C for 2 h. After heating, a new sharp signal attributed to $\{(neop)B\}_2O$ (δ = 17.6) emerged.

c) Reactivity Tests Involving Diaryl Sulfides

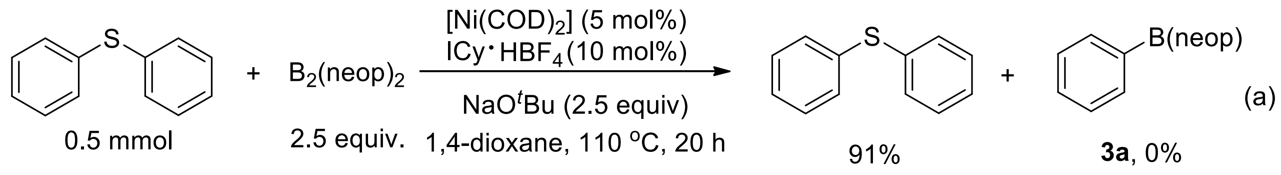

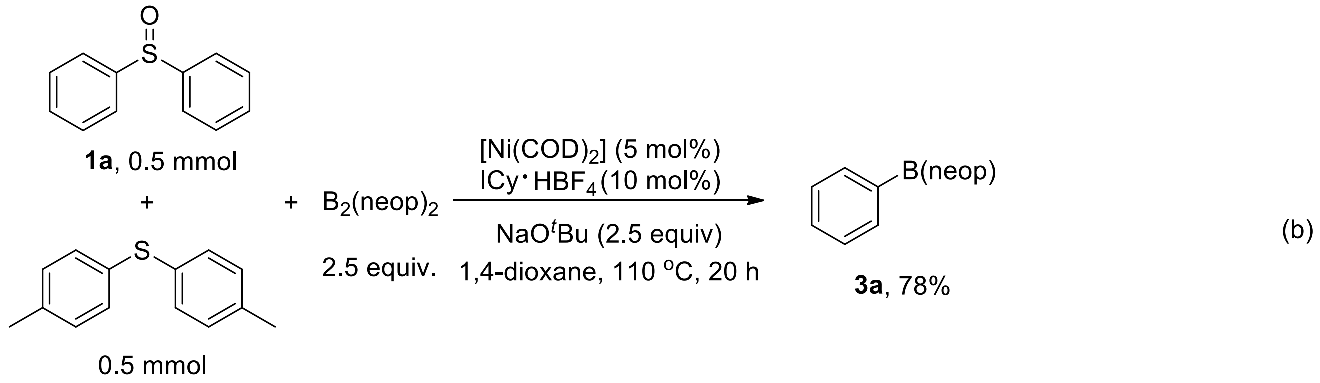

In an argon-filled glovebox, [Ni(COD)$_2$] (5 mol%), ICy•HBF$_4$ (10 mol%) and 1,4-dioxane (3 mL) were added to a 10 mL thick-walled reaction tube equipped with a magnetic stirring bar. NaO^{t}Bu (1.25 mmol, 2.5 equiv.), B$_2$(neop)$_2$ (1.25 mmol, 2.5 equiv.) and [(a): diphenylsulfide (0.5 mmol); (b): di-*p*-tolylsulfide (0.5 mmol), **1a** (0.5 mmol)] were added. The reaction mixture was stirred at 110 °C for 20 h, then diluted with Et$_2$O (2 mL) and filtered through a pad of Celite (Ø 3 mm x 8 mm). The product was isolated by flash column chromatography (hexane/ethyl acetate (95/5)) after careful removal of the solvent *in vacuo*.

d) Reactivity Tests of *trans*-[Ni(ICy)$_2$(4-CF$_3$-C$_6$H$_4$){(SO)-4-MeO-C$_6$H$_4$}] 4 with B$_2$(neop)$_2$.

We studied the reactivity of [Ni^{II}] with stoichiometric amounts of B$_2$(neop)$_2$. In a Young's tap NMR tube *trans*-[Ni(ICy)$_2$(4-CF$_3$-C$_6$H$_4$){(SO)-4-MeO-C$_6$H$_4$}] **4** (24.7 mg, 30.0 µmol, 1.0 equiv.) and B$_2$(neop)$_2$ (6.8 mg, 30 µmol, 1.0 equiv.) were dissolved in C$_6$D$_6$ (0.7 mL). Investigating the reaction mixture by NMR spectroscopy after 1 h at room temperature did not show any borylated product. Heating the reaction mixture to 110 °C for 1 h revealed small amounts of **3e** in the ^{19}F and ^{11}B NMR spectra.

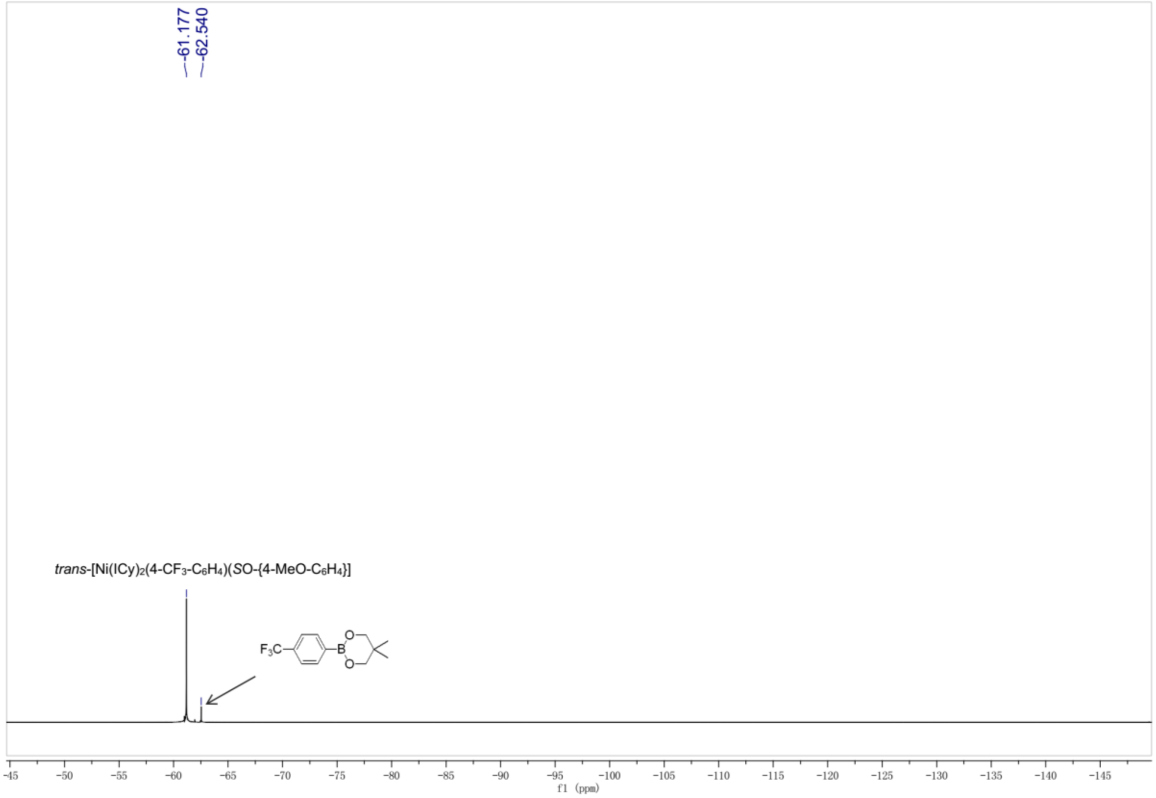

Figure S4. ^{19}F NMR spectrum (recorded in C$_6$D$_6$; 470 MHz) of the reaction mixture of *trans*-[Ni(ICy)$_2$(4-CF$_3$-C$_6$H$_4$){(SO)-4-MeO-C$_6$H$_4$}] **4** and B$_2$(neop)$_2$ (110 °C, 1h).

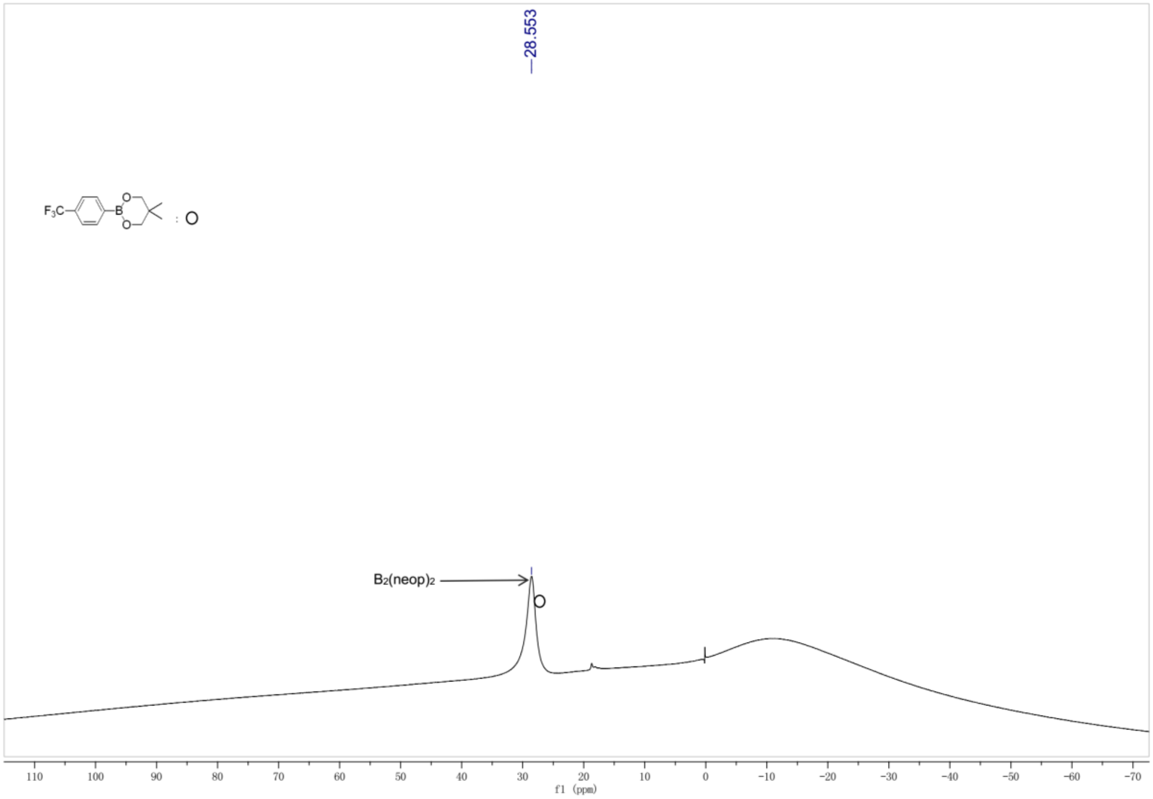

Figure S5. ^{11}B NMR spectrum (recorded in C_6D_6; 160 MHz) of the reaction mixture of *trans*-[Ni(ICy)$_2$(4-CF_3-C_6H_4){(SO)-4-MeO-C_6H_4}] **4** and B_2(neop)$_2$ (110 °C, 1h).

e) Catalytic Test Reaction Using [Ni(COD)$_2$]

A mixture of 1-methoxy-4-((4-(trifluoromethyl)phenyl)sulfinyl)benzene **1y** (0.025 mmol, 7.5 mg), B_2(neop)$_2$ (0.05 mmol, 11.3 mg, 2 eq.), NaO^{t}Bu (0.05 mmol, 4.8 mg, 2.0 eq.), [Ni(COD)$_2$] (0.0025 mmol, 0.7 mg, 10 mol%) and ICy•HBF$_4$ (0.005 mmol, 1.6 mg, 20 mol%) was suspended in 0.7 mL C_6D_6 in a Young's tap NMR tube. Then the mixture was studied by NMR spectroscopy after 1 h at room temperature, which revealed only the formation of *trans*-[Ni(ICy)$_2$(4-CF$_3$-C$_6$H$_4$){(SO)-4-MeO-C$_6$H$_4$}] **4**; however, Heating the reaction mixture to 110 °C for 2 h, it showed significant borylation product and the presence of **4**.

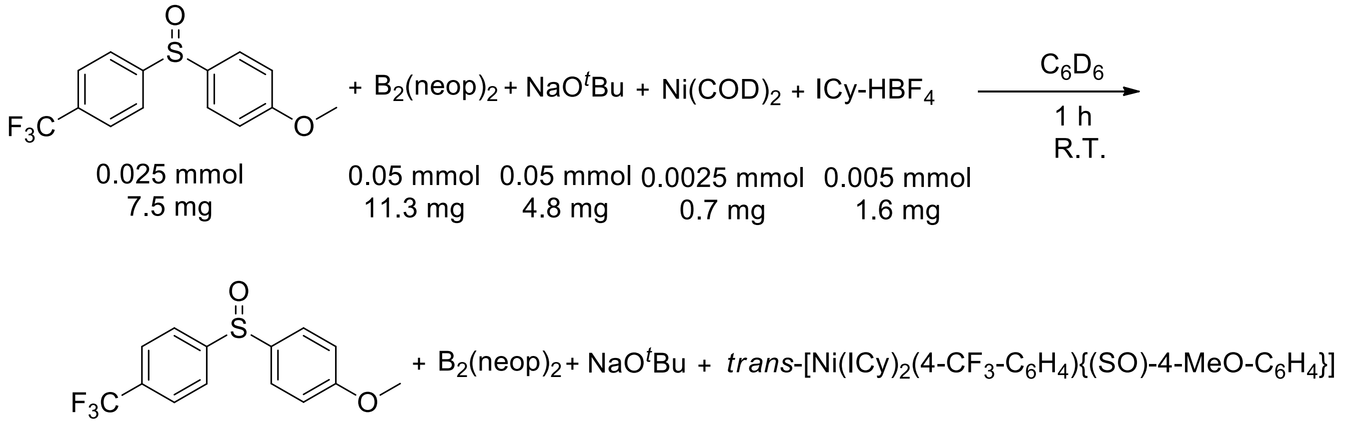

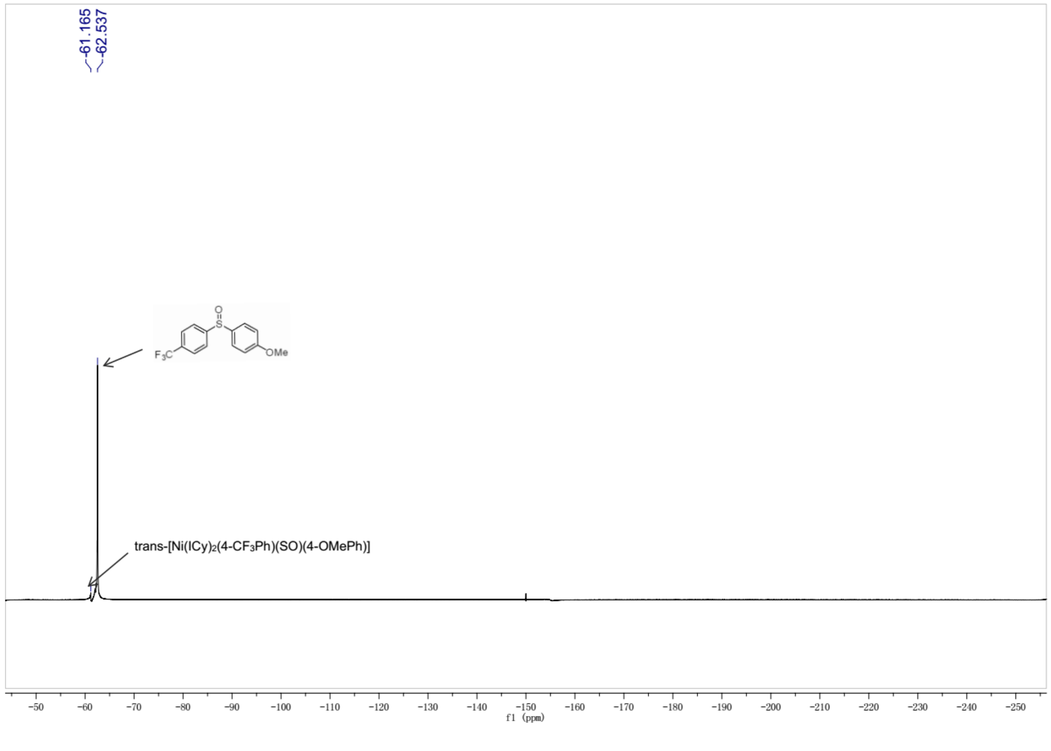

Figure S6. ^{19}F NMR spectrum (recorded in C_6D_6; 470 MHz) after 1 h at room temperature.

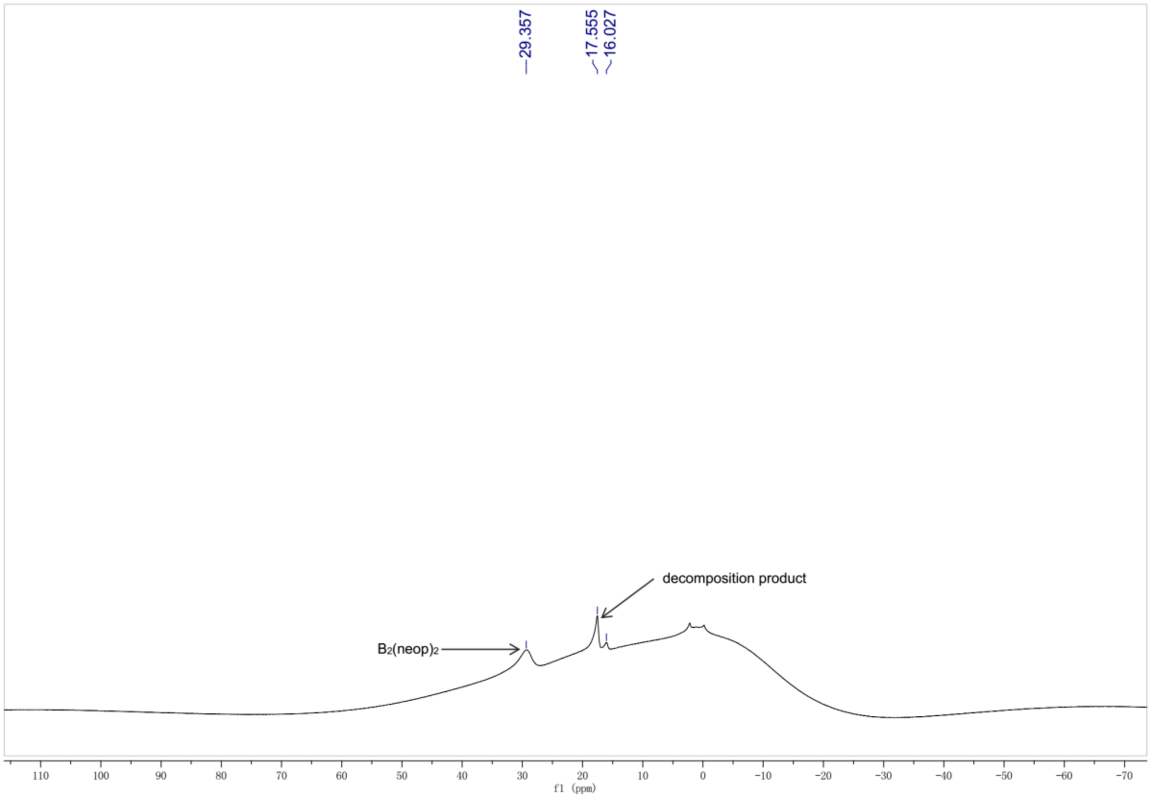

Figure S7. ^{11}B NMR spectrum (recorded in C_6D_6; 160 MHz) after 1 h at room temperature.

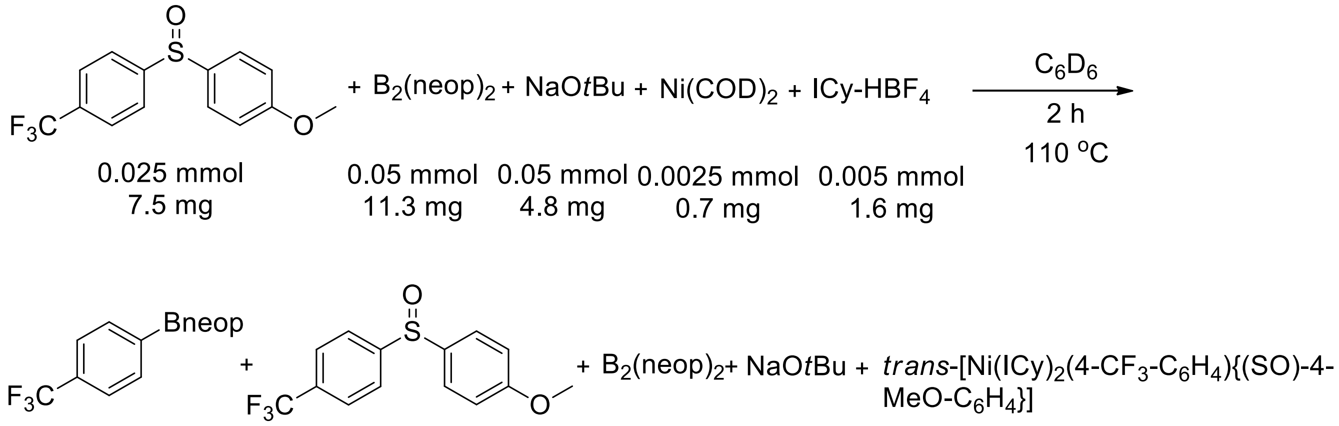

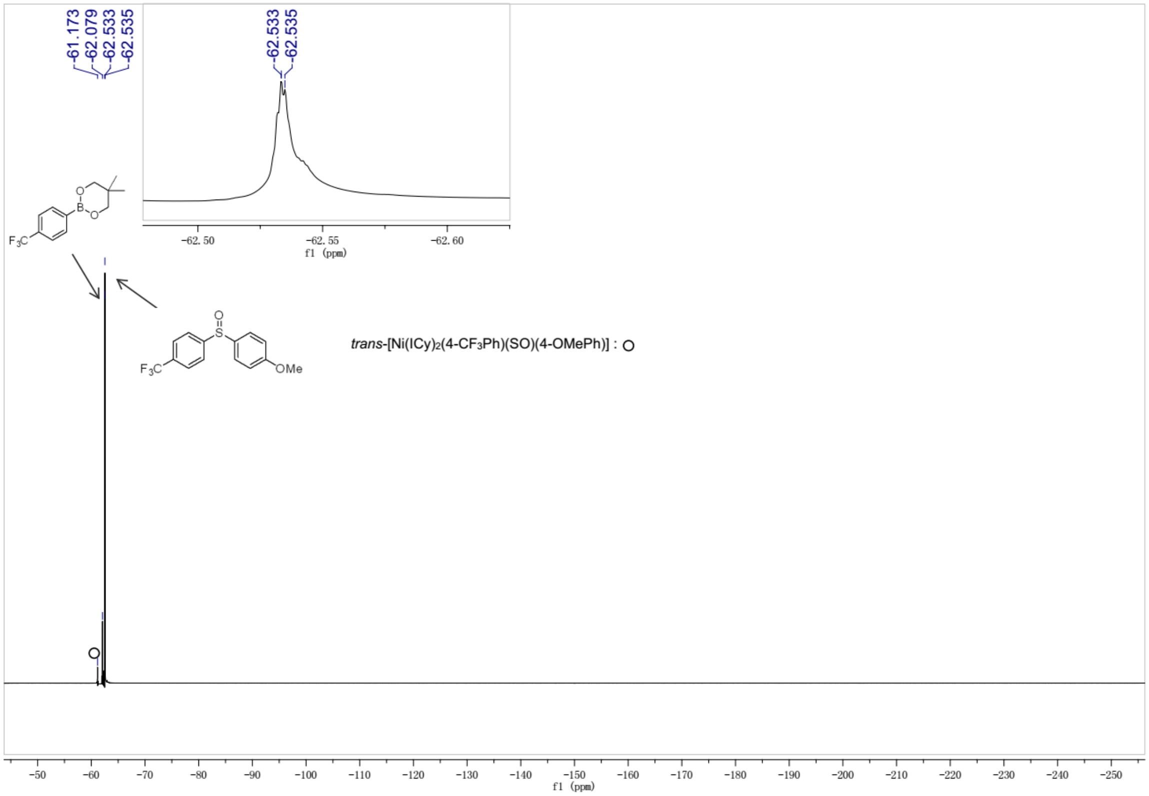

Figure S8. ^{19}F NMR spectrum (recorded in C_6D_6; 470 MHz) at 110 ^{o}C for 2 h.

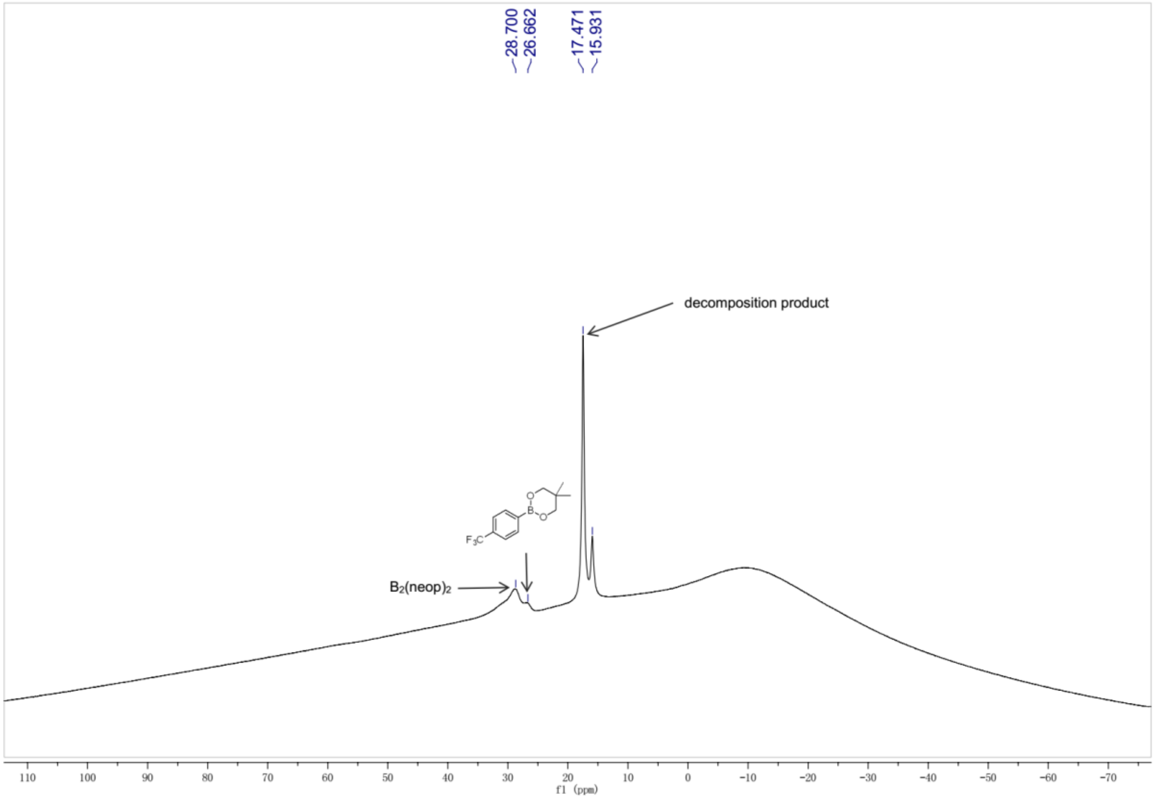

Figure S9. ^{11}B NMR spectrum (recorded in C_6D_6; 160 MHz) at 110 ^{o}C for 2 h.

f) Stoichiometric Reaction of [Ni(COD)$_2$], ICy with B_2(neop)$_2$

In a Young's tap NMR tube, B_2(neop)$_2$ (6.7 mg, 30.0 µmol, 1.0 equiv.), ICy (13.9 mg, 60.0 µmol, 2.0 equiv.) and [Ni(COD)$_2$] (8.3 mg, 30.0 µmol, 1.0 equiv.) were added to 1,4-dioxane (0.7 mL). Investigation of the reaction mixture by $^{11}B\{^1H\}$ NMR spectroscopy revealed no nickel-boryl complex.

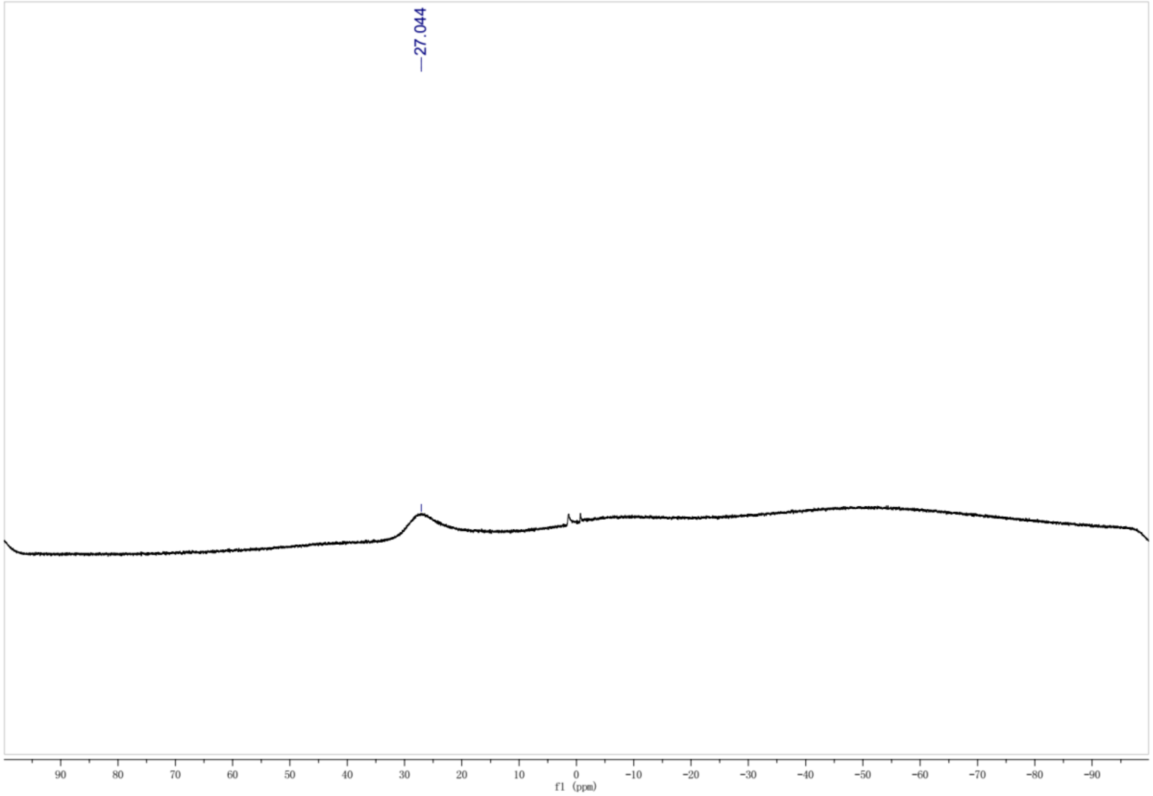

Figure S10. ^{11}B NMR spectrum (recorded in 1,4-dioxane; 96 MHz) at room temperature.

g) Reactivity of *trans*-[Ni(ICy)$_2$(C$_6$H$_5$){(SO)-C$_6$H$_5$}]

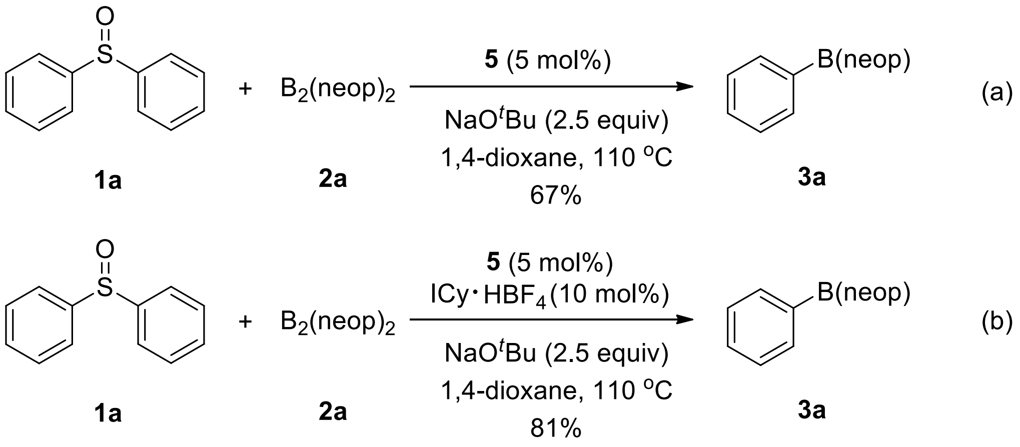

In an argon-filled glovebox, *trans*-[Ni(ICy)$_2$(C$_6$H$_5$){(SO)-C$_6$H$_5$}] **5** (5 mol%), [(a): no ICy•HBF$_4$; (b): 10 mol% ICy•HBF$_4$] and 1,4-dioxane (3 mL) were added to a 10 mL thick-walled reaction tube equipped with a magnetic stirring bar. NaO^{t}Bu (1.25 mmol, 2.5 equiv.), B$_2$(neop)$_2$ (1.25 mmol, 2.5 equiv.) and the aryl sulfoxide (0.5 mmol, 1.0 equiv.) were added. The reaction mixture was stirred at 110 °C for 20 h, then diluted with Et$_2$O (2 mL) and filtered through a pad of Celite (Ø 3 mm x 8 mm). The product was isolated by flash column chromatography (hexane/ethyl acetate (95/5)) after careful removal of the solvent *in vacuo* (especially noting that volatile arylboronates can evaporate with the solvent)

1.8 VT-NMR spectra of 5 in THF-d_8

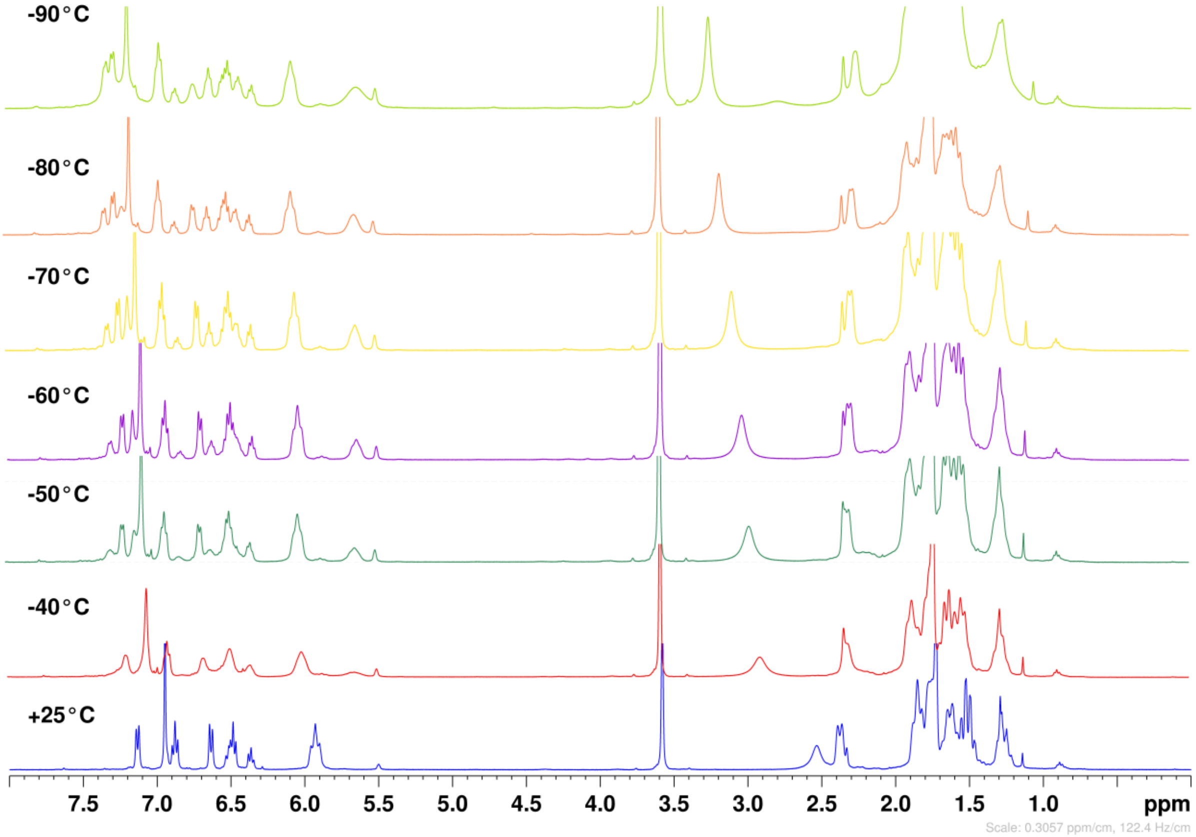

Figure S11. ^{1}H VT-NMR spectra of **5** in THF-d_8.

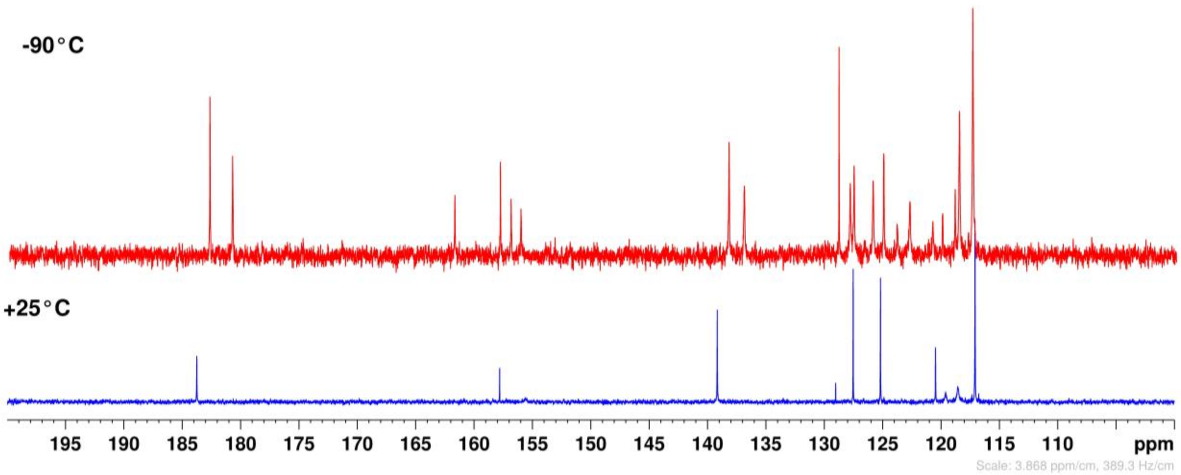

Figure S12. $^{13}C\{^{1}H\}$ VT-NMR spectra of **5** in THF-d_8.

2 Crystallographic Details

Crystals were immersed in a film of perfluoropolyether oil, mounted on MiTeGen sample holders, and transferred to a Bruker X8 Apex-2 diffractometer, with CCD area detector and mirror-monochromated Mo-K_α radiation. Data were collected at 100 K, using an Oxford Cryosystems low-temperature device. The images were processed and corrected for Lorentz-polarization effects and absorption as implemented in the Bruker software packages. The structures were solved using the intrinsic phasing method (SHELXT)[22] and Fourier expansion technique. All non-hydrogen atoms were refined in anisotropic approximation, with hydrogen atoms 'riding' in idealized positions, by full-matrix least squares against F^2 of all data, using SHELXL[23] software and the SHELXLE graphical user interface.[24] Diamond[25] software was used for graphical representation. Crystal data and experimental details are listed in Table S5; full structural information has been deposited with Cambridge Crystallographic Data Centre. CCDC-1997417 (**5-I**), 1997418 (**5**) and 2022071 (**4**).

Table S5: Crystal data and structure refinements for **5**, **5-I** and **4**.

Compound	**5**	**5-I**	**4**
CCDC number	1997418	1997417	2022071
Empirical formula	$C_{42}H_{58}N_4NiOS{\cdot}2(CH_2Cl_2)$	$C_{42}H_{58}N_4NiOS$	$C_{40}H_{59}F_3N_4NiO_2S{\cdot}C_4H_8O_5$
Formula weight (g·mol^{-1})	895.54	725.69	895.82
Temperature (K)	100(2)	100(2)	100(2)
Radiation, λ (Å)	Mo-K_α 0.71073	Mo-K_α 0.71073	Mo-K_α 0.71073
Crystal size (mm³)	0.20×0.24×0.29	0.08×0.16×0.33	0.43 × 0.39 × 0.19
Crystal color, habit	Yellow block	Yellow block	Yellow block
Crystal system	Monoclinic	Monoclinic	Triclinic
Space group	$P2_1/c$	$P2_1/c$	$P\bar{1}$
a (Å)	11.488(4)	12.990(4)	9.519(3)
b (Å)	30.107(8)	20.882(7)	12.442(6)
c (Å)	13.646(5)	15.209(5)	20.272(7)
α (°)	90	90	79.01(3)
β (°)	106.006(17)	106.228(8)	86.851(12)
γ (°)	90	90	74.603(10)
Volume (Å^3)	4537(2)	3961(2)	2272.2(16)
Z	4	4	2
ρ_{calc} (g·cm^{-3})	1.311	1.217	1.309
μ (mm^{-1})	0.747	0.579	0.530
F(000)	1896	1560	956.0
θ range (°)	1.353 - 26.738	1.702 - 26.360	3.454 - 55.694
Reflections collected	66732	67257	59217
Unique reflections	9640	8083	10784
Parameters / restraints	496 / 0	564 / 389	542 / 0
GooF on F^2	1.019	1.052	1.042
R_1 [I>2σ(I)]	0.0418	0.0711	0.0419
wR^2 (all data)	0.1031	0.1703	0.1140
Max./min. residual electron density (e·Å^{-3})	0.551 / –0.526	1.955 / –0.815	1.88 / –0.70

Table S6: Bond lengths (Å) and angles (°) of **5** and **5-I**.

	**5**	**5-I**	**4**
Ni – C (NHC)	1.900(2)	1.895(4)	1.8940(17)
Ni – C (NHC)	1.894(2)	1.901(4)	1.9066(17)
Ni – C (phenyl)	1.929(2)	1.930(4)	1.9254(18)
Ni – S (**5**) / O (**5-I**)	2.2284(8)	1.924(7) / 1.91(2)	2.2259(8)
C – Ni – C	177.73(9)	178.66(18)	176.16(7)
	89.93(9)	90.02(17)	88.31(7)
	88.08(9)	88.79(18)	88.59(7)
C – Ni – S (**4**, **5**) / O (**5-I**)	170.33(7)	173.5(2) / 175.8(7)	169.45(5)
	90.87(7)	94.0(5) / 89.5(16)	91.09(6)
	91.27(7)	87.3(5) / 91.6(16)	92.37(6)
Sum ∠ C – Ni – C / O / S	360.15(10)	360.1(4) / 359.9(17)	360.36(7)

3 Computational Details

DFT calculations were carried out with the Gaussian09 package.[26] The geometries of the different structures were optimized at the DFT level using the B3LYP[27] hybrid functional with the def2-SVP basis set.[28] Frequency analysis was carried out at the same level to verify the stationary points as an intermediate or transition state and to obtain the thermodynamic energy corrections assuming a standard state of 1 atm and 298.15 K. Solvent effects were taken into consideration by single point calculations of the gas-phase stationary points with the SMD[29] continuum solvation model with 1,4-dioxane as the solvent. To obtain more accurate energy information, solvation single-point energy calculations were performed at the M06[30] level of theory using the larger def2-TZVP basis set.[28] All of the three-dimensional molecular diagrams of the molecules were generated with CYLView.[31]

Table S7: Absolute calculated electronic energies, correction of enthalpies, and free energies.

Geometry	$E_{(elec\text{-}B3LYP)}$[a]	$G_{(corr\text{-}B3LYP)}$[b]	$H_{(corr\text{-}B3LYP)}$[c]	$E_{(M06\ 1,4\text{-}dioxane)}$[d]	IF[e]
**5-I**	-3834.557506	0.84834	0.982195	-3835.429856	
**TS-iso**	-3834.527749	0.850293	0.980803	-3835.415038	-190.90
**5**	-3834.537444	0.849771	0.982028	-3835.431747	

[a] Electronic energy calculated at the B3LYP/def2-SVP level in the gas phase. [b] Thermal correction to Gibbs free energy calculated at the B3LYP/def2-SVP level in the gas phase. [c] Thermal correction to enthalpy calculated at the B3LYP/def2-SVP level in the gas phase [d] Electronic energy calculated at the M06/def2-TZVP level in 1,4-dioxane. [e] B3LYP calculated imaginary frequencies for the transition state.

4 NMR Spectra

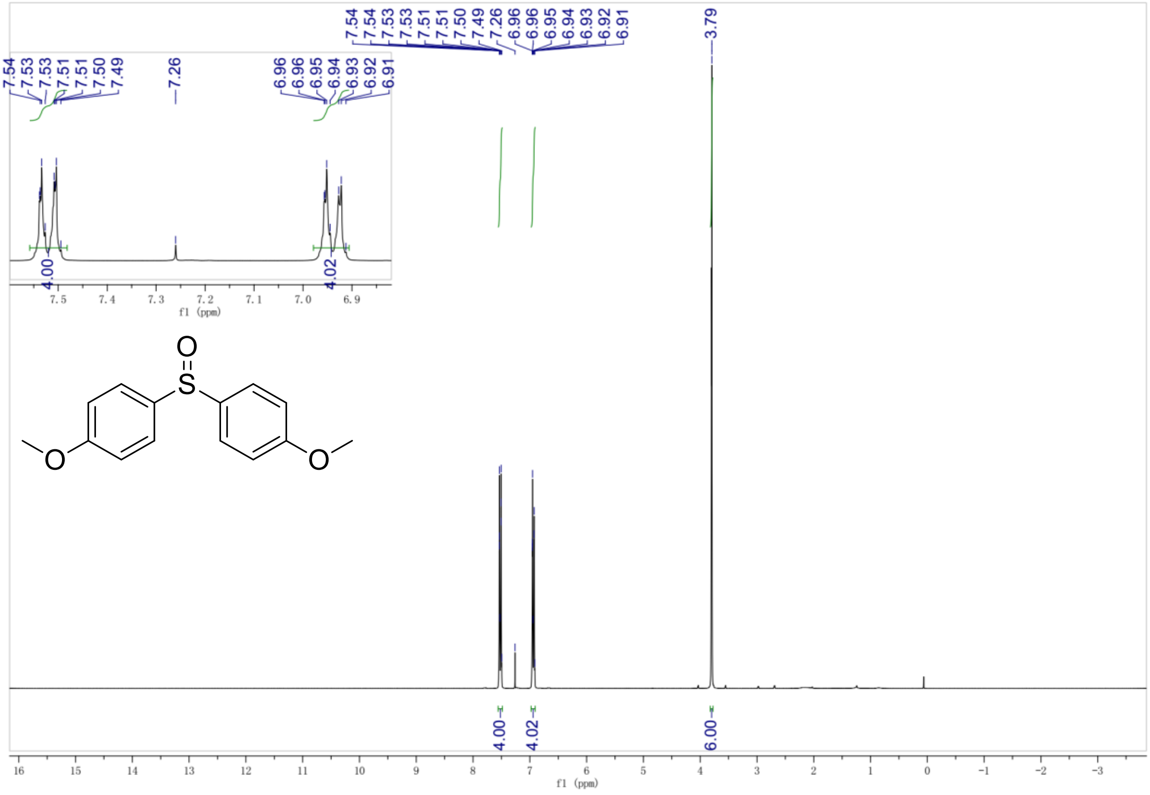

^{1}H NMR spectrum of compound **1c** in $CDCl_3$ (300 MHz).

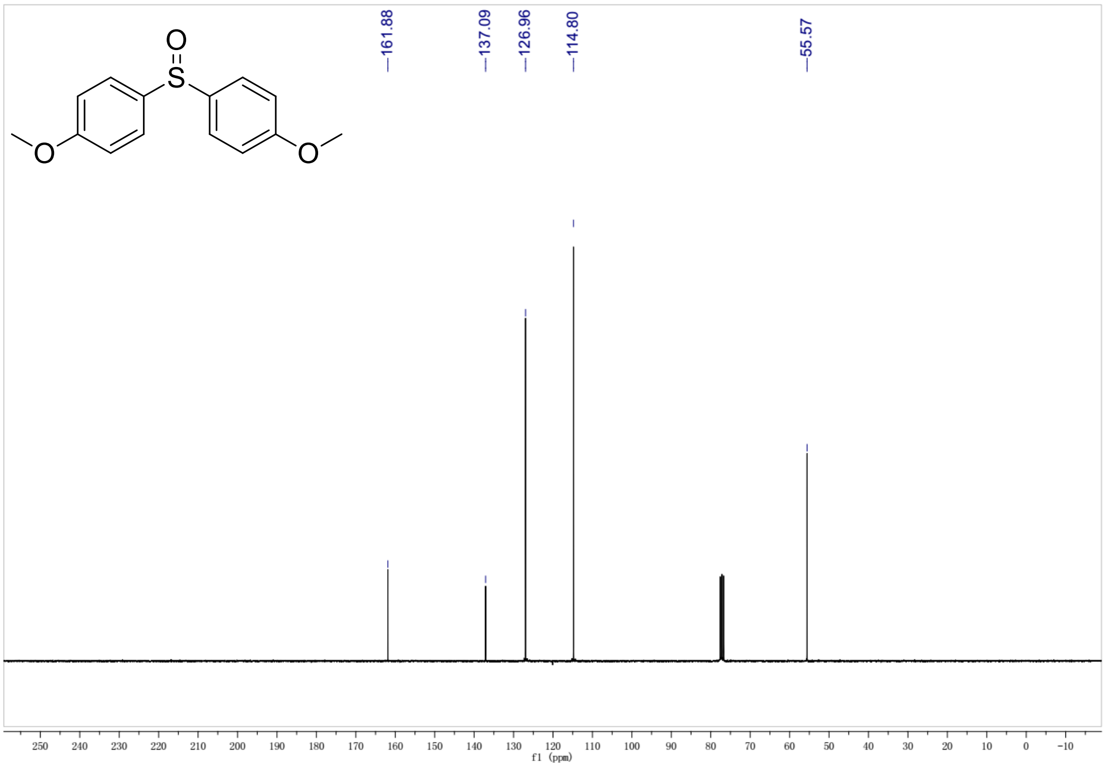

^{13}C{^{1}H} NMR spectrum of compound **1c** in $CDCl_3$ (75 MHz).

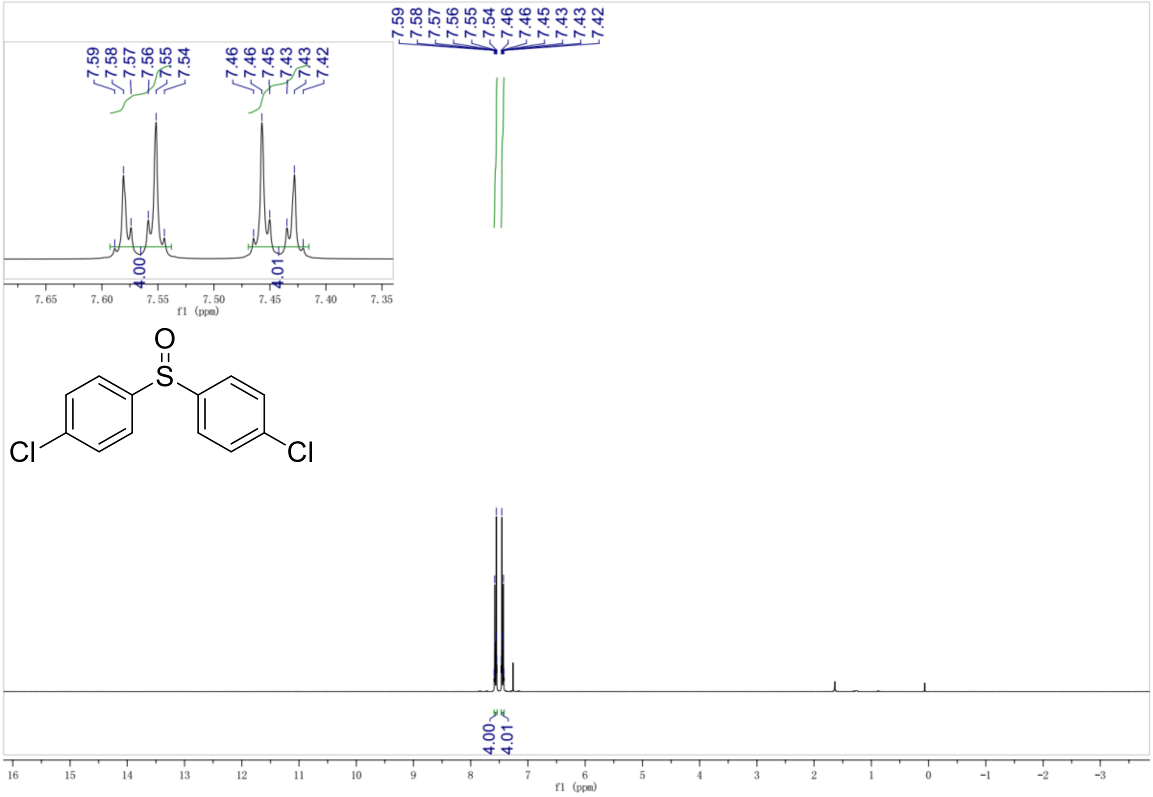

^{1}H NMR spectrum of compound **1d** in $CDCl_3$ (300 MHz).

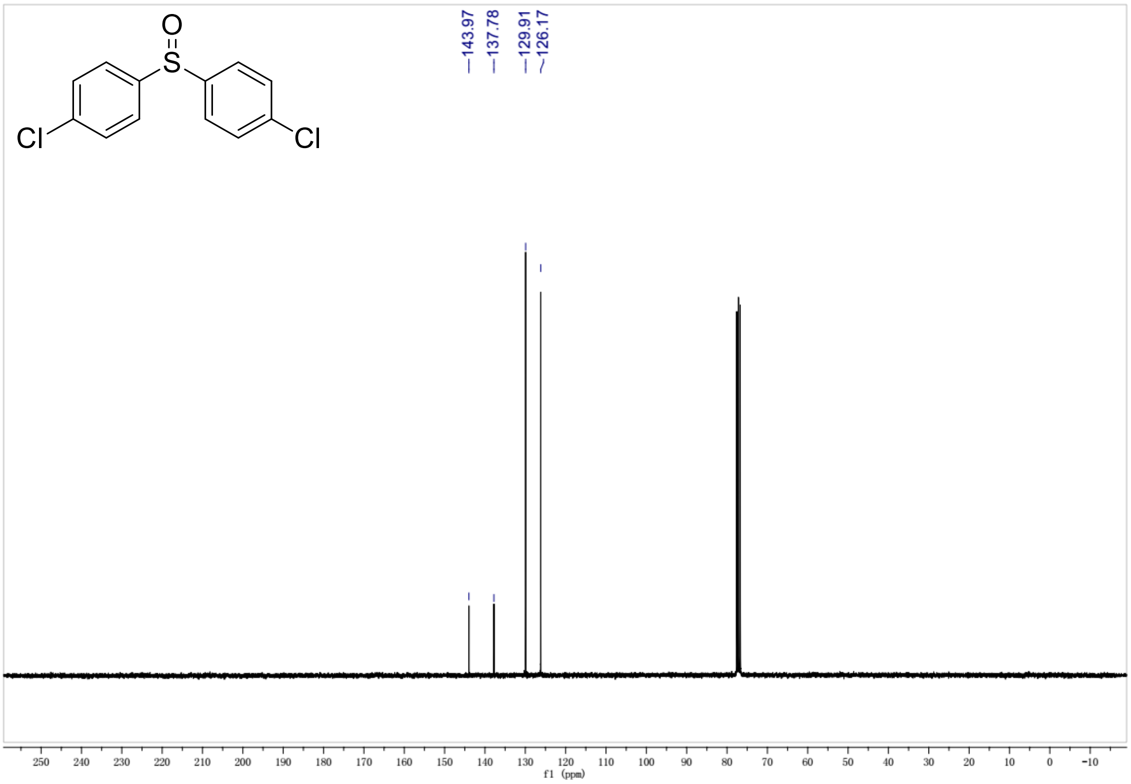

^{13}C{^{1}H} NMR spectrum of compound **1d** in $CDCl_3$ (75 MHz).

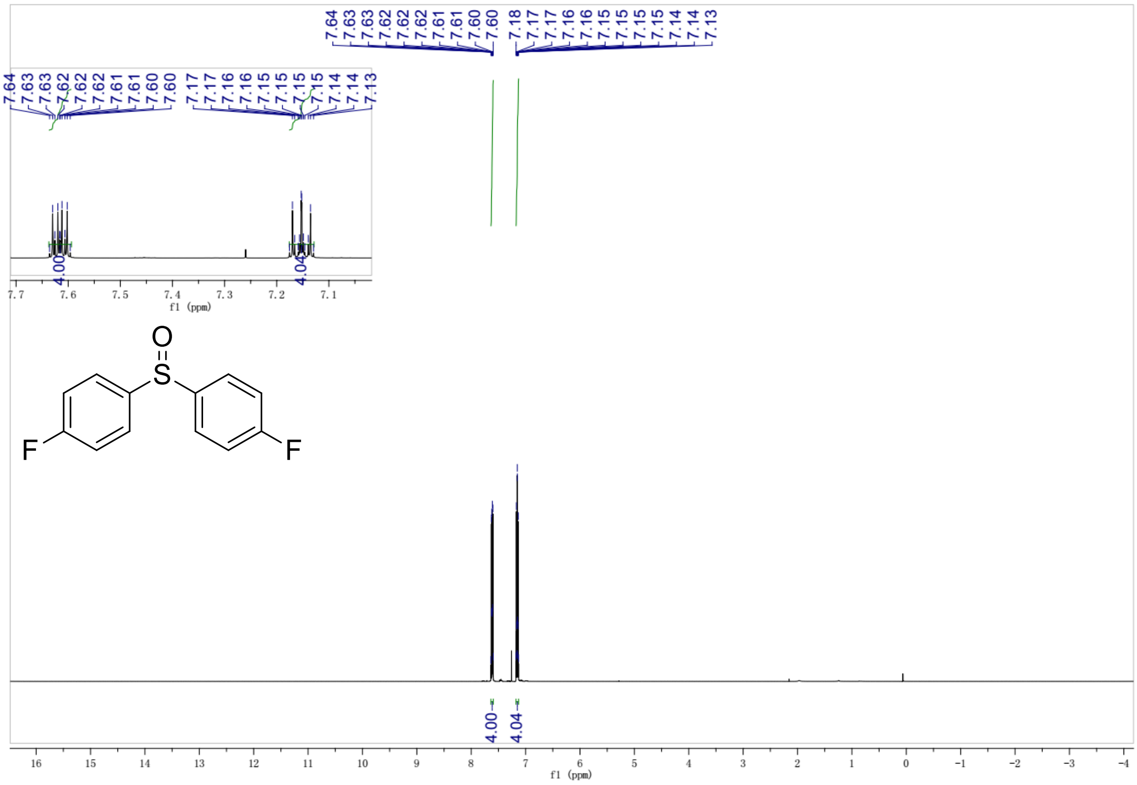

^{1}H NMR spectrum of compound **1e** in $CDCl_3$ (500 MHz).

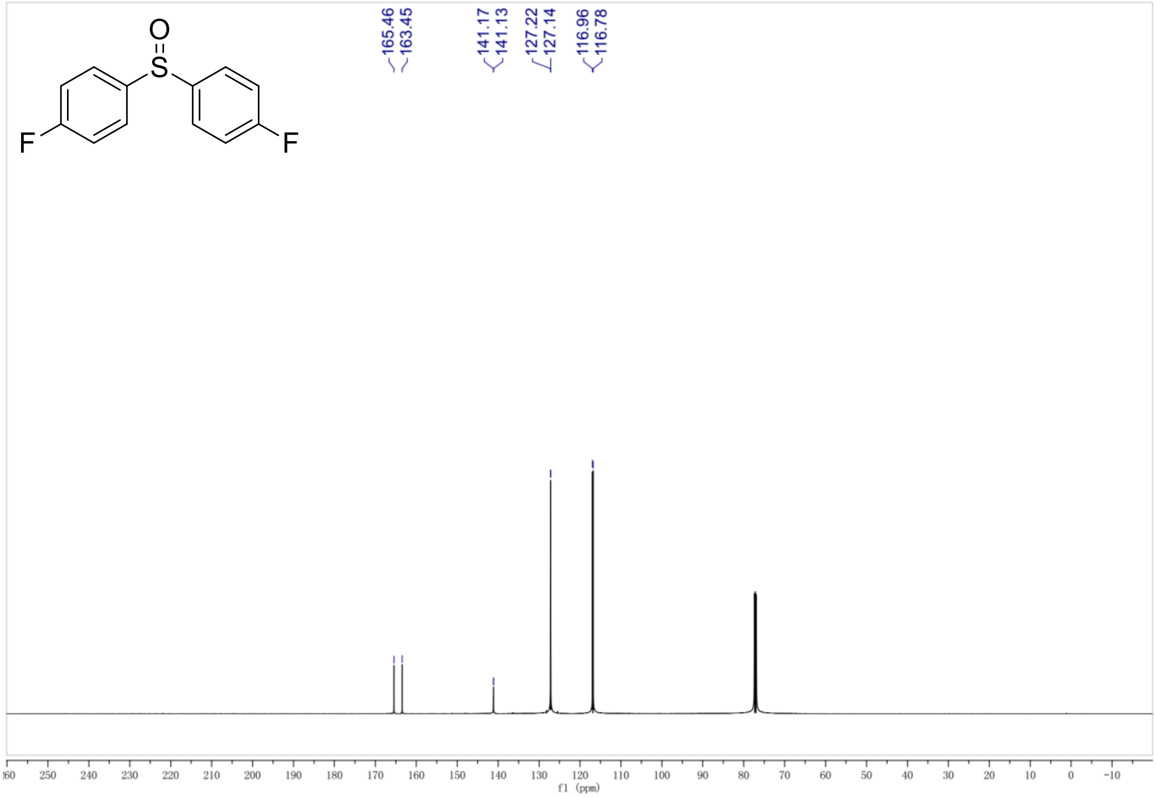

^{13}C{^{1}H} NMR spectrum of compound **1e** in $CDCl_3$ (125 MHz).

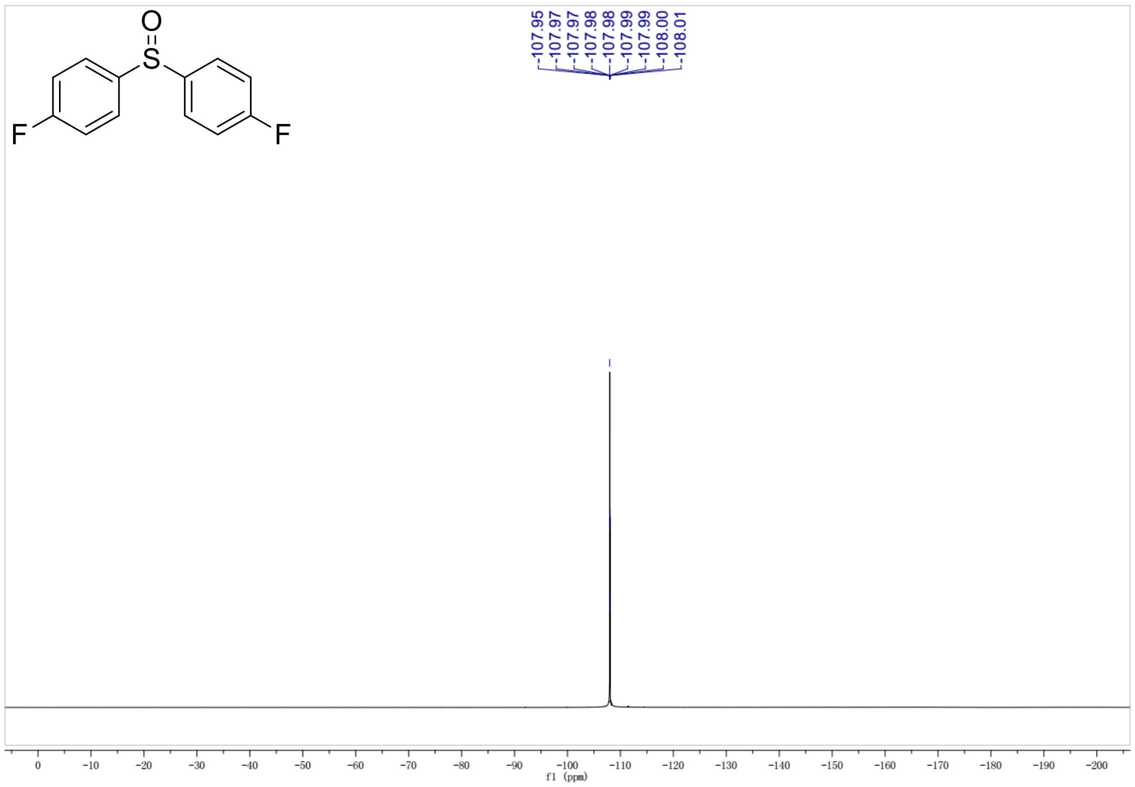

$^{19}F\{^{1}H\}$ NMR spectrum of compound **1e** in $CDCl_3$ (470 MHz).

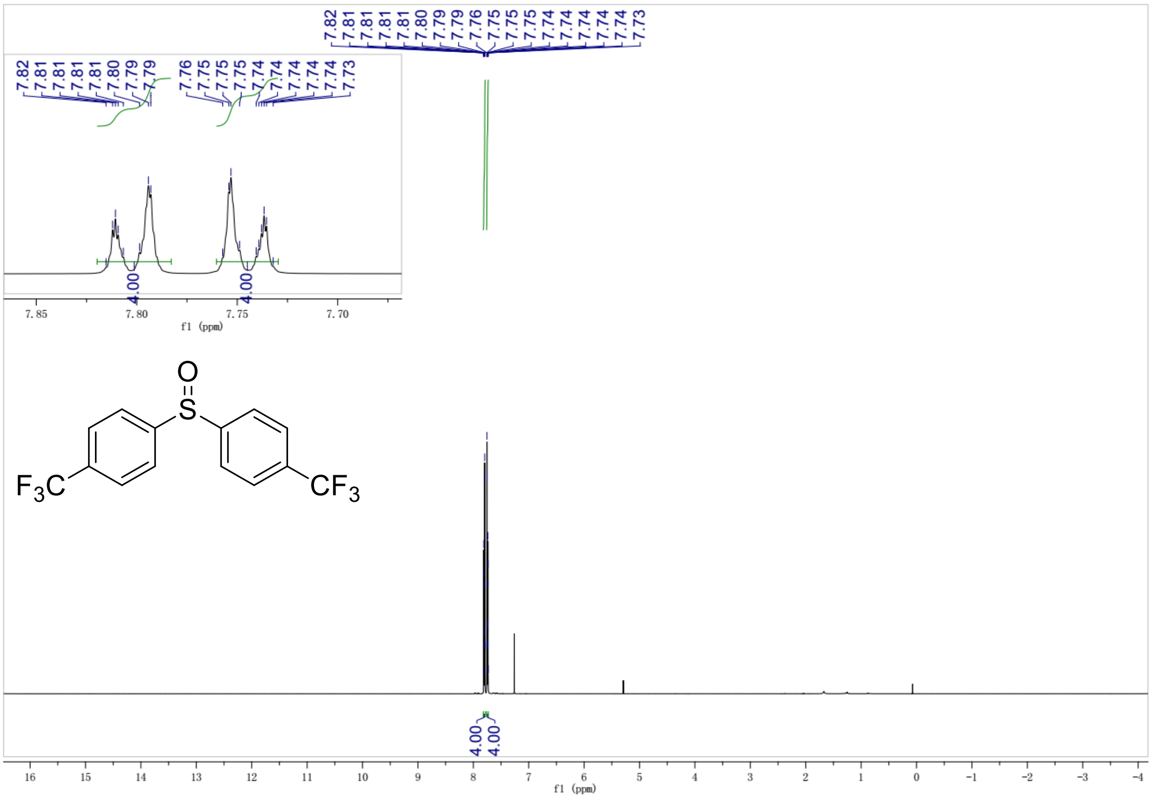

^{1}H NMR spectrum of compound **1f** in $CDCl_3$ (500 MHz).

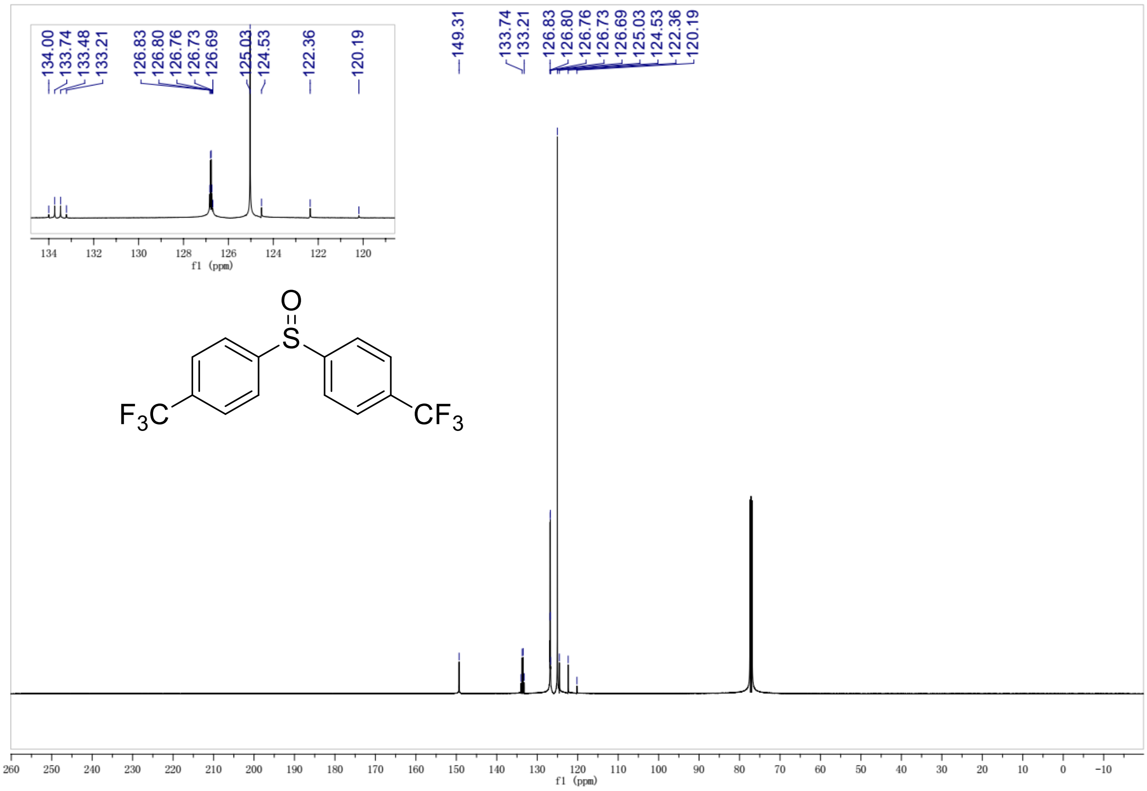

$^{13}C\{^1H\}$ NMR spectrum of compound **1f** in $CDCl_3$ (125 MHz).

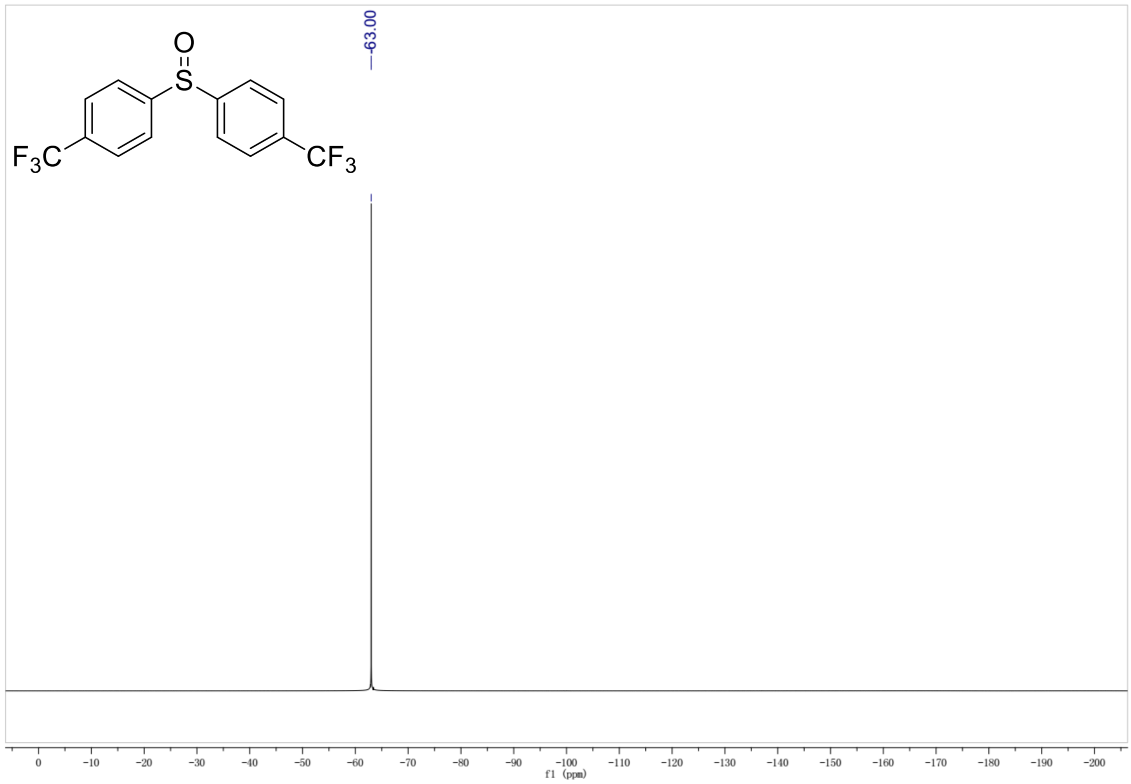

$^{19}F\{^1H\}$ NMR spectrum of compound **1f** in $CDCl_3$ (470 MHz).

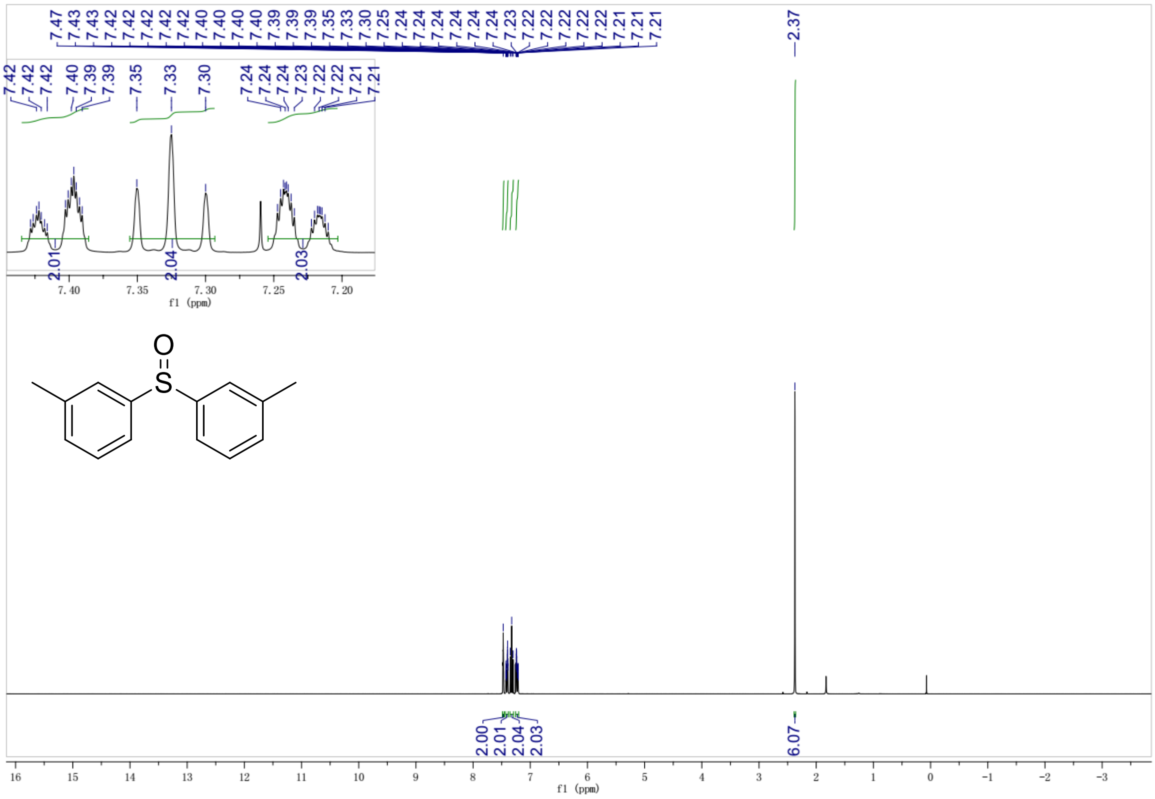

^{1}H NMR spectrum of compound **1h** in $CDCl_3$ (300 MHz).

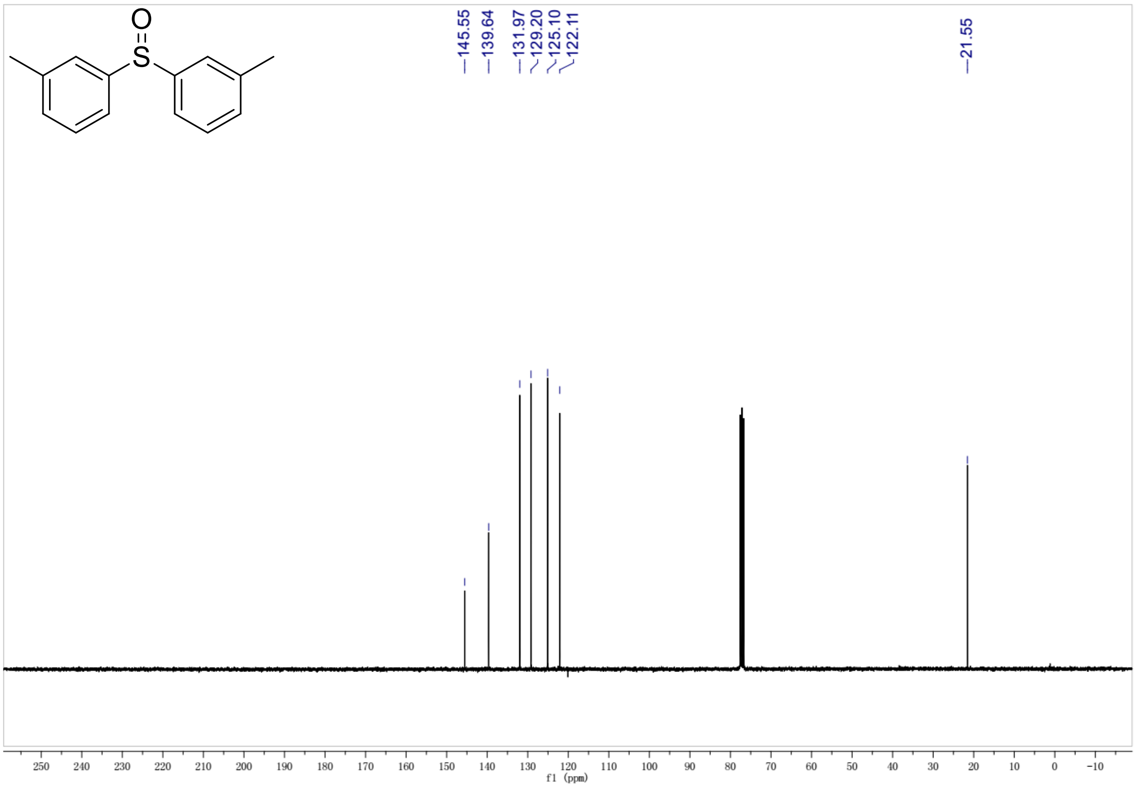

^{13}C{^{1}H} NMR spectrum of compound **1h** in $CDCl_3$ (75 MHz).

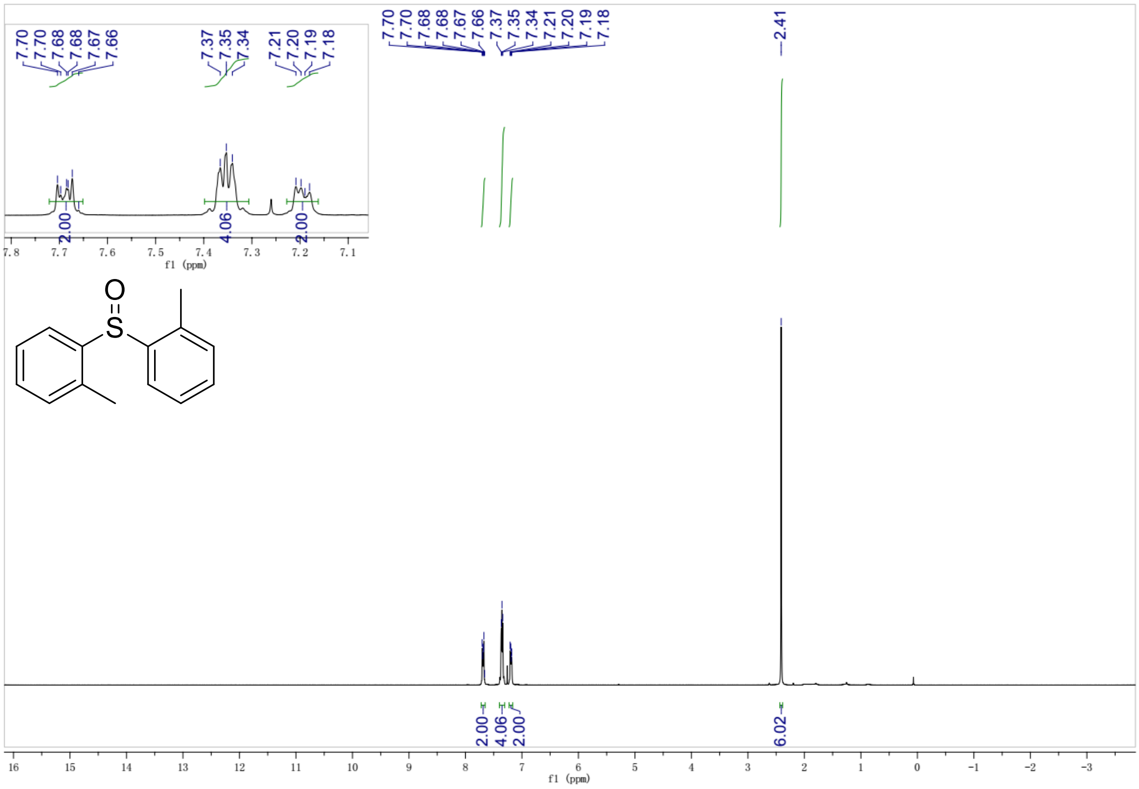

^{1}H NMR spectrum of compound **1i** in $CDCl_3$ (300 MHz).

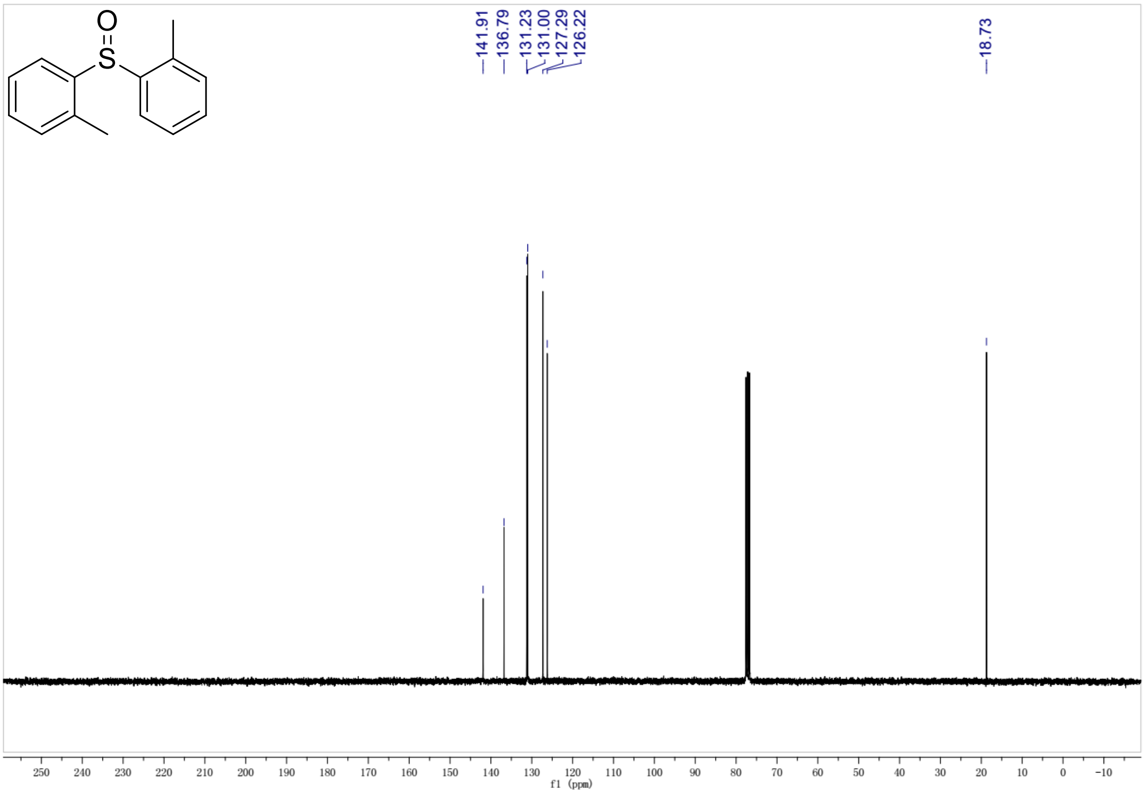

$^{13}C\{^1H\}$ NMR spectrum of compound **1i** in $CDCl_3$ (75 MHz).

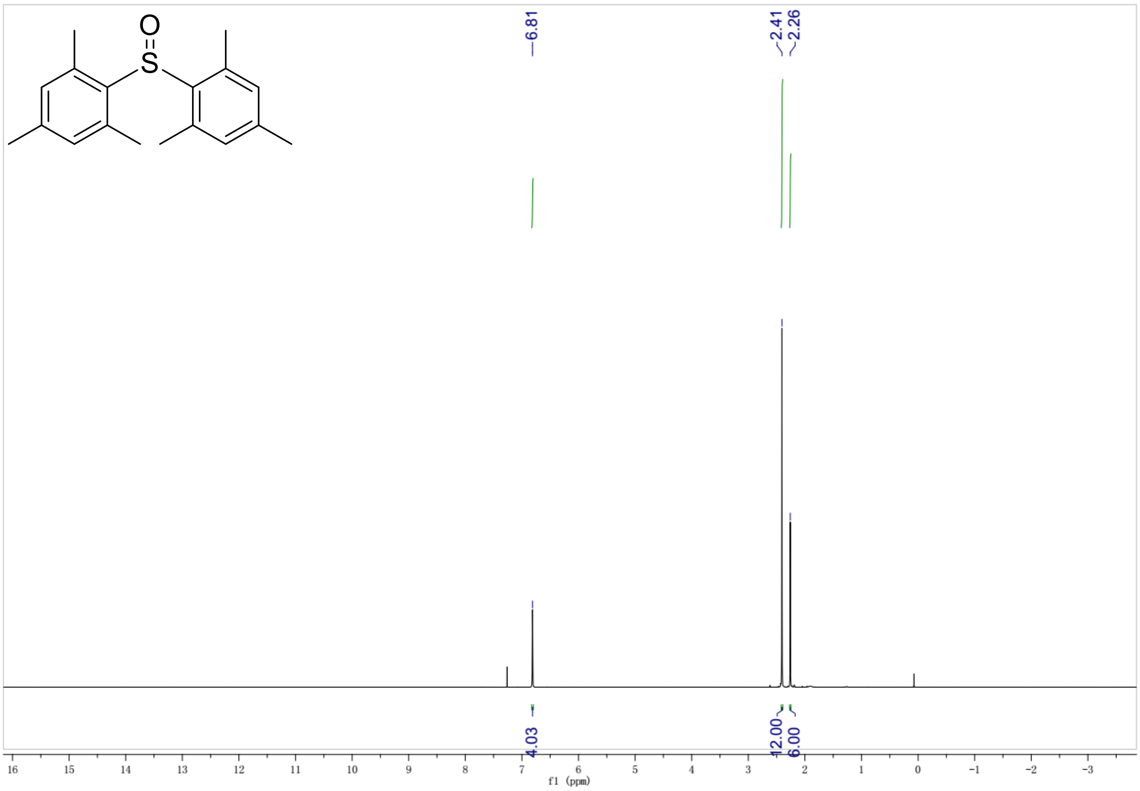

^{1}H NMR spectrum of compound **1j** in $CDCl_3$ (300 MHz).

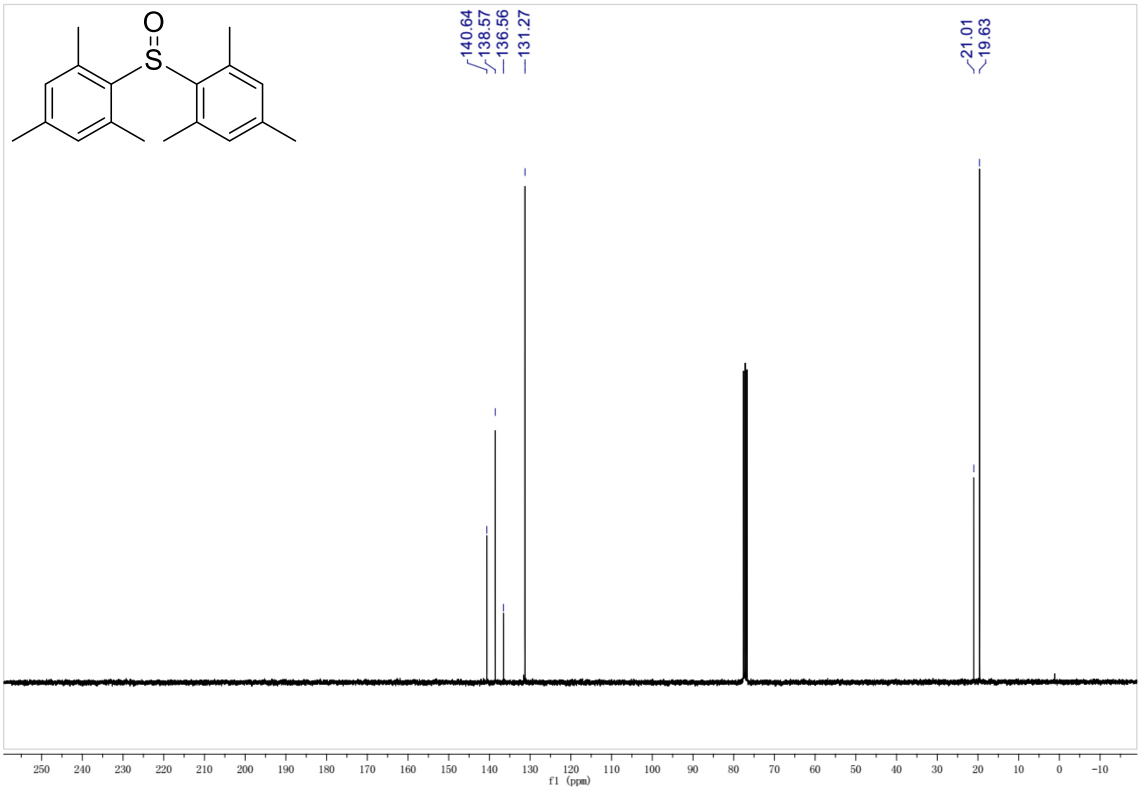

$^{13}C\{^{1}H\}$ NMR spectrum of compound **1j** in $CDCl_3$ (75 MHz).

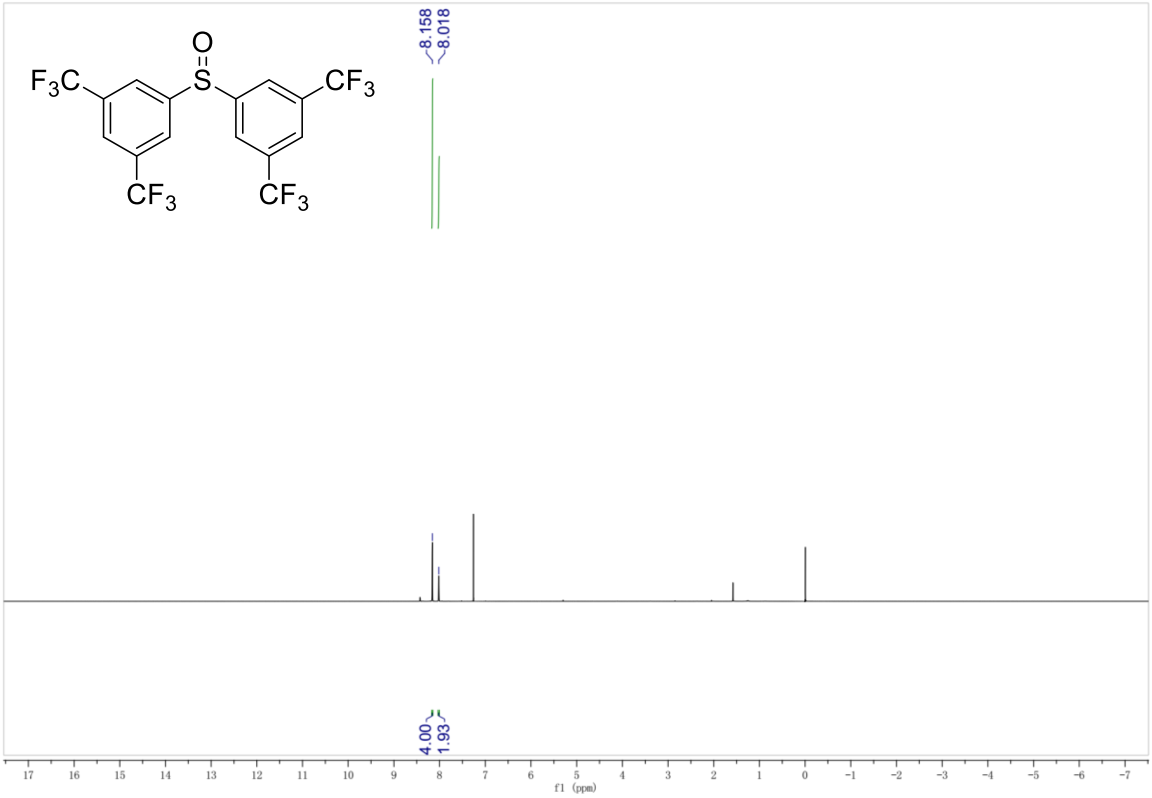

^{1}H NMR spectrum of compound **1k** in $CDCl_3$ (400 MHz).

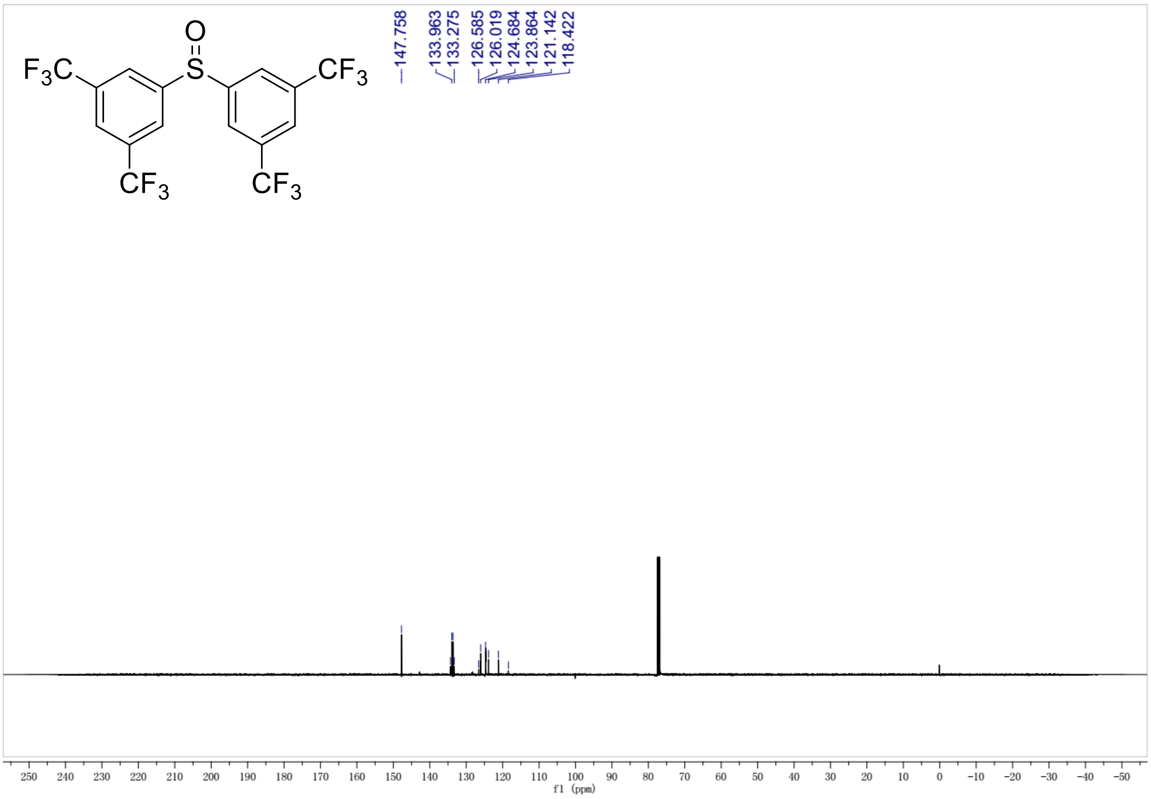

$^{13}C\{^{1}H\}$ NMR spectrum of compound **1k** in $CDCl_3$ (100 MHz).

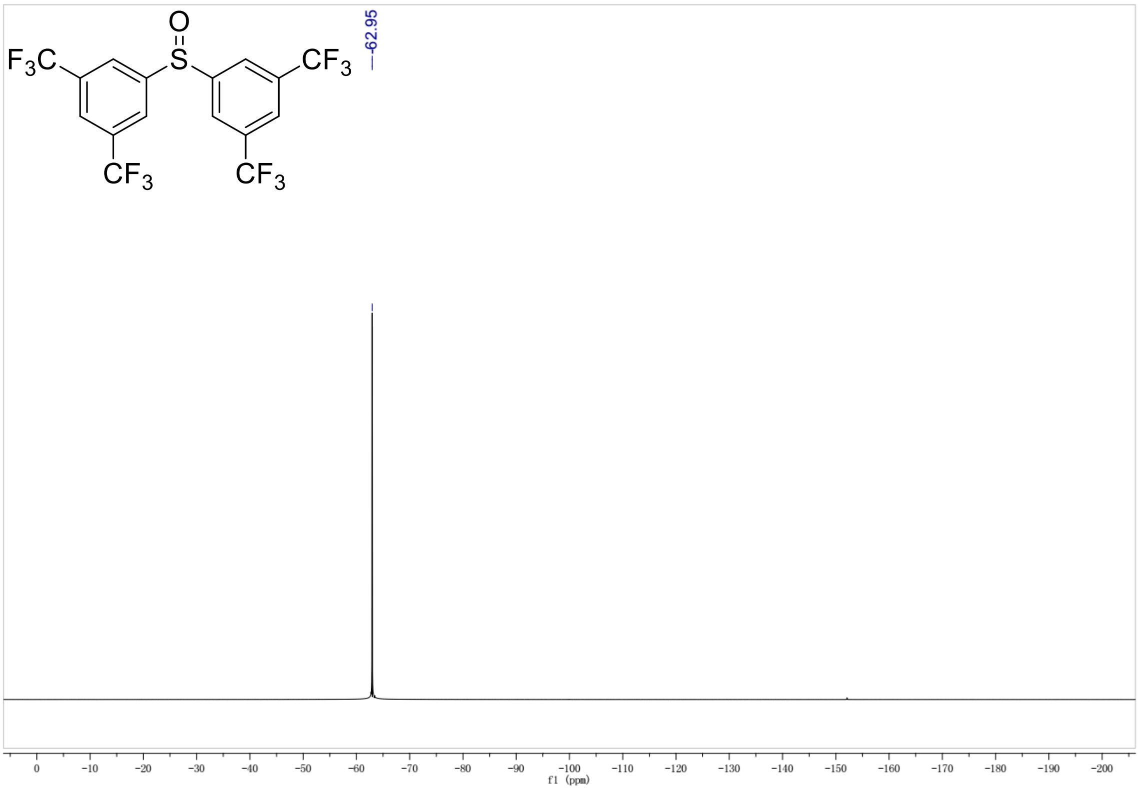

$^{19}F\{^{1}H\}$ NMR spectrum of compound **1k** in $CDCl_3$ (376 MHz).

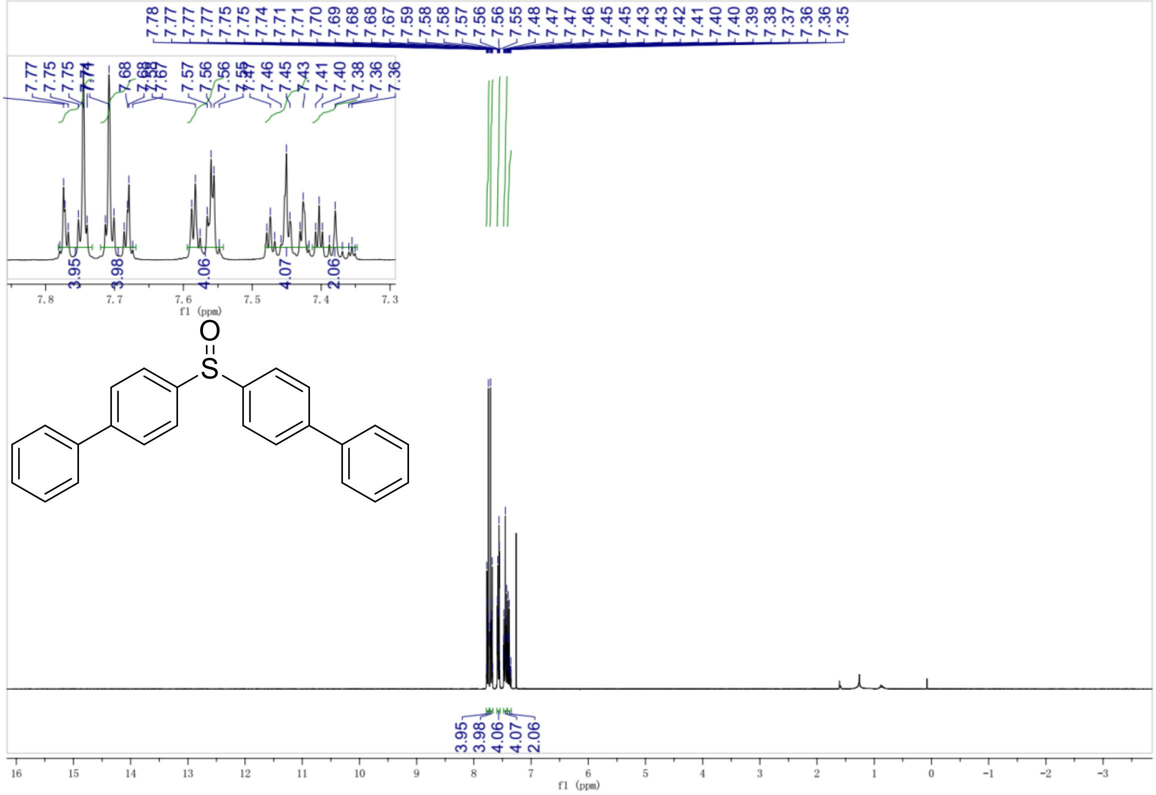

^{1}H NMR spectrum of compound **1l** in $CDCl_3$ (300 MHz).

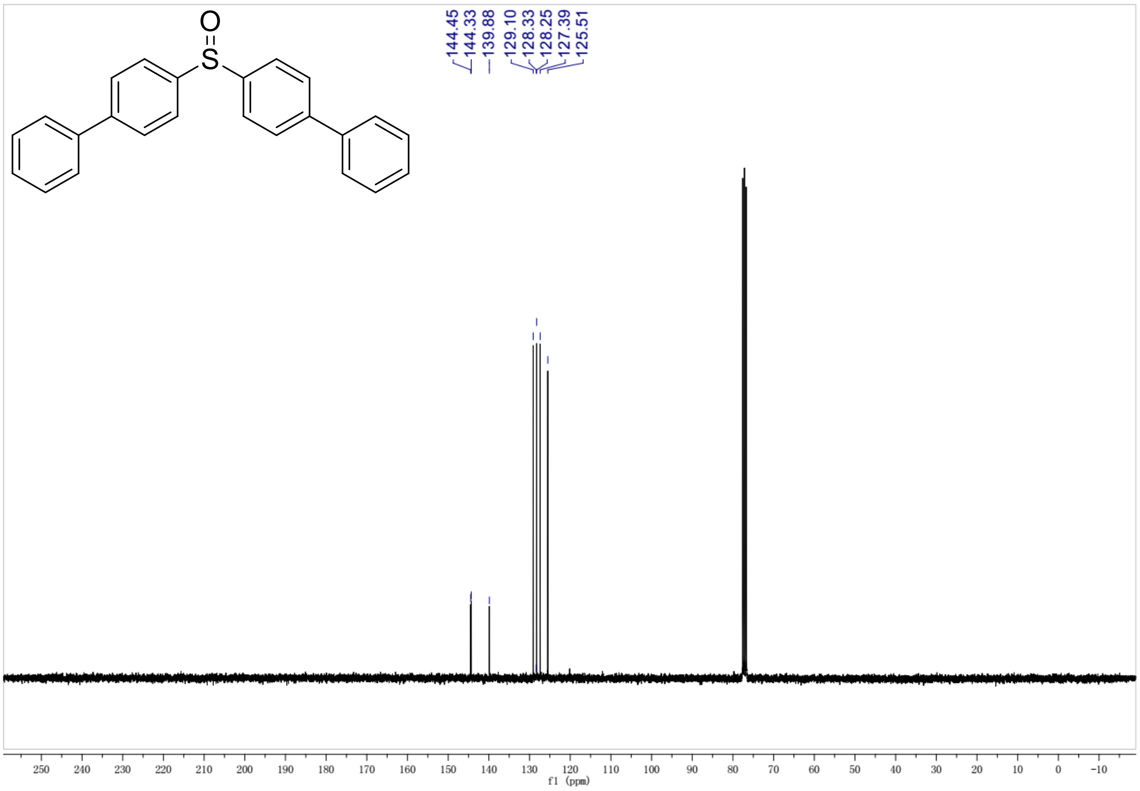

$^{13}C\{^{1}H\}$ NMR spectrum of compound **1l** in $CDCl_3$ (75 MHz).

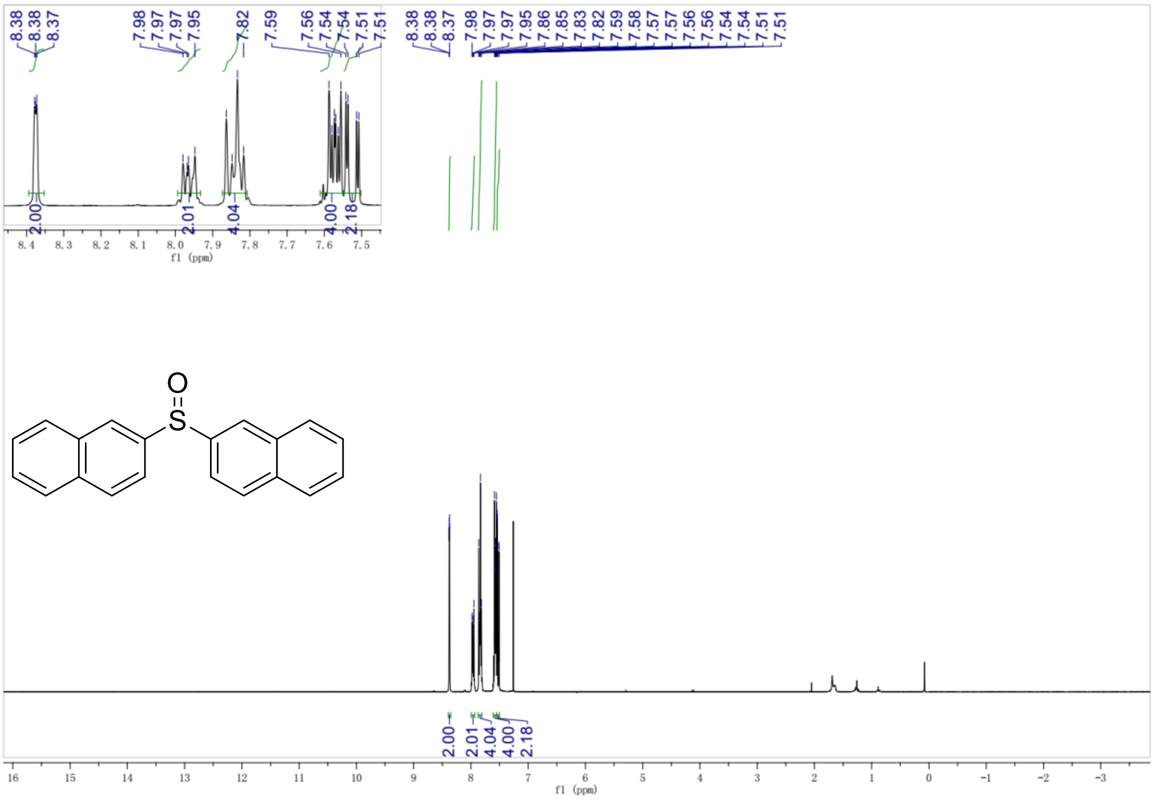

^{1}H NMR spectrum of compound **1m** in $CDCl_3$ (300 MHz).

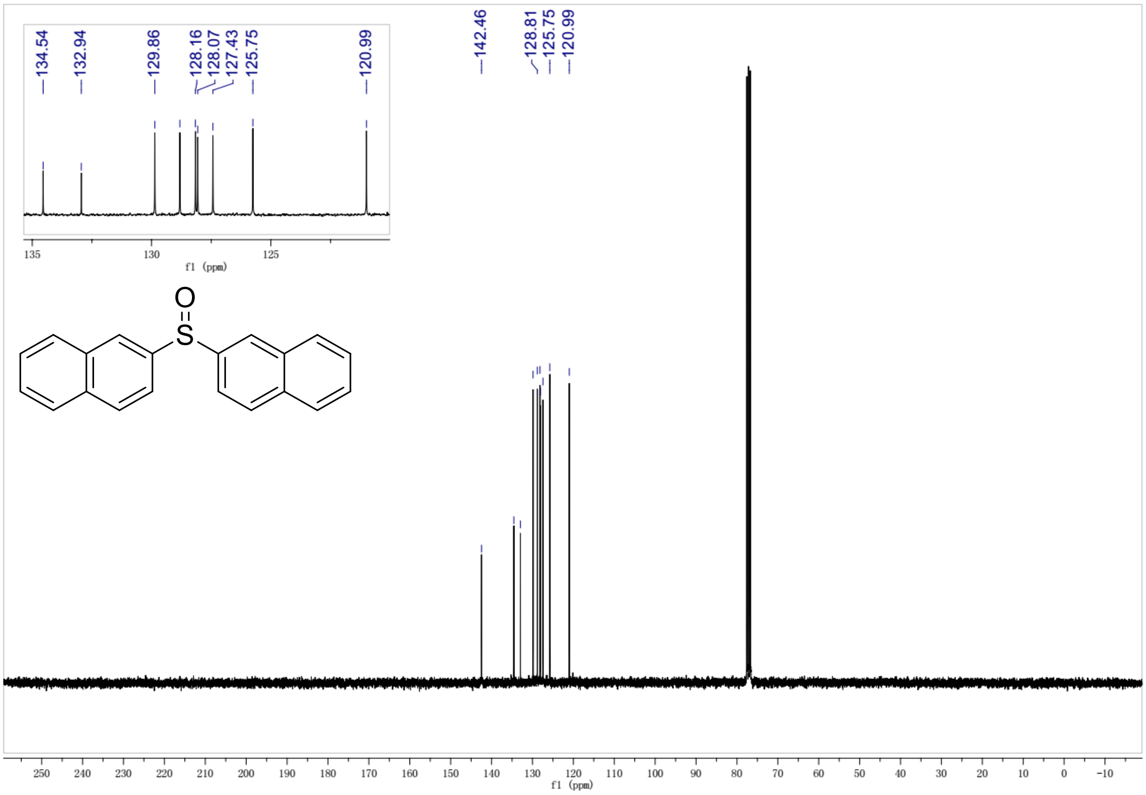

$^{13}C\{^{1}H\}$ NMR spectrum of compound **1m** in $CDCl_3$ (75 MHz).

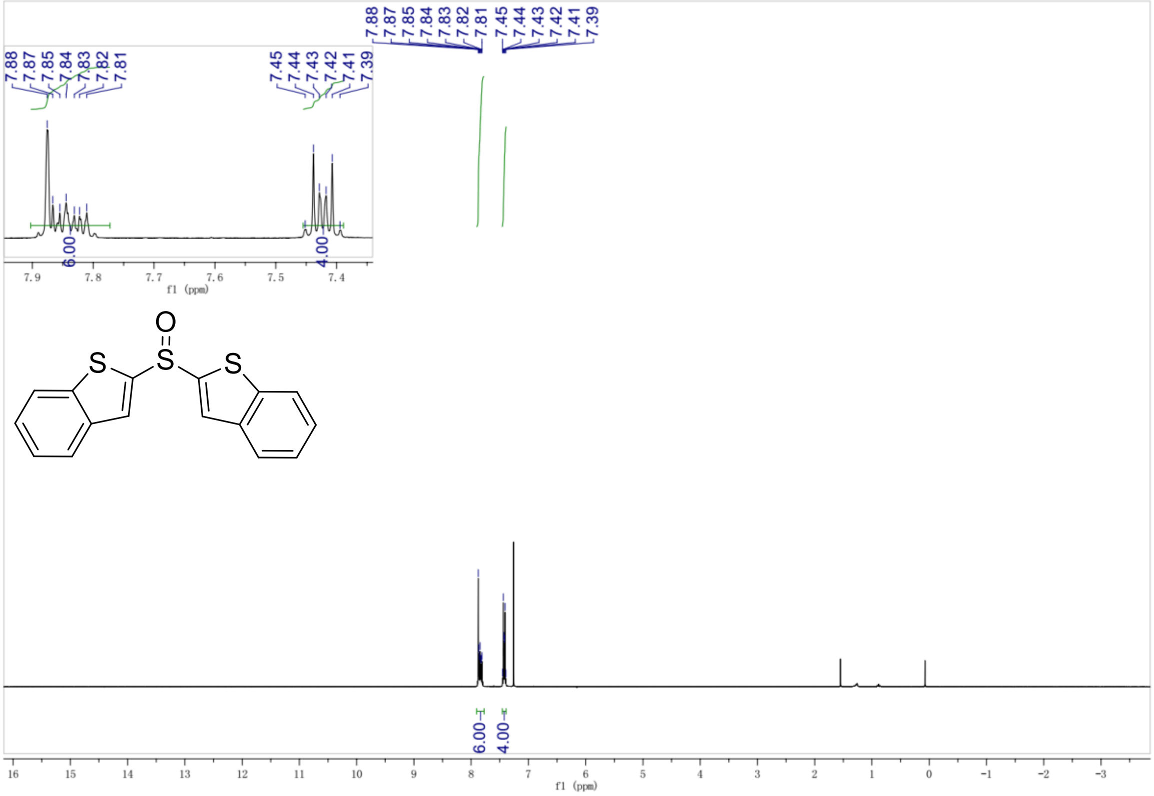

^{1}H NMR spectrum of compound **1n** in $CDCl_3$ (300 MHz).

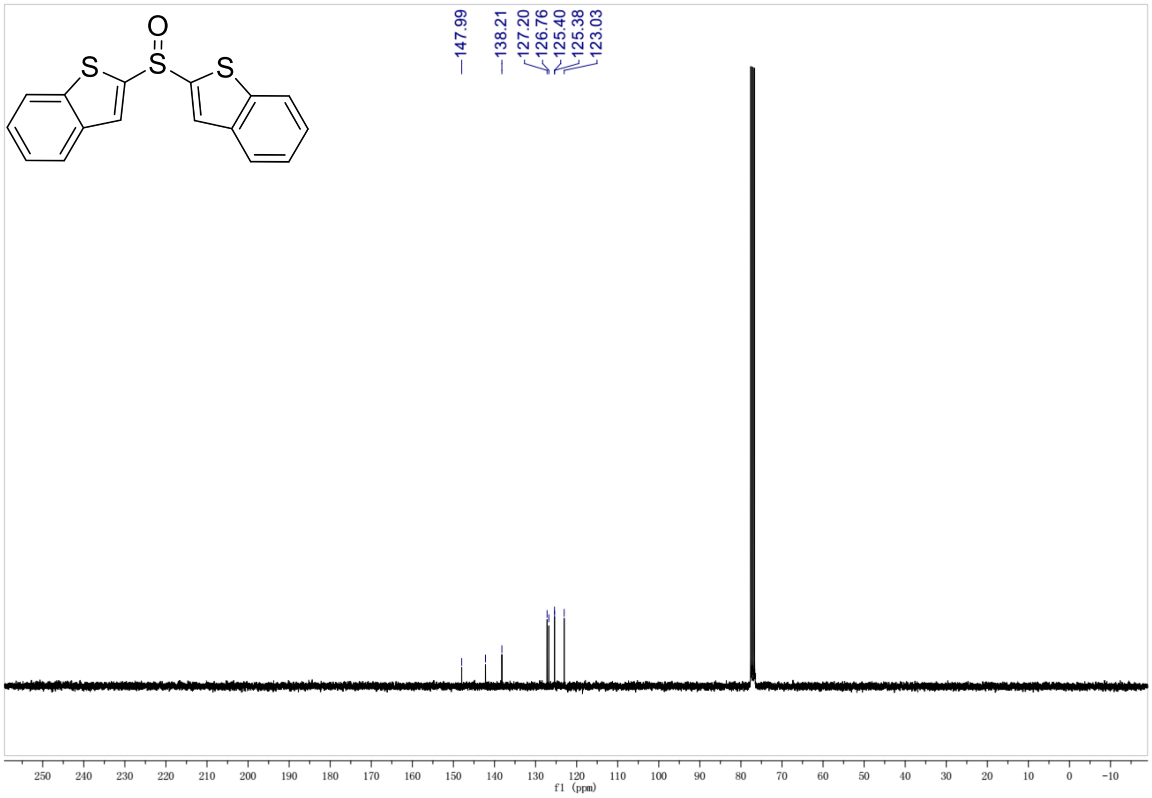

$^{13}C\{^{1}H\}$ NMR spectrum of compound **1n** in $CDCl_3$ (75 MHz).

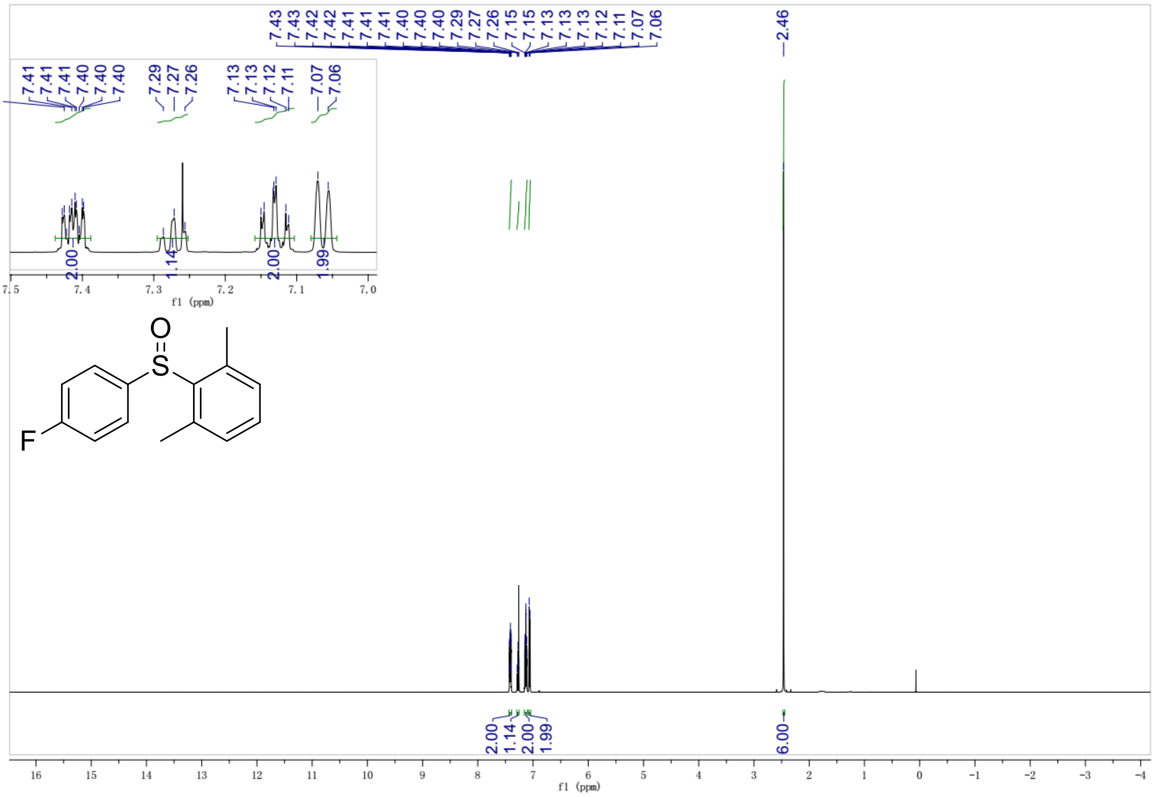

^{1}H NMR spectrum of compound **1o** in $CDCl_3$ (500 MHz).

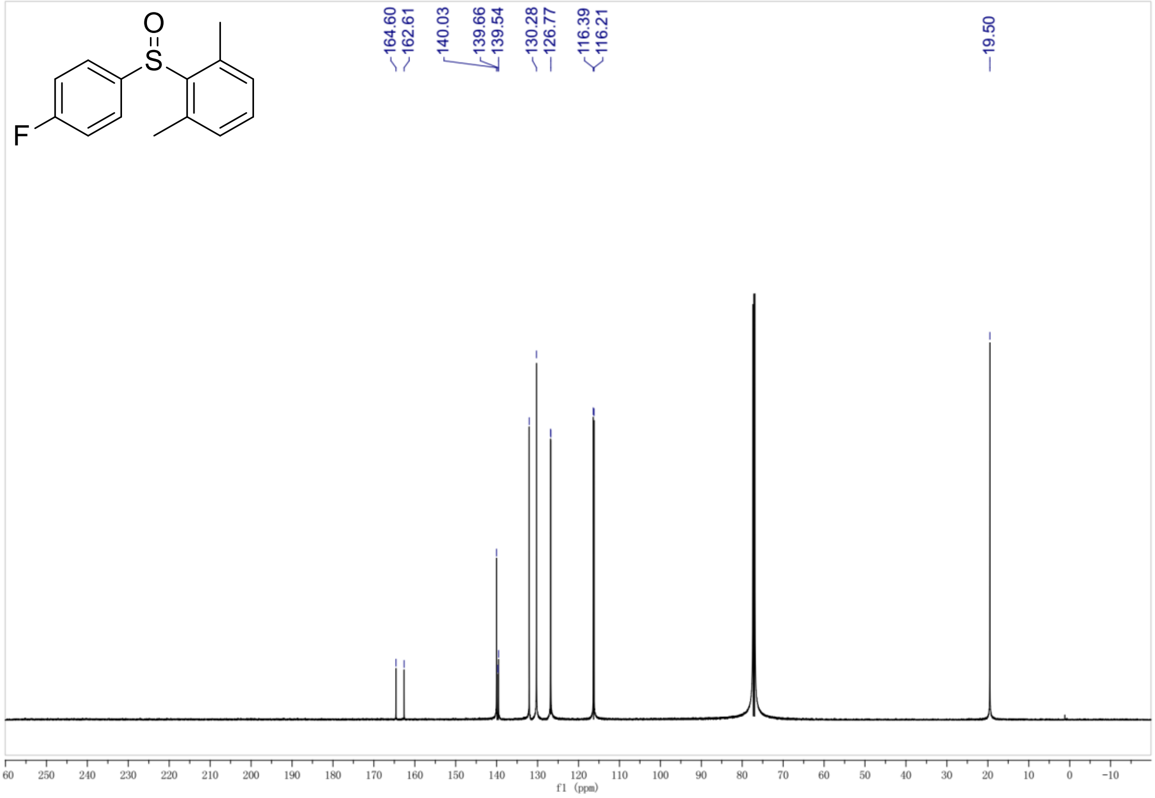

$^{13}C\{^{1}H\}$ NMR spectrum of compound **1o** in $CDCl_3$ (125 MHz).

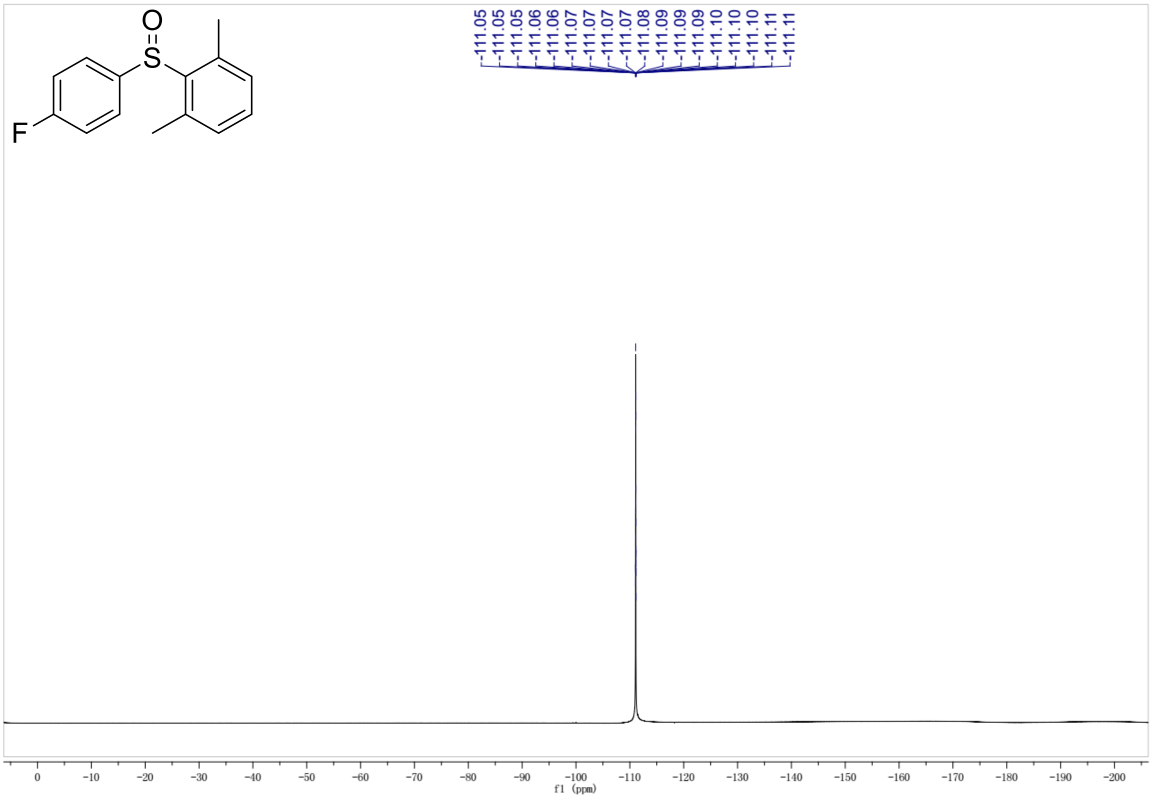

$^{19}F\{^{1}H\}$ NMR spectrum of compound **1o** in $CDCl_3$ (470 MHz).

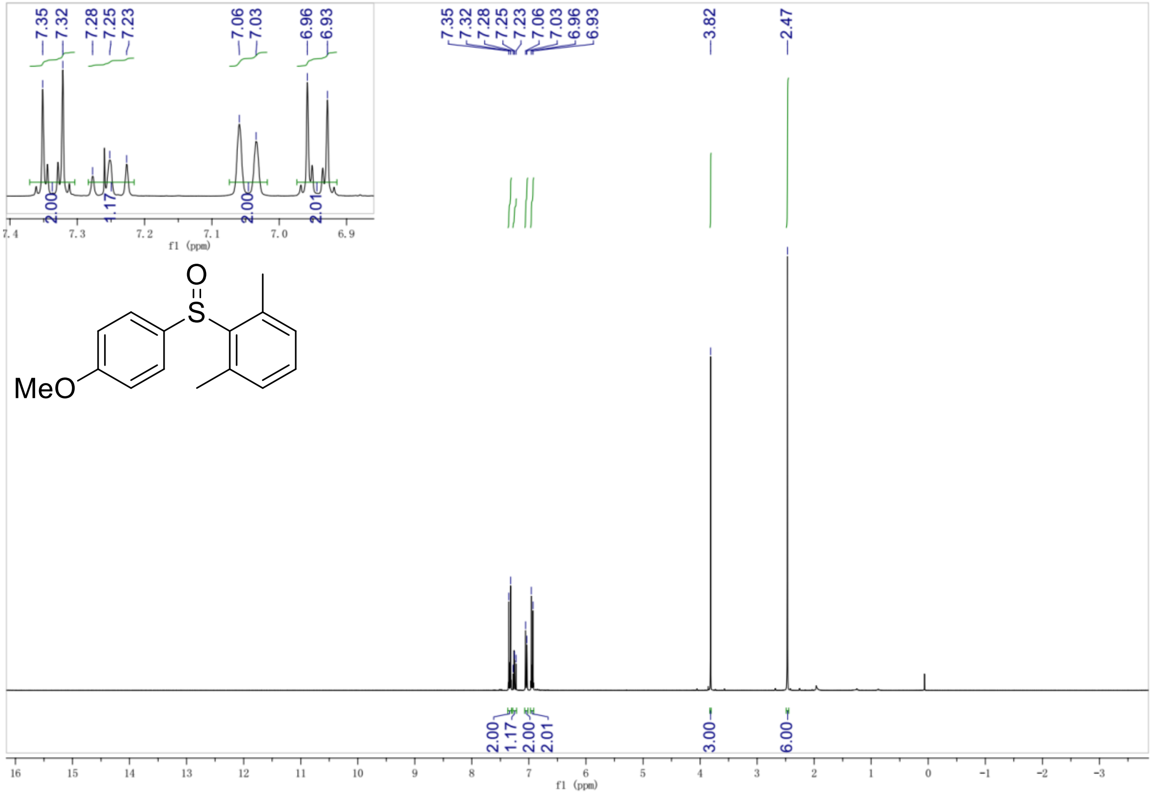

^{1}H NMR spectrum of compound **1p** in $CDCl_3$ (300 MHz).

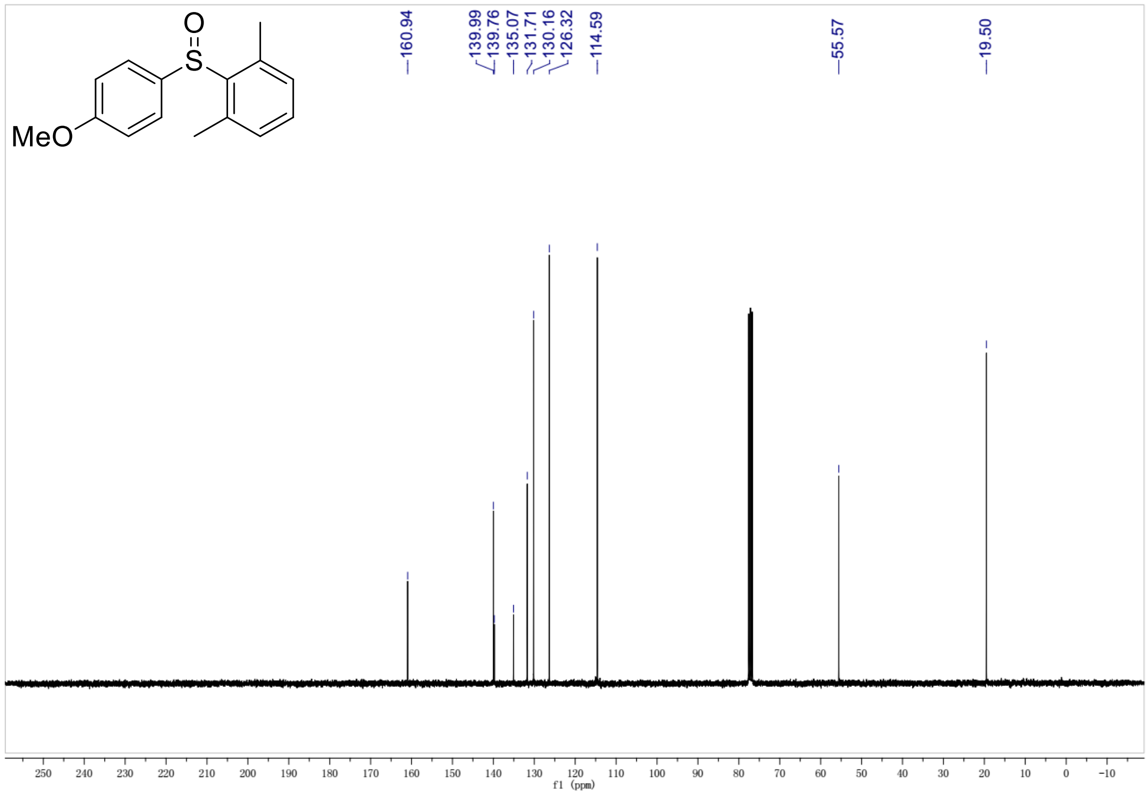

^{13}C{^{1}H} NMR spectrum of compound **1p** in $CDCl_3$ (75 MHz).

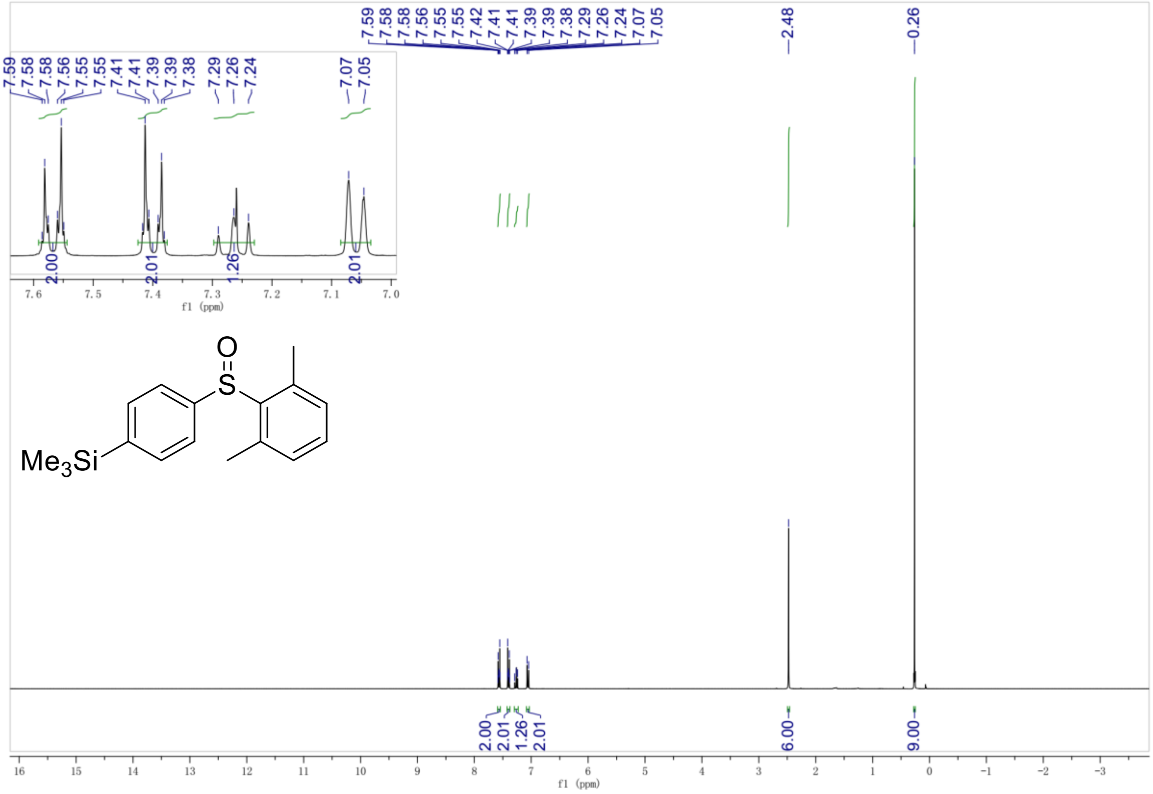

^{1}H NMR spectrum of compound **1q** in $CDCl_3$ (300 MHz).

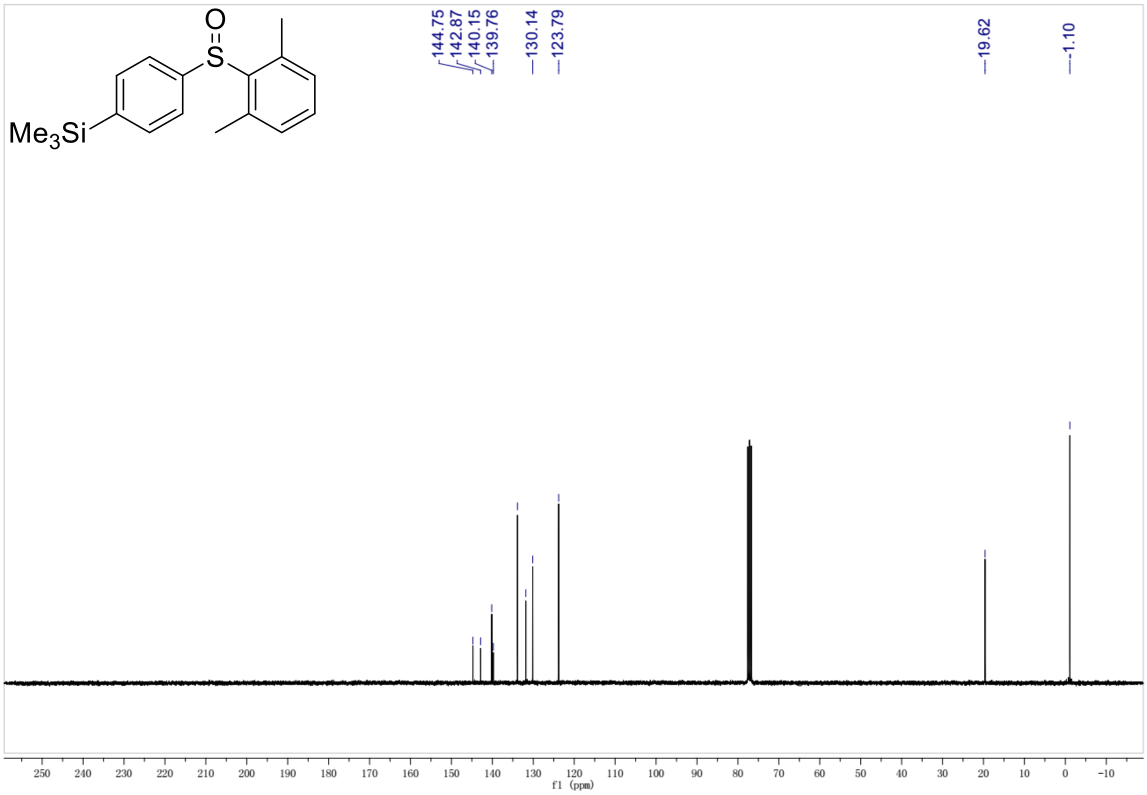

^{13}C{^{1}H} NMR spectrum of compound **1q** in $CDCl_3$ (75 MHz).

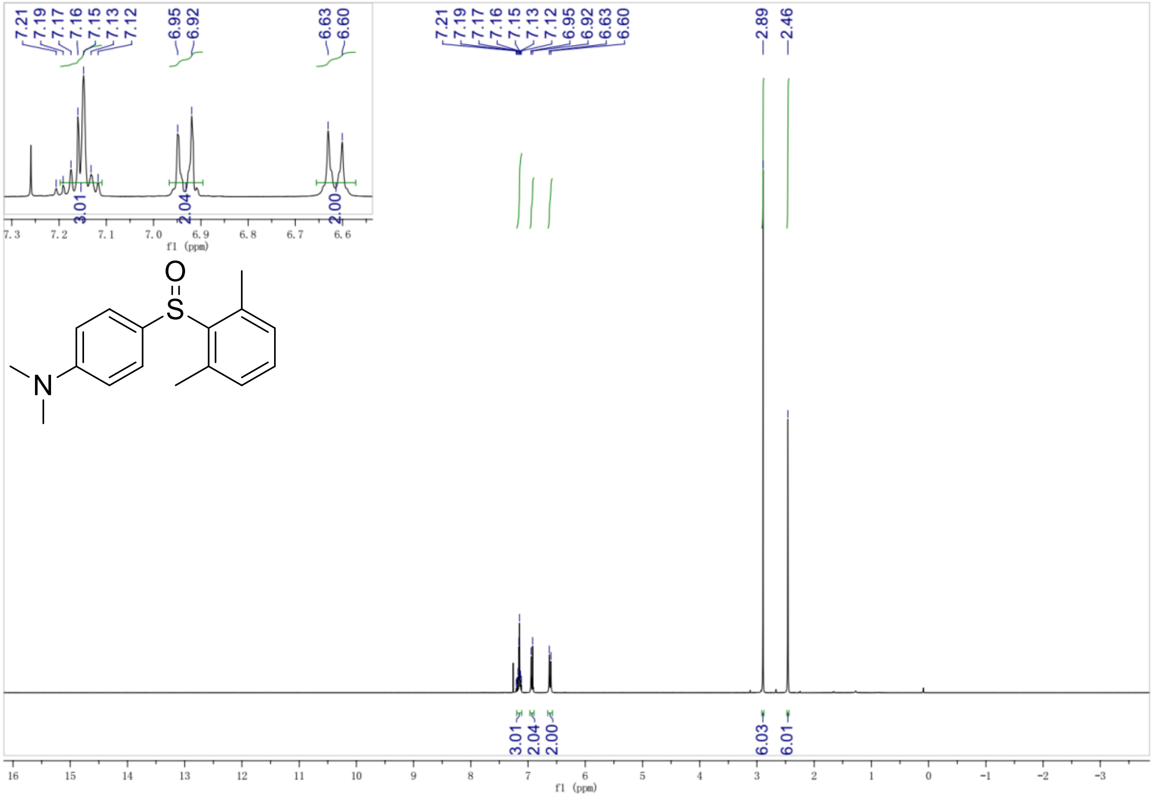

^{1}H NMR spectrum of compound **1r** in $CDCl_3$ (300 MHz).

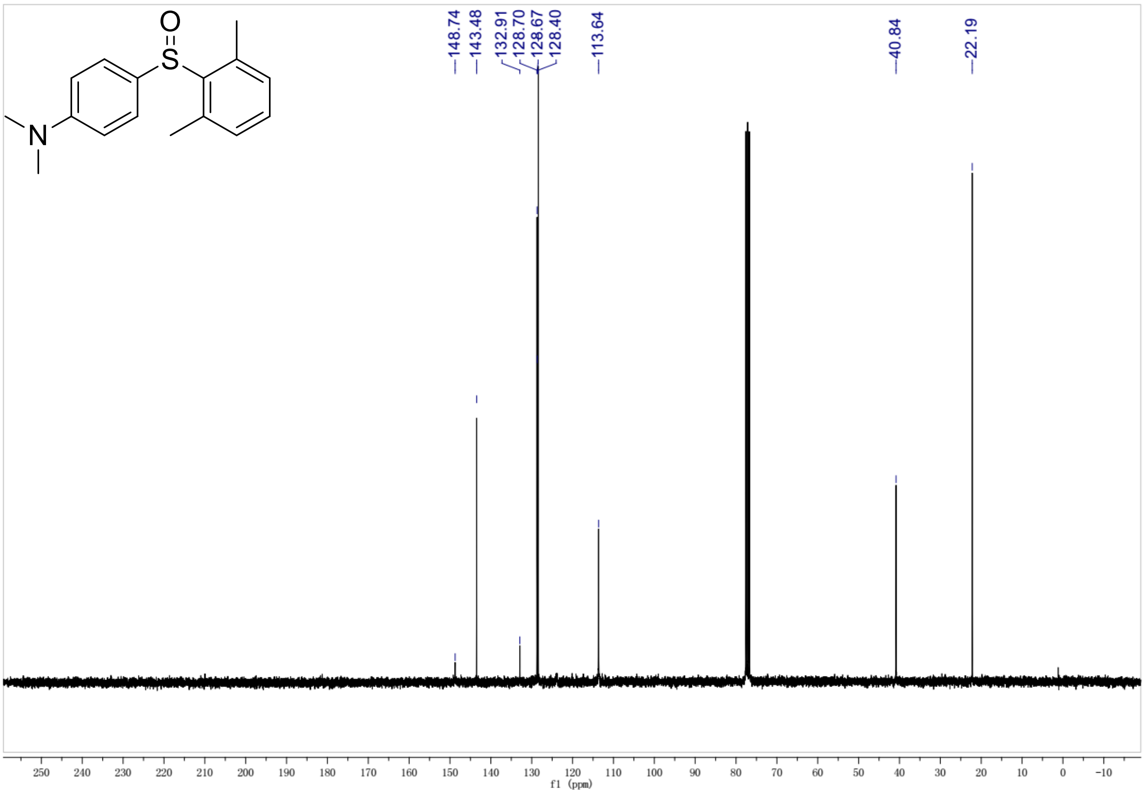

$^{13}C\{^{1}H\}$ NMR spectrum of compound **1r** in $CDCl_3$ (75 MHz).

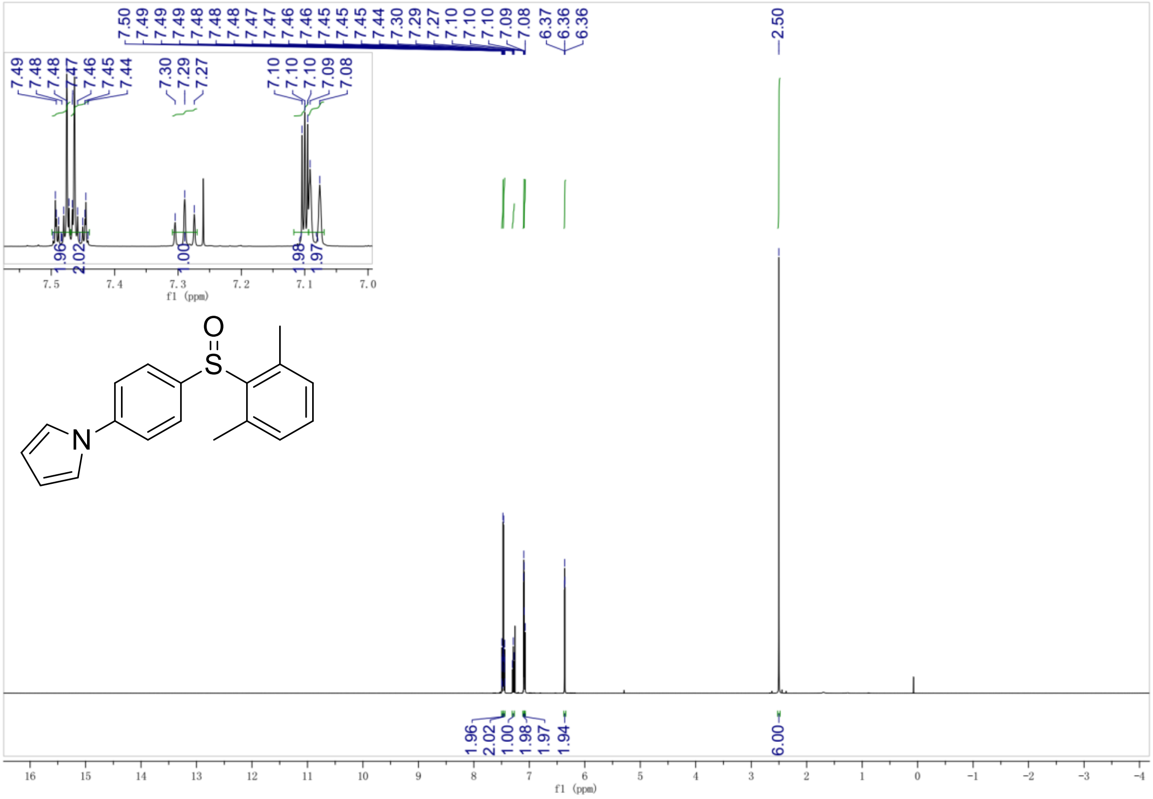

^{1}H NMR spectrum of compound **1s** in $CDCl_3$ (500 MHz).

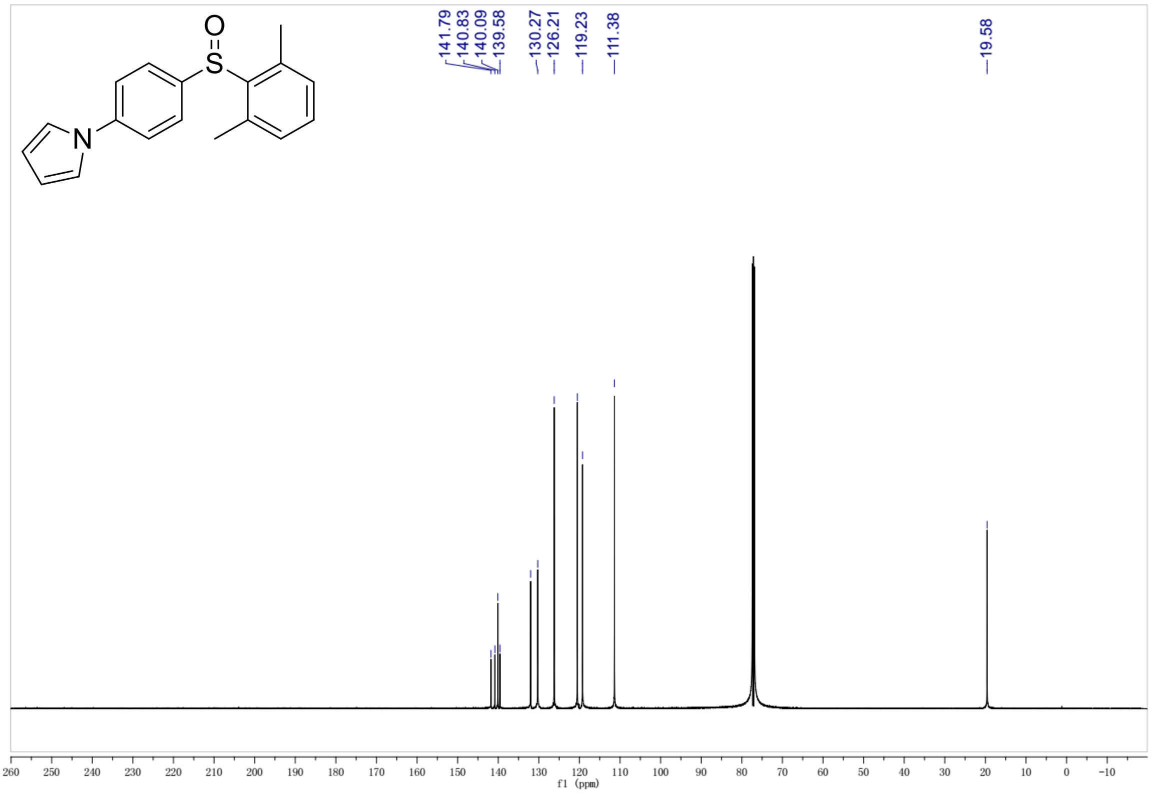

^{13}C{^{1}H} NMR spectrum of compound **1s** in $CDCl_3$ (125 MHz).

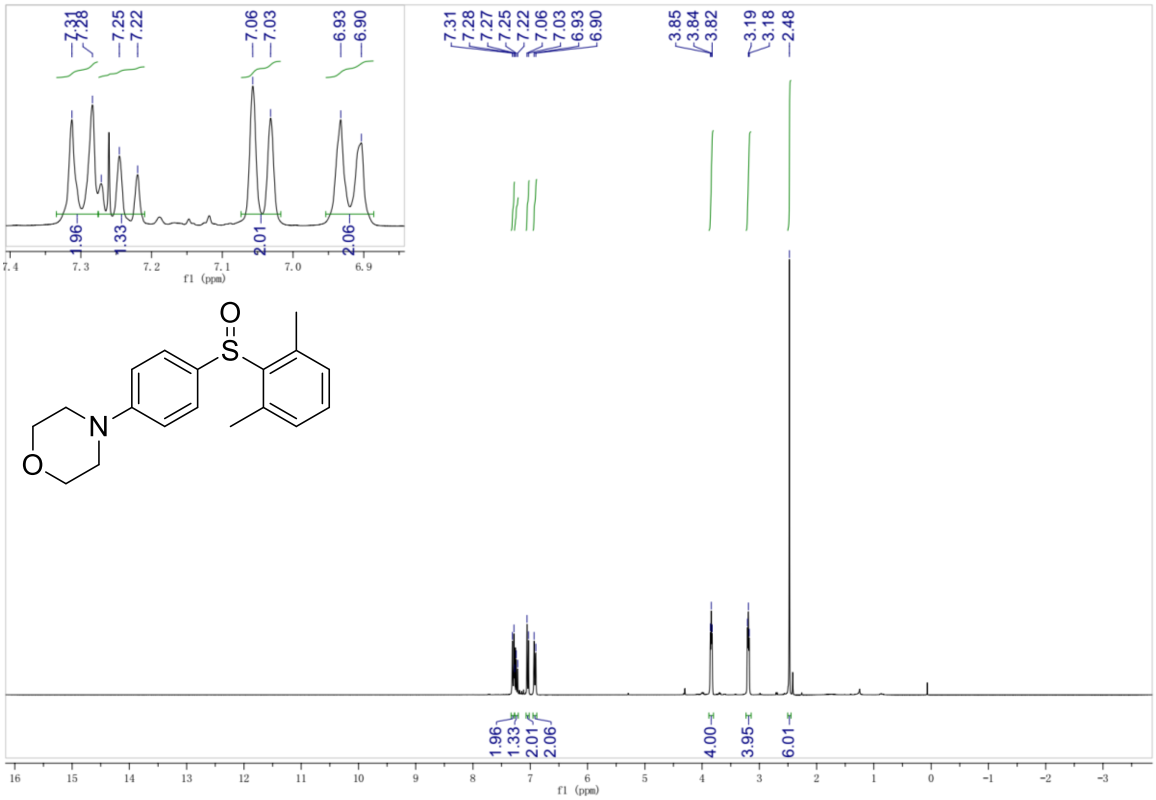

^{1}H NMR spectrum of compound **1t** in $CDCl_3$ (300 MHz).

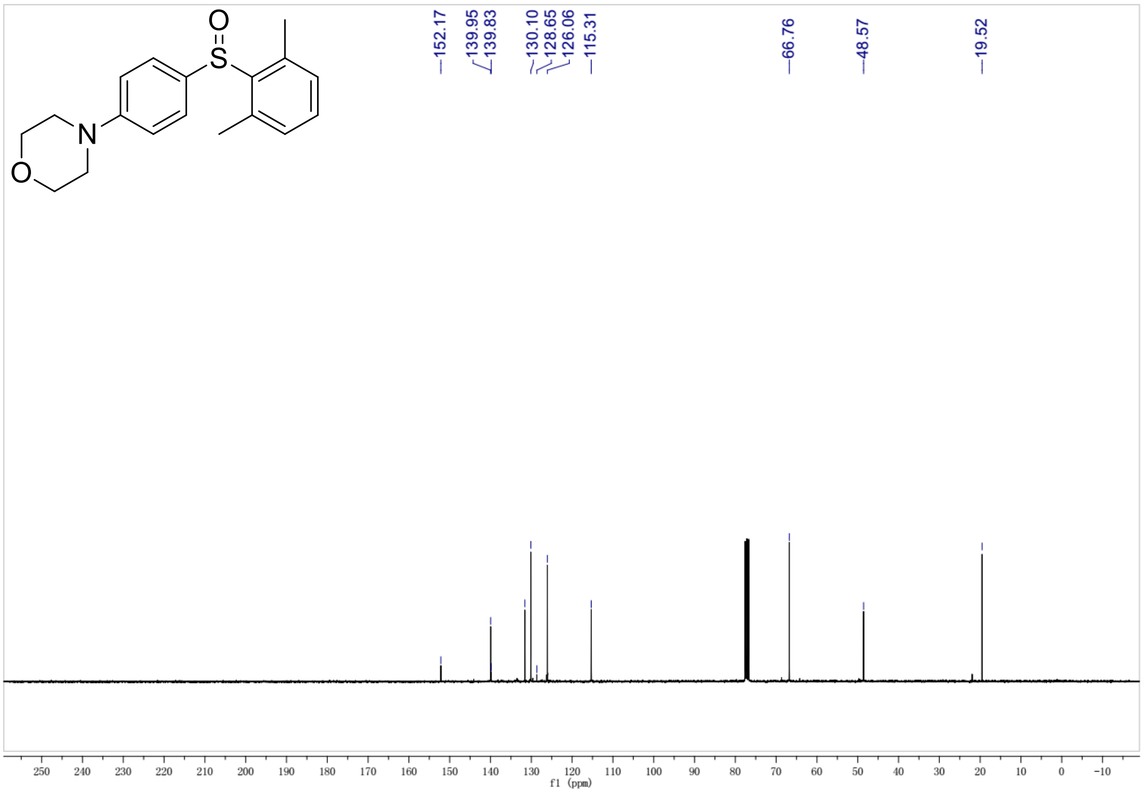

^{13}C{^{1}H} NMR spectrum of compound **1t** in $CDCl_3$ (75 MHz).

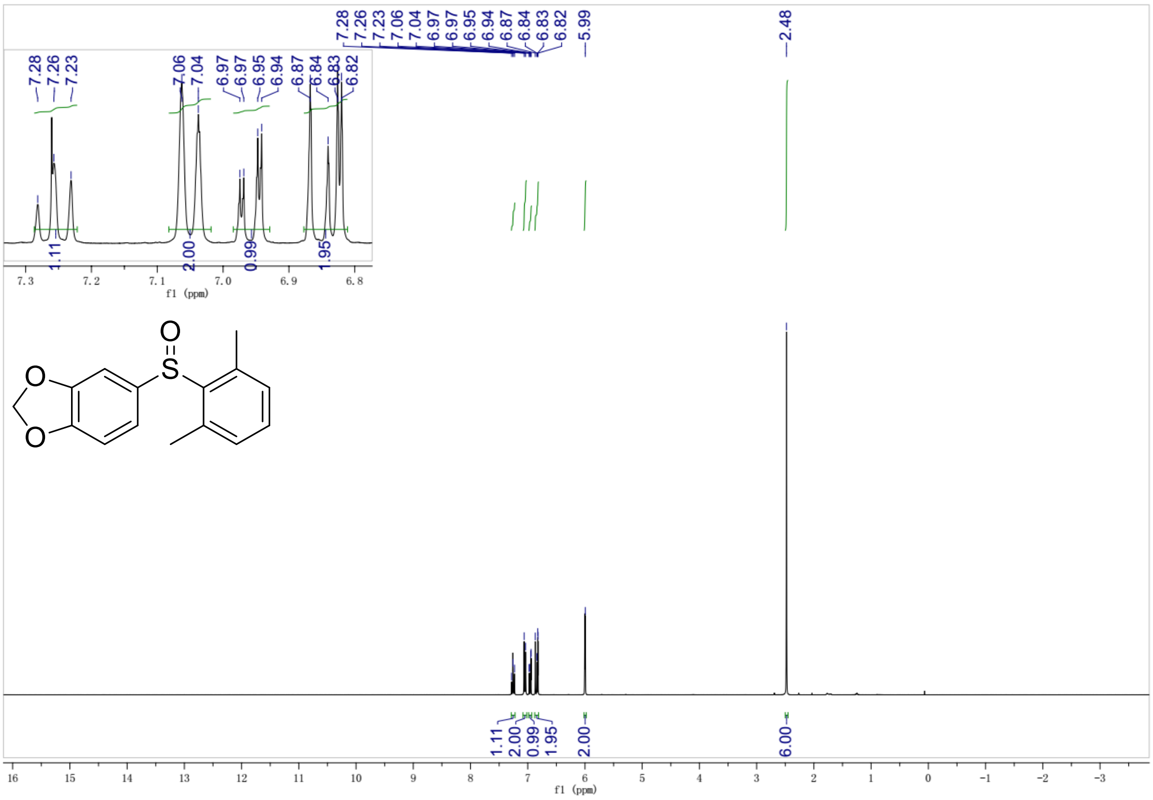

^{1}H NMR spectrum of compound **1u** in $CDCl_3$ (300 MHz).

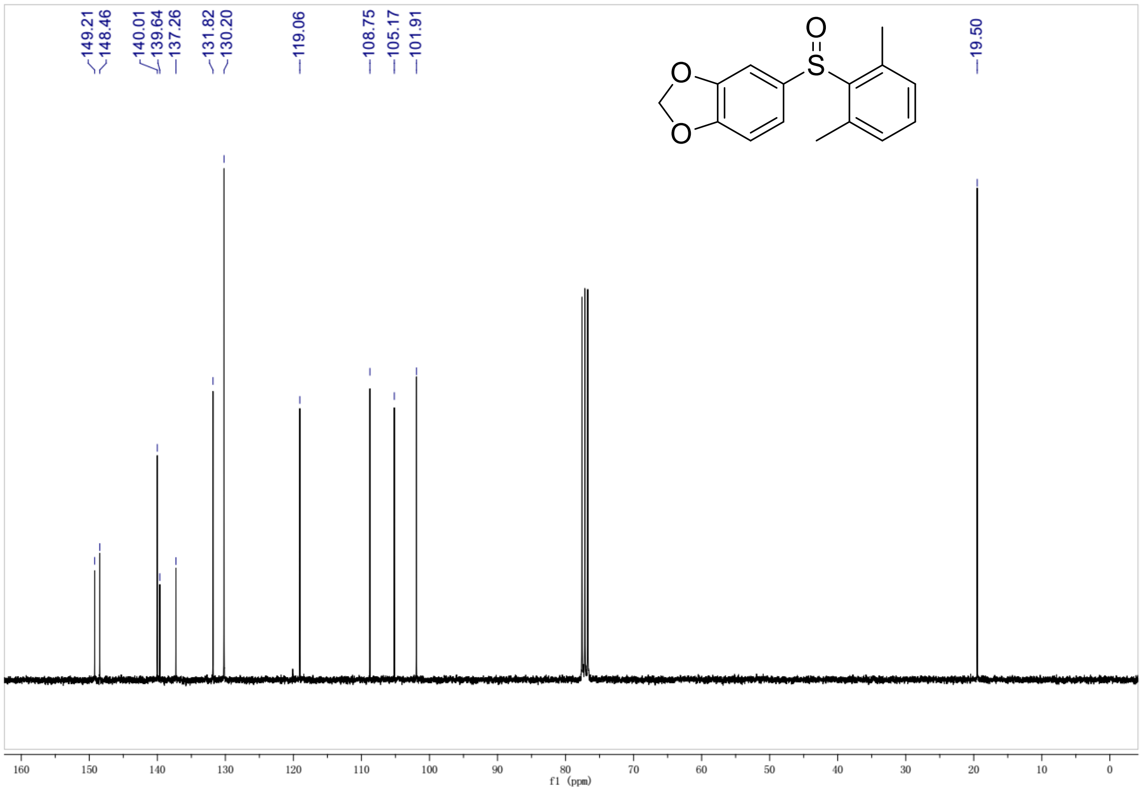

^{13}C{^{1}H} NMR spectrum of compound **1u** in $CDCl_3$ (75 MHz).

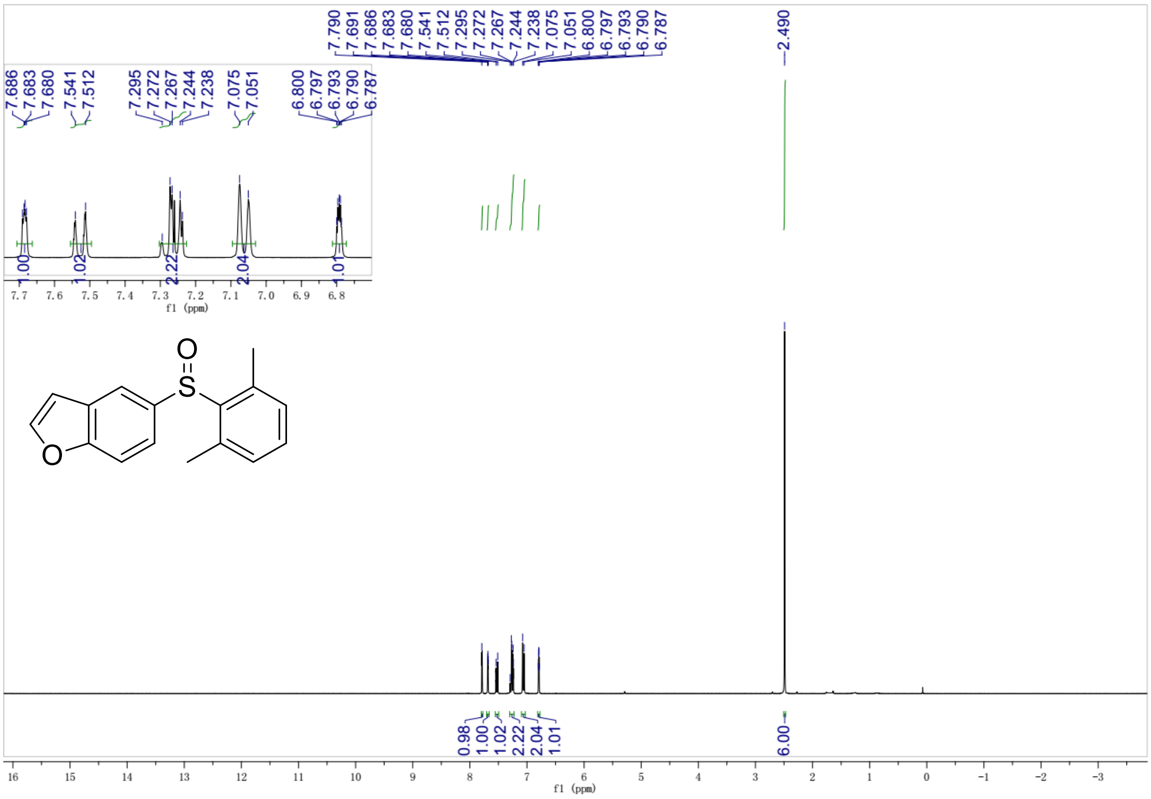

^{1}H NMR spectrum of compound **1v** in $CDCl_3$ (300 MHz).

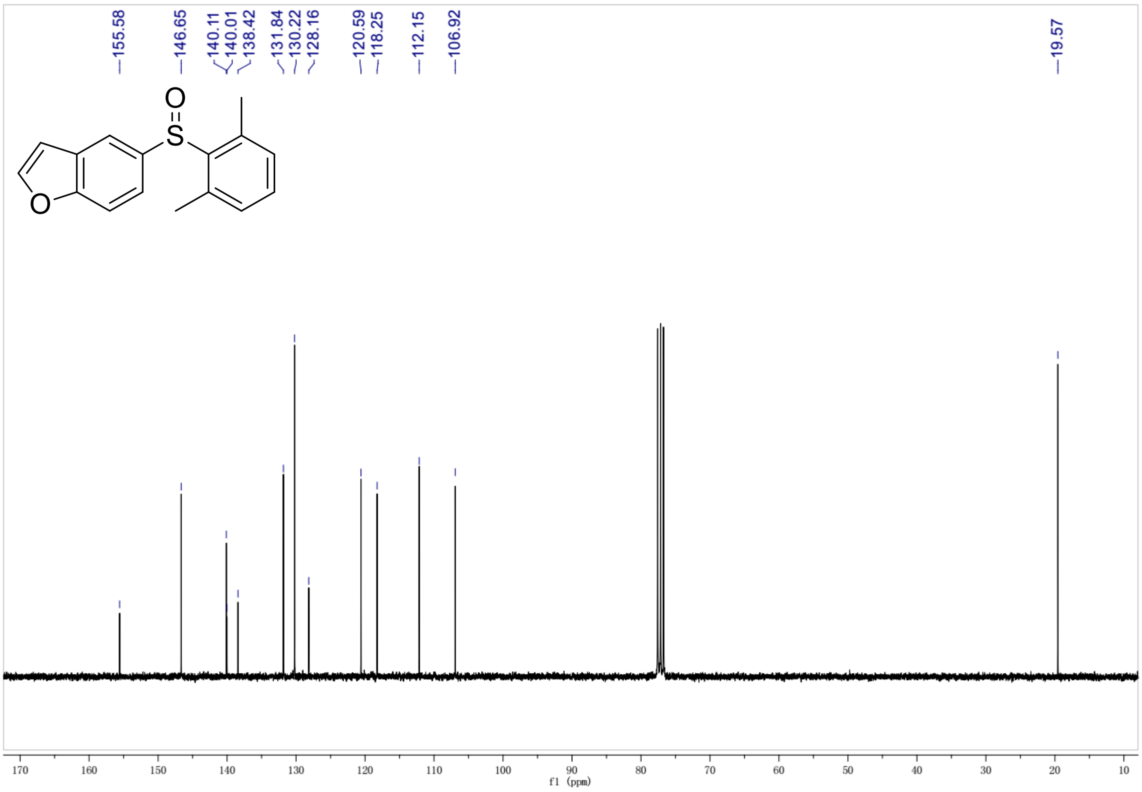

^{13}C{^{1}H} NMR spectrum of compound **1v** in $CDCl_3$ (75 MHz).

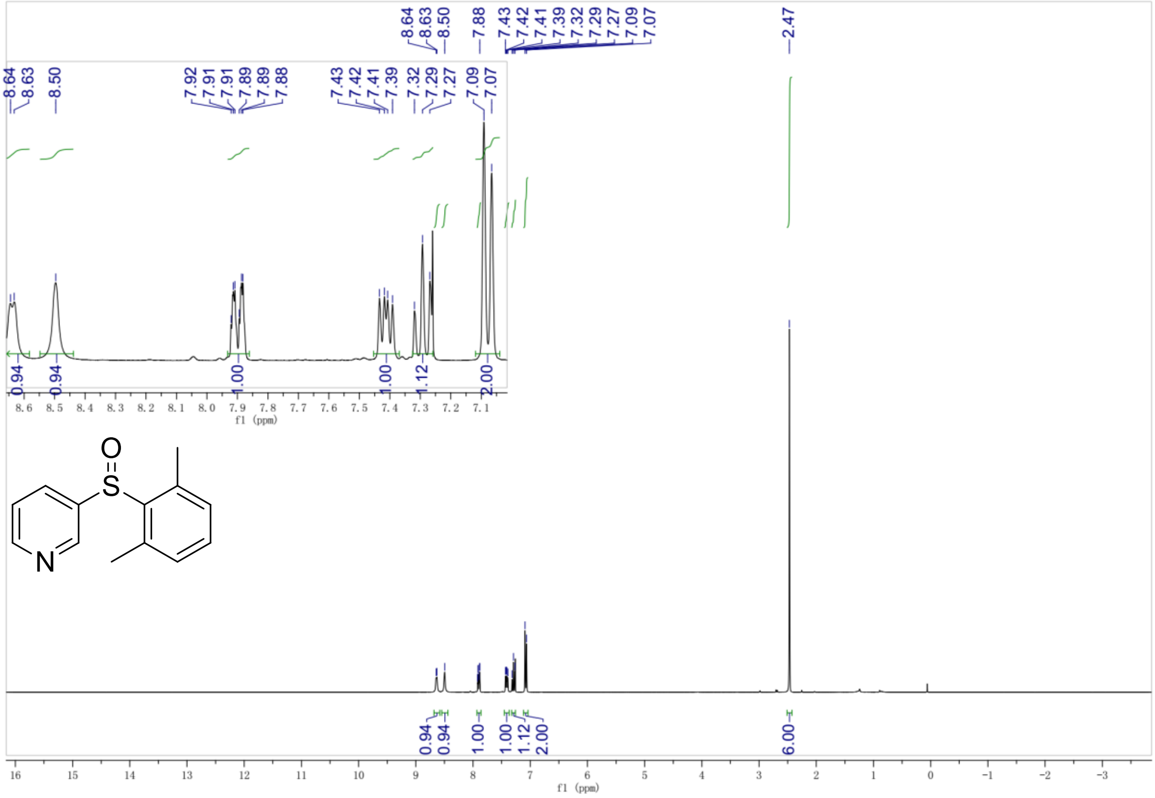

^{1}H NMR spectrum of compound **1w** in $CDCl_3$ (300 MHz).

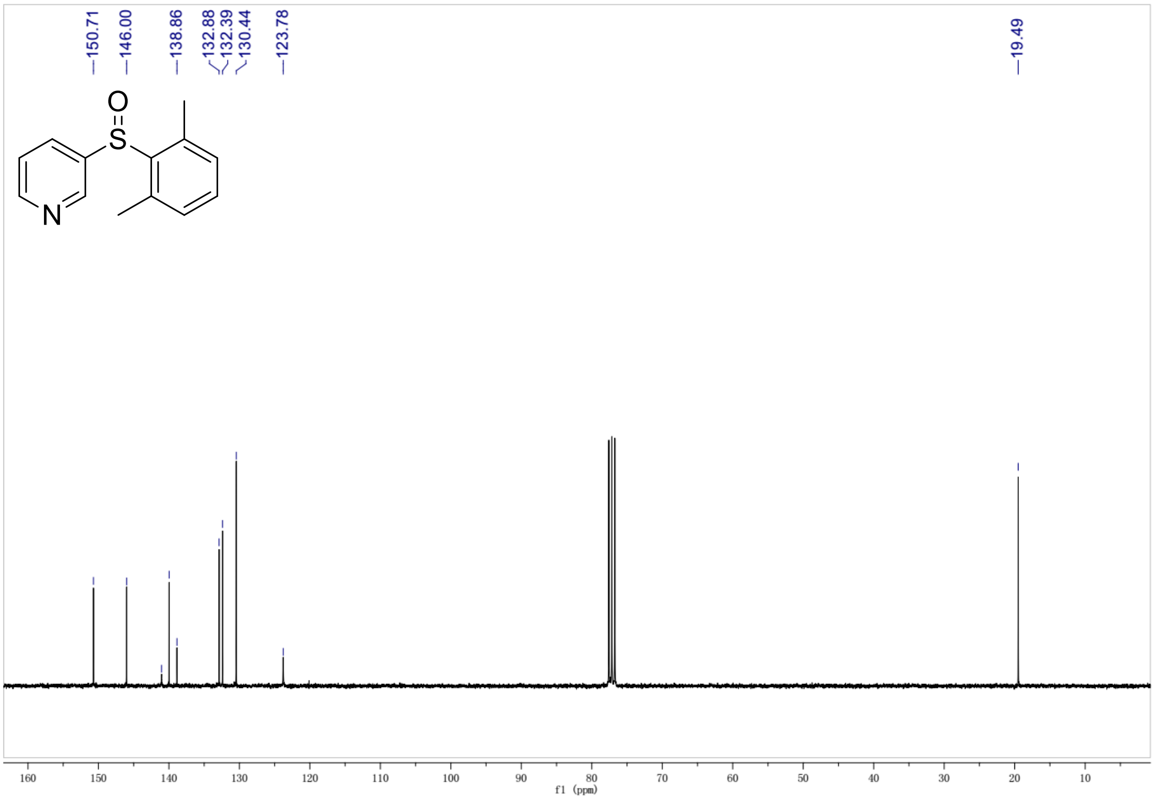

^{13}C{^{1}H} NMR spectrum of compound **1w** in $CDCl_3$ (75 MHz).

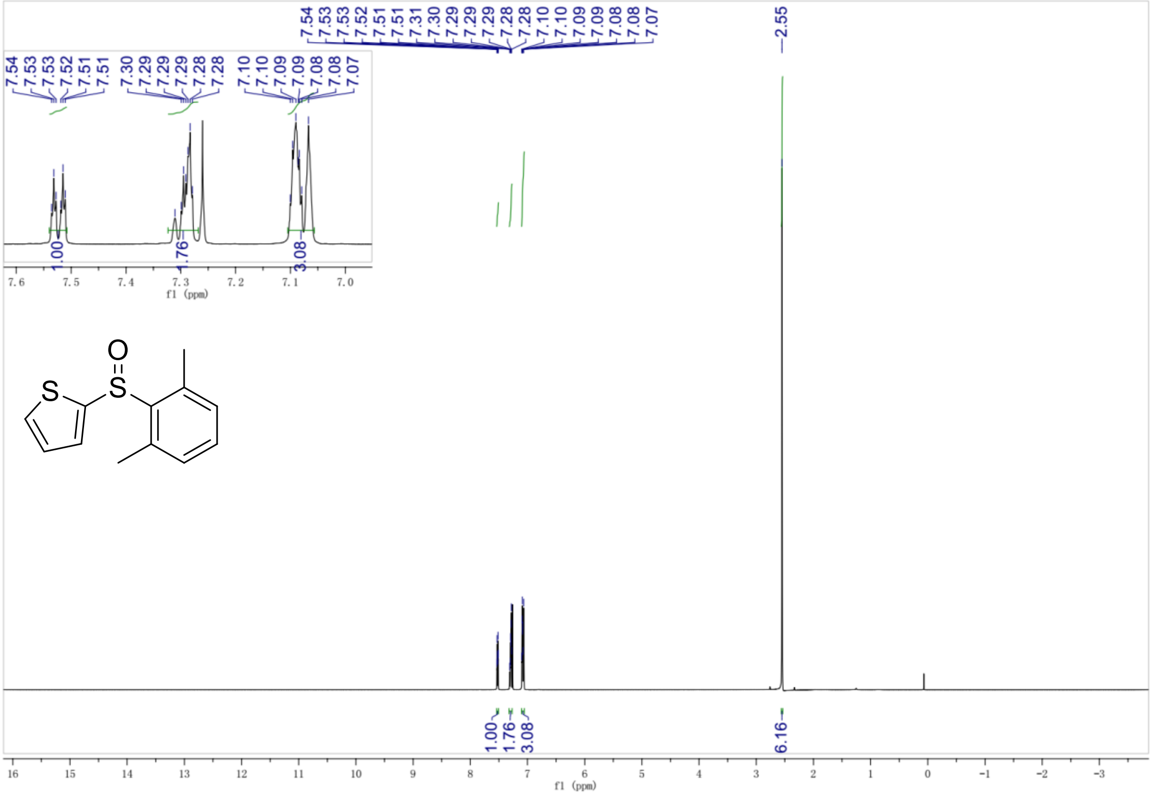

^{1}H NMR spectrum of compound **1x** in $CDCl_3$ (300 MHz).

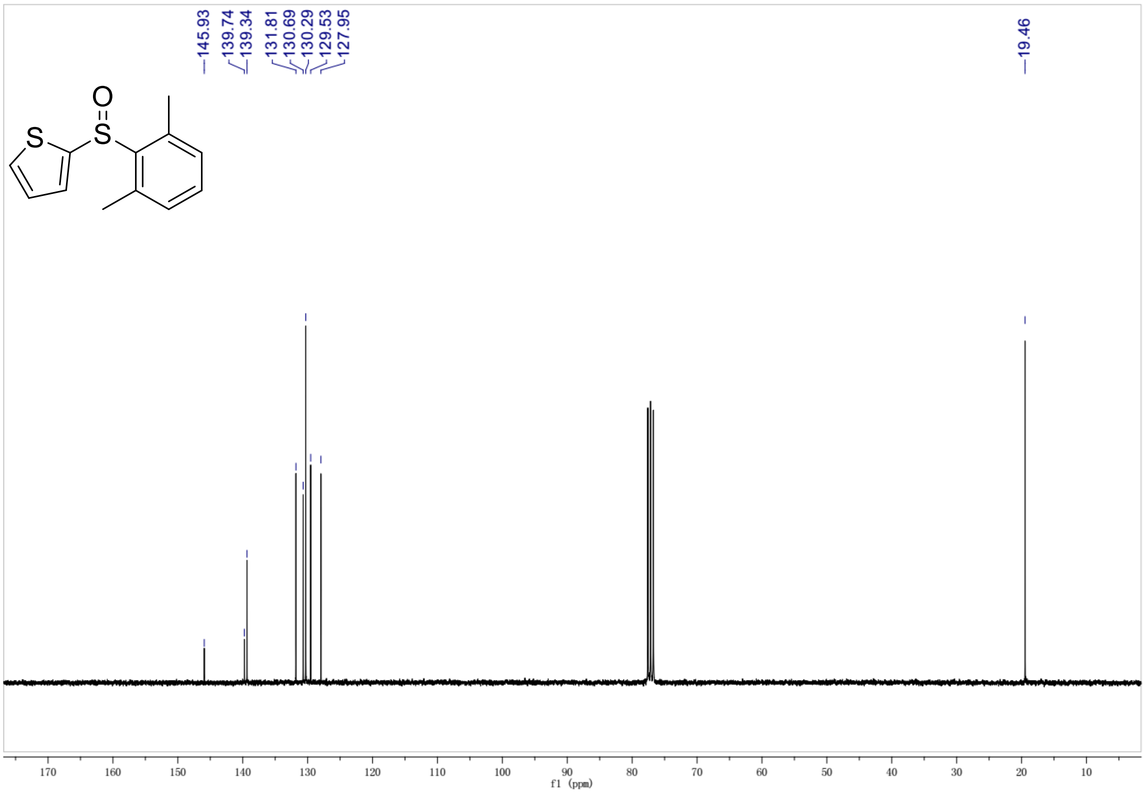

$^{13}C\{^{1}H\}$ NMR spectrum of compound **1x** in $CDCl_3$ (75 MHz).

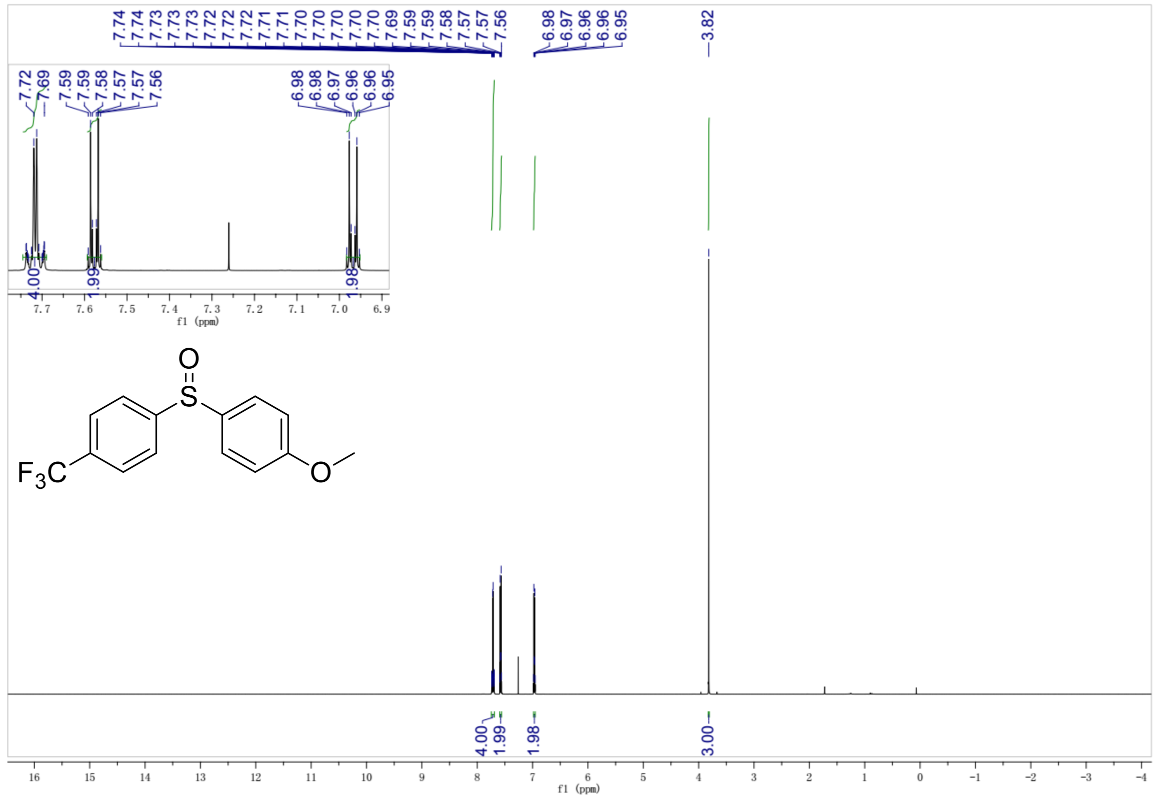

^{1}H NMR spectrum of compound **1y** in $CDCl_3$ (500 MHz).

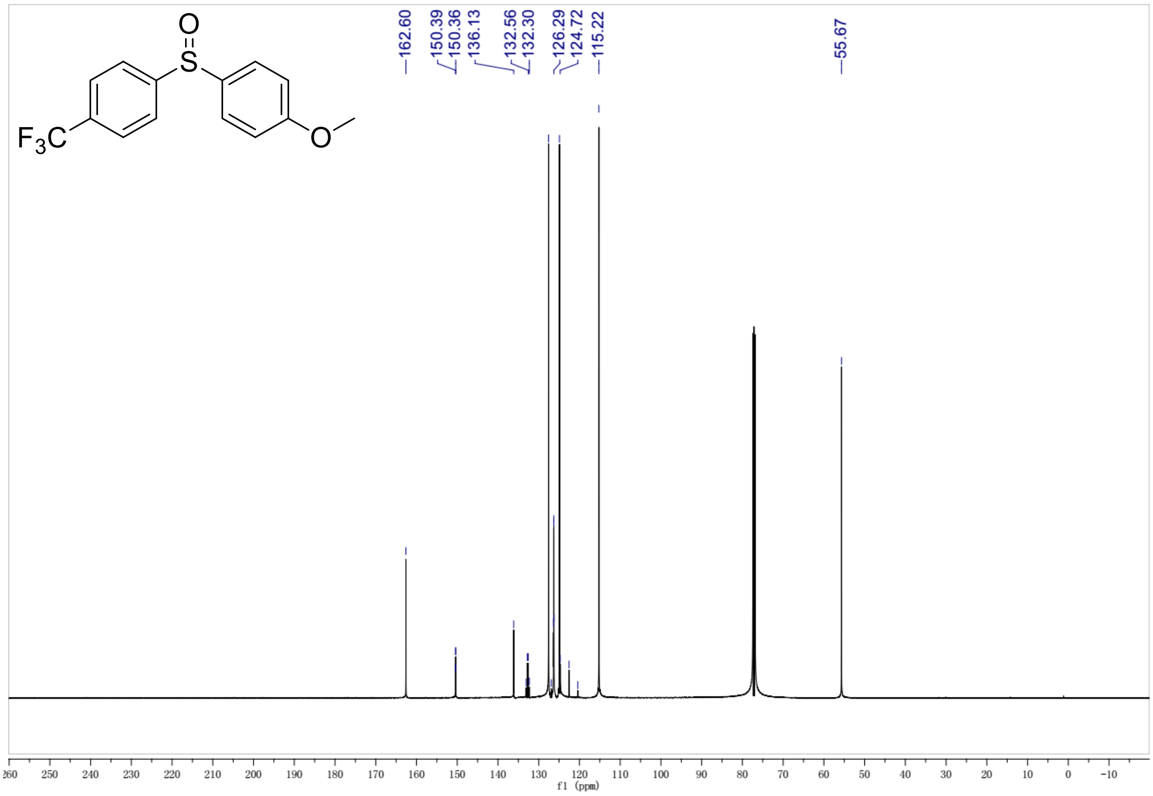

$^{13}C\{^{1}H\}$ NMR spectrum of compound **1y** in $CDCl_3$ (125 MHz).

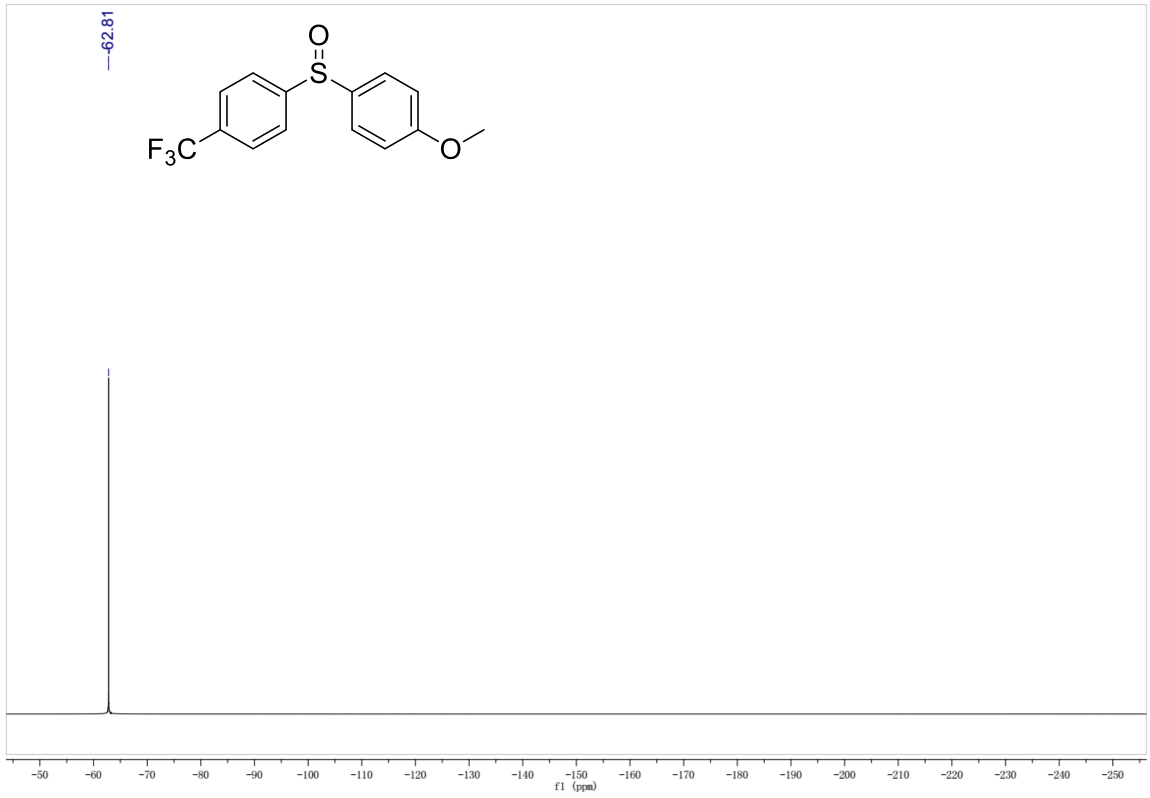

$^{19}F\{^{1}H\}$ NMR spectrum of compound **1y** in $CDCl_3$ (470 MHz).

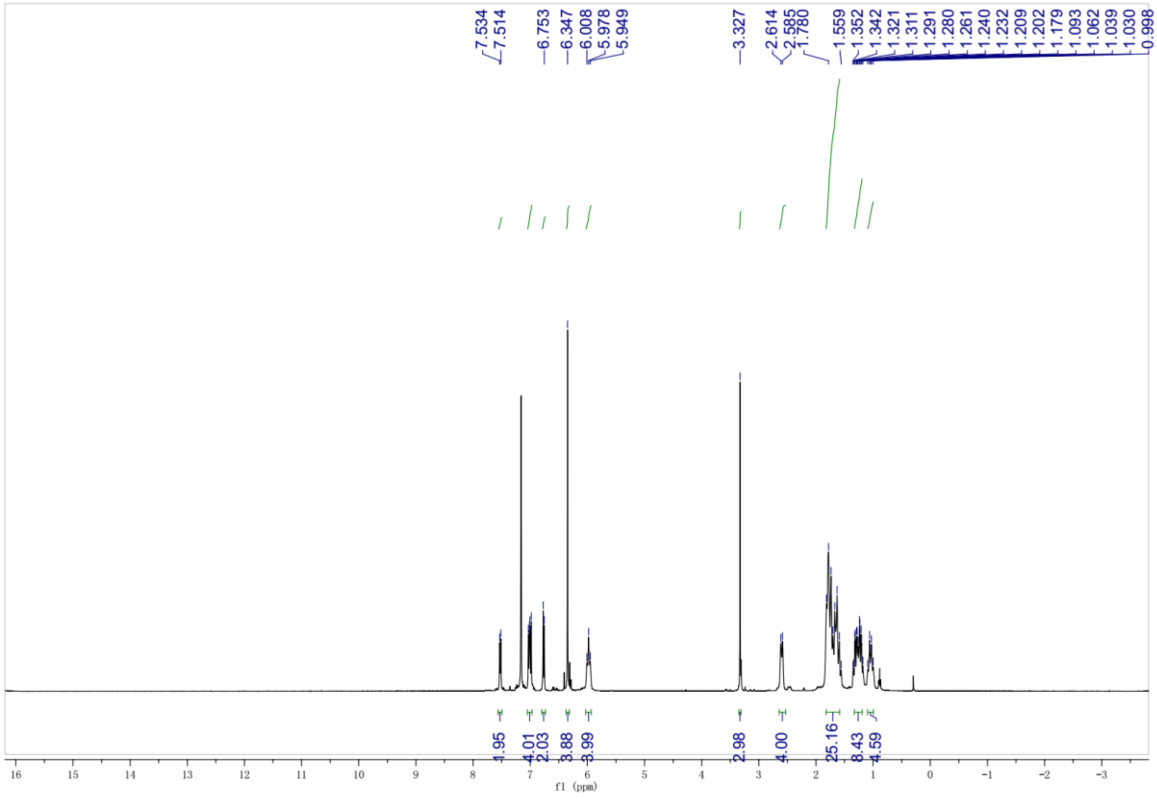

^{1}H NMR spectrum of *trans*-[Ni(ICy)$_2$(4-CF$_3$-C$_6$H$_4$)(SO-{4-MeO-C$_6$H$_4$}] **4** in C_6D_6 (400 MHz).

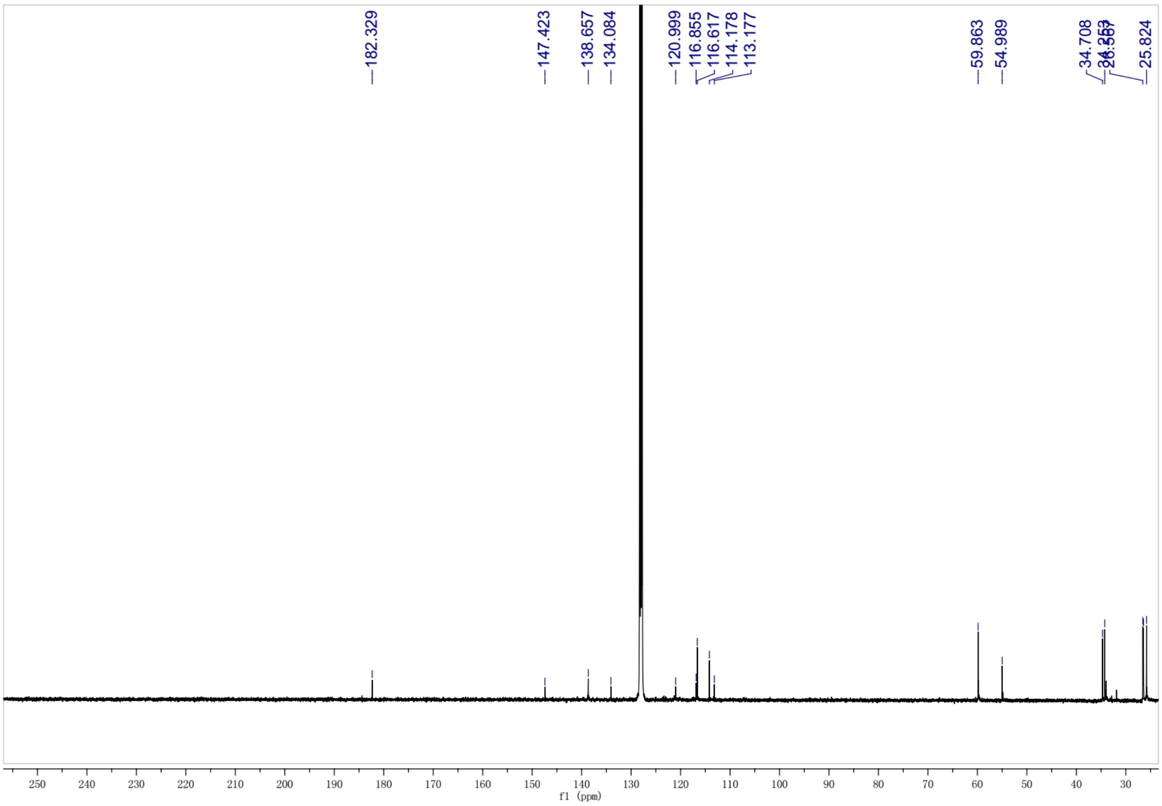

$^{13}C\{^1H\}$ NMR spectrum of *trans*-[Ni(ICy)$_2$(4-CF$_3$-C$_6$H$_4$)(SO-{4-MeO-C$_6$H$_4$}] **4** in C_6D_6 (100 MHz).

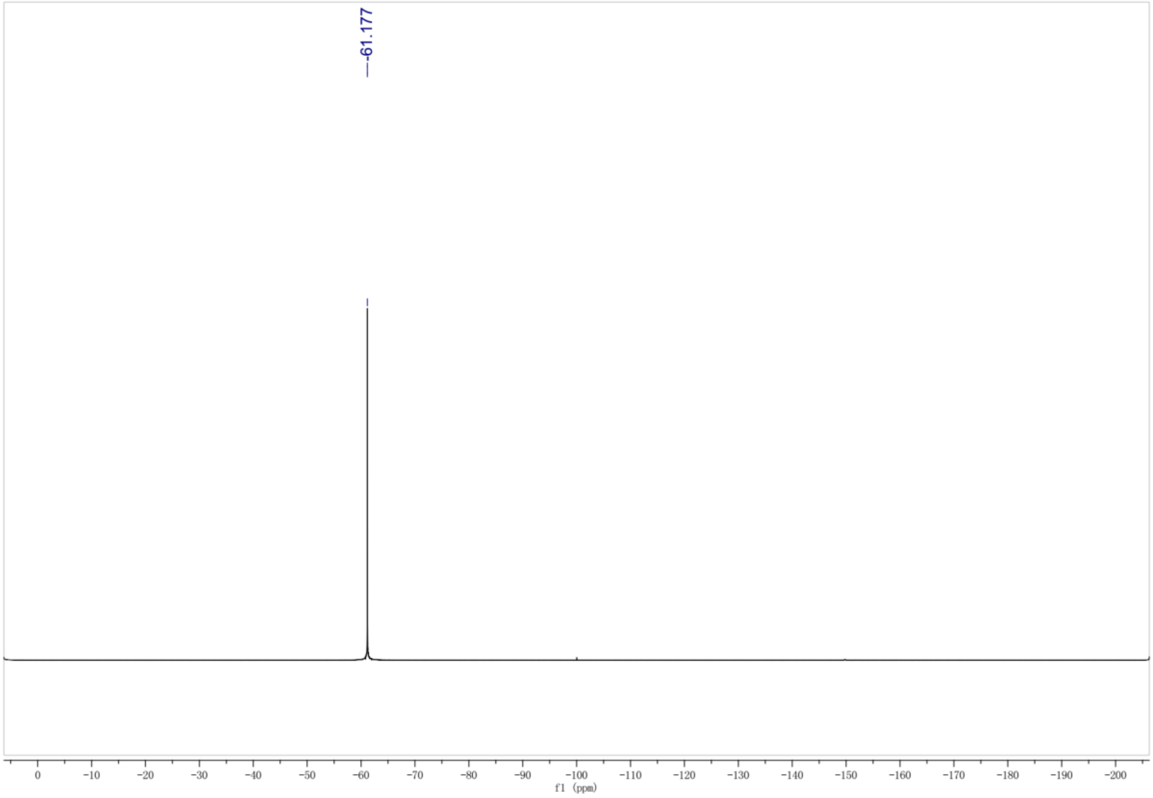

^{19}F NMR spectrum of *trans*-[Ni(ICy)$_2$(4-CF$_3$-C$_6$H$_4$)(SO-{4-MeO-C$_6$H$_4$}] **4** in C_6D_6 (376 MHz).

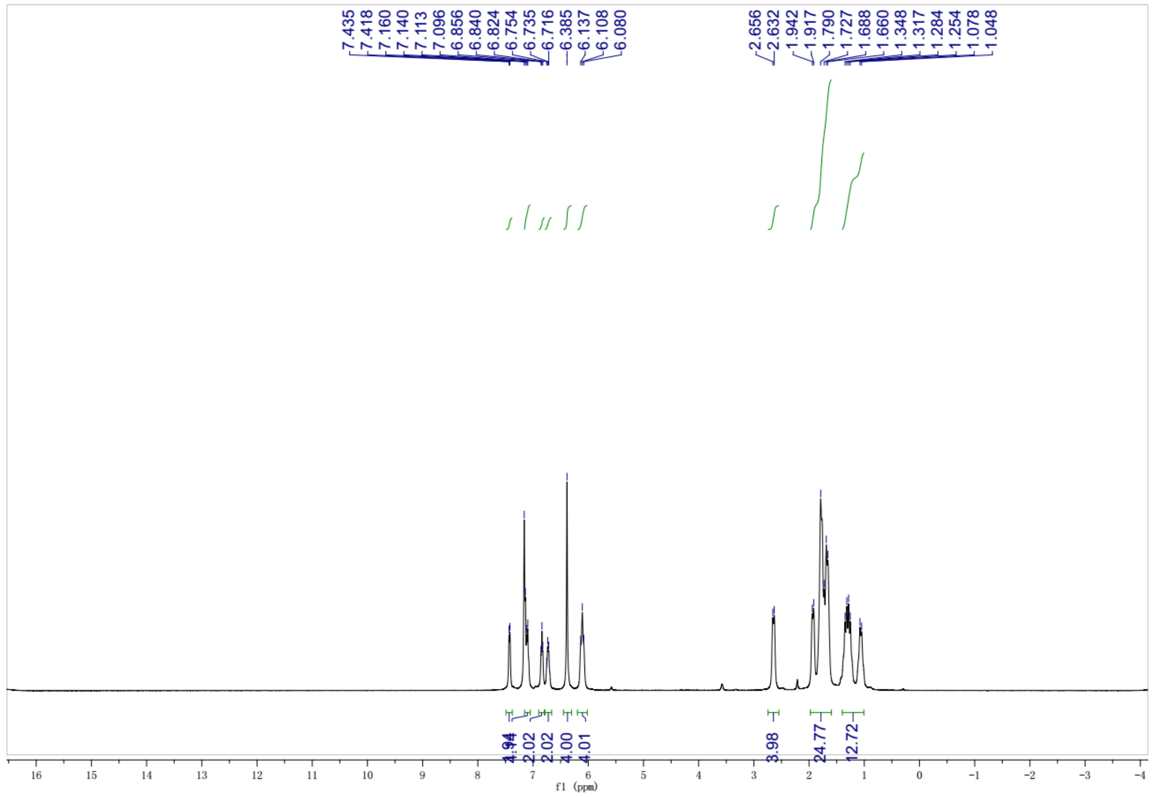

^{1}H NMR spectrum of *trans*-[Ni(ICy)$_2$(C_6H_5)(SO-C_6H_5)] **5** in C_6D_6 (400 MHz).

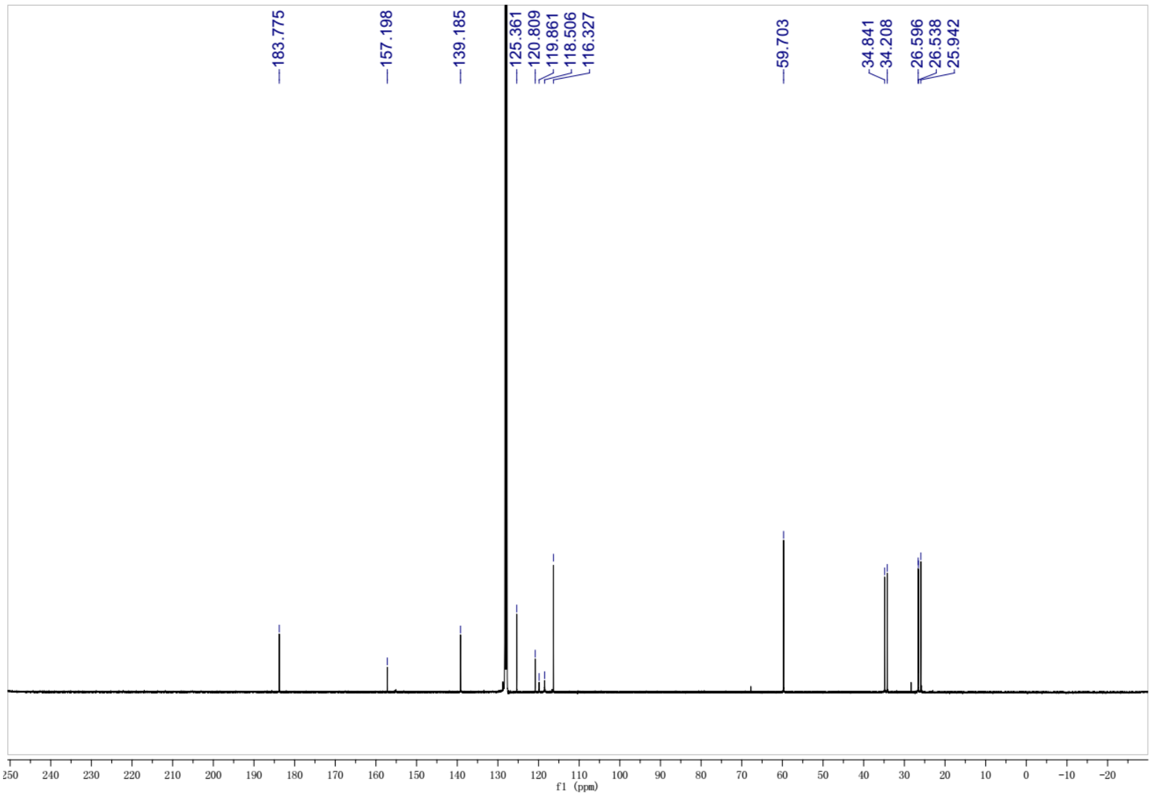

^{13}C{^{1}H} NMR spectrum of *trans*-[Ni(ICy)$_2$(C_6H_5)(SO-C_6H_5)] **5** in C_6D_6 (100 MHz).

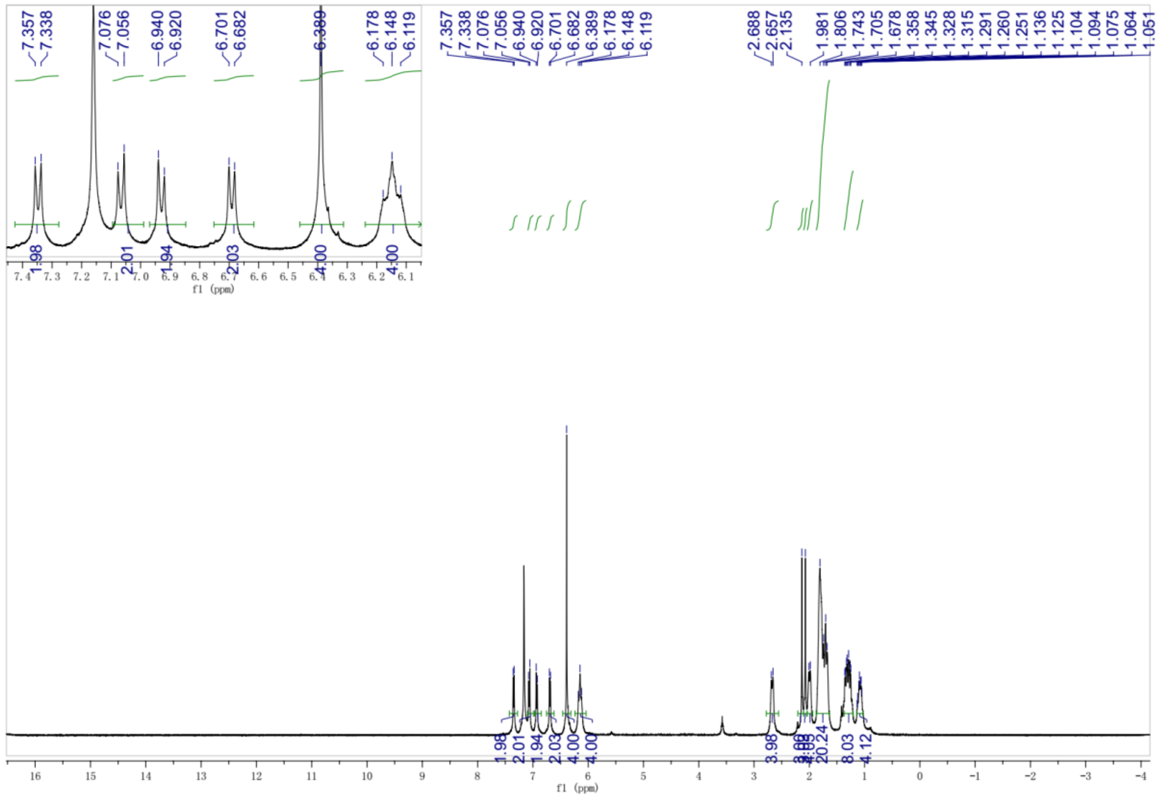

^{1}H NMR spectrum of *trans*-[Ni(ICy)$_2$(4-CH_3-C_6H_4)(SO-{4-CH_3-C_6H_4}] **6** in C_6D_6 (400 MHz).

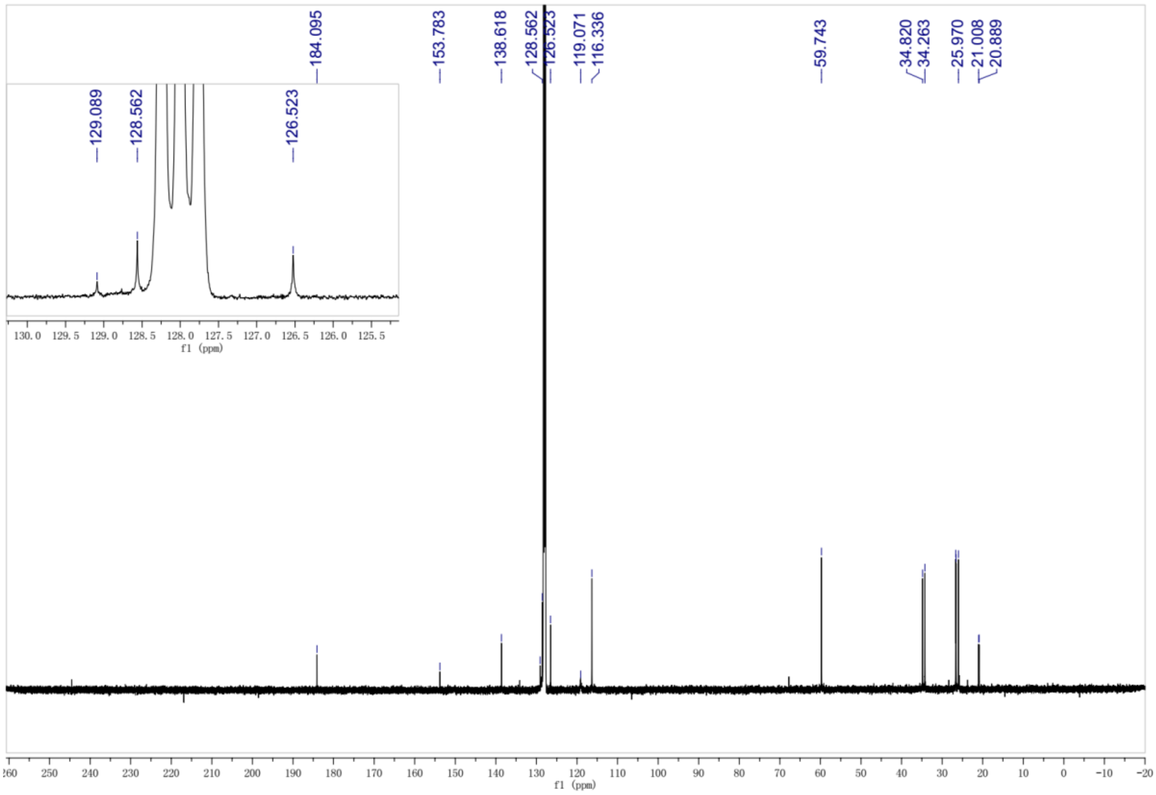

$^{13}C\{^{1}H\}$ NMR spectrum of *trans*-[Ni(ICy)$_2$(4-CH_3-C_6H_4)(SO-{4-CH_3-C_6H_4}] **6** in C_6D_6 (100 MHz).

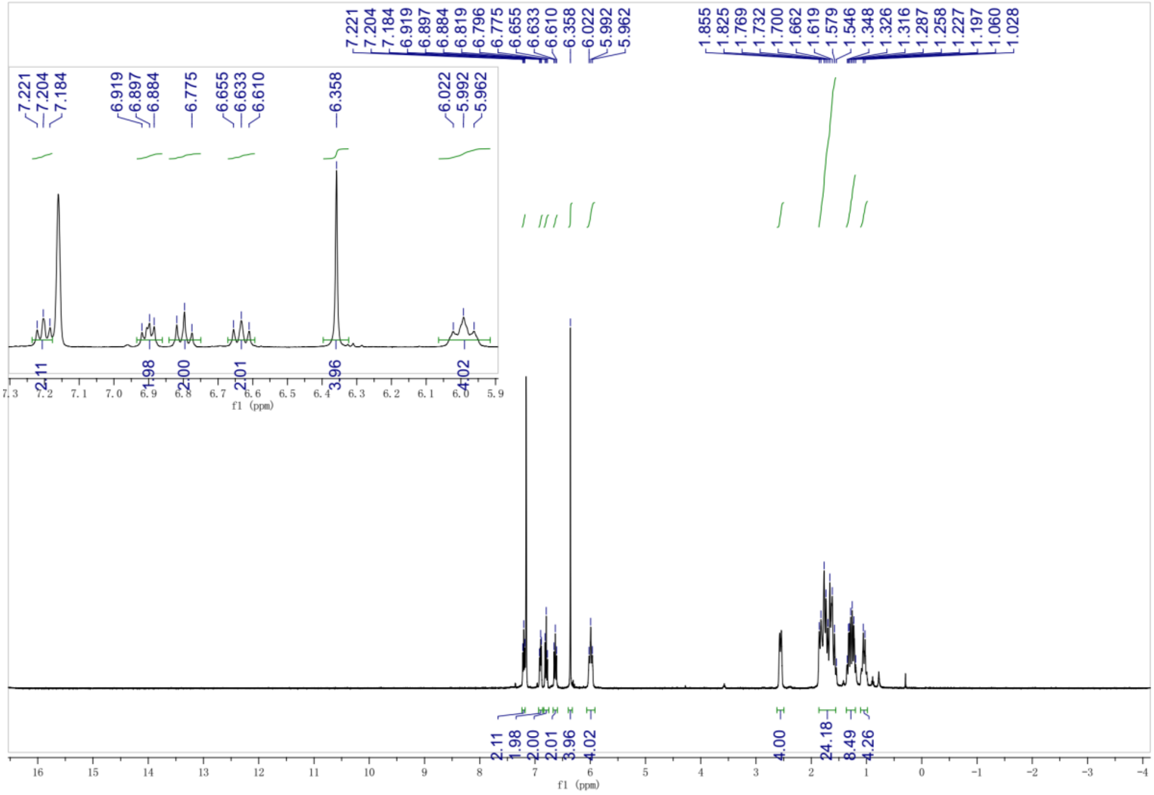

^{1}H NMR spectrum of *trans*-[Ni(ICy)$_2$(4-F-C_6H_4)(SO-{4-F-C_6H_4}] **7** in C_6D_6 (400 MHz).

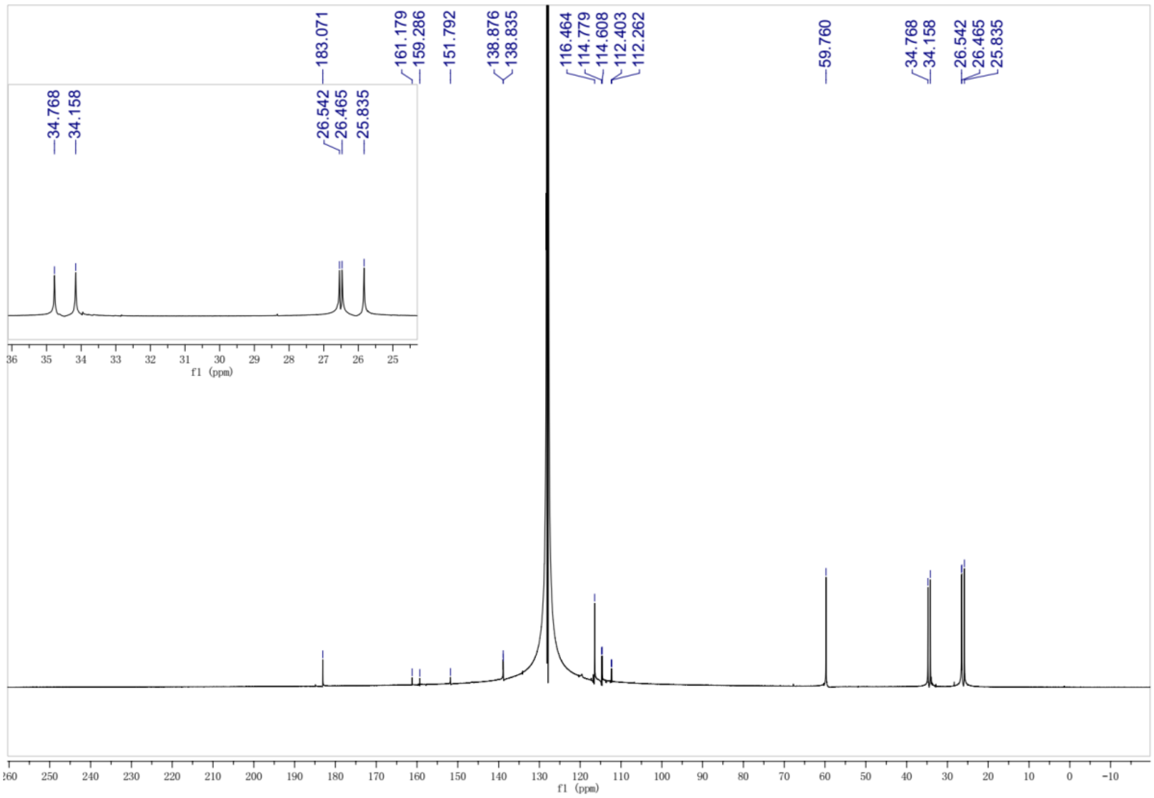

$^{13}C\{^{1}H\}$ NMR spectrum of *trans*-[Ni(ICy)$_2$(4-F-C_6H_4)(SO-{4-F-C_6H_4}] **7** in C_6D_6 (100 MHz).

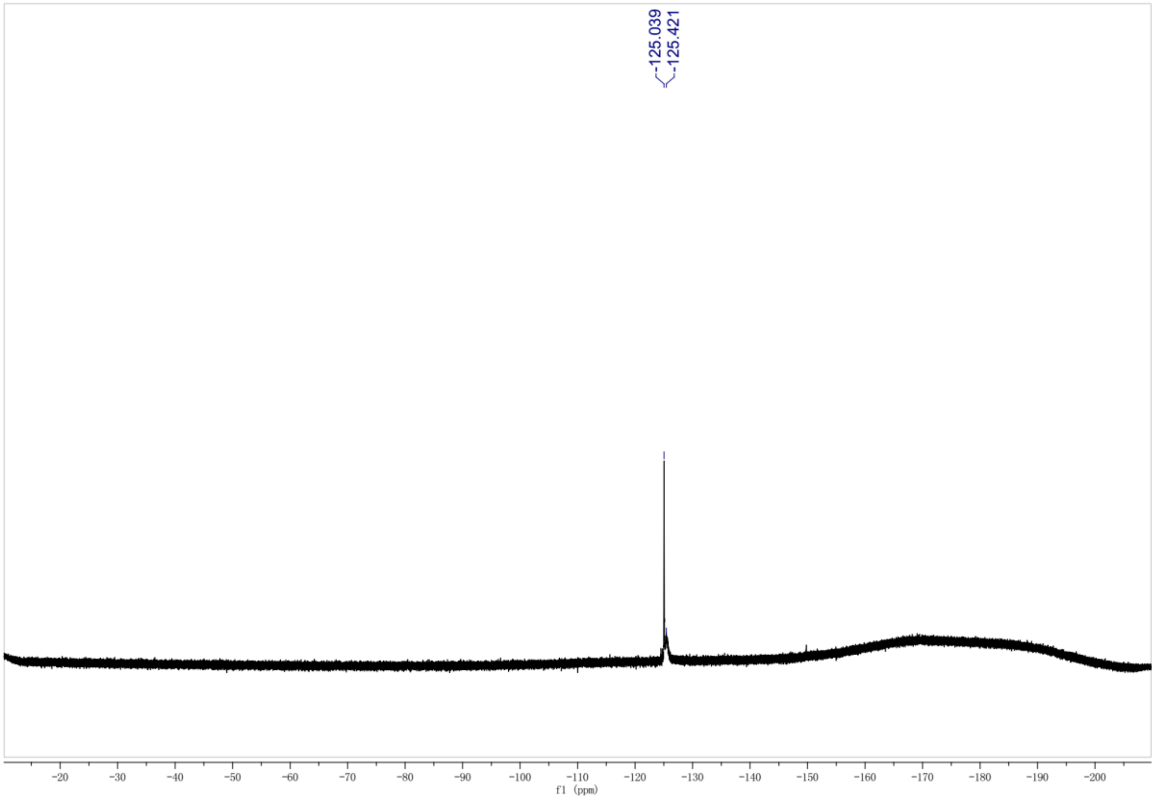

^{19}F NMR spectrum of *trans*-[Ni(ICy)$_2$(4-F-C_6H_4)(SO-{4-F-C_6H_4}] **7** in C_6D_6 (376 MHz).

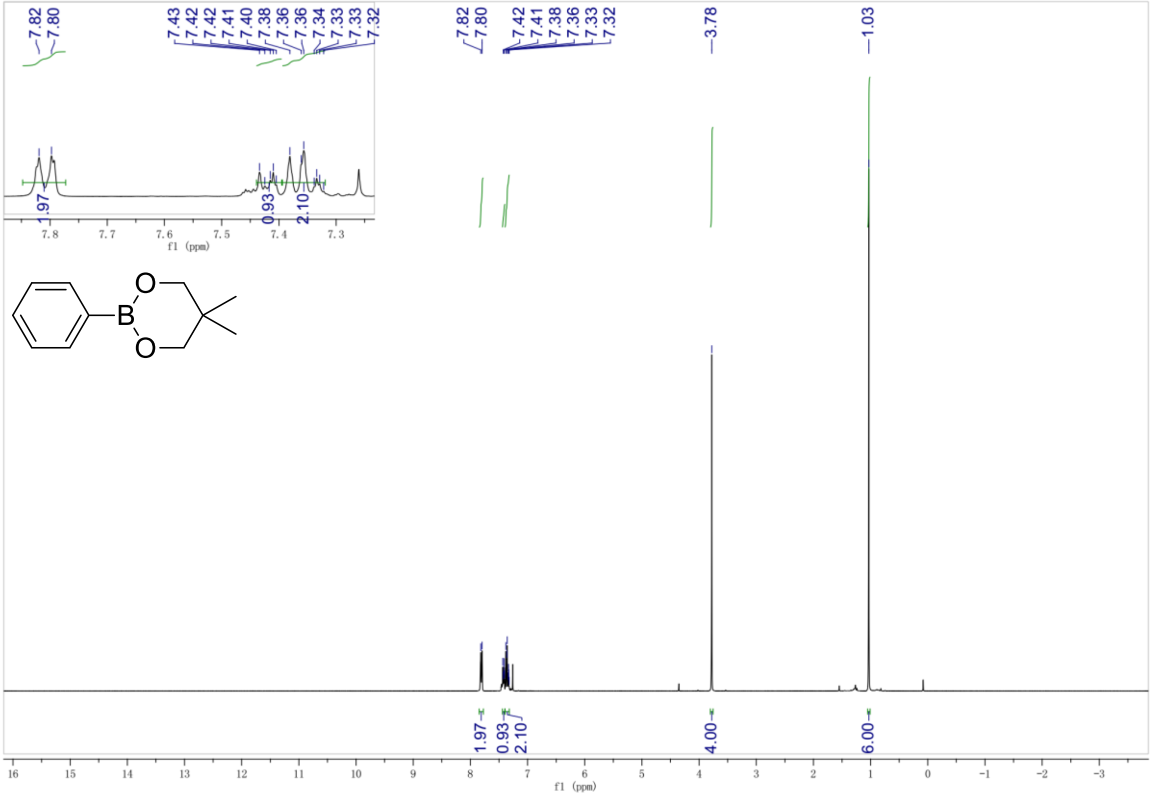

^{1}H NMR spectrum of compound **3a** in $CDCl_3$ (300 MHz).

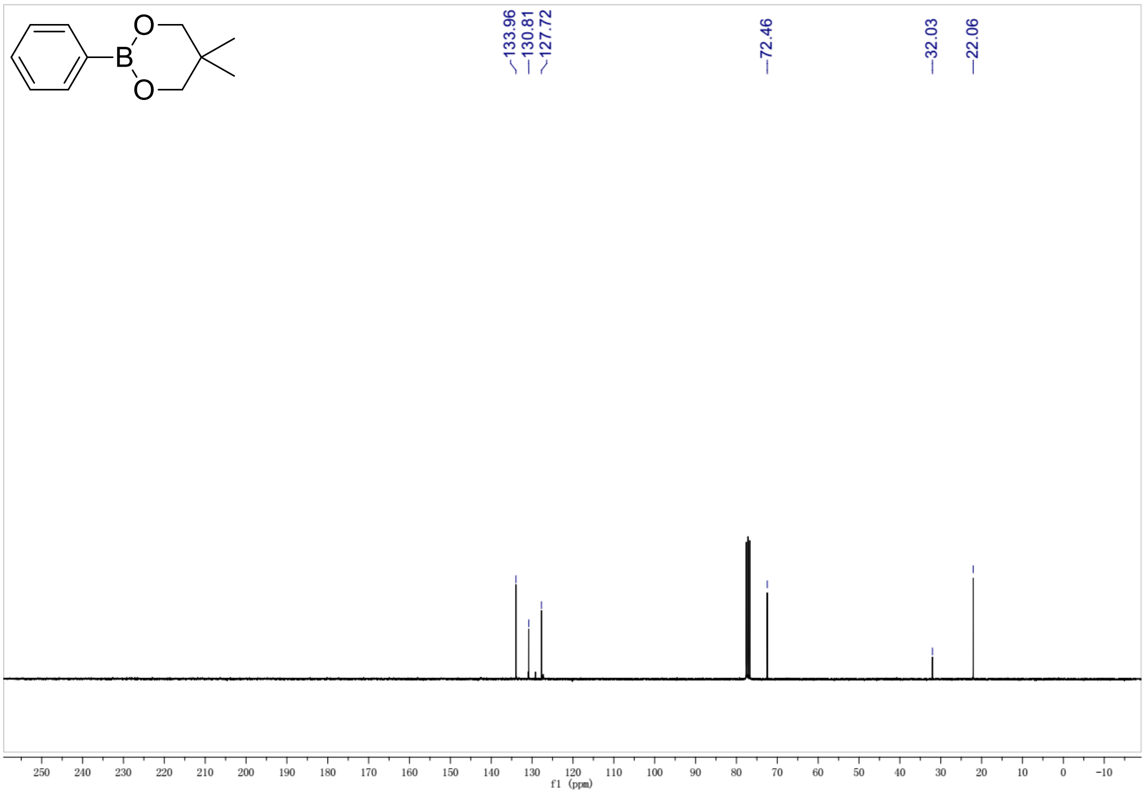

$^{13}C\{^{1}H\}$ NMR spectrum of compound **3a** in $CDCl_3$ (75 MHz).

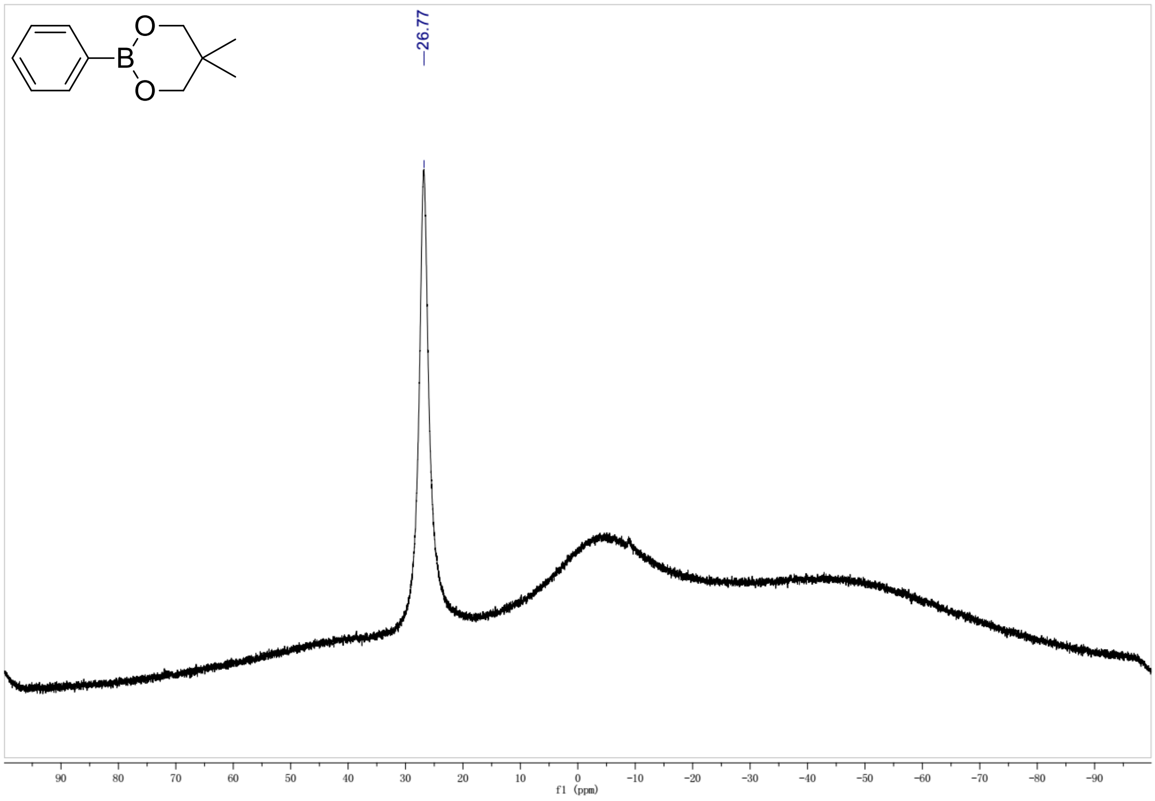

$^{11}B\{^{1}H\}$ NMR spectrum of compound **3a** in $CDCl_3$ (96 MHz).

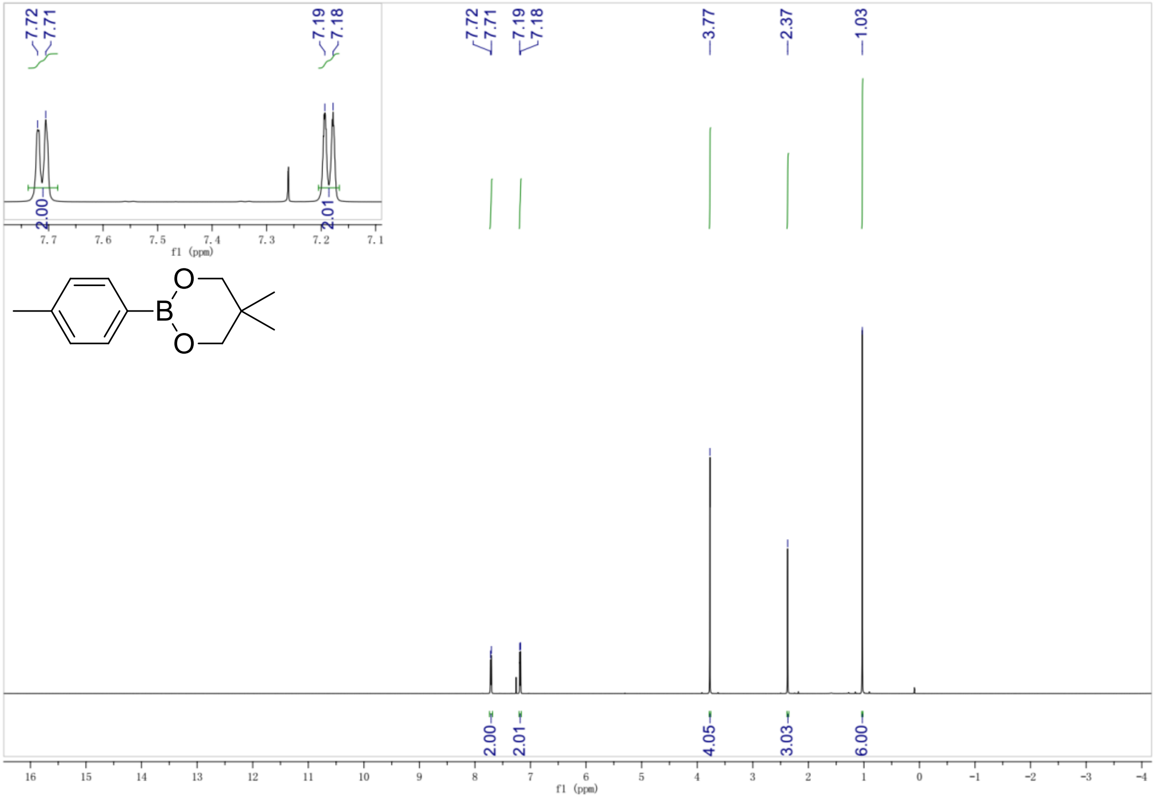

^{1}H NMR spectrum of compound **3b** in $CDCl_3$ (500 MHz).

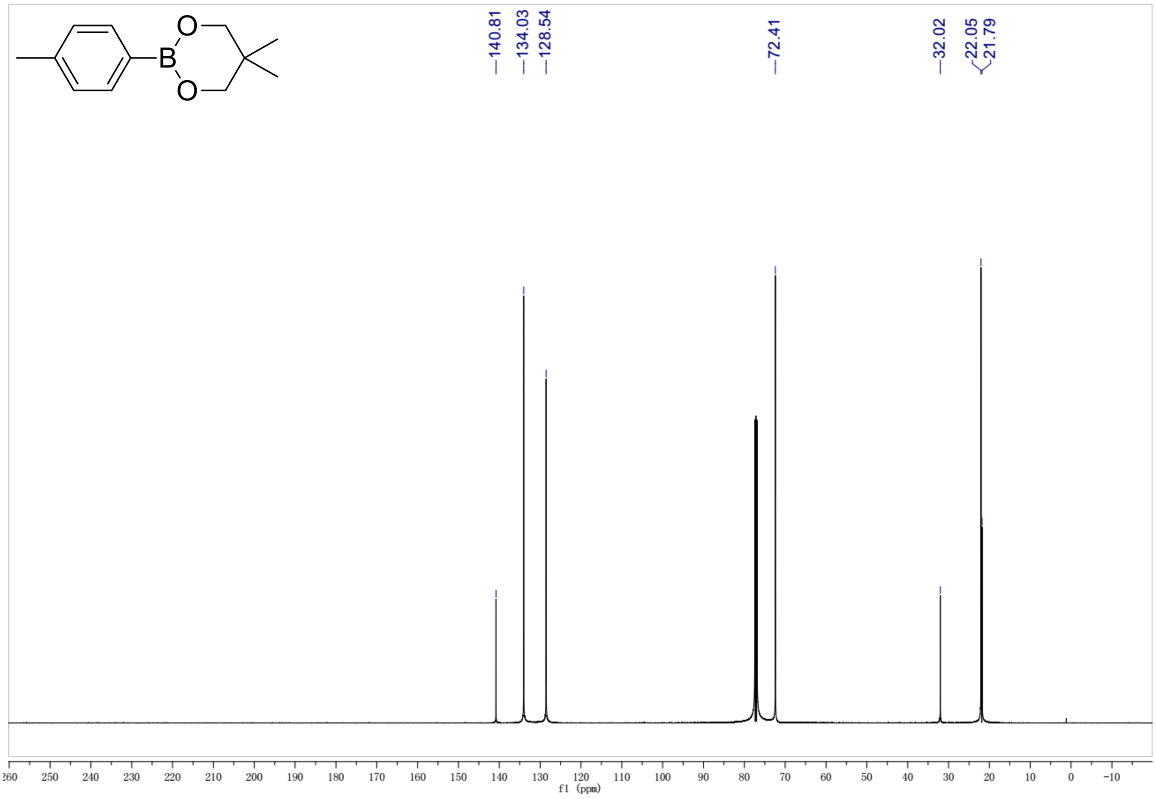

$^{13}C\{^1H\}$ NMR spectrum of compound **3b** in $CDCl_3$ (125 MHz).

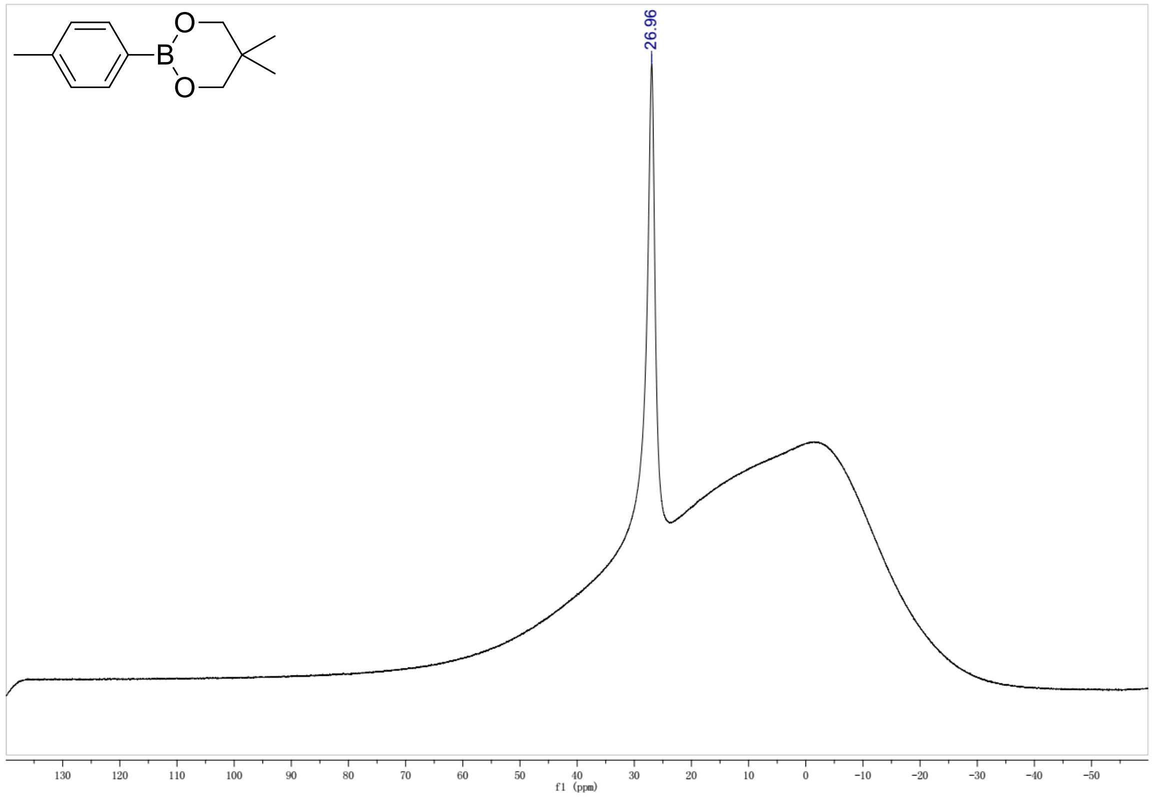

$^{11}B\{^1H\}$ NMR spectrum of compound **3b** in $CDCl_3$ (160 MHz).

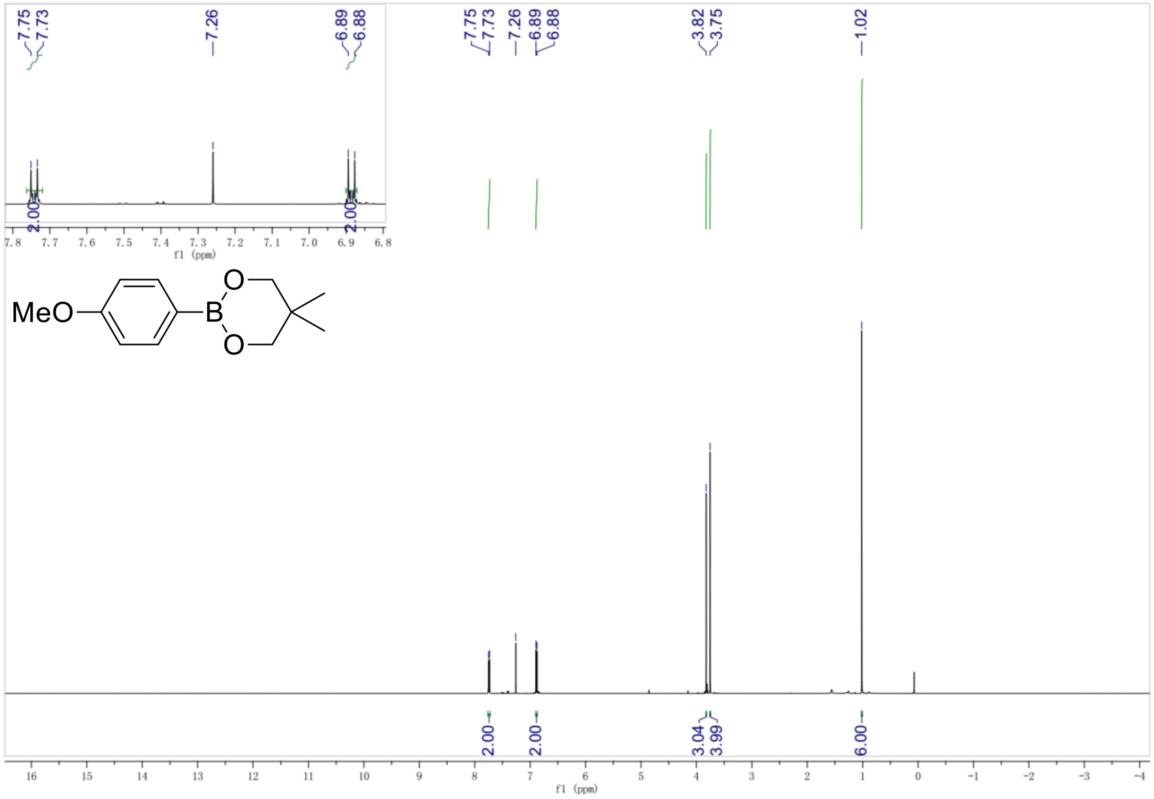

^1H NMR spectrum of compound **3c** in $CDCl_3$ (500 MHz).

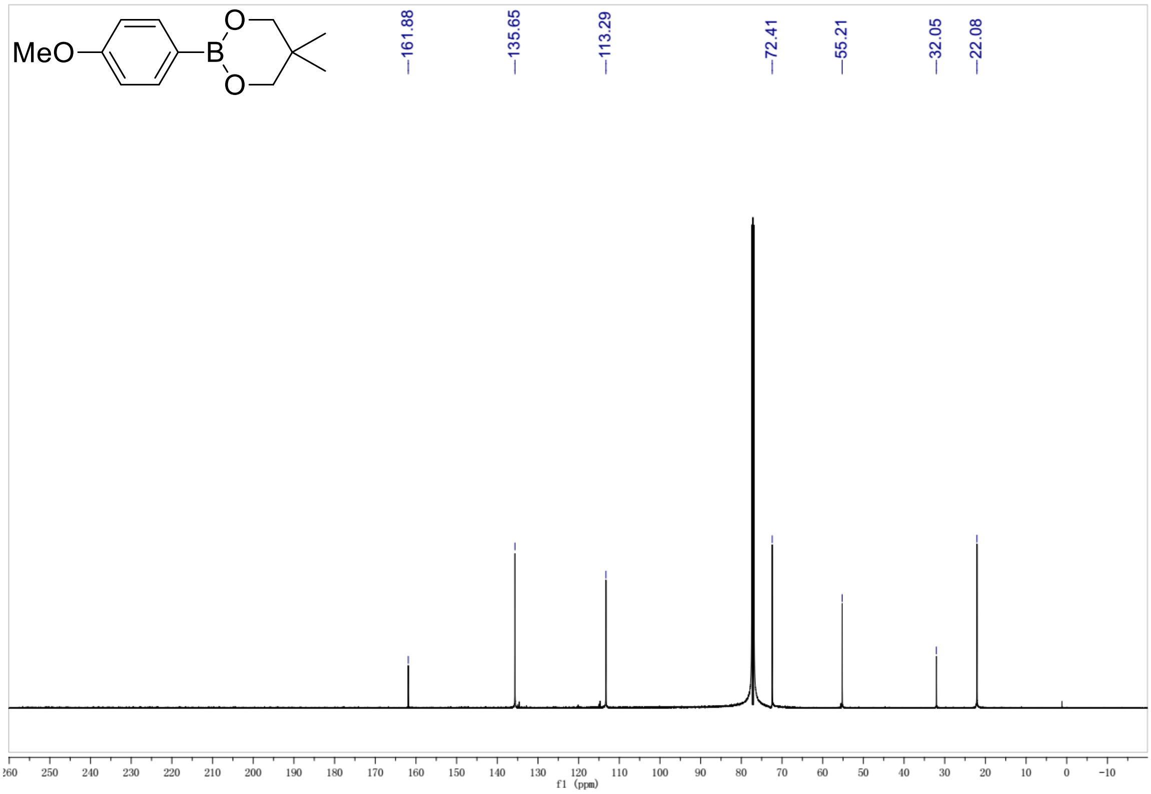

$^{13}C\{^1H\}$ NMR spectrum of compound **3c** in $CDCl_3$ (125 MHz).

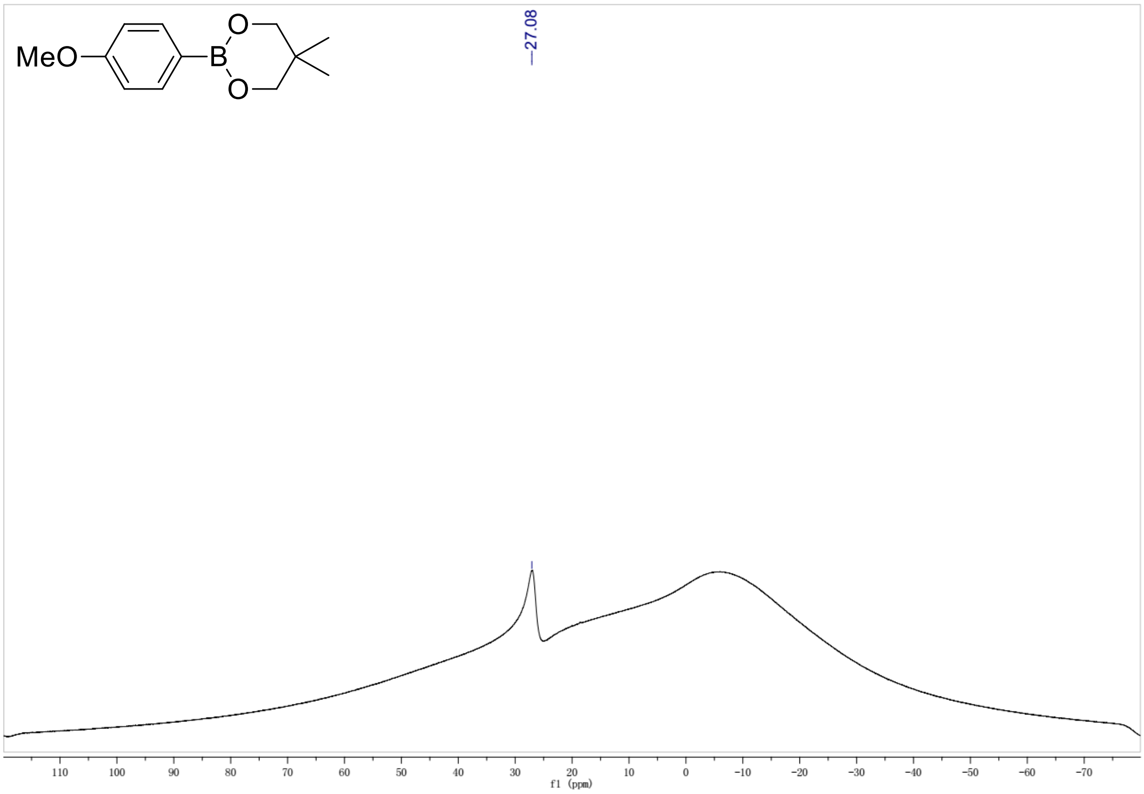

$^{11}B\{^{1}H\}$ NMR spectrum of compound **3c** in $CDCl_3$ (160 MHz).

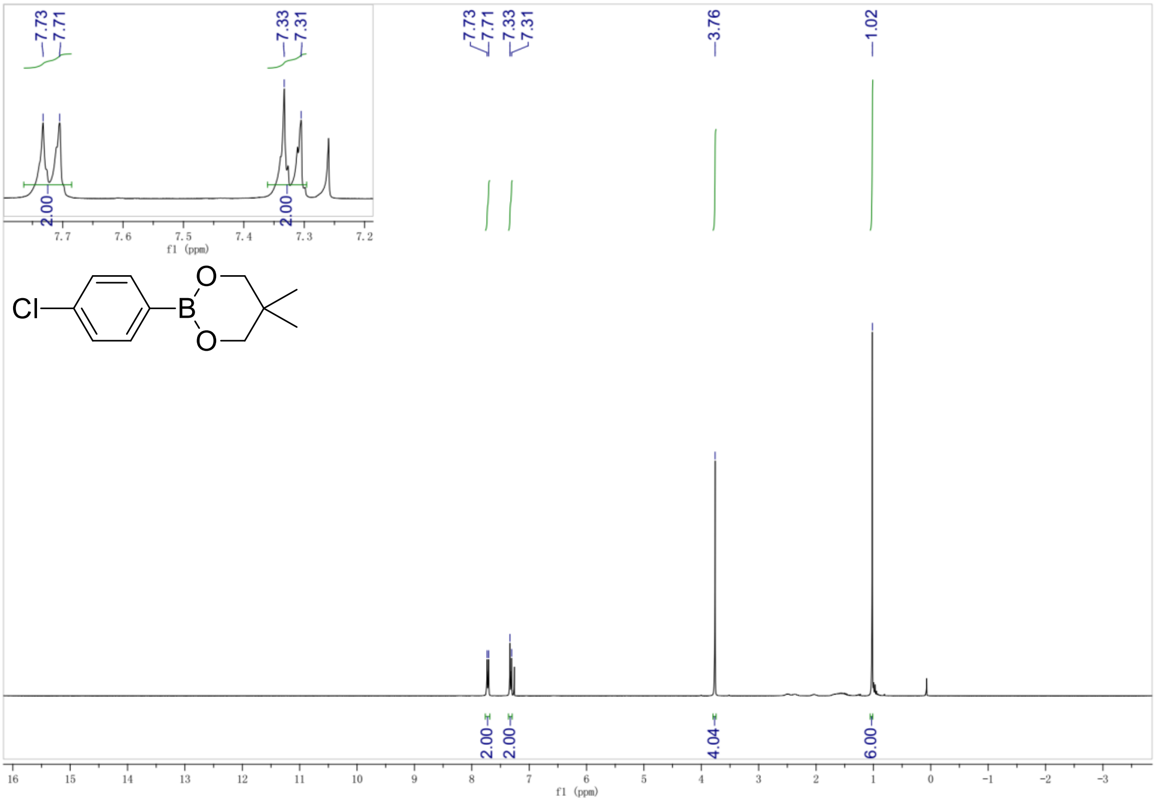

^{1}H NMR spectrum of compound **3d** in $CDCl_3$ (300 MHz).

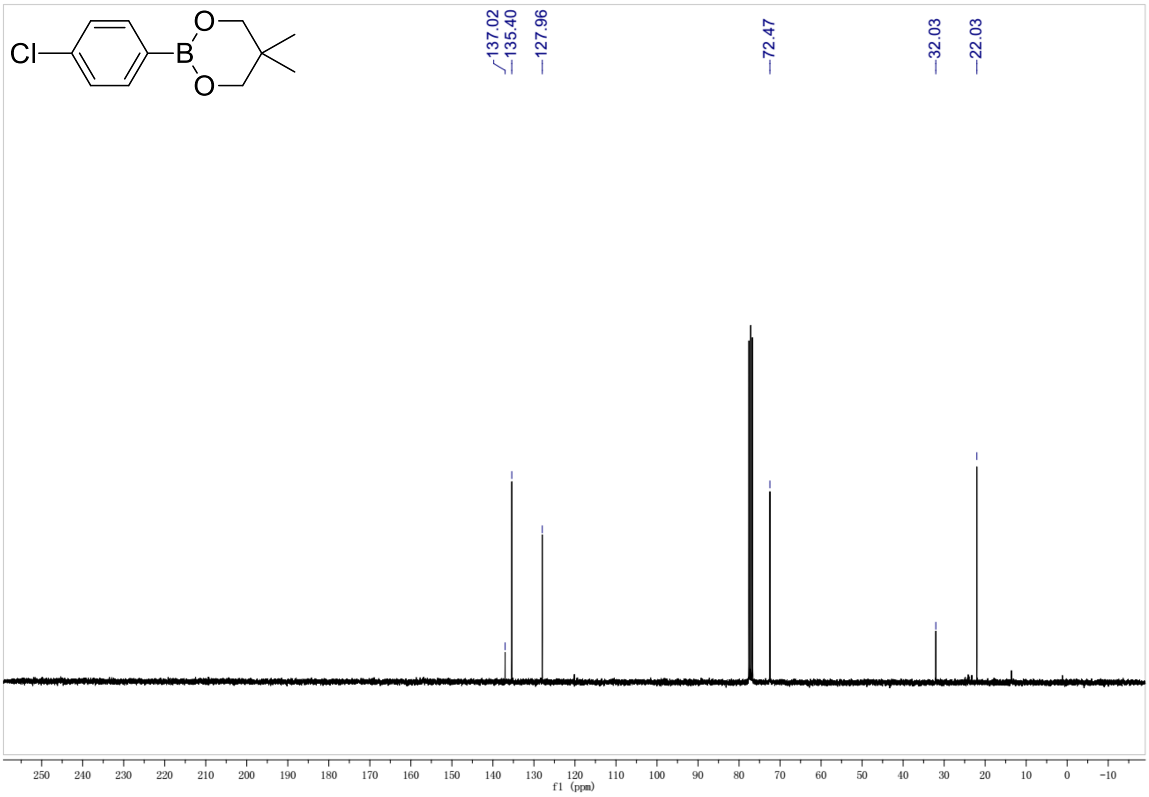

$^{13}C\{^{1}H\}$ NMR spectrum of compound **3d** in $CDCl_3$ (75 MHz).

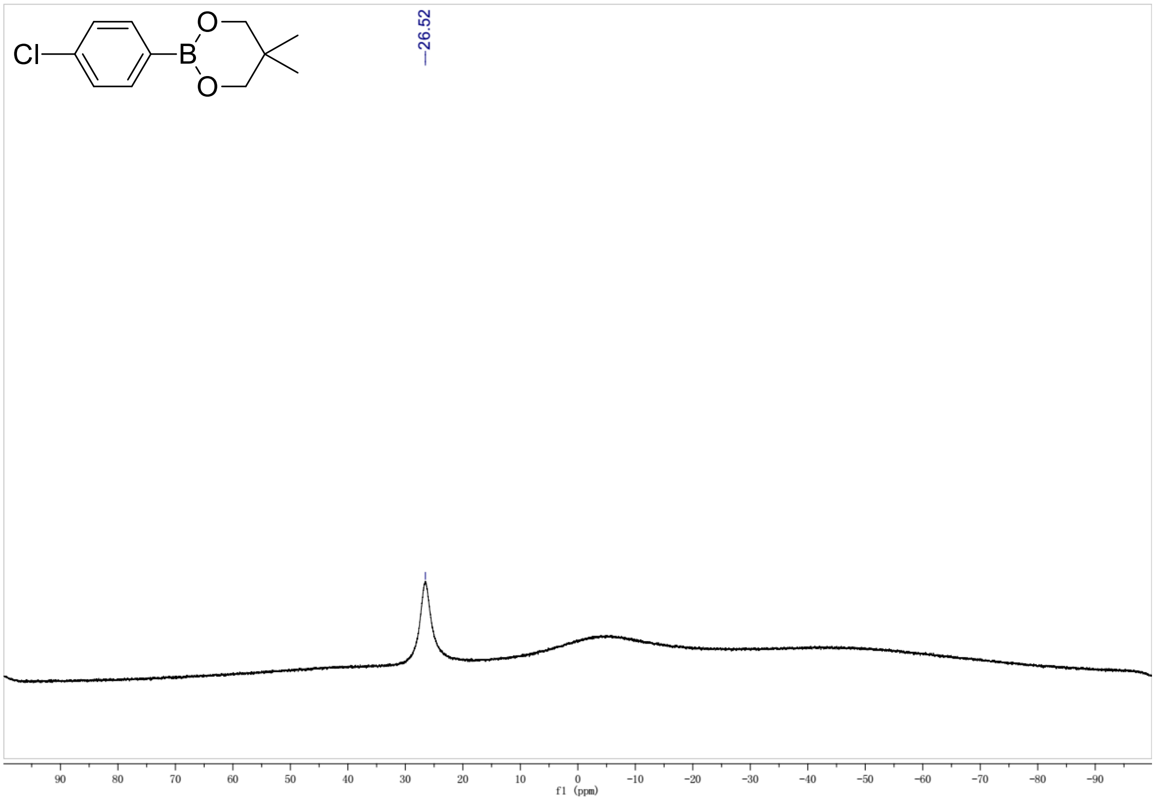

$^{11}B\{^{1}H\}$ NMR spectrum of compound **3d** in $CDCl_3$ (96 MHz).

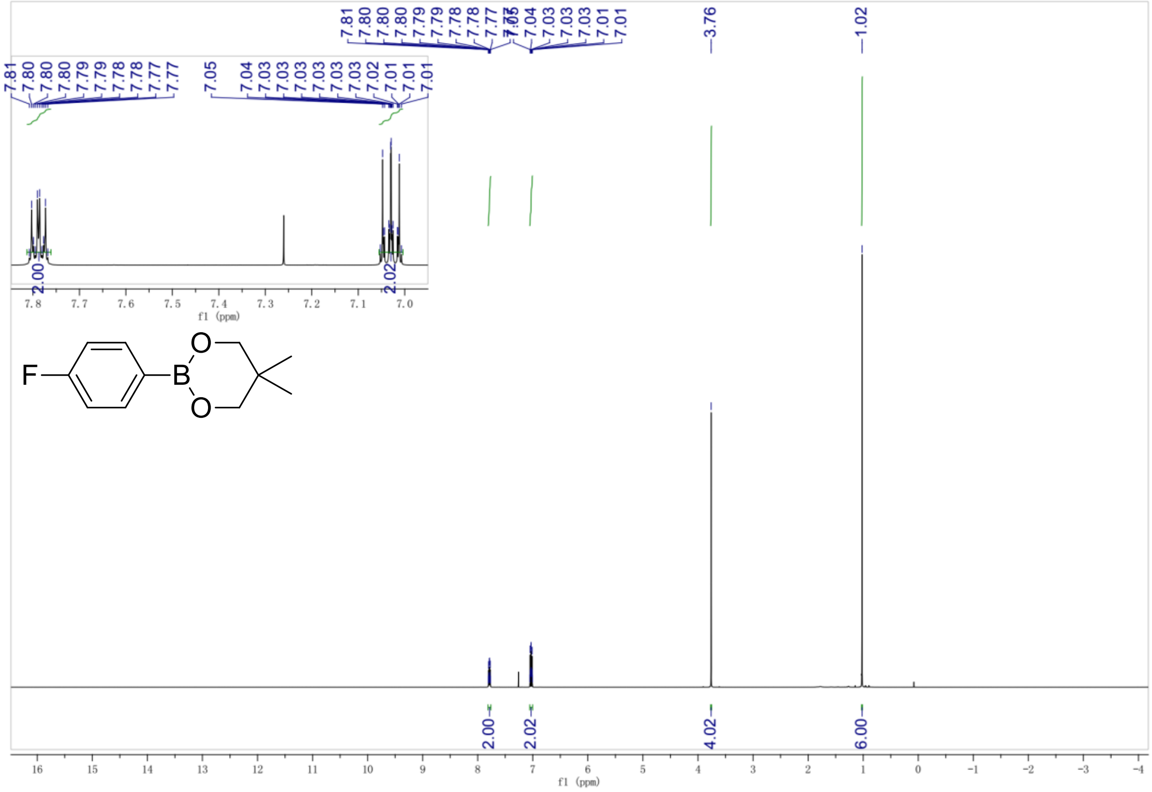

^{1}H NMR spectrum of compound **3e** in $CDCl_3$ (500 MHz).

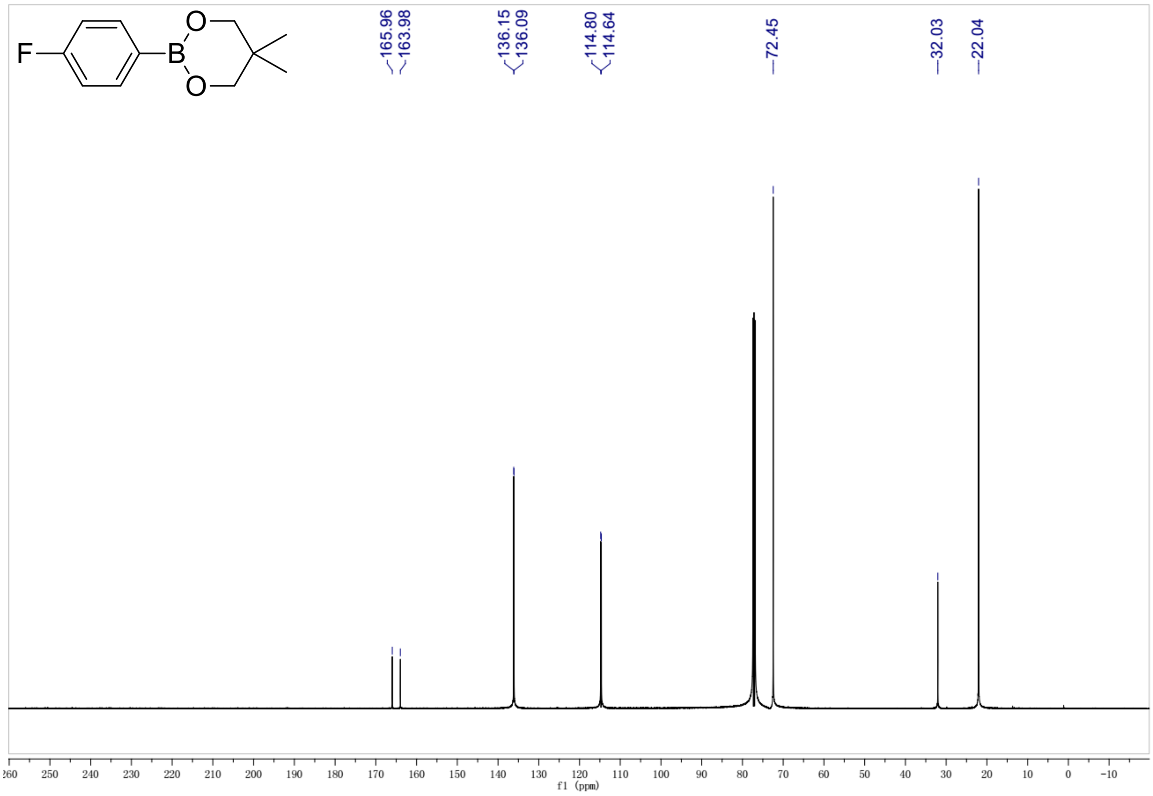

$^{13}C\{^{1}H\}$ NMR spectrum of compound **3e** in $CDCl_3$ (125 MHz).

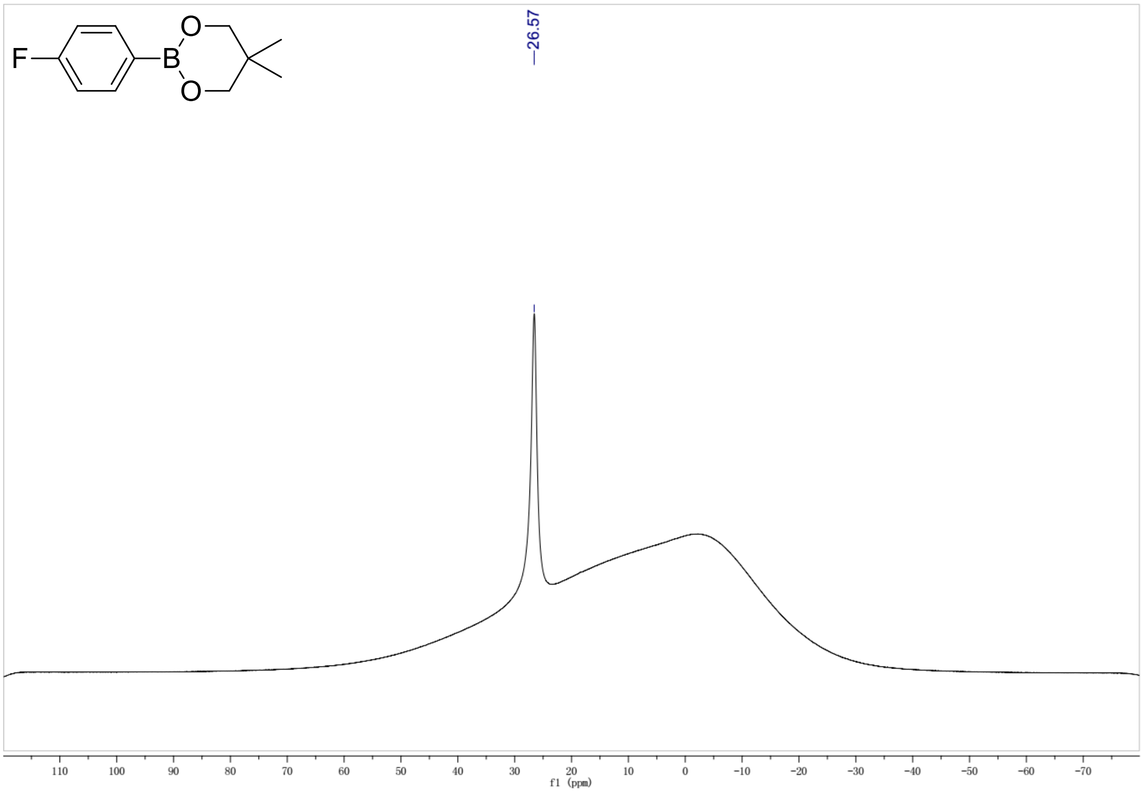

$^{11}B\{^1H\}$ NMR spectrum of compound **3e** in $CDCl_3$ (160 MHz).

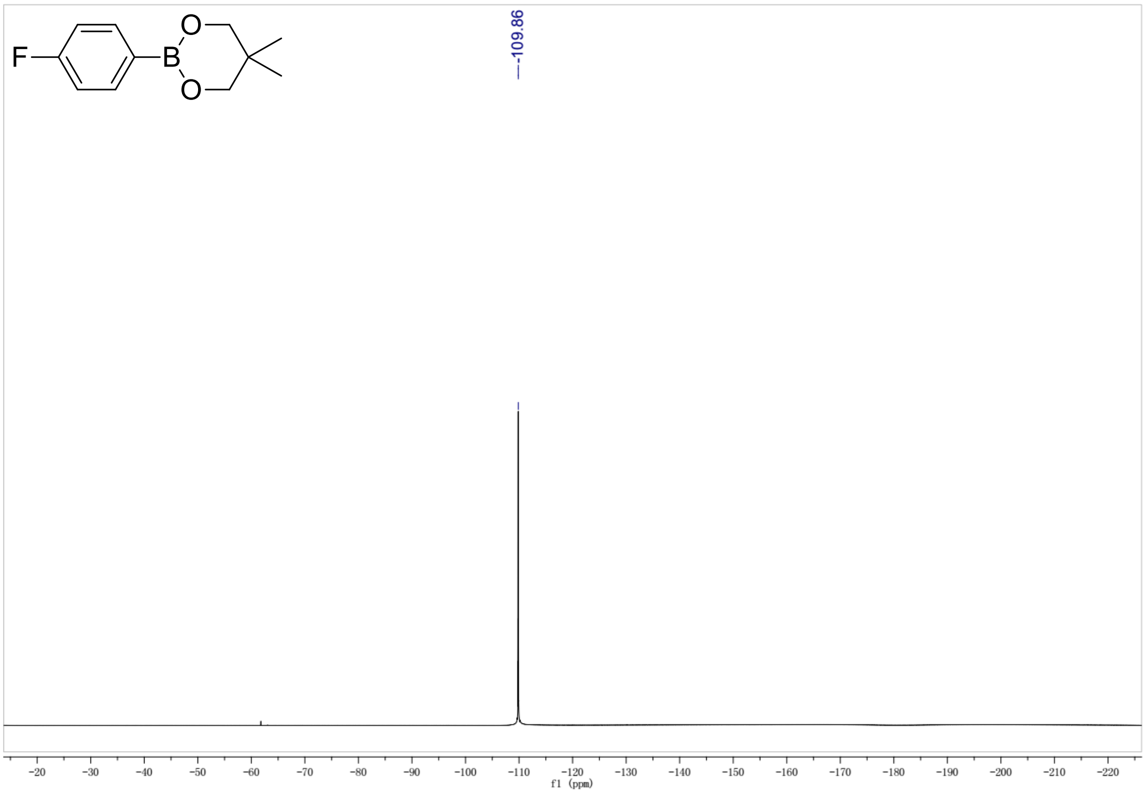

$^{19}F\{^1H\}$ NMR spectrum of compound **3e** in $CDCl_3$ (470 MHz).

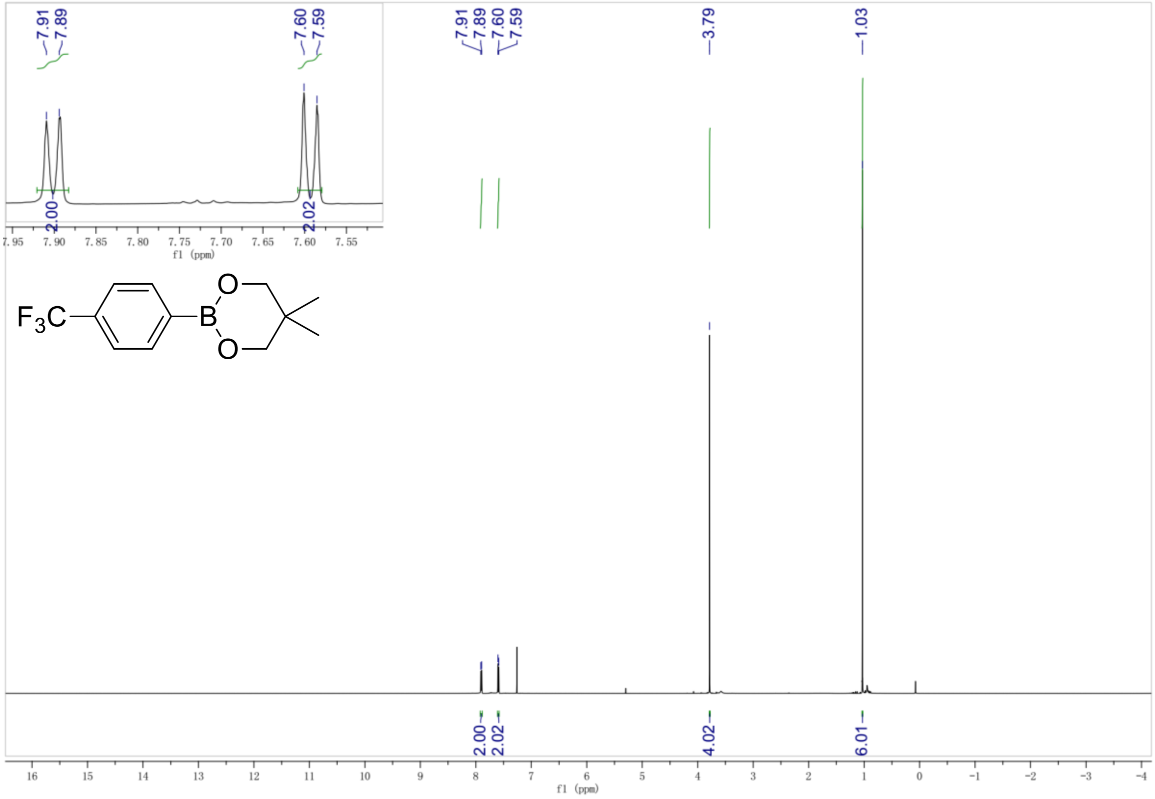

^{1}H NMR spectrum of compound **3f** in $CDCl_3$ (500 MHz).

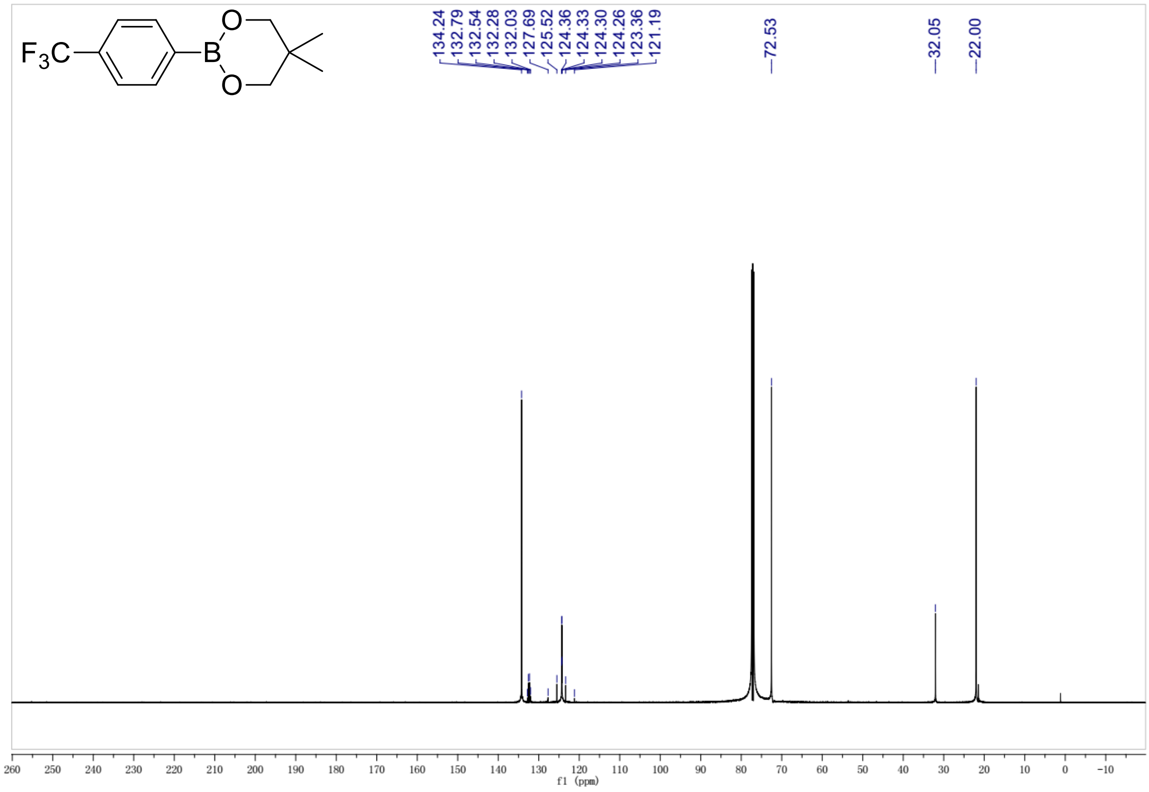

^{13}C{^{1}H} NMR spectrum of compound **3f** in $CDCl_3$ (125 MHz).

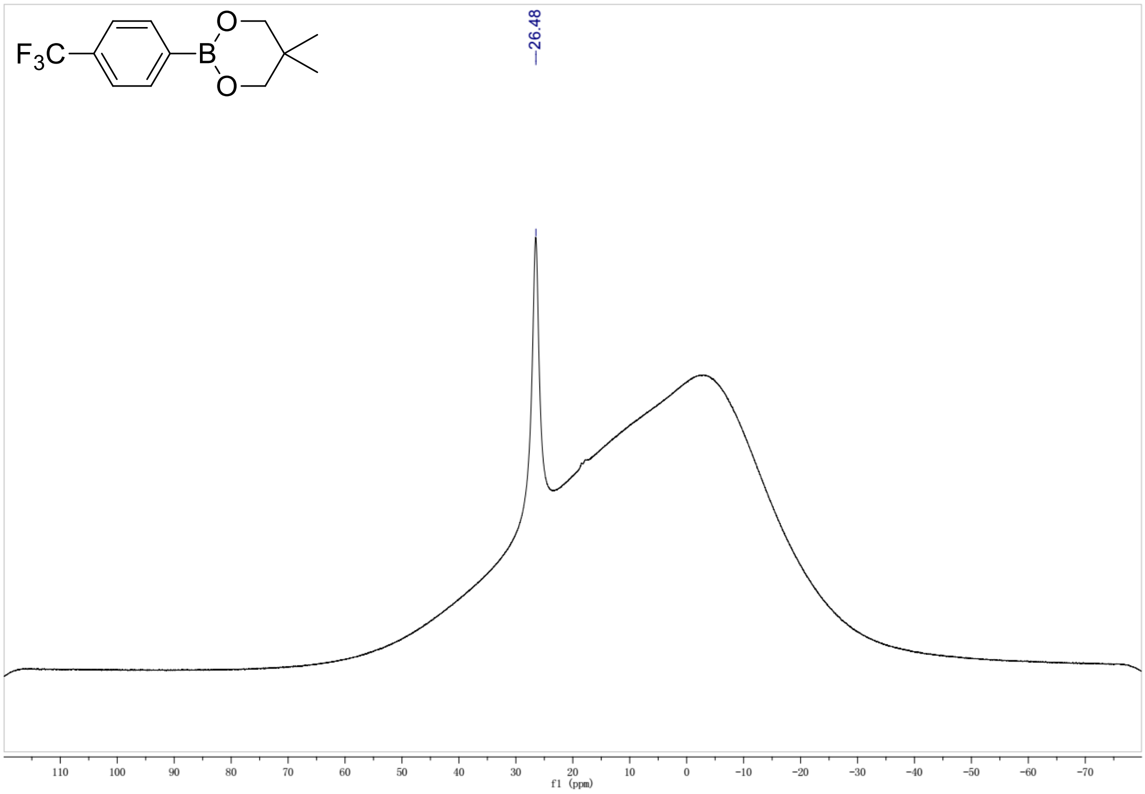

^{11}B{^{1}H} NMR spectrum of compound **3f** in $CDCl_3$ (160 MHz).

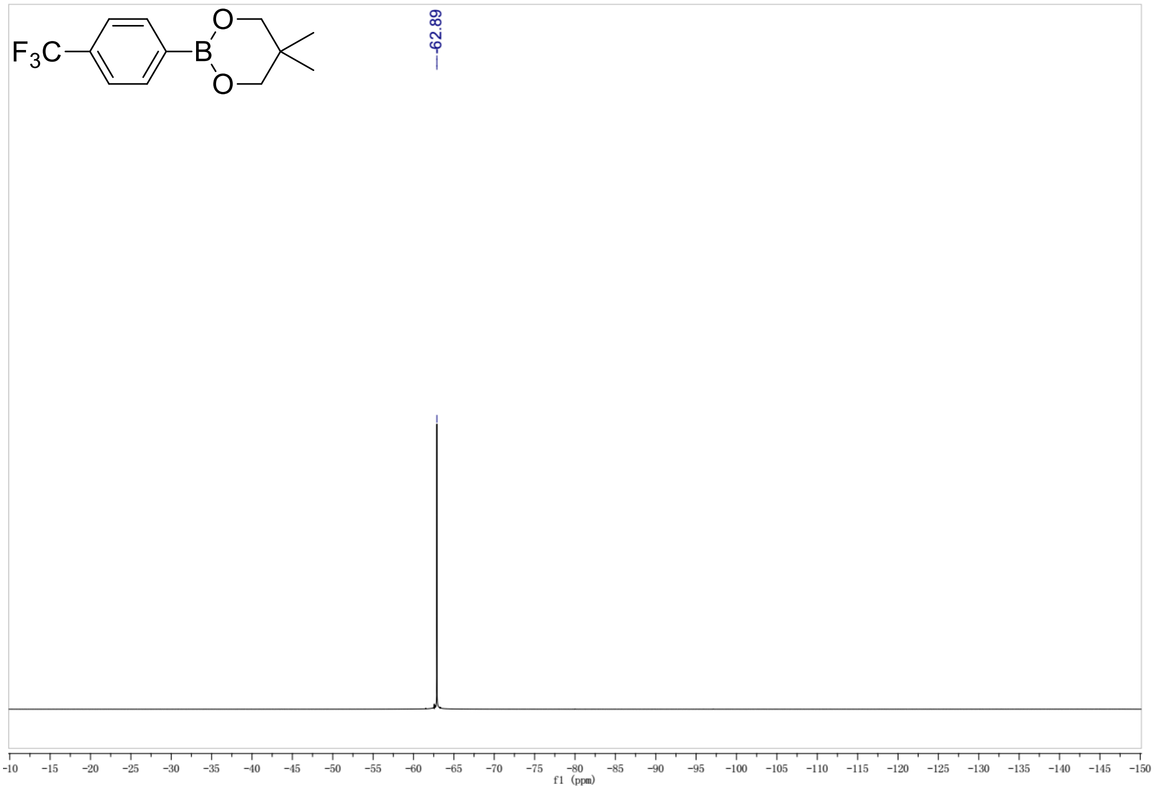

^{19}F{^{1}H} NMR spectrum of compound **3f** in $CDCl_3$ (470 MHz).

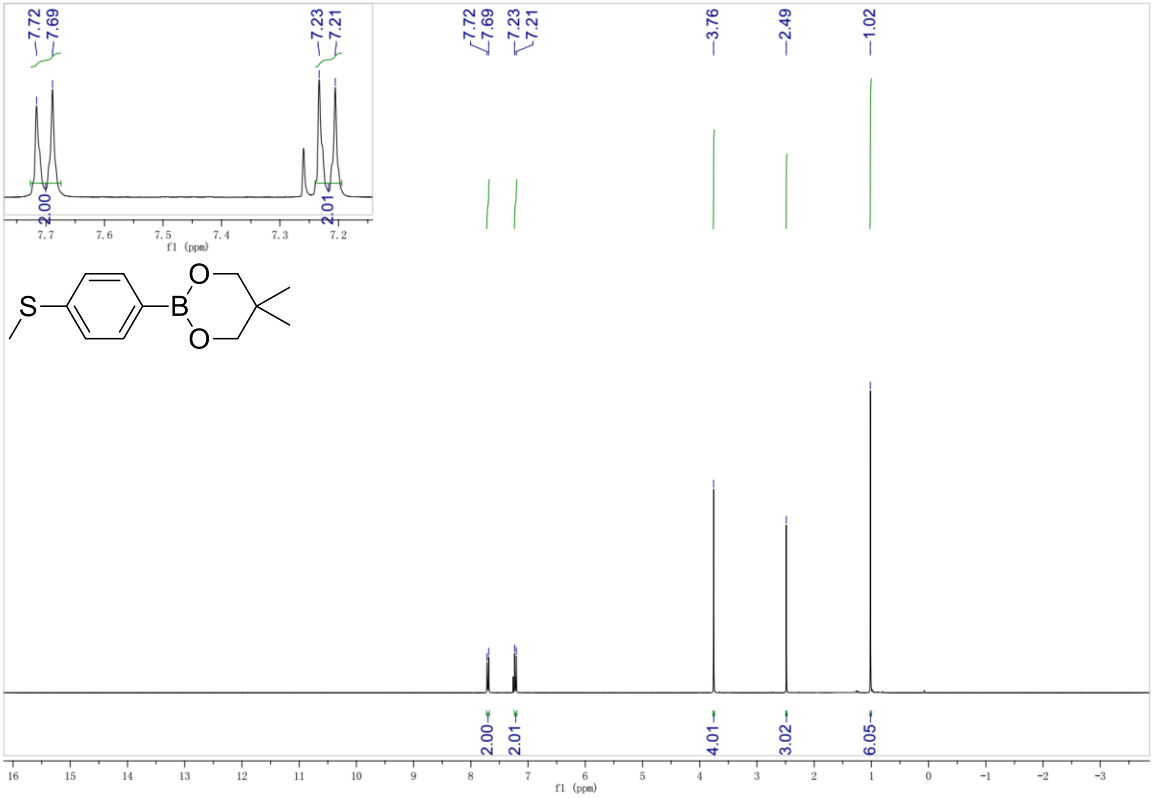

^{1}H NMR spectrum of compound **3g** in $CDCl_3$ (300 MHz).

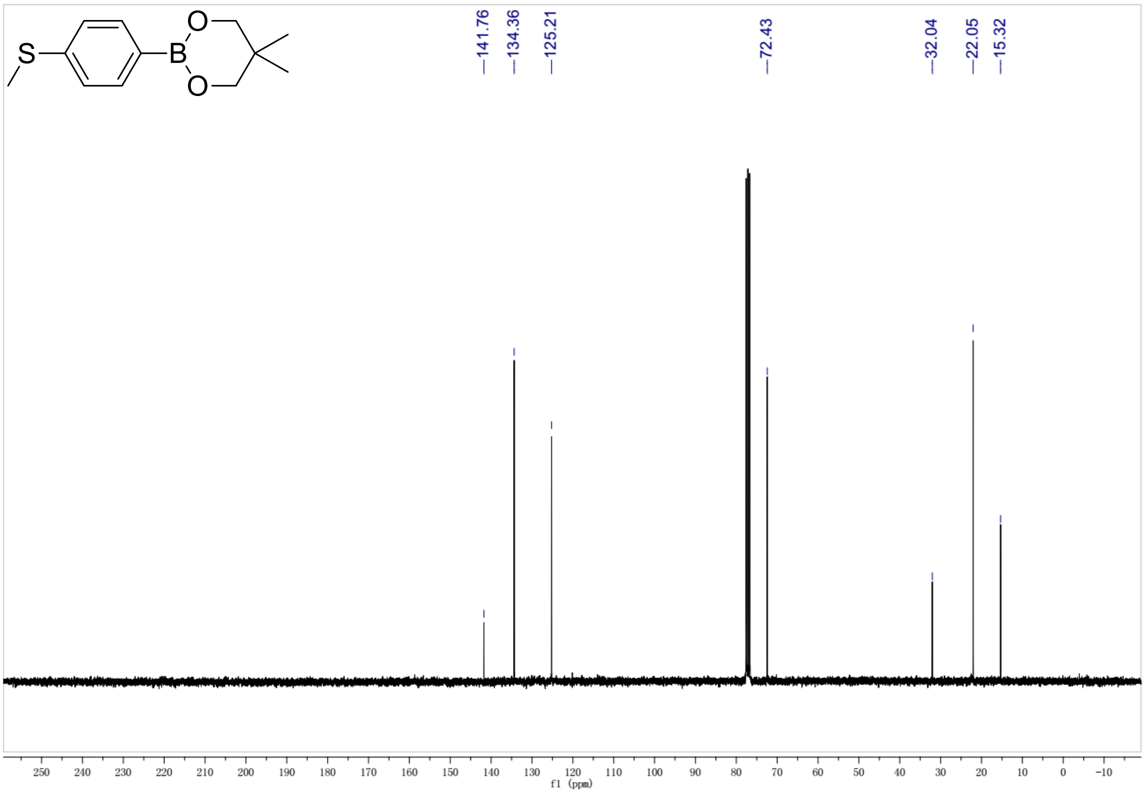

$^{13}C\{^{1}H\}$ NMR spectrum of compound **3g** in $CDCl_3$ (75 MHz).

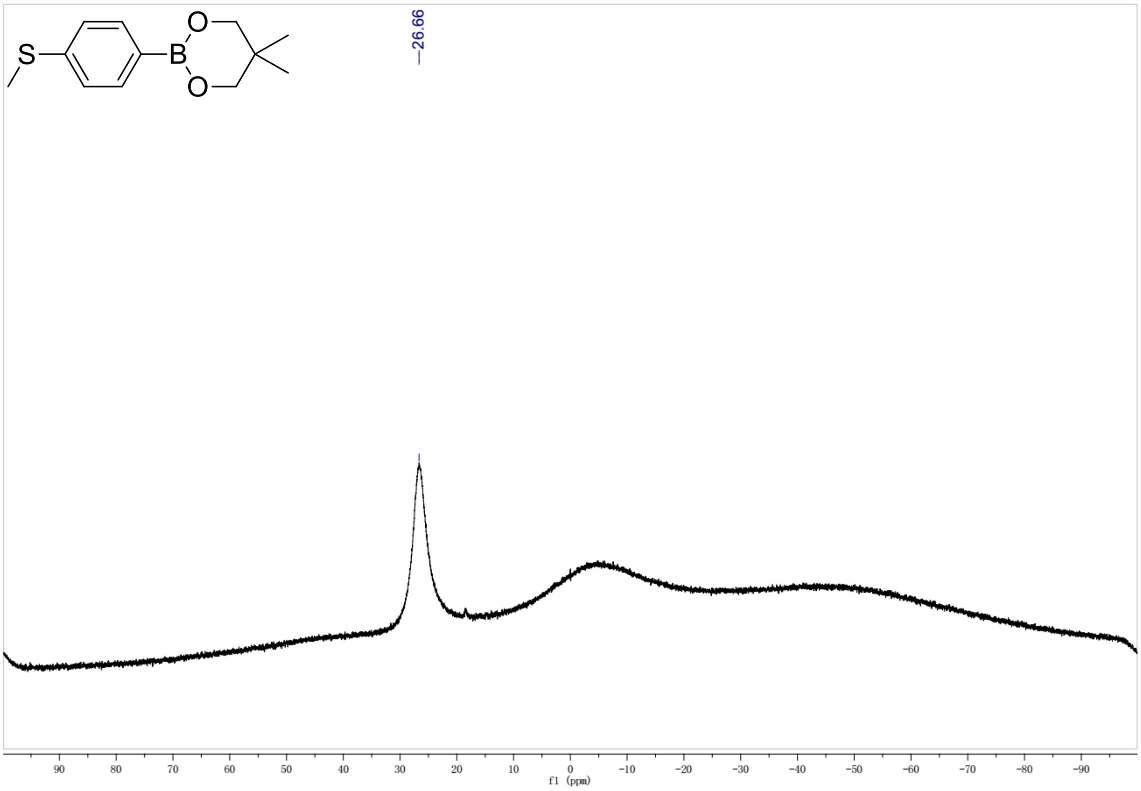

$^{11}B\{^{1}H\}$ NMR spectrum of compound **3g** in $CDCl_3$ (96 MHz).

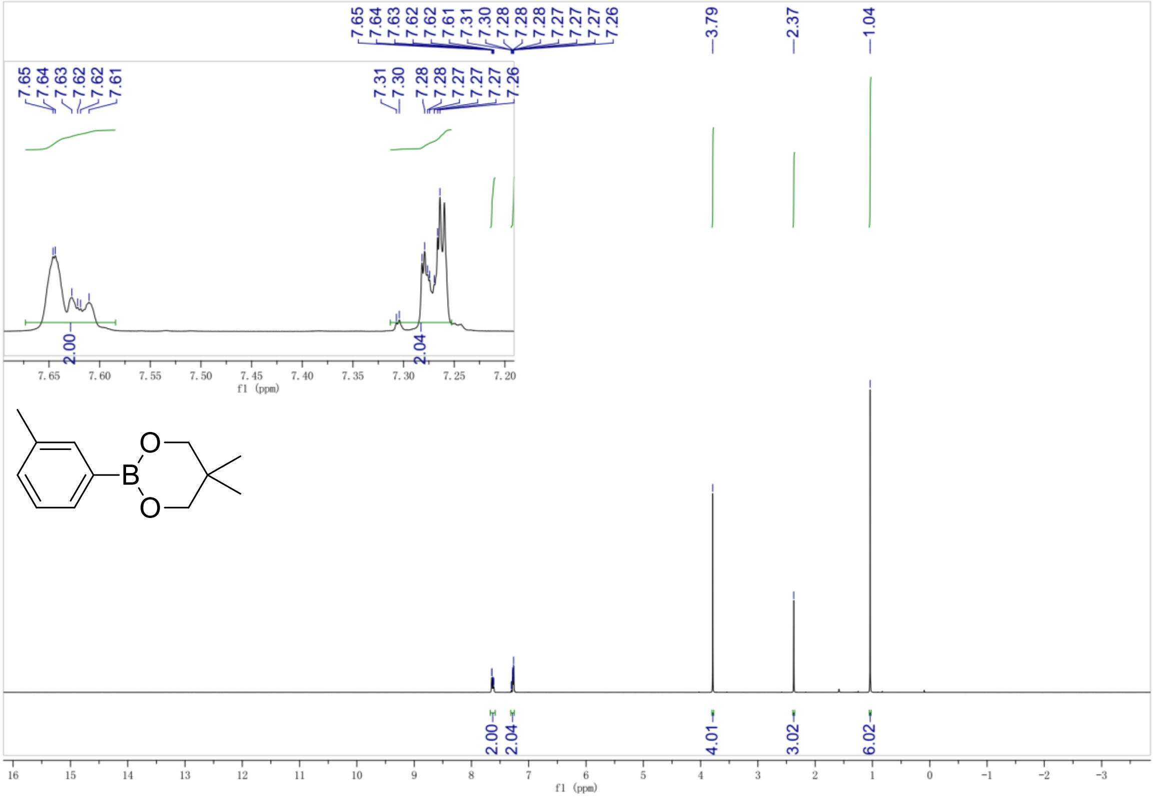

^{1}H NMR spectrum of compound **3h** in $CDCl_3$ (300 MHz).

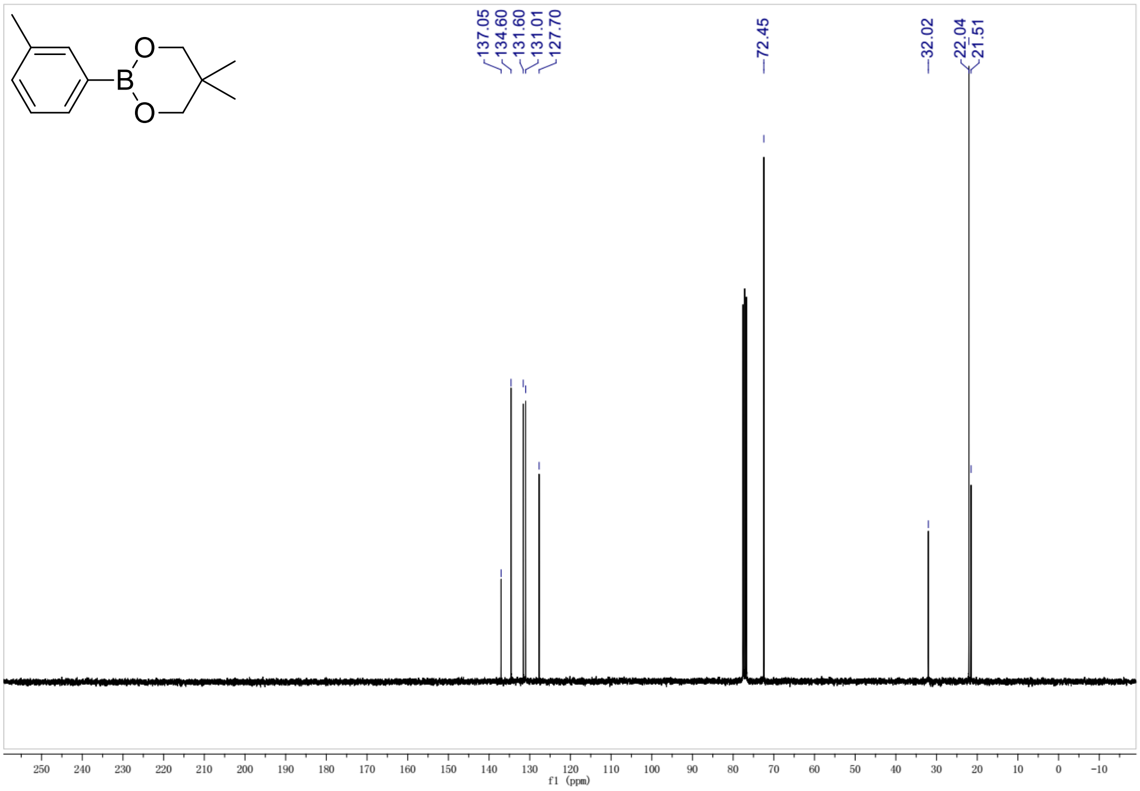

$^{13}C\{^1H\}$ NMR spectrum of compound **3h** in $CDCl_3$ (75 MHz).

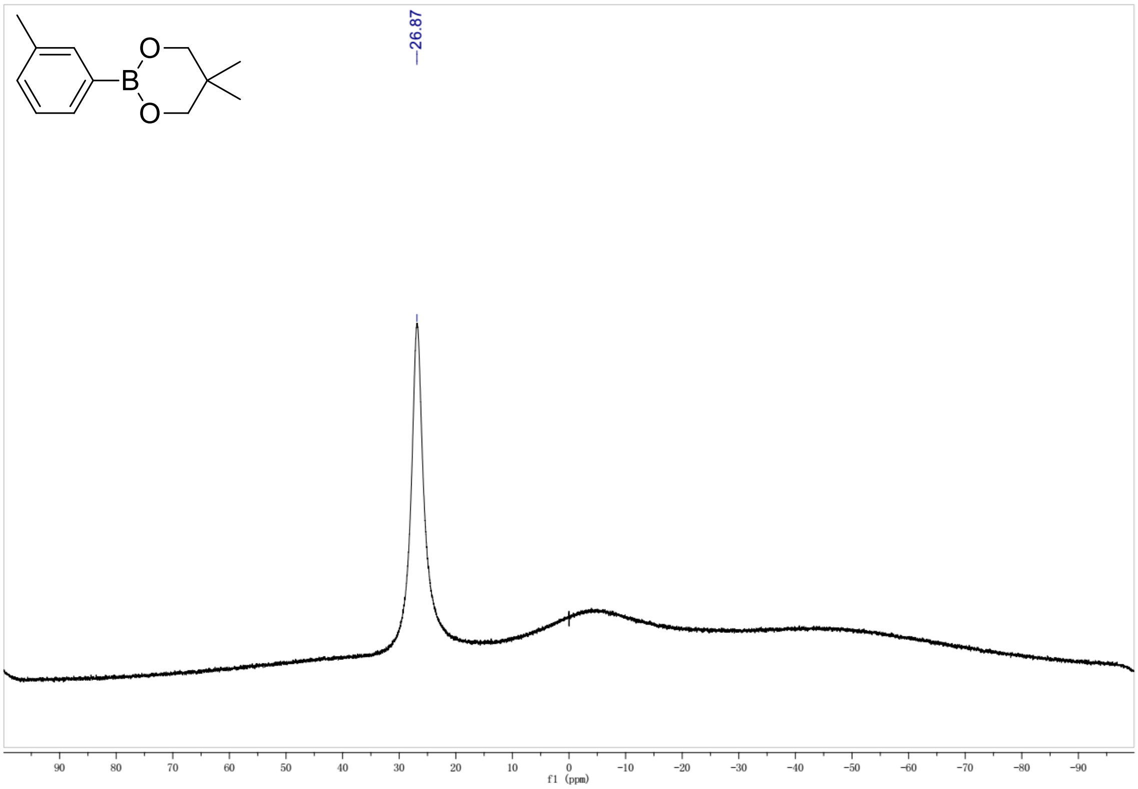

$^{11}B\{^1H\}$ NMR spectrum of compound **3h** in $CDCl_3$ (96 MHz).

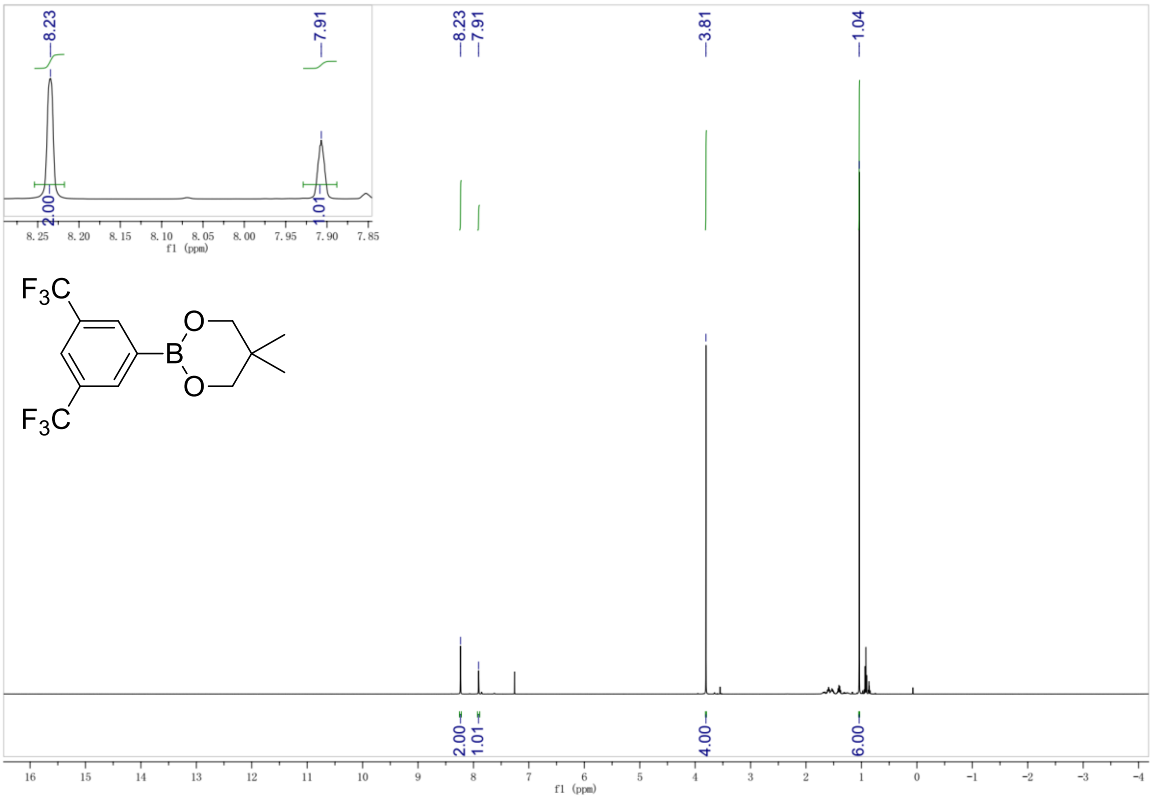

^{1}H NMR spectrum of compound **3k** in $CDCl_3$ (500 MHz).

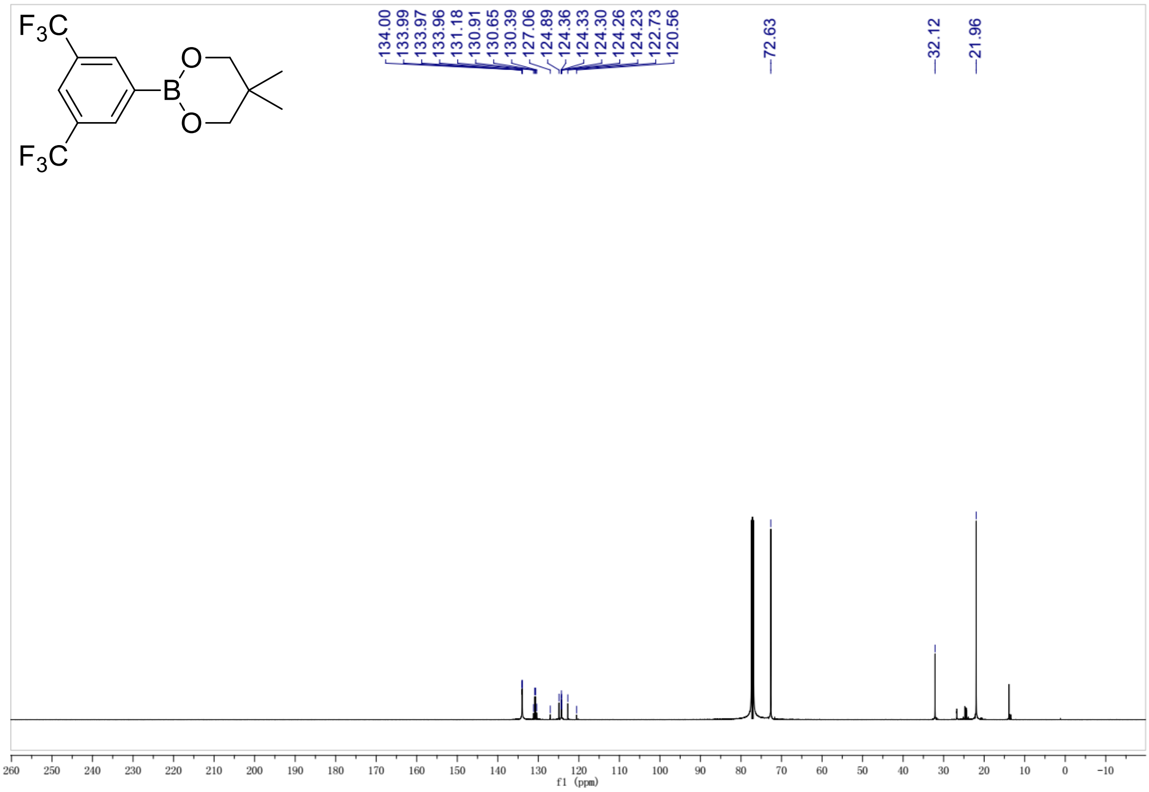

$^{13}C\{^{1}H\}$ NMR spectrum of compound **3k** in $CDCl_3$ (125 MHz).

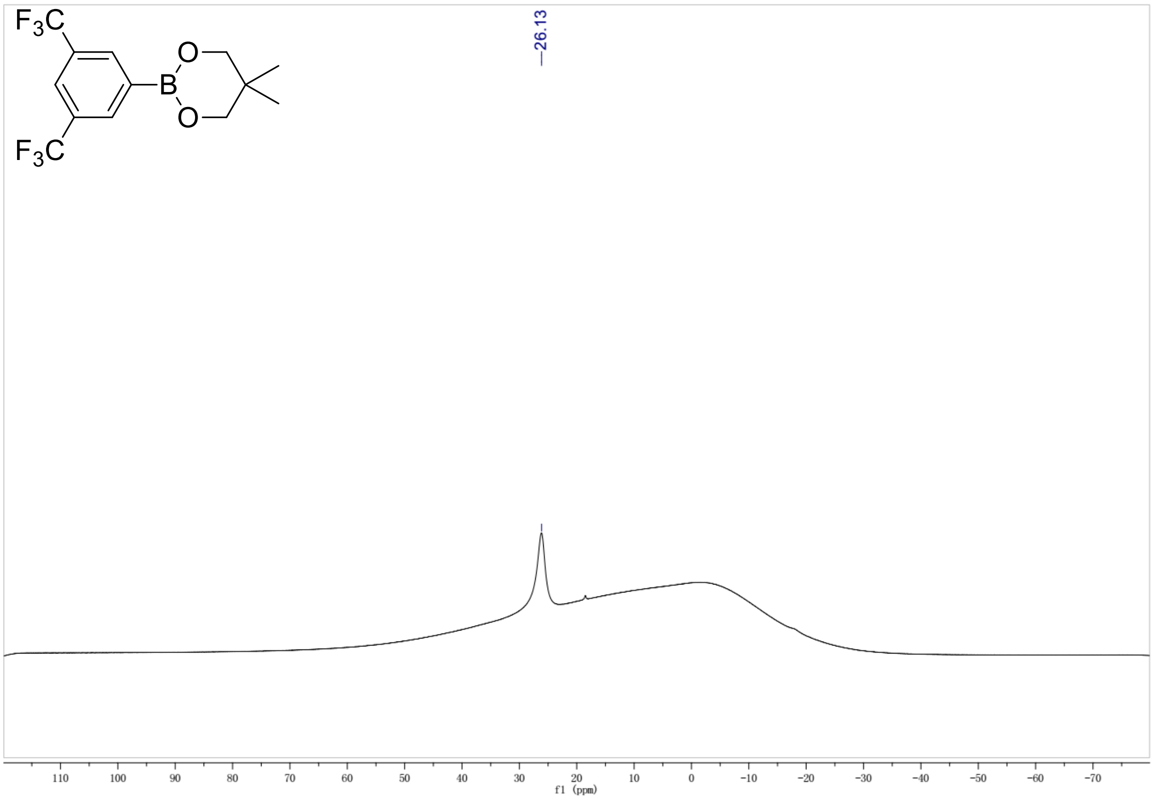

$^{11}B\{^{1}H\}$ NMR spectrum of compound **3k** in $CDCl_3$ (160 MHz).

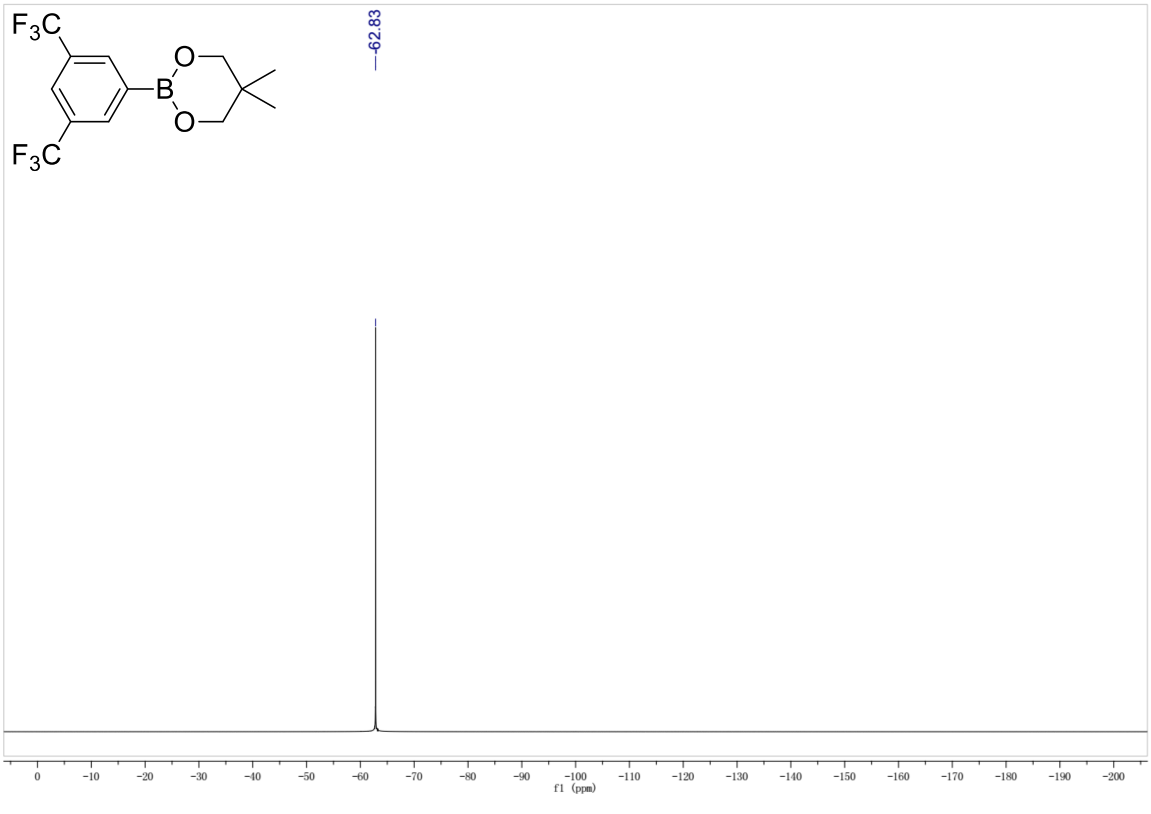

$^{19}F\{^{1}H\}$ NMR spectrum of compound **3k** in $CDCl_3$ (470 MHz).

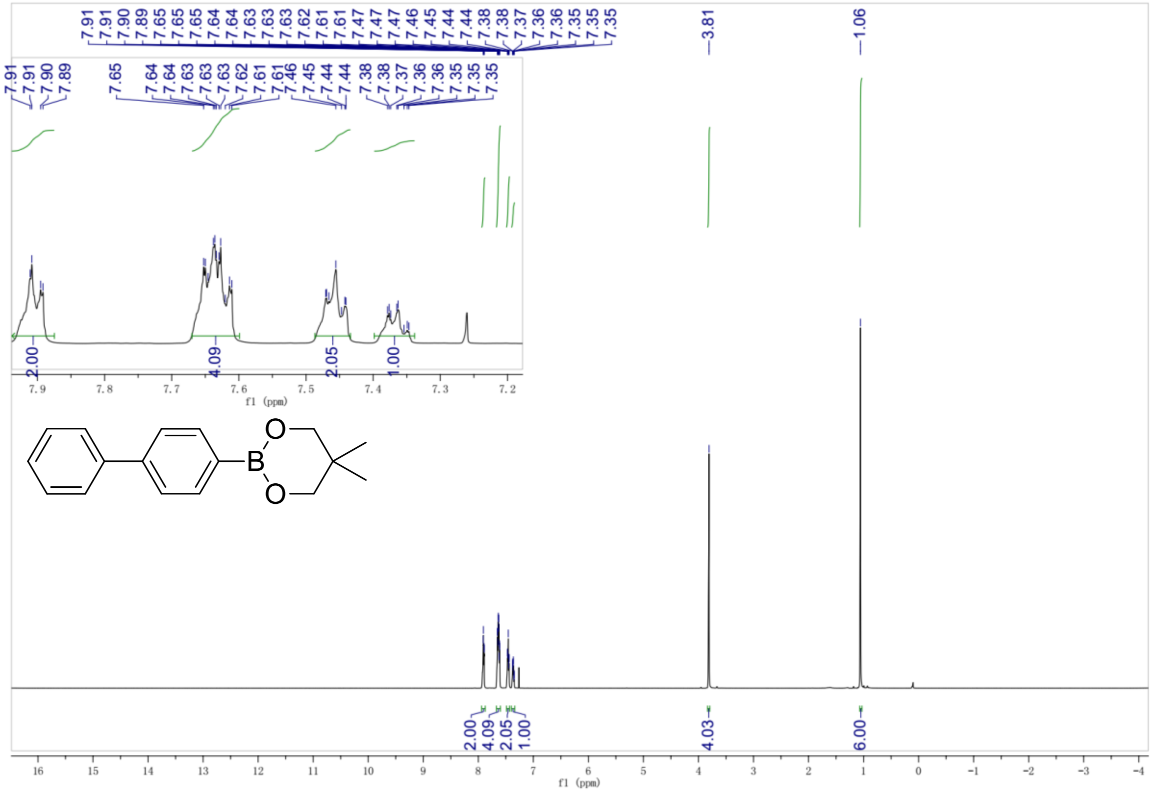

^1H NMR spectrum of compound **3l** in $CDCl_3$ (500 MHz).

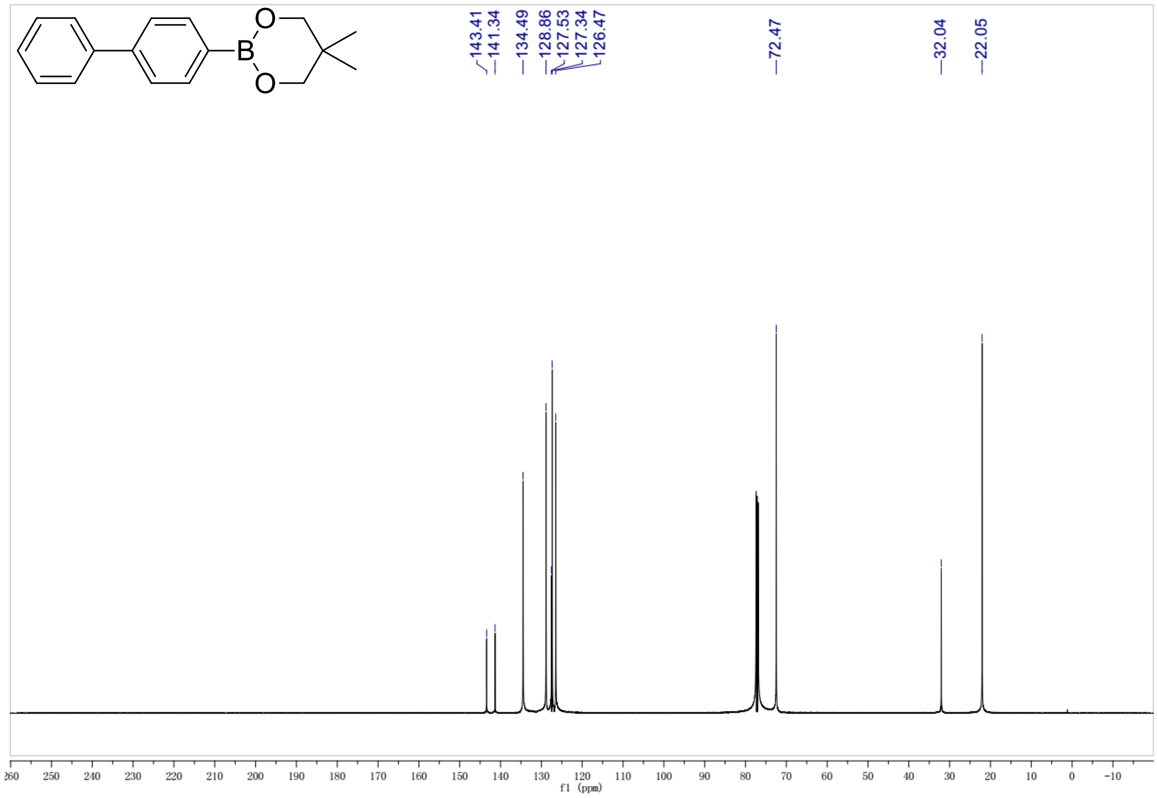

$^{13}C\{^1H\}$ NMR spectrum of compound **3l** in $CDCl_3$ (125 MHz).

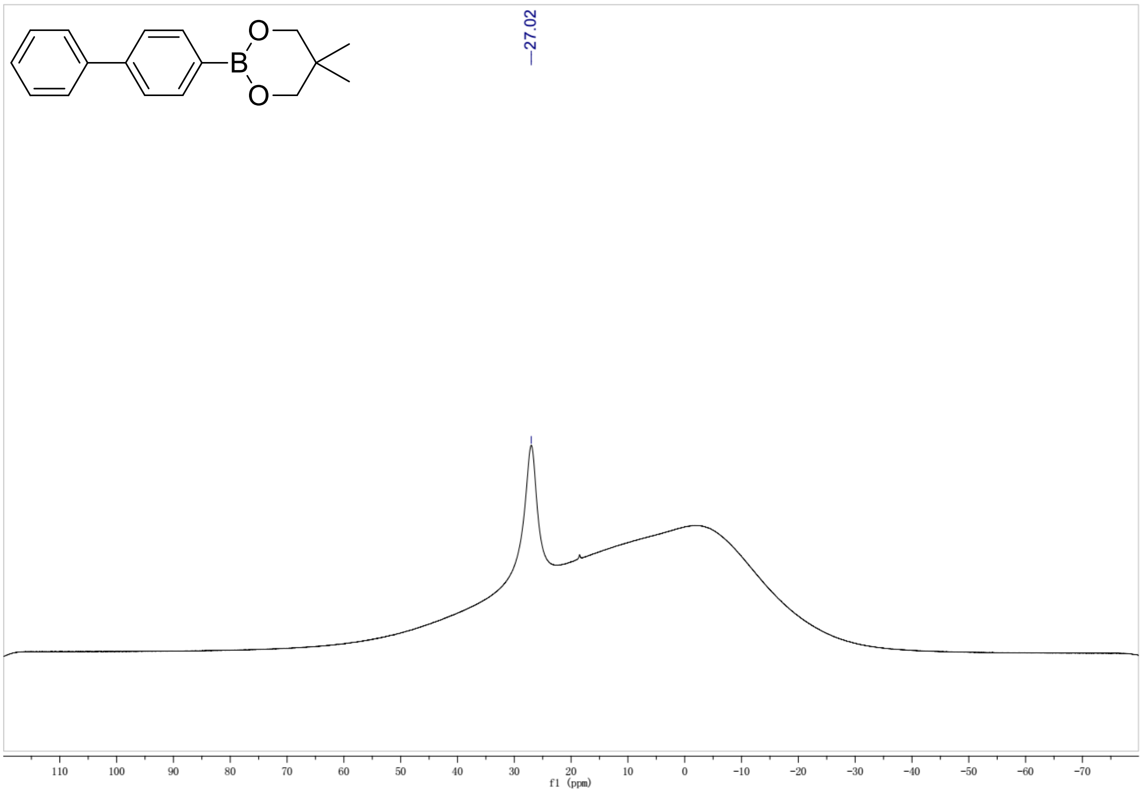

$^{11}B\{^{1}H\}$ NMR spectrum of compound **3l** in $CDCl_3$ (160 MHz).

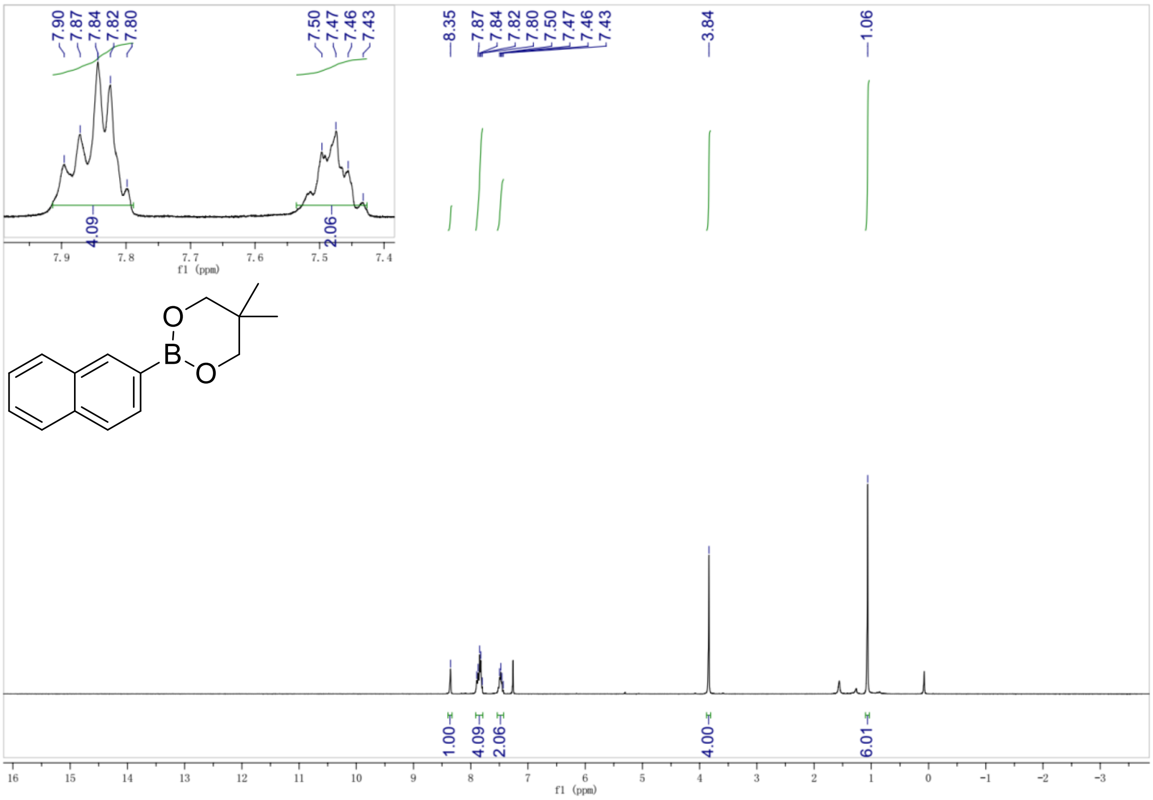

^{1}H NMR spectrum of compound **3m** in $CDCl_3$ (300 MHz).

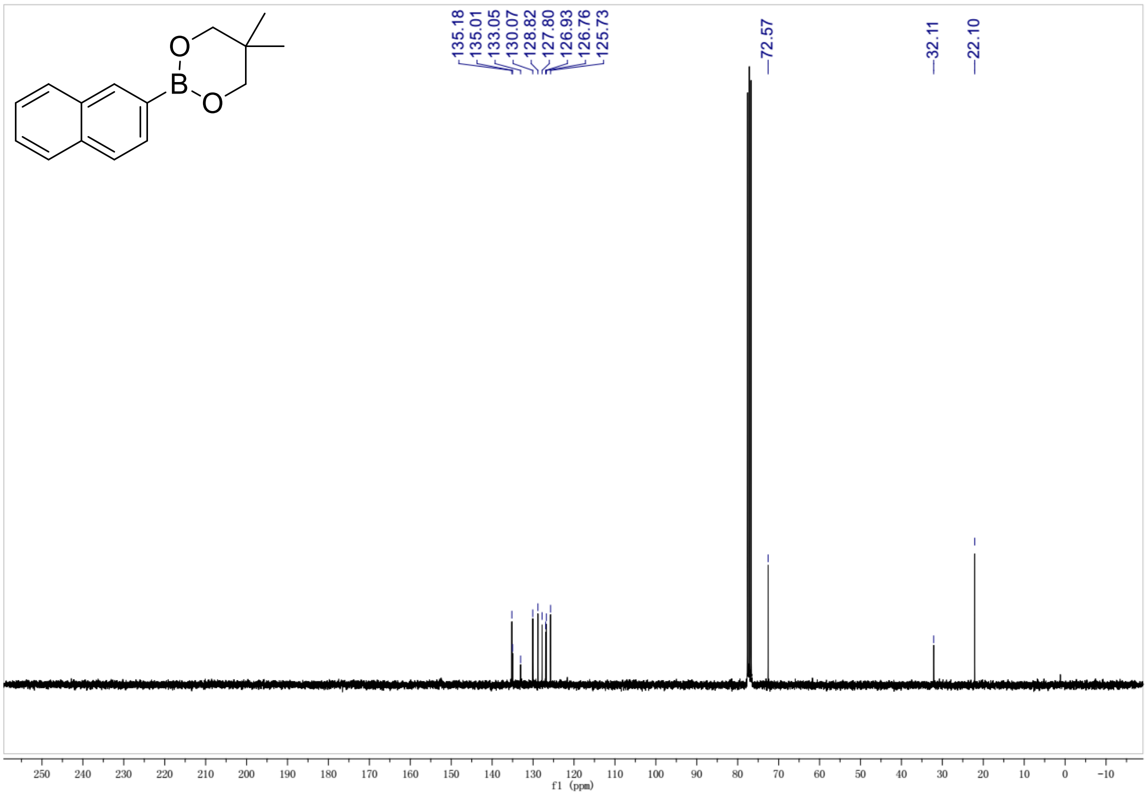

$^{13}C\{^{1}H\}$ NMR spectrum of compound **3m** in $CDCl_3$ (75 MHz).

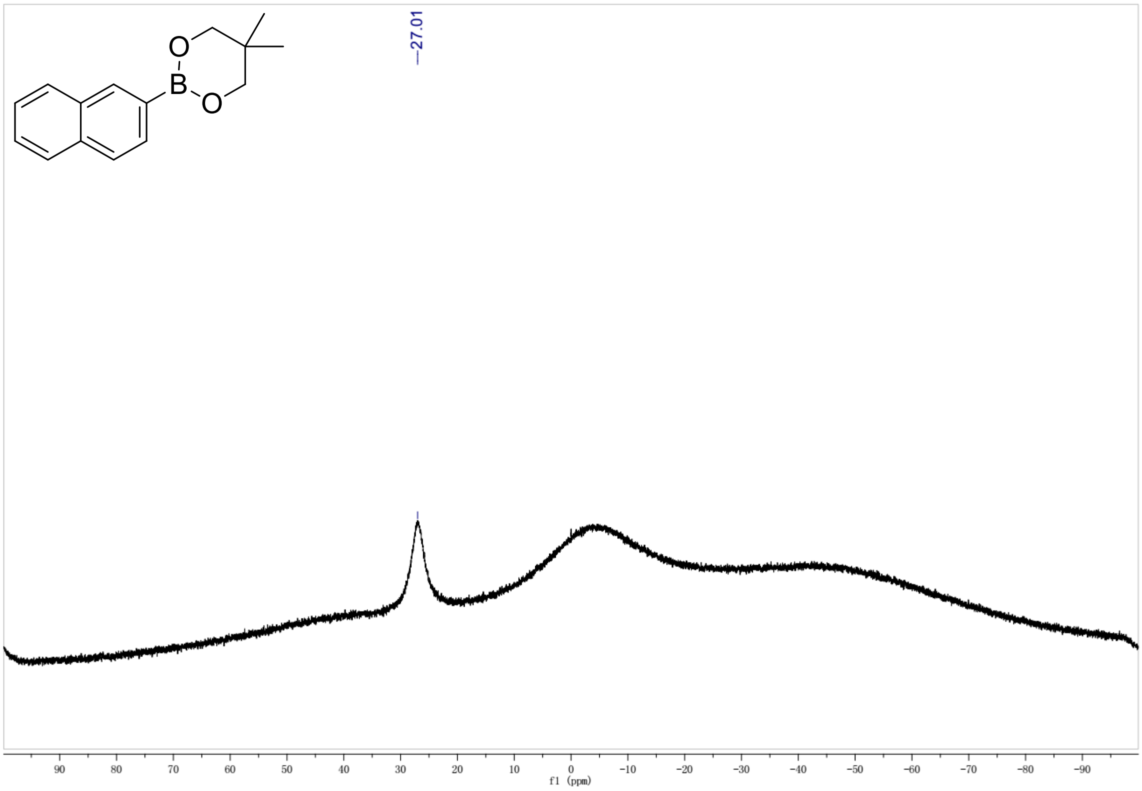

$^{11}B\{^{1}H\}$ NMR spectrum of compound **3m** in $CDCl_3$ (96 MHz).

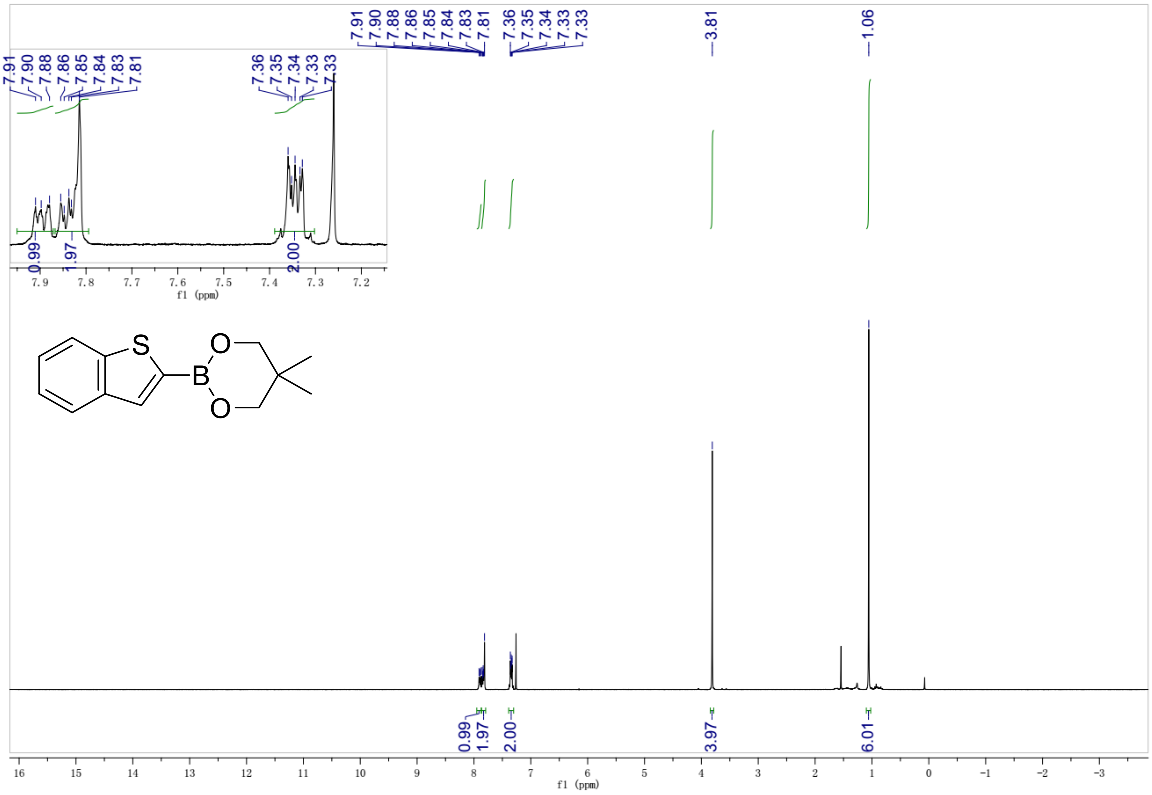

^{1}H NMR spectrum of compound **3n** in $CDCl_3$ (300 MHz).

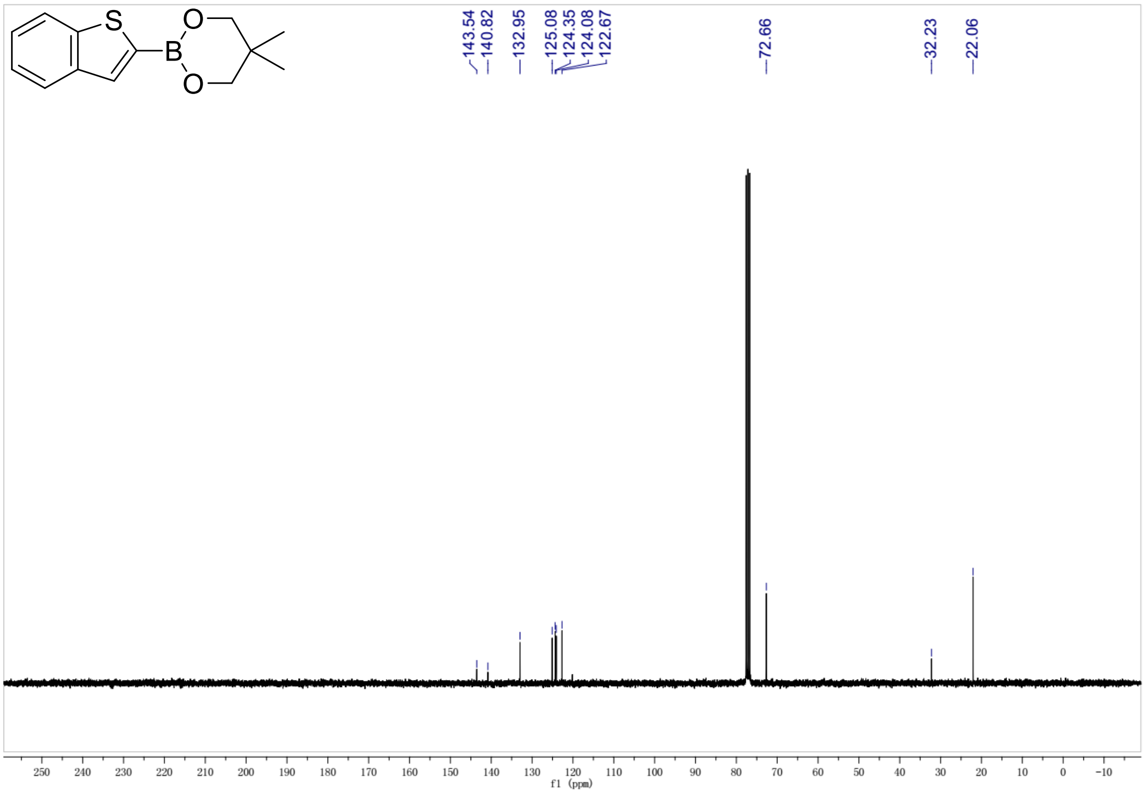

^{13}C{^{1}H} NMR spectrum of compound **3n** in $CDCl_3$ (75 MHz).

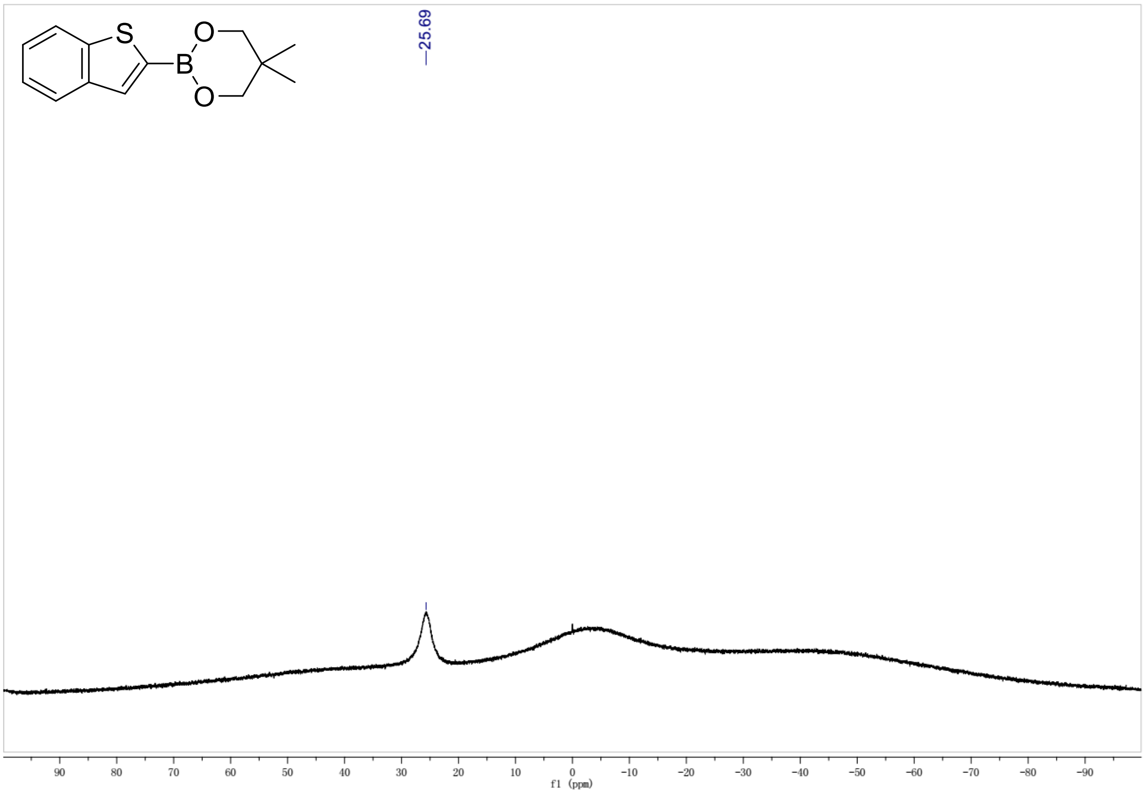

$^{11}B\{^{1}H\}$ NMR spectrum of compound **3n** in $CDCl_3$ (96 MHz).

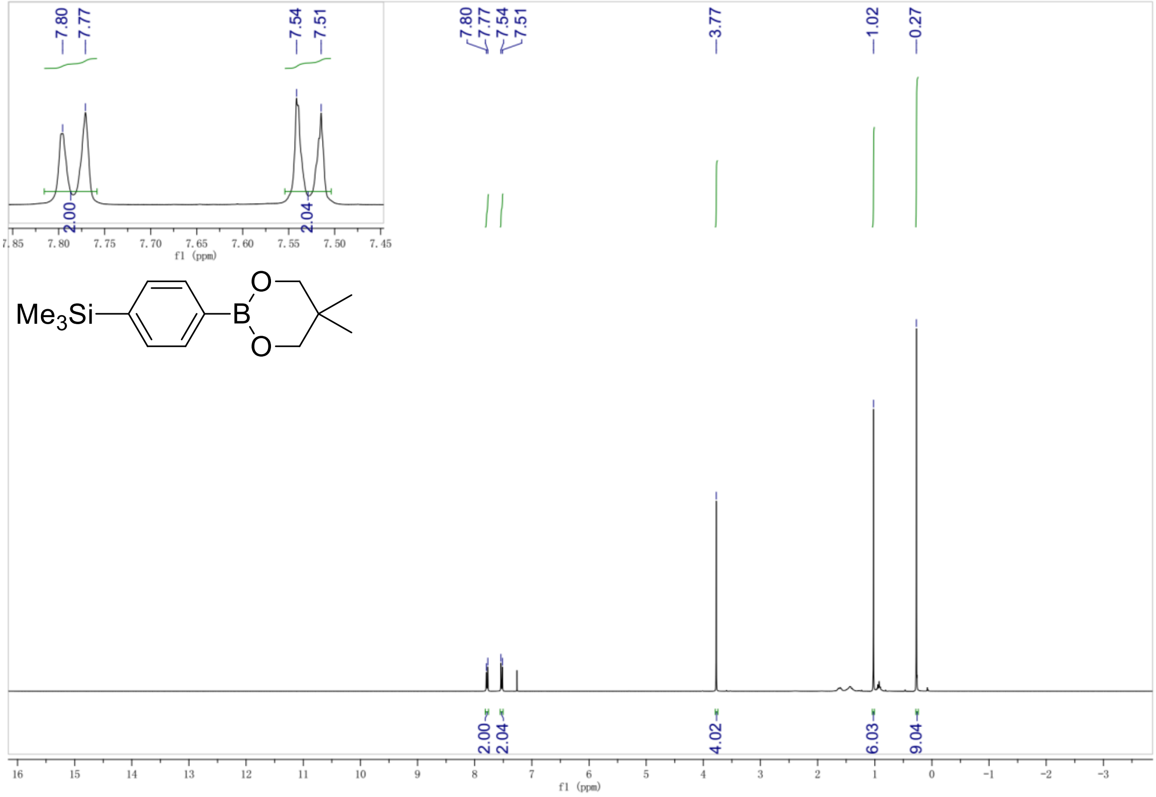

^{1}H NMR spectrum of compound **3q** in $CDCl_3$ (300 MHz).

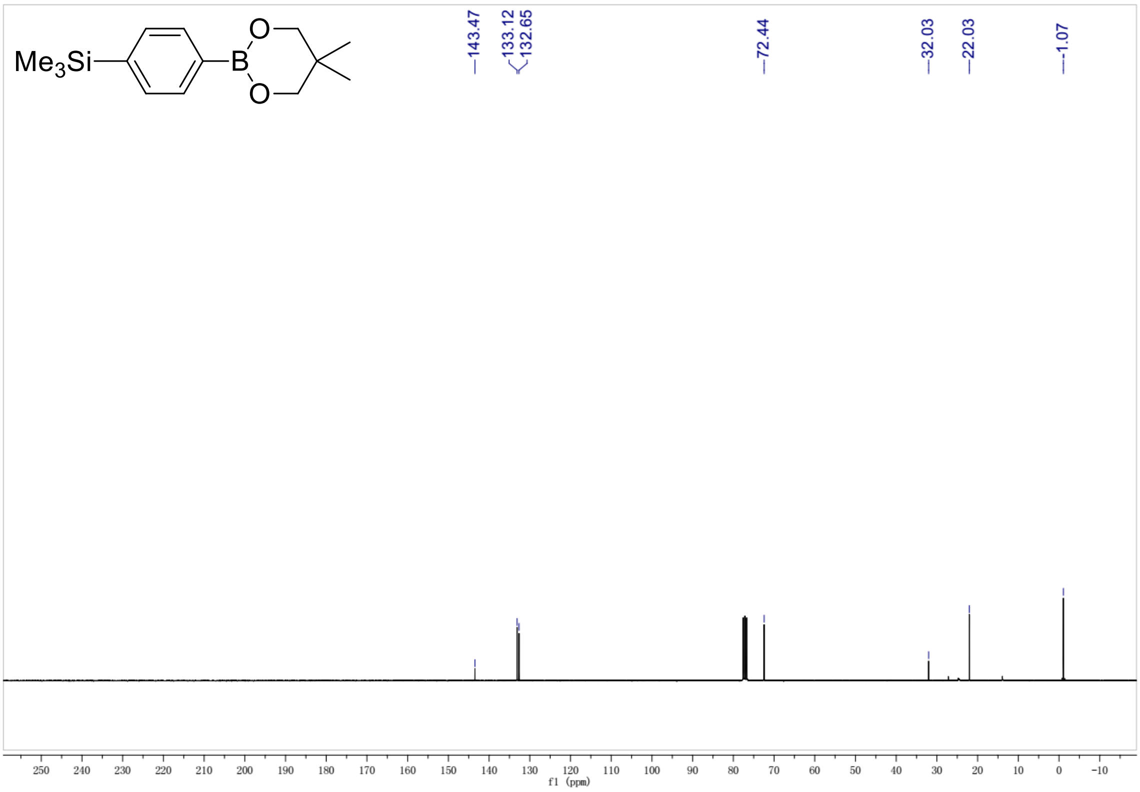

$^{13}C\{^1H\}$ NMR spectrum of compound **3q** in $CDCl_3$ (75 MHz).

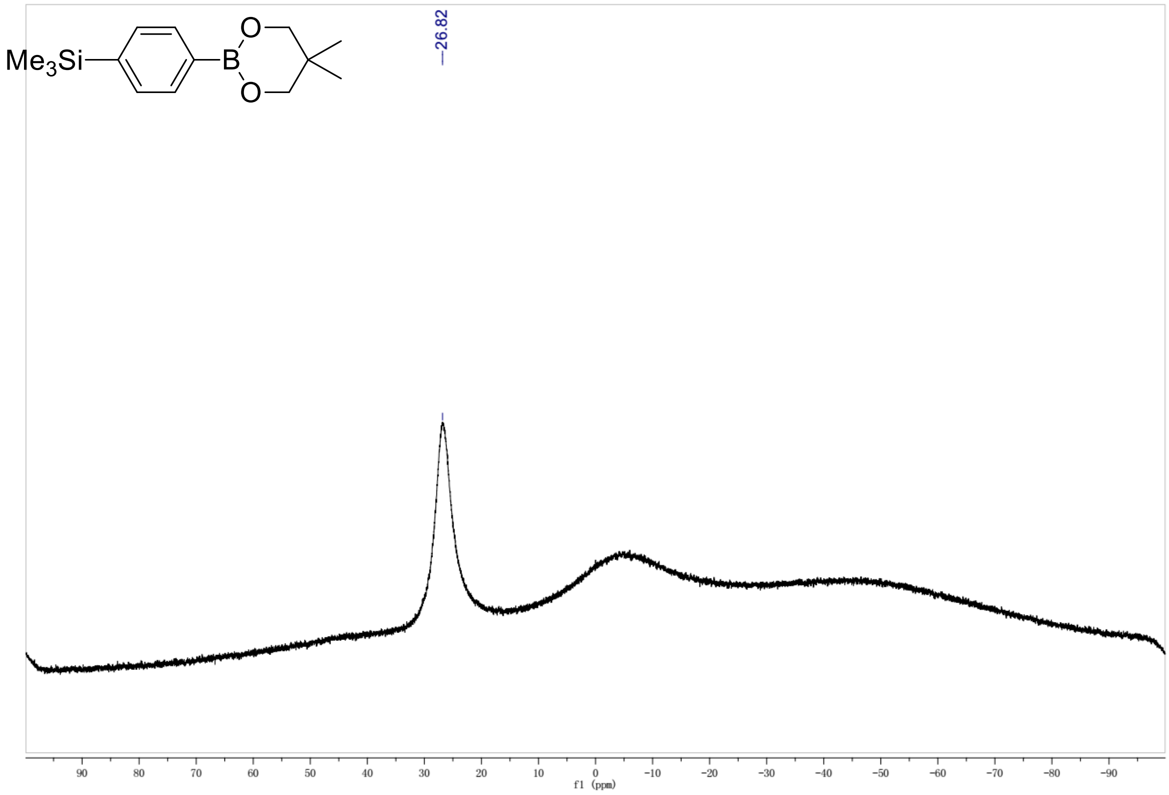

$^{11}B\{^1H\}$ NMR spectrum of compound **3q** in $CDCl_3$ (96 MHz).

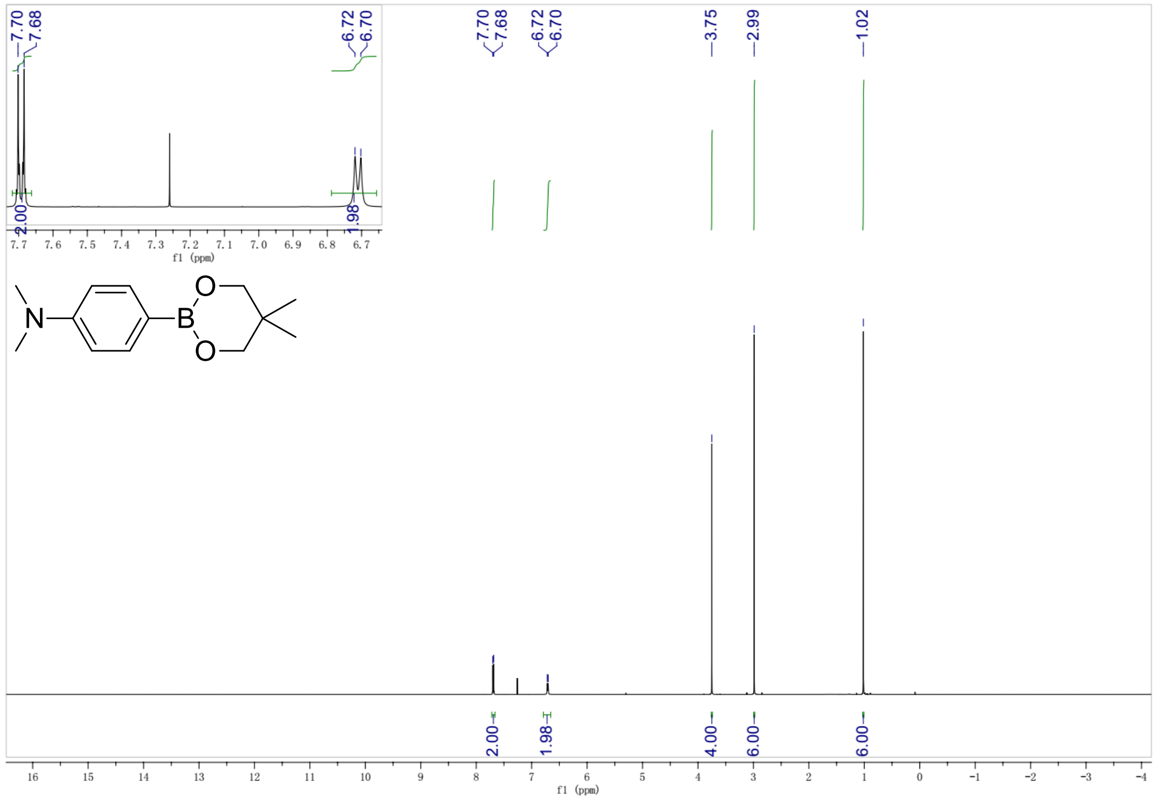

^{1}H NMR spectrum of compound **3r** in $CDCl_3$ (500 MHz).

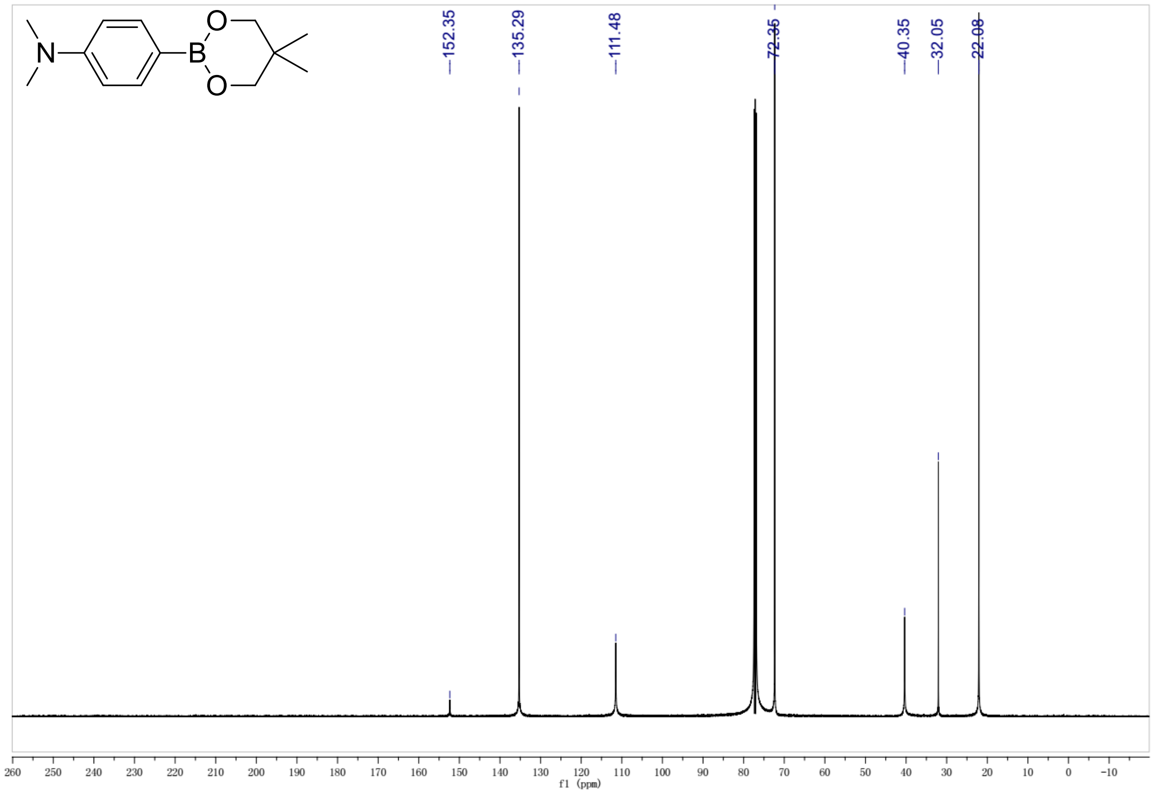

^{13}C{^{1}H} NMR spectrum of compound **3r** in $CDCl_3$ (125 MHz).

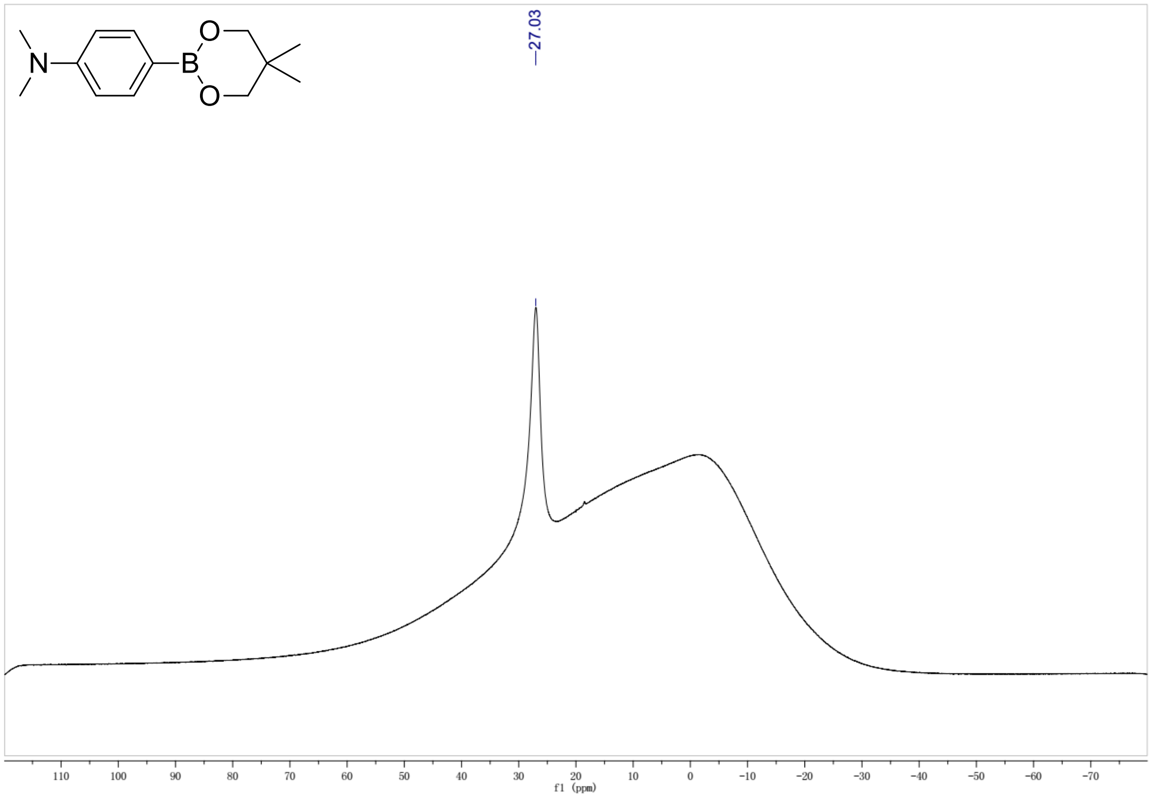

$^{11}B\{^{1}H\}$ NMR spectrum of compound **3r** in $CDCl_3$ (160 MHz).

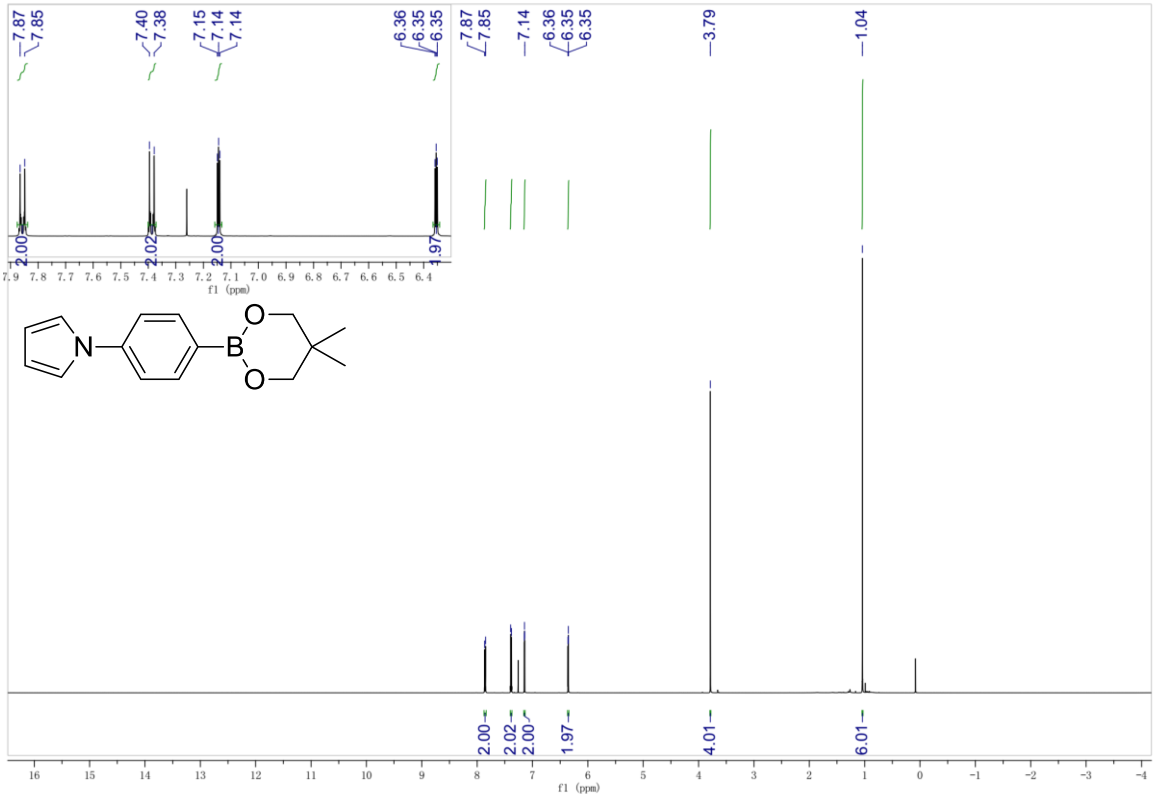

^{1}H NMR spectrum of compound **3s** in $CDCl_3$ (500 MHz).

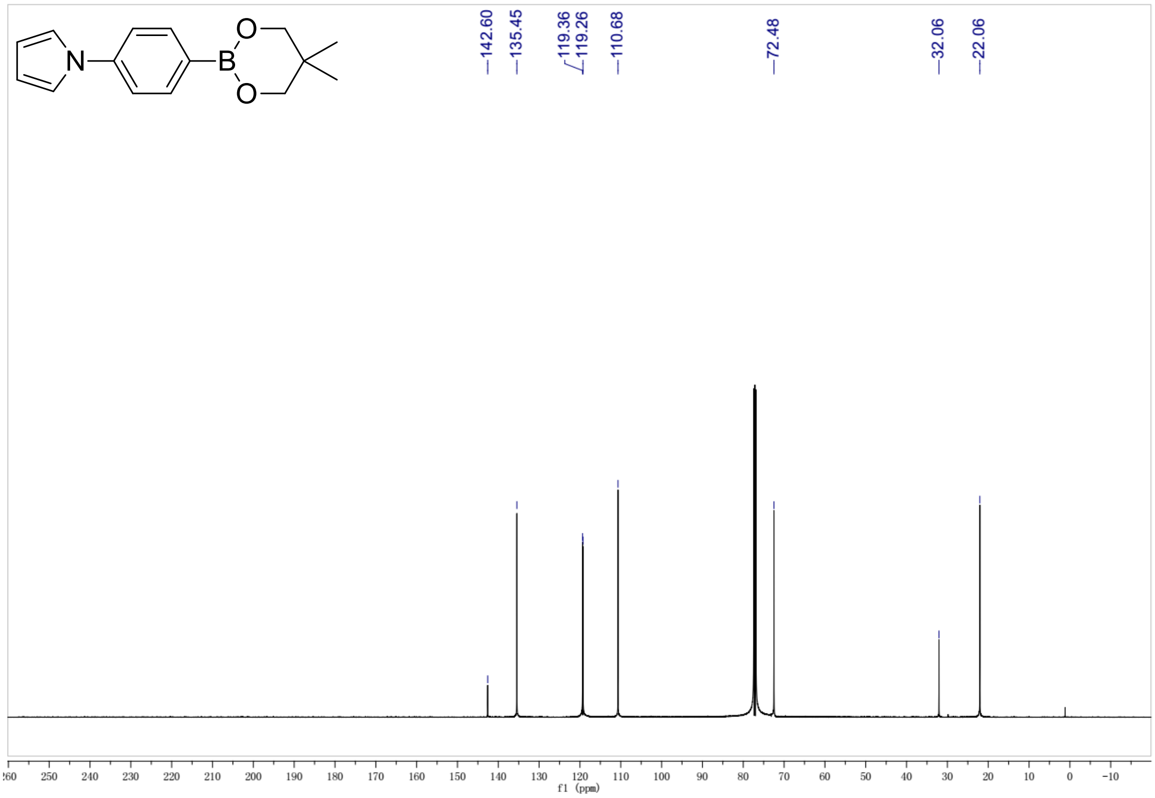

$^{13}C\{^{1}H\}$ NMR spectrum of compound **3s** in $CDCl_3$ (125 MHz).

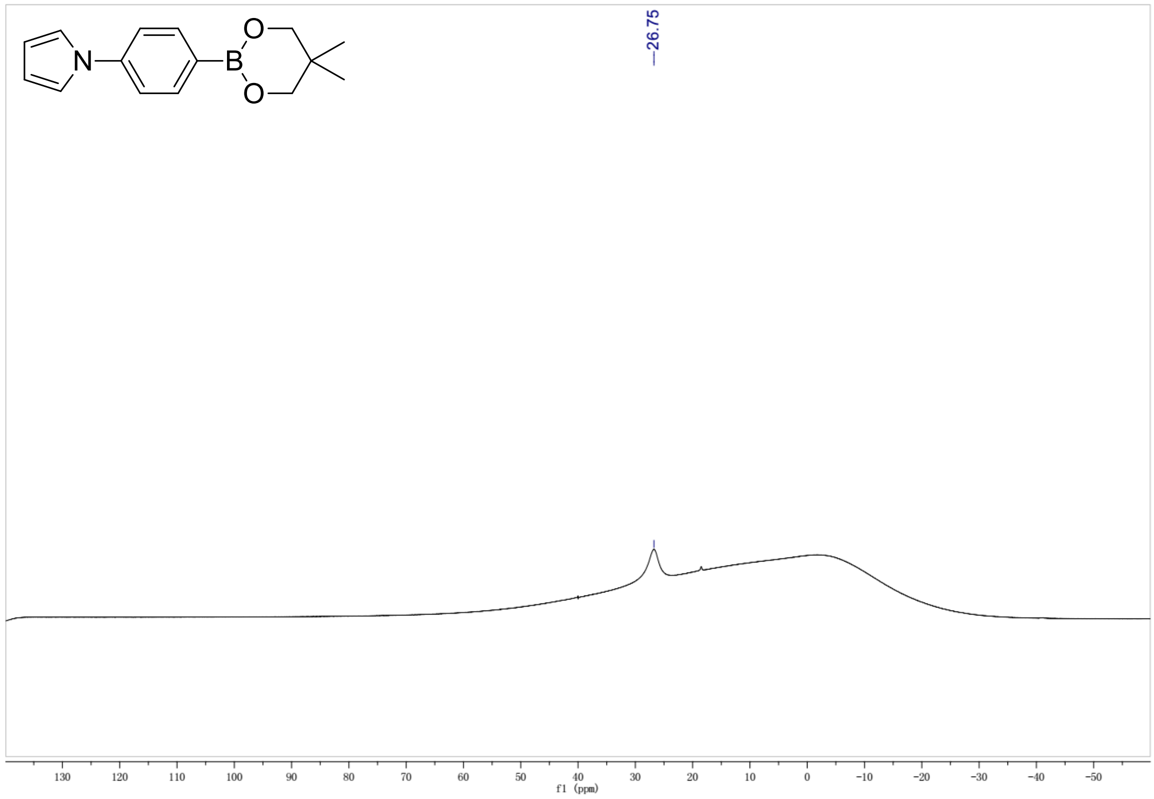

$^{11}B\{^{1}H\}$ NMR spectrum of compound **3s** in $CDCl_3$ (160 MHz).

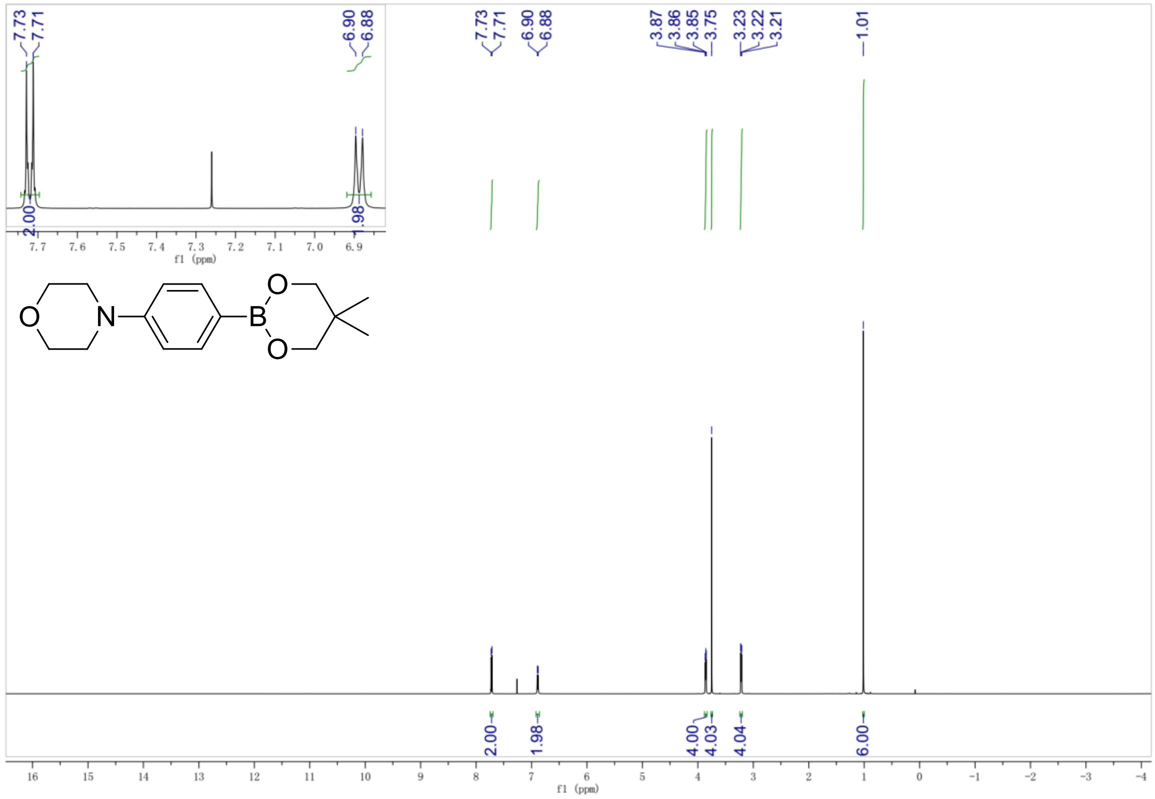

^{1}H NMR spectrum of compound **3t** in $CDCl_3$ (500 MHz).

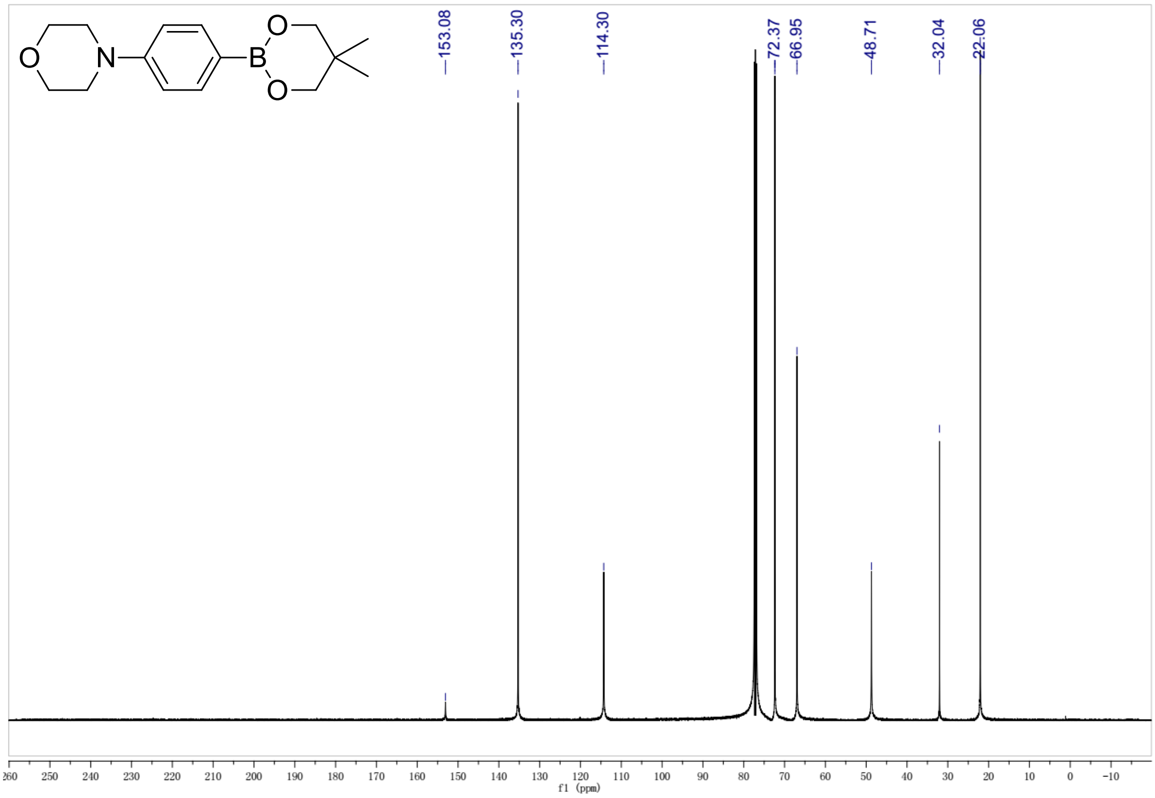

^{13}C{^{1}H} NMR spectrum of compound **3t** in $CDCl_3$ (125 MHz).

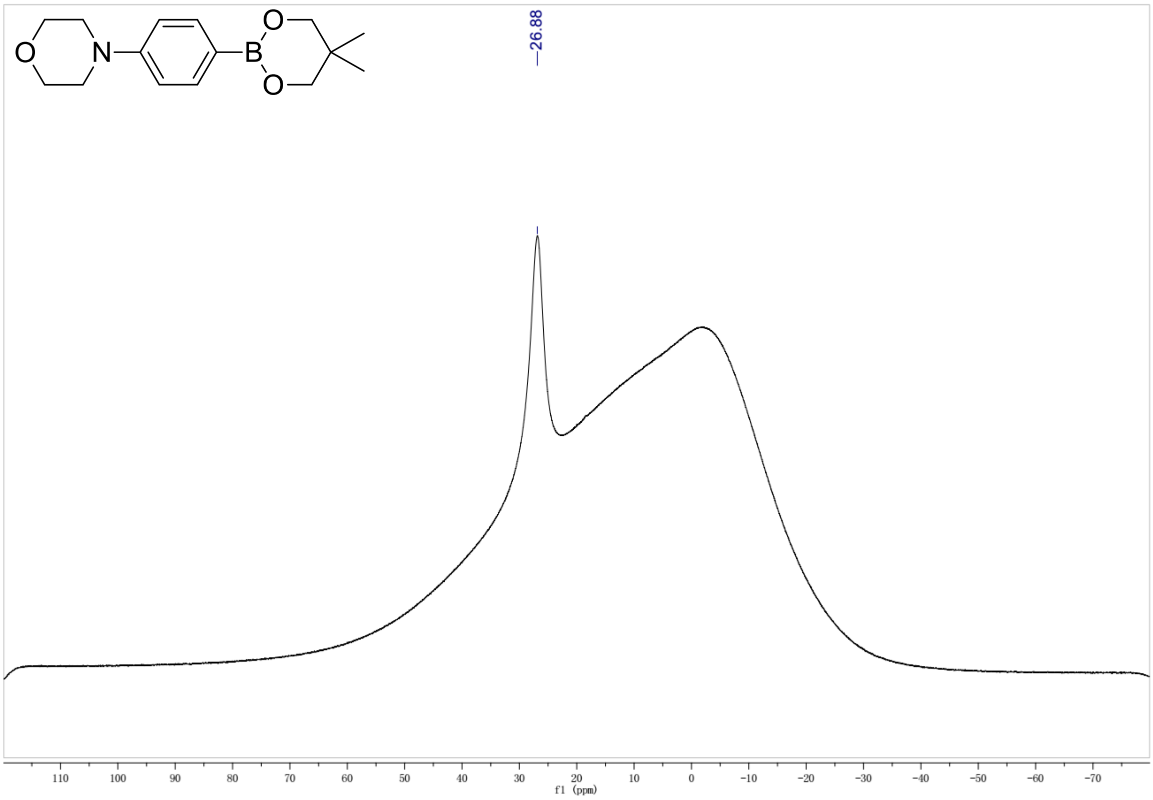

$^{11}B\{^{1}H\}$ NMR spectrum of compound **3t** in $CDCl_3$ (160 MHz).

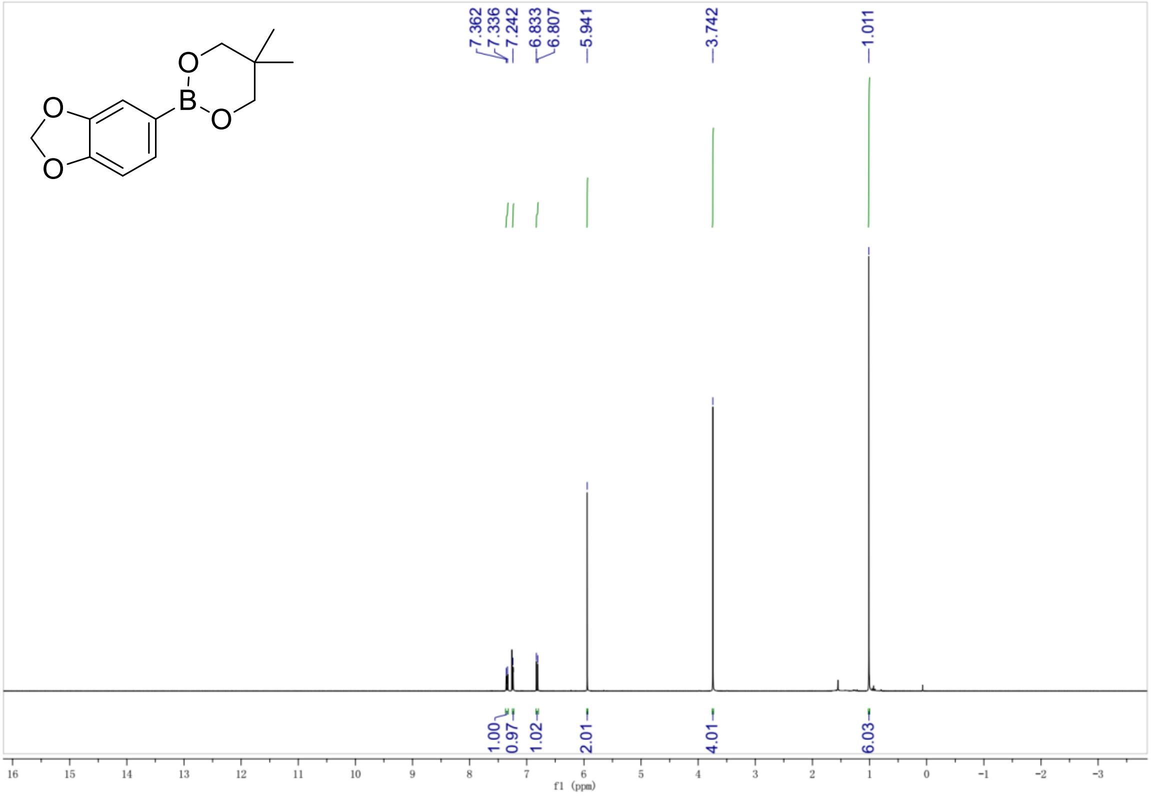

^{1}H NMR spectrum of compound **3u** in $CDCl_3$ (300 MHz).

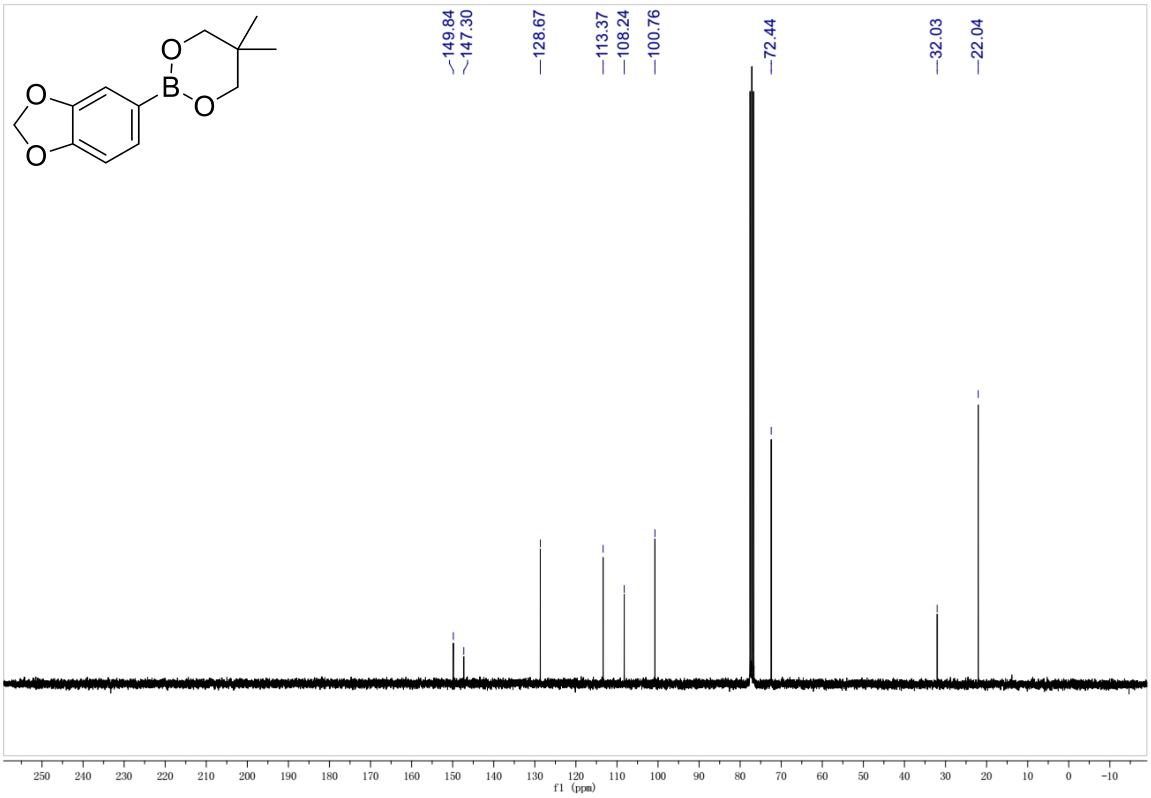

$^{13}C\{^{1}H\}$ NMR spectrum of compound **3u** in $CDCl_3$ (75 MHz).

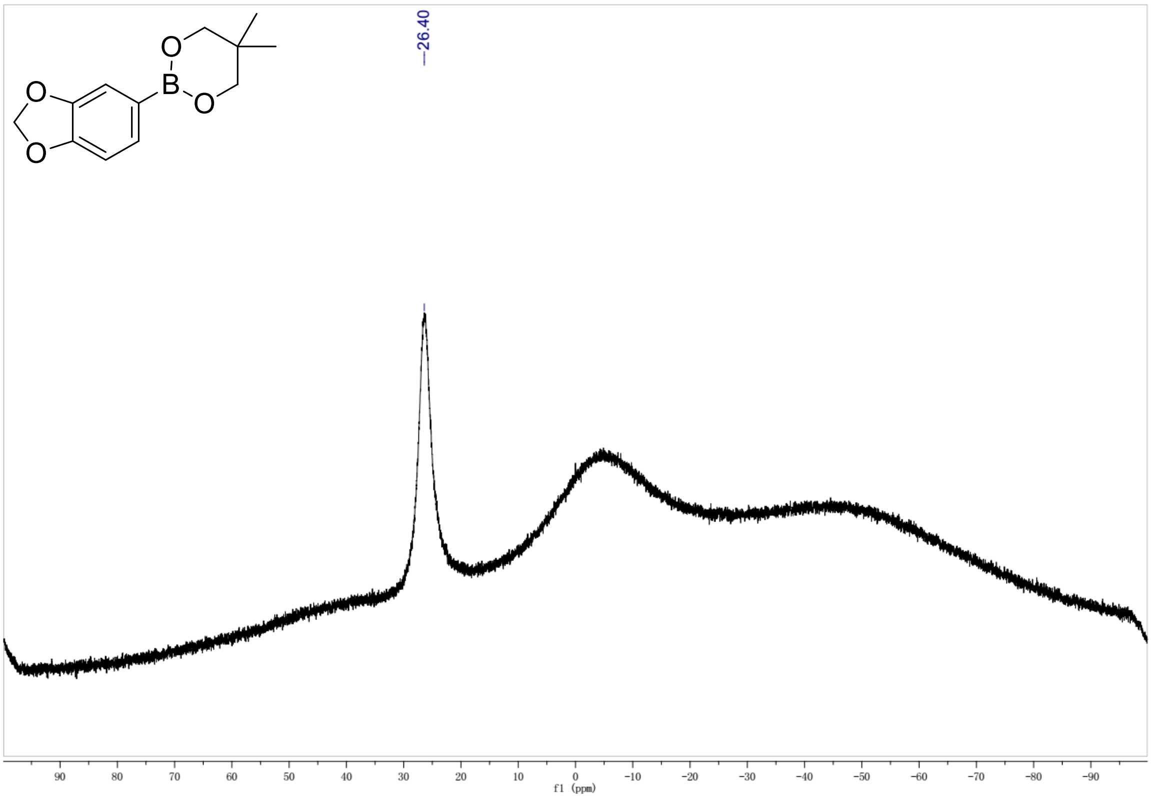

$^{11}B\{^{1}H\}$ NMR spectrum of compound **3u** in $CDCl_3$ (96 MHz).

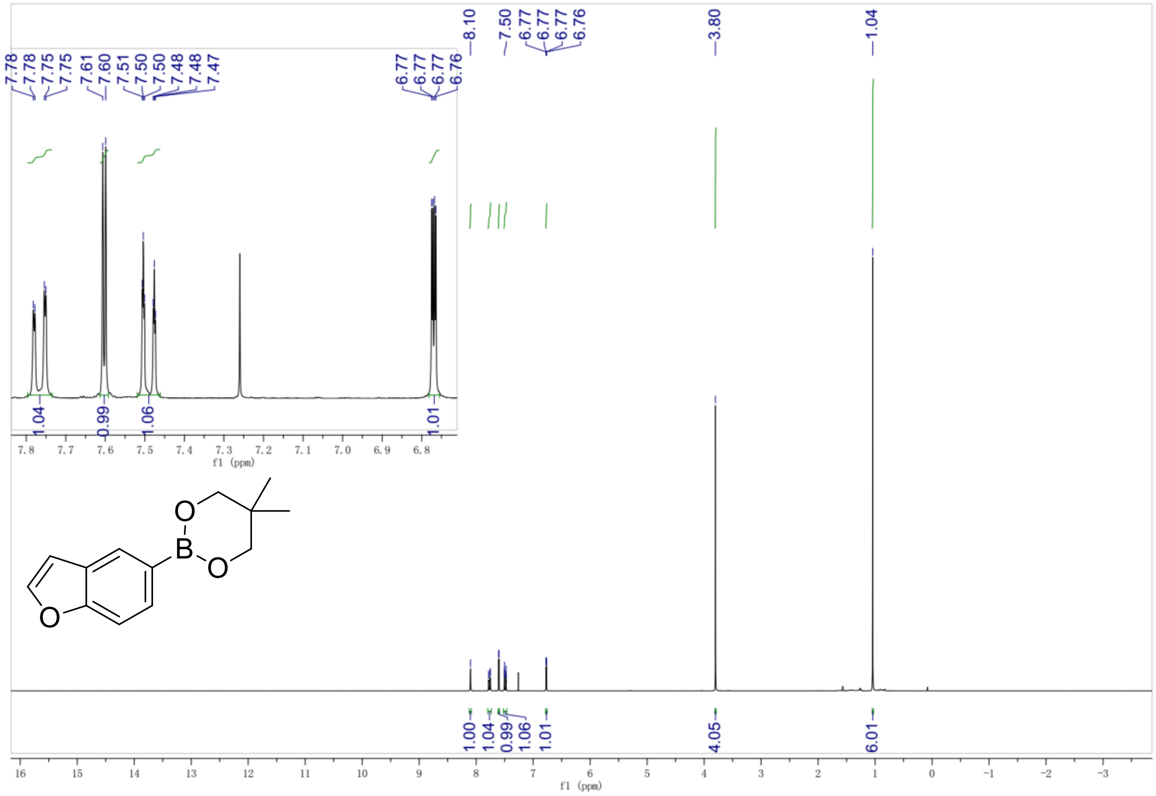

^{1}H NMR spectrum of compound **3v** in $CDCl_3$ (300 MHz).

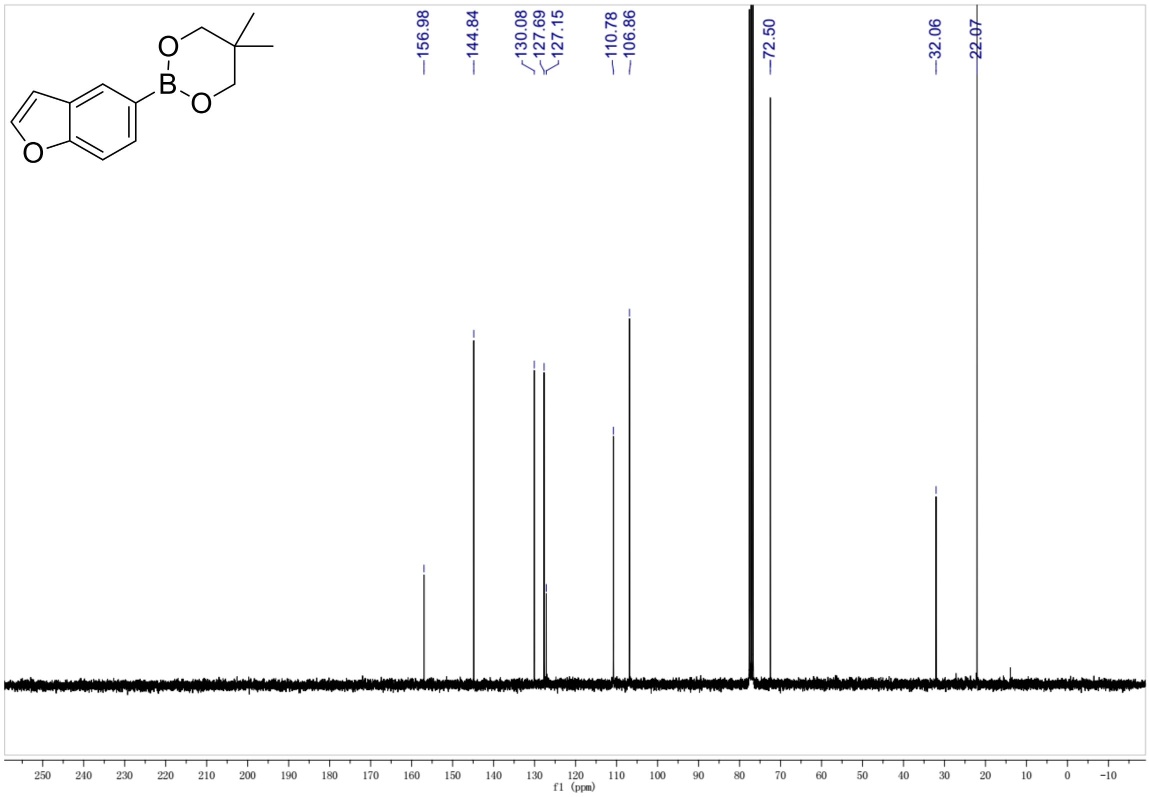

$^{13}C\{^{1}H\}$ NMR spectrum of compound **3v** in $CDCl_3$ (75 MHz).

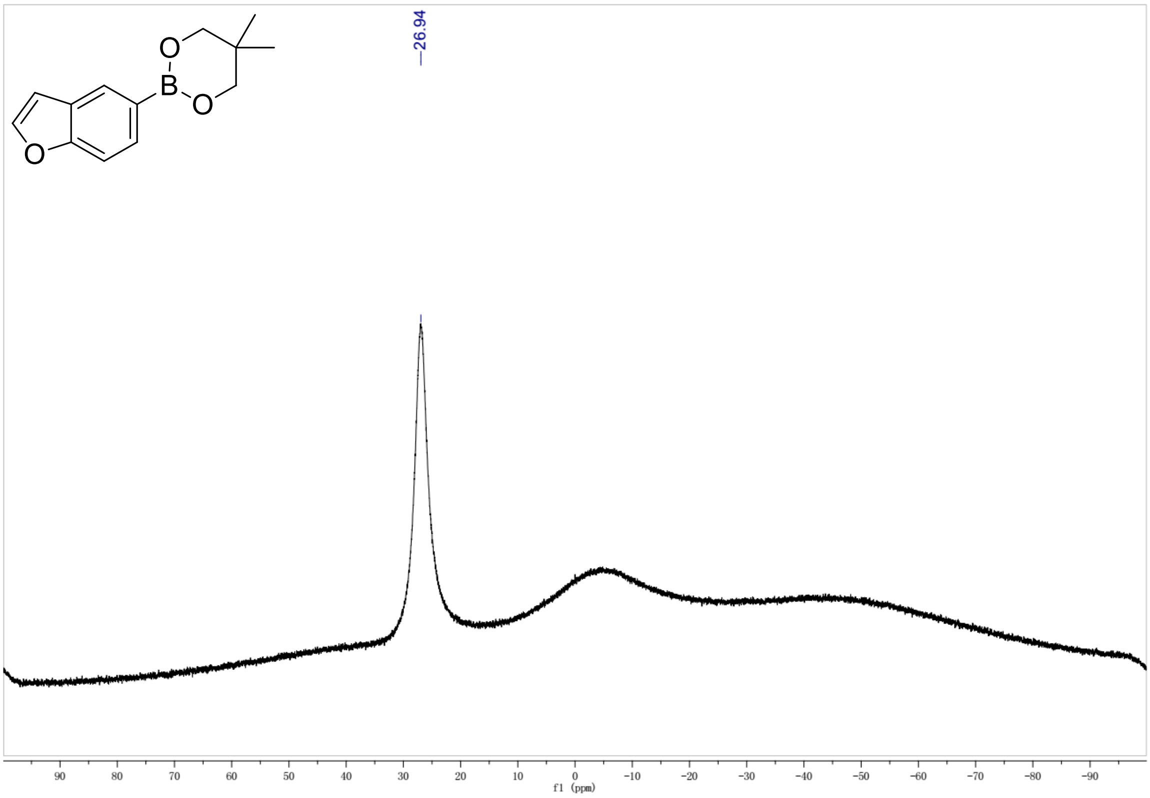

$^{11}B\{^{1}H\}$ NMR spectrum of compound **3v** in $CDCl_3$ (96 MHz).

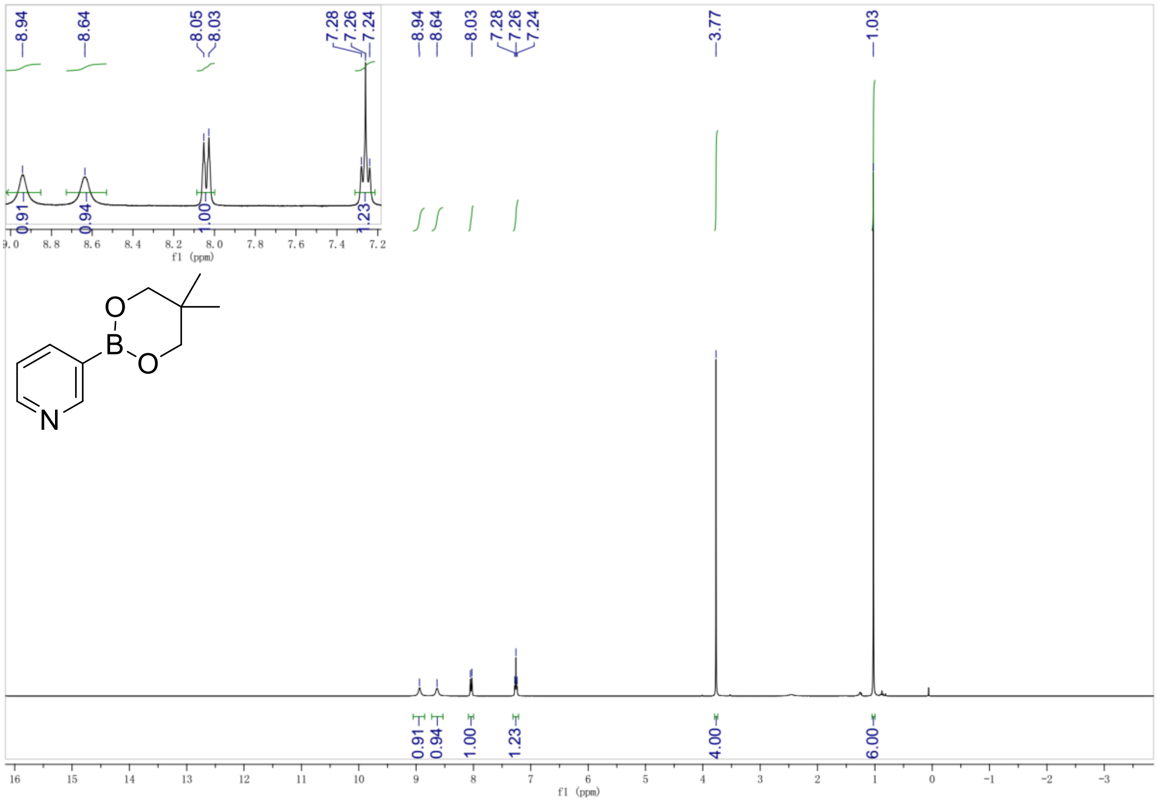

^{1}H NMR spectrum of compound **3w** in $CDCl_3$ (300 MHz).

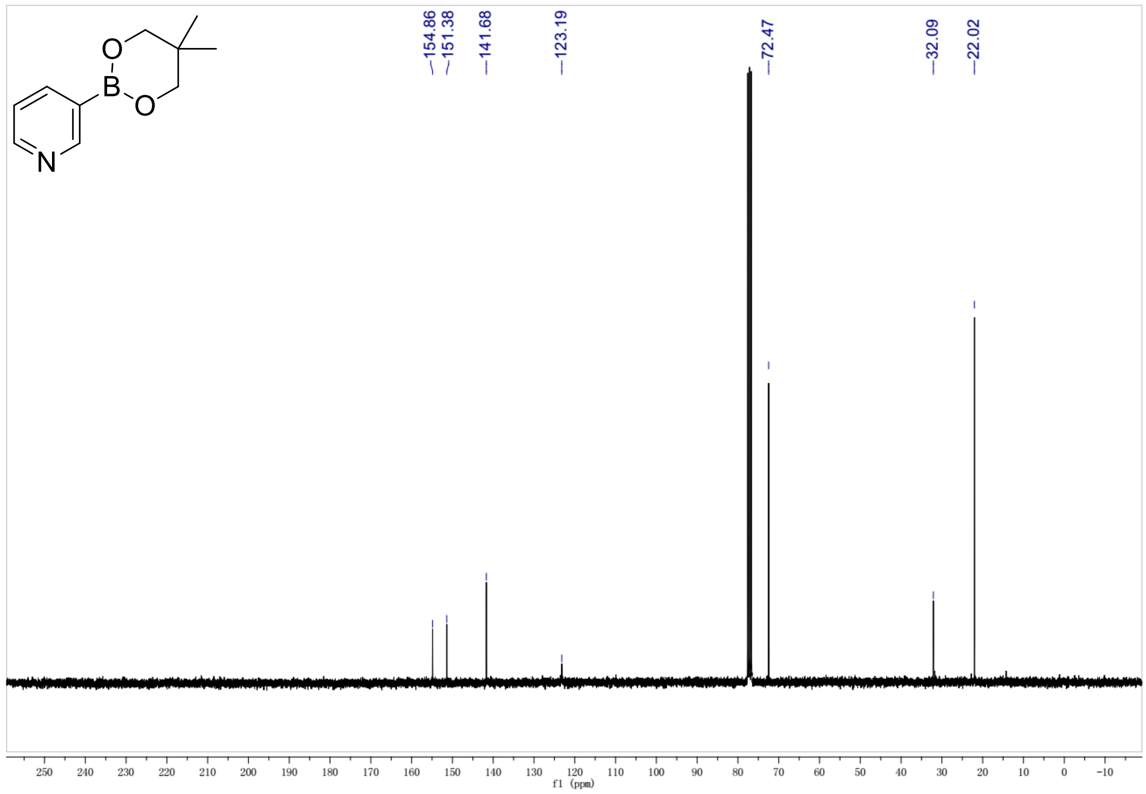

$^{13}C\{^{1}H\}$ NMR spectrum of compound **3w** in $CDCl_3$ (75 MHz).

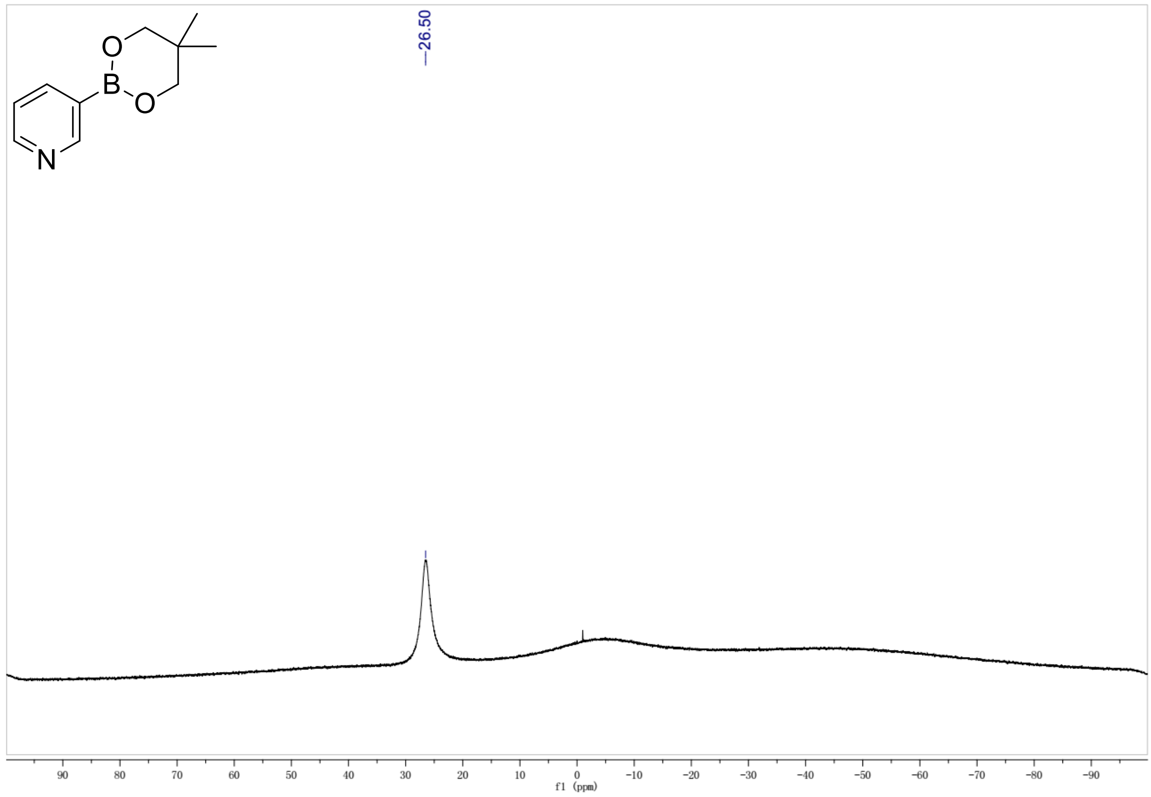

$^{11}B\{^{1}H\}$ NMR spectrum of compound **3v** in $CDCl_3$ (96 MHz).

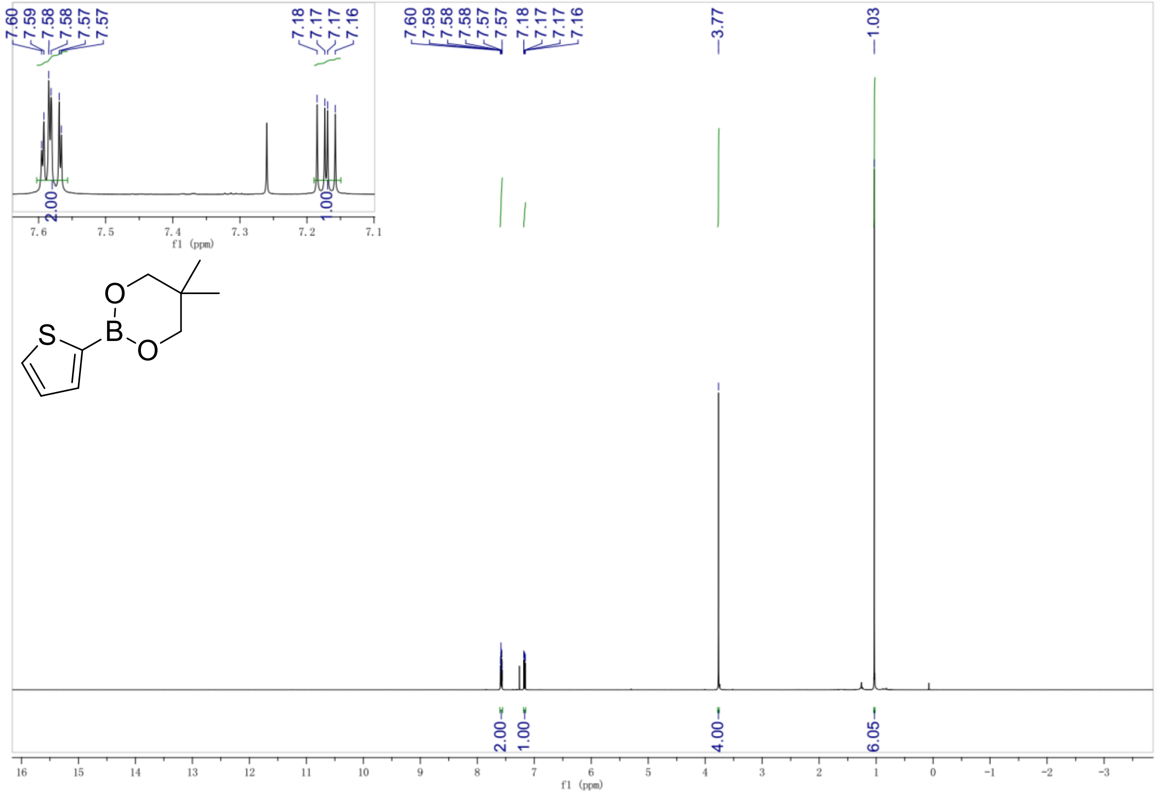

^{1}H NMR spectrum of compound **3x** in $CDCl_3$ (300 MHz).

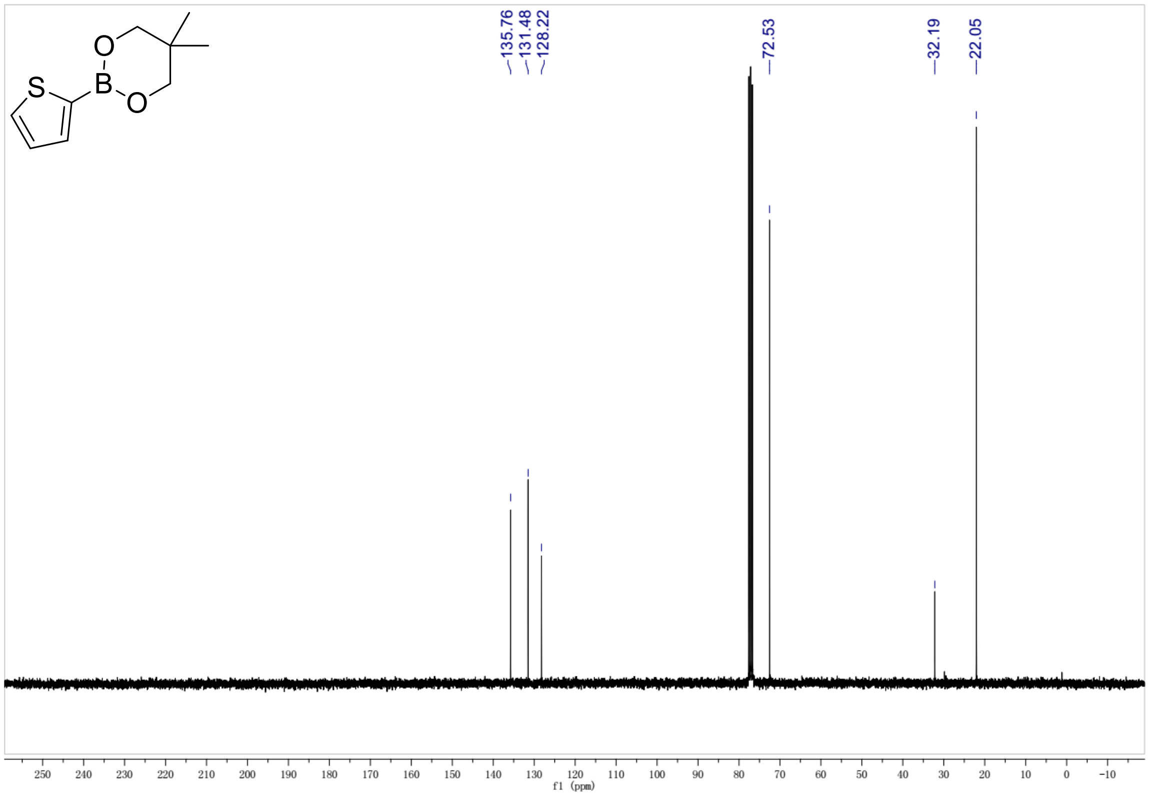

$^{13}C\{^{1}H\}$ NMR spectrum of compound **3x** in $CDCl_3$ (75 MHz).

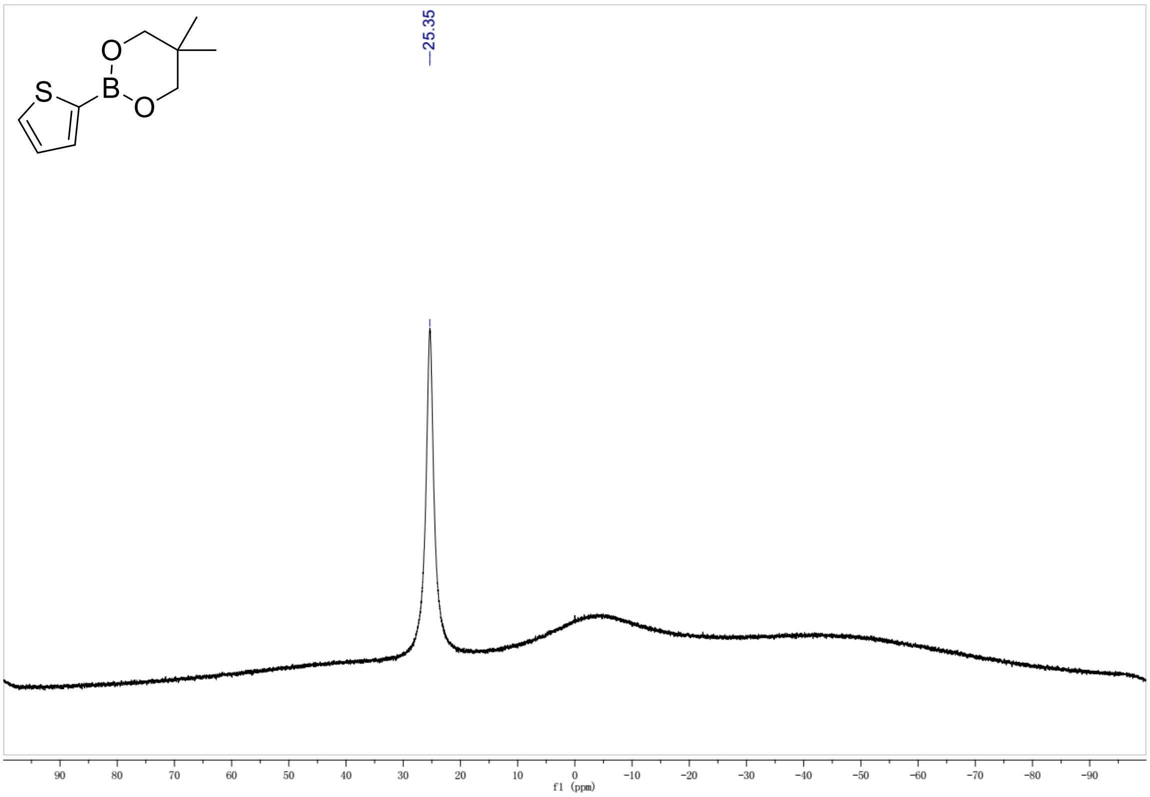

$^{11}B\{^{1}H\}$ NMR spectrum of compound **3x** in $CDCl_3$ (96 MHz).

5 Cartesian Coordinates for All Optimized Geometries

5-I

C	0.37310100	-1.77062000	2.68837900
H	0.37456100	-1.88922300	1.59613200
C	-0.50297700	-2.86758300	3.31276600
H	-1.53127700	-2.79121200	2.92211700
H	-0.56769900	-2.71222000	4.40544900
C	0.08086500	-4.25968000	3.03135500
H	-0.54044100	-5.03108400	3.51632300
H	0.02915000	-4.45691100	1.94474500
C	1.53925800	-4.37012500	3.49469600
H	1.57451800	-4.30027700	4.59827100
H	1.94590900	-5.36187300	3.23463900
C	2.40557600	-3.26056300	2.88546100
H	2.47317900	-3.39631800	1.79179100
H	3.43719000	-3.32087300	3.26928900
C	1.82881700	-1.86697500	3.16662100
H	1.87640600	-1.66092800	4.25223800
H	2.41630600	-1.09164500	2.65387900
C	-1.60825700	-2.48807000	-2.04432500
H	-1.70587200	-2.24245200	-0.97765900
C	-0.76260600	-3.76593100	-2.17439100
H	0.22525500	-3.59779100	-1.71551900
H	-0.58498800	-3.98061300	-3.24421500
C	-1.47202600	-4.96173800	-1.52496000
H	-0.86990700	-5.87499400	-1.66348500
H	-1.53469300	-4.79522700	-0.43374900
C	-2.88595500	-5.15764900	-2.08729200
H	-2.81576200	-5.44950800	-3.15193800
H	-3.39033600	-5.99150000	-1.57078500
C	-3.71919100	-3.87490800	-1.96777000
H	-3.89434200	-3.64664200	-0.90122800
H	-4.71464300	-4.01724200	-2.41989600

C	-3.01851600	-2.67612300	-2.62151400
H	-2.95336400	-2.84417900	-3.71229500
H	-3.59959400	-1.75378000	-2.46766800
C	1.03436100	1.67336700	-2.48192000
H	1.21344700	1.57826900	-1.40007900
C	0.27848100	2.97962000	-2.76853200
H	-0.68211900	2.97719900	-2.22860800
H	0.03762500	3.03455500	-3.84656500
C	1.11793400	4.19967500	-2.36343300
H	0.57576600	5.12752200	-2.61170100
H	1.24416600	4.19780700	-1.26538800
C	2.50156500	4.18905600	-3.02691100
H	2.38224200	4.32507300	-4.11824700
H	3.10044100	5.04467200	-2.67220200
C	3.23957600	2.87166300	-2.75796500
H	3.47182100	2.78816600	-1.68162900
H	4.20855400	2.85792200	-3.28350500
C	2.40189700	1.65869900	-3.18316600
H	2.26457900	1.68717700	-4.28012500
H	2.92027100	0.71934700	-2.93866300
C	-1.42216100	2.77270500	1.88670000
H	-1.45512300	2.50353600	0.82162100
C	-0.43084100	3.93344900	2.06752600
H	0.56174400	3.62390900	1.70129500
H	-0.31741000	4.15586600	3.14435300
C	-0.91598700	5.19284500	1.33685400
H	-0.21180800	6.02280500	1.51347400
H	-0.90532800	5.00518200	0.24763600
C	-2.33445800	5.58787200	1.76738600
H	-2.31908400	5.89551500	2.82975000
H	-2.67587400	6.46700300	1.19562300
C	-3.31663700	4.42271400	1.59105500
H	-3.42452800	4.19101800	0.51631600

H	-4.32077200	4.70603400	1.94740300
C	-2.84237000	3.16095700	2.32475400
H	-2.85518400	3.34642500	3.41455400
H	-3.52390000	2.31833000	2.12993800
C	-0.57005700	0.41773100	1.91672000
N	-0.95362800	1.55440800	2.55953900
C	-0.80605200	1.42207100	3.93359400
H	-1.05083400	2.21419000	4.63456500
C	-0.32050300	0.17184700	4.15415200
H	-0.06494000	-0.32774500	5.08360600
N	-0.18166500	-0.42719600	2.91086100
C	-0.37519200	-0.31431000	-1.89846600
N	-0.92121300	-1.32774800	-2.62619600
C	-0.66620000	-1.16115700	-3.97908500
H	-1.00805400	-1.86017300	-4.73614400
C	0.05827100	-0.01708900	-4.10148500
H	0.45995400	0.46656500	-4.98611300
N	0.23488400	0.48318200	-2.82068700
C	-2.40442000	0.17002100	-0.11018400
C	-3.25543200	-0.59834300	0.71694500
H	-2.82072200	-1.27773500	1.45699500
C	-4.65256200	-0.52533400	0.63911900
H	-5.26621000	-1.14605200	1.30000500
C	-5.26439900	0.34480000	-0.26868300
H	-6.35420400	0.41221400	-0.32859500
C	-4.45492000	1.12691500	-1.09830900
H	-4.91201400	1.81270600	-1.81899200
C	-3.05867700	1.02879700	-1.02220800
H	-2.46715300	1.64267400	-1.70883900
Ni	-0.48619400	0.03669600	0.01242400
O	1.44546400	-0.01109400	0.26898600
S	2.36446500	-1.11871100	-0.53061700
C	4.00492800	-0.62358700	-0.10450200

C	4.24928600	0.50796300	0.69443300
H	3.39491100	1.08010400	1.05946400
C	5.56391600	0.87059400	1.00394900
H	5.74281800	1.75194000	1.62740400
C	6.64630800	0.12354400	0.52931200
H	7.67092400	0.41282800	0.77560100
C	6.39996800	-1.00440700	-0.26608100
H	7.23611600	-1.60013800	-0.64376600
C	5.09529200	-1.37974200	-0.58220100
H	4.91592600	-2.26391700	-1.20174700

TS-iso

Ni	0.13435200	0.29592200	0.14069200
S	-0.00959000	-0.53870900	-2.19339800
O	1.41186300	-0.88480700	-1.51538200
C	-0.14059800	-1.43213800	0.99339000
C	0.32384700	-3.45157600	1.91249200
H	0.94178200	-4.26686800	2.27533800
C	-1.02792200	-3.33261400	1.85977400
H	-1.80666600	-4.02531000	2.16308600
C	0.40279100	2.04853600	-0.67005600
C	0.02417100	4.13385900	-1.47945800
H	-0.54869900	5.02081400	-1.73184300
C	1.34295300	3.85932600	-1.65632400
H	2.13289600	4.46265400	-2.09273700
C	0.21869100	1.13063900	1.85394900
C	1.46208300	1.45272900	2.43806100
H	2.39003200	1.22603400	1.90446400
C	1.56111400	2.06645100	3.69345800
H	2.54834100	2.29647900	4.10673600
C	0.40891400	2.38983800	4.41608000
H	0.48167000	2.86985300	5.39569500
C	-0.83763200	2.08943200	3.86060000
H	-1.75391400	2.33427800	4.40742800

C	-0.92386000	1.47091100	2.60556400
H	-1.92059000	1.25239500	2.21184500
C	-0.80747700	-2.08051700	-2.57317900
C	-1.99530400	-2.06685200	-3.33170000
H	-2.41882400	-1.11311400	-3.66220600
C	-2.62460700	-3.26437100	-3.67132700
H	-3.54608000	-3.24141600	-4.26042600
C	-2.07579400	-4.49262600	-3.27597000
H	-2.56683100	-5.42982500	-3.54952600
C	-0.88626900	-4.50407400	-2.53909300
H	-0.44532000	-5.45744800	-2.23302600
C	-0.24995100	-3.31025200	-2.18849200
H	0.67722300	-3.30557500	-1.61513000
N	0.84994800	-2.28314700	1.38468000
N	-1.29544700	-2.09192100	1.29975200
N	-0.53518000	3.01704200	-0.87642700
N	1.55786300	2.58319000	-1.15780900
C	-1.96492100	2.85695700	-0.59138200
C	-2.50023900	3.95684200	0.33710800
C	-2.78563400	2.76319200	-1.88893800
H	-2.02905200	1.89167900	-0.06734800
C	-3.99243000	3.74912900	0.63083200
H	-2.36127400	4.94358500	-0.14095400
H	-1.91387200	3.96417400	1.26898500
C	-4.27768600	2.56659700	-1.59119700
H	-2.64749900	3.69202800	-2.47178000
H	-2.39505300	1.93907700	-2.50724200
C	-4.81992900	3.65647400	-0.65789000
H	-4.36412500	4.56503900	1.27229500
H	-4.11993400	2.81763900	1.21257100
H	-4.84793100	2.54538700	-2.53454200
H	-4.42474600	1.57719100	-1.12048600
H	-5.87964500	3.46501000	-0.42005000

H	-4.79007800	4.63084700	-1.18002500
C	2.84808300	1.87872900	-1.23379900
C	3.24768700	1.60103200	-2.69146500
C	3.95677500	2.62745900	-0.47949400
H	2.66011500	0.90260800	-0.76646300
C	4.56688200	0.82228000	-2.74971400
H	3.35562700	2.55757400	-3.23690000
H	2.44500600	1.02512200	-3.17361700
C	5.27922900	1.85031400	-0.54374100
H	4.10379200	3.62661200	-0.92972100
H	3.65031200	2.79514600	0.56554800
C	5.68934800	1.54058800	-1.98966600
H	4.85812800	0.65636500	-3.80016700
H	4.39536700	-0.17704700	-2.31268700
H	6.07240900	2.41759100	-0.02870200
H	5.16635400	0.90170200	0.01210900
H	6.61135100	0.93509800	-2.00190300
H	5.93393200	2.48743900	-2.50704100
C	-2.65045500	-1.56098800	1.10981700
C	-3.48496800	-2.42574900	0.15228800
C	-3.37568400	-1.37387800	2.45395600
H	-2.49697500	-0.57531100	0.64384700
C	-4.88284100	-1.82635500	-0.05069000
H	-3.58413600	-3.44286400	0.57303400
H	-2.96162400	-2.52869000	-0.80858800
C	-4.77357500	-0.77466000	2.25131600
H	-3.47231600	-2.35601700	2.95111600
H	-2.76735500	-0.74195400	3.11893100
C	-5.61500700	-1.61268500	1.28012100
H	-5.47237400	-2.47599000	-0.71811700
H	-4.78682100	-0.85673100	-0.57369300
H	-5.28420900	-0.67927400	3.22388700
H	-4.67378700	0.25293100	1.85445100

H	-6.59364400	-1.13367700	1.10875800
H	-5.82687200	-2.59579400	1.74011800
C	2.29139900	-1.99533900	1.31154000
C	2.91988800	-1.97901500	2.71468100
C	3.02350000	-2.95472100	0.36108800
H	2.34653100	-0.98987900	0.87524400
C	4.42104700	-1.66996700	2.64893600
H	2.77898900	-2.96515900	3.19380100
H	2.39538800	-1.24055500	3.34137100
C	4.52781400	-2.65294500	0.32088700
H	2.86895800	-3.99827500	0.69334300
H	2.58606700	-2.83904400	-0.64084300
C	5.15714100	-2.64225900	1.71903400
H	4.85327700	-1.69831300	3.66306800
H	4.56404200	-0.63679000	2.28199500
H	5.03636300	-3.38571400	-0.32742800
H	4.67989900	-1.66694700	-0.15410100
H	6.22662400	-2.37885600	1.65735200
H	5.11272100	-3.66046600	2.14917600

5

C	0.01720400	1.66930500	0.81110800
N	-1.06639700	2.28124000	1.37203100
C	-0.79860400	3.61183600	1.65558100
H	-1.52831000	4.27897300	2.10381900
C	0.48318700	3.84423600	1.27060800
H	1.07707700	4.75154600	1.31984600
N	0.96984900	2.64871600	0.76036500
C	3.39422200	2.79723400	1.30937400
H	3.21371000	2.18569300	2.20756600
H	3.29139600	3.85257100	1.62039300
C	2.33608100	2.47478400	0.24145800
H	2.40222200	1.40684200	-0.01366400

C	-2.63378000	1.61716500	3.18312200
H	-2.69423400	2.66066500	3.54284500
H	-1.79805400	1.13712100	3.71466900
C	-3.95561000	0.90197300	3.49149600
H	-3.85151900	-0.16961900	3.24224100
H	-4.15846400	0.94660700	4.57420400
C	-5.12487700	1.49827100	2.69802100
H	-6.05296300	0.93878000	2.90268600
H	-5.30573000	2.53496300	3.03854900
C	-4.82805800	1.50543400	1.19346400
H	-5.65250600	1.97932200	0.63586700
H	-4.77097400	0.46305000	0.83033500
C	-3.50832100	2.22005200	0.87340800
H	-3.28515300	2.16742200	-0.20328900
H	-3.60671500	3.29203900	1.12572900
C	-2.34269500	1.61670200	1.67341600
H	-2.19130000	0.57630800	1.34991100
C	4.81182200	2.57159300	0.76636400
H	5.55314100	2.83748300	1.53791000
H	4.95348100	1.49493200	0.55956700
C	5.05905600	3.37049900	-0.51948600
H	6.06547300	3.15451600	-0.91512600
H	5.04360000	4.45111600	-0.28421600
C	3.99293200	3.06526000	-1.57867500
H	4.15080500	3.68361400	-2.47728800
H	4.09051900	2.01492000	-1.90771500
C	2.57131100	3.28325300	-1.04442100
H	1.83165400	2.97822500	-1.79960800
H	2.41267100	4.35741200	-0.83675700
C	0.28270000	-2.04250000	-0.23926200
N	1.40632300	-2.79646900	-0.35903700
C	1.09108600	-4.10317700	-0.70004400

H	1.84249700	-4.87137000	-0.85284200
C	-0.26226700	-4.16829600	-0.78788900
H	-0.91118700	-5.00343700	-1.03169500
N	-0.74179200	-2.89820300	-0.49771500
C	2.76880500	-2.25484600	-0.31491800
H	2.63823600	-1.21263900	0.01573600
C	3.40089700	-2.24242500	-1.71684700
H	2.72636900	-1.70620800	-2.40379500
H	3.47072300	-3.28276600	-2.08503000
C	4.80497600	-1.62806200	-1.67656900
H	5.25886100	-1.66409600	-2.68068700
H	4.72528200	-0.55605300	-1.41601500
C	5.70638500	-2.33160200	-0.65277100
H	5.89091400	-3.36992100	-0.98598000
H	6.69399000	-1.84209900	-0.60650800
C	5.06178600	-2.35767300	0.73989000
H	4.99332400	-1.32456000	1.12871600
H	5.69800800	-2.91038000	1.45098100
C	3.65593300	-2.97440100	0.71039200
H	3.73600100	-4.04373200	0.44252200
H	3.18530900	-2.92496400	1.70520000
C	-2.14046000	-2.48602100	-0.65599900
H	-2.17483200	-1.45907300	-0.25722600
C	-3.11432100	-3.35828900	0.14916900
H	-2.82848200	-3.36324800	1.21362300
H	-3.04628000	-4.40412800	-0.20078000
C	-4.55765000	-2.86466100	-0.02816200
H	-5.24851500	-3.52223300	0.52523800
H	-4.65416400	-1.86278600	0.42946600
C	-4.96020700	-2.78193800	-1.50718400
H	-4.99446400	-3.80355100	-1.92960200
H	-5.98129800	-2.37495900	-1.59962600

C	-3.96733500	-1.93544200	-2.31555100
H	-4.02662100	-0.88119500	-1.98854900
H	-4.23929200	-1.93697800	-3.38379300
C	-2.52972900	-2.43632400	-2.14254200
H	-2.44368600	-3.45543800	-2.56344300
H	-1.79755300	-1.81212600	-2.67707200
C	0.34236100	-0.72111600	2.08964600
C	1.53575800	-0.47620300	2.80138000
H	2.36067600	0.05349900	2.31392900
C	1.71676300	-0.88600400	4.12910000
H	2.66217400	-0.67385400	4.63879500
C	0.69407300	-1.55753100	4.80545100
H	0.82805400	-1.87681400	5.84258100
C	-0.50161800	-1.81635300	4.12913700
H	-1.31318700	-2.34729400	4.63716000
C	-0.66393800	-1.40926500	2.79819900
H	-1.61050900	-1.65188000	2.30411600
Ni	0.16828800	-0.17589300	0.23692200
S	0.45047900	0.36047700	-2.00355600
O	0.51493600	-0.90900500	-2.91262400
C	-0.88787300	1.35904900	-2.70647500
C	-1.27079400	2.61678700	-2.20799500
H	-0.81092100	3.02157500	-1.30585300
C	-2.24030700	3.36794000	-2.87703800
H	-2.53090600	4.34453200	-2.47885600
C	-2.83374400	2.88486400	-4.05020100
H	-3.59150800	3.47729700	-4.56928000
C	-2.43366300	1.64413000	-4.55623500
H	-2.87602300	1.26386900	-5.48181200
C	-1.46039900	0.88734900	-3.89762000
H	-1.10245600	-0.06861100	-4.28692100

6 References

[1] D. J. Krysan, P. B. Mackenzie, *J. Org. Chem.* **1990**, *55*, 4229-4230.

[2] A. J. Arduengo, S. F. Gamper, J. C. Calabrese, F. Davidson, *J. Am. Chem. Soc.* **1994**, *116*, 4391-4394.

[3] L. Kuehn, D. G. Jammal, K. Lubitz, T. B. Marder, U. Radius, *Chem. Eur. J.* **2019***, 25,* 9514-9521.

[4] W. A. Herrmann, C. Köcher, L. J. Gooßen, G. R. J. Artus, *Chem. Eur. J.* **1996**, *2*, 1627-1636.

[5] a) T. Schaub, M. Backes, U. Radius, *Organometallics* **2006**, *25*, 4196-4206; b) T. Schaub, U. Radius, A. Brucks, M. P. Choules, M. T. Olsen, T. B. Rauchfuss, *Inorg. Synth.* **2010**, *35*, 78-83; c) X. Bantreil, S. P. Nolan, *Nat. Protoc.* **2011**, *6*, 69-77.

[6] G. A. Olah, E. R. Marinez, G. K. S. Prakash, *Synlett* **1999**, *9*, 1397-1398.

[7] Y. Yoshida, S. Otsuka, K. Nogi, H. Yorimitsu, *Org. Lett.* **2018**, *20*, 1134-1137.

[8] J.-H. Chun, C. L. Morse, F. T. Chin, V. W. Pike, *Chem. Commun*. **2013**, *49*, 2151-2153.

[9] D.-L. Chen, Y. Sun, M. Chen, X. Li, L. Zhang, X. Huang, Y. Bai, F. Luo, B. Peng, *Org. Lett.* **2019**, *21*, 3986-3989.

[10] T. Hampel, S. Ruppenthal, D. Sälinger, R. Brückner, *Chem. Eur. J.* **2012**, *18*, 3136-3140.

[11] S. Ruppenthal, R. Brückner, *Eur. J. Org. Chem.* **2018**, *18*, 89-98.

[12] E. Sperotto, G. P. M. van Klink, J. G. de Vries, G. van Koten, *J. Org. Chem.* **2008**, *73*, 5625-5628.

[13] C. B. Rauhut, L. Melzig, P. Knochel, *Org. Lett.* **2008**, *10*, 3891-3894.

[14] D. A. Wilson, C. J. Wilson, C. Moldoveanu, A. M. Resmerita, P. Corcoran, L. M. Hoang, B. M. Rosen, V. Percec, *J. Am. Chem. Soc.* **2010**, *132*, 1800-1801.

[15] Y. Nagashima, R. Takita, K. Yoshida, K. Hirano, *J. Am. Chem. Soc.* **2013**, *135*, 18730-18733.

[16] M. Tobisu, H. Kinuta, Y. Kita, E. Rémond, N. Chatani, *J. Am. Chem. Soc.* **2012**, *134*, 115-118.

[17] F. Shen, H. Zheng, X. Xue, L. Lu, Q. Shen, *Org. Lett.* **2019**, *21*, 6347-6351.

[18] A. L. Barsamian, Z. Wu, P. R. Blakemore, *Org. Biomol. Chem.* **2015**, *13*, 3781-3786.

[19] C. A. Malapit, J. R. Bour, S. R. Laursen, M. S. Sanford, *J. Am. Chem. Soc.* **2019**, *141*, 17322-17330.

[20] J. Xie, K. Sekine, S. Witzel, P. Krämer, M. Rudolph, F. Rominger, A. S. K. Hashmi, *Angew. Chem., Int. Ed.* **2018**, *57*, 16648-16653; *Angew. Chem.* **2018**, *130*, 16890-16895.

[21] J. Hu, Y. Zhao, J. Liu, Y. Zhang, Z. Shi, *Angew. Chem., Int. Ed.* **2016**, *55*, 8718-8722; *Angew. Chem.* **2016**, *128*, 8860-8864.

[22] G. M. Sheldrick, *Acta Crystallogr.* **2015**, *A71*, 3-8.
[23] G. M. Sheldrick, *Acta Crystallogr.* **2008**, *A64*, 112-122.
[24] C. B. Hübschle, G. M. Sheldrick, B. Dittrich, *J. Appl. Cryst.* **2011**, *44*, 1281-1284.
[25] K. Brandenburg, Diamond (version 4.4.0), Crystal and Molecular Structure Visualization, Crystal Impact H. Putz & K. Brandenburg GbR, Bonn (Germany), **2017**.
[26] M. J. Frisch, G. W. Trucks, H. B. Schlegel, G. E. Scuseria, M. A. Robb, J. R. Cheeseman, G. Scalmani, V. Barone, B. Mennucci, G. A. Petersson, H. Nakatsuji, M. Caricato, X. Li, H. P. Hratchian, A. F. Izmaylov, J. Bloino, G. Zheng, J. L. Sonnenberg, M. Hada, M. Ehara, K. Toyota, R. Fukuda, J. Hasegawa, M. Ishida, T. Nakajima, Y. Honda, O. Kitao, H. Nakai, T. Vreven, J. J. A. Montgomery, J. E. Peralta, F. Ogliaro, M. Bearpark, J. J. Heyd, E. Brothers, K. N. Kudin, V. N. Staroverov, T. Keith, R. Kobayashi, J. Normand, K. Raghavachari, A. Rendell, J. C. Burant, S. S. Iyengar, J. Tomasi, M. Cossi, N. Rega, J. M. Millam, M. Klene, J. E. Knox, J. B. Cross, V. Bakken, C. Adamo, J. Jaramillo, R. Gomperts, R. E. Stratmann, O. Yazyev, A. J. Austin, R. Cammi, C. Pomelli, J. W. Ochterski, R. L. Martin, K. Morokuma, V. G. Zakrzewski, G. A. Voth, P. Salvador, J. J. Dannenberg, S. Dapprich, A. D. Daniels, O. Farkas, J. B. Foresman, J. V. Ortiz, J. Cioslowski, D. J. Fox, Gaussian 09, revision D.01; Gaussian, Inc.: Wallingford, CT, **2013**.
[27] a) A. D. Becke, *J. Chem. Phys.* **1993**, *98*, 5648-5652; b) C. Lee, W. Yang, R. G. Parr, *Phys. Rev. B* **1988**, *37*, 785-789.
[28] B. P. Pritchard, D. Altarawy, B. Didier, T. D. Gibson, T. L. Windus, *J. Chem. Inf. Model* **2019**, *59*, 4814-4820.
[29] a) M. Cossi, V. Barone, R. Cammi, J. Tomasi, *Chem. Phys. Lett.* **1996**, *255*, 327-335; b) E. Cancès, B. Mennucci, J. Tomasi, *J. Chem. Phys.* **1997**, *107*, 3032-3041; c) A. V. Marenich, C. J. Cramer, D. G. Truhlar, *J. Phys. Chem. B* **2009**, *113*, 6378-6396.
[30] Y. Zhao, D. G. Truhlar, *Acc. Chem. Res.* **2008**, *41*, 157-167.
[31] C. Y. Legault, CYLView, Université de Sherbrooke, Canada, **2009**.
